# Convergent Loss of ABC Transporter Genes From *Clostridioides difficile* Genomes Is Associated With Impaired Tyrosine Uptake and *p*-Cresol Production

**DOI:** 10.3389/fmicb.2018.00901

**Published:** 2018-05-08

**Authors:** Matthias Steglich, Julia D. Hofmann, Julia Helmecke, Johannes Sikorski, Cathrin Spröer, Thomas Riedel, Boyke Bunk, Jörg Overmann, Meina Neumann-Schaal, Ulrich Nübel

**Affiliations:** ^1^Leibniz Institute DSMZ-German Collection of Microorganisms and Cell Cultures, Braunschweig, Germany; ^2^German Center for Infection Research (DZIF), Braunschweig, Germany; ^3^Department of Bioinformatics and Biochemistry, Technische Universität Braunschweig, Braunschweig, Germany; ^4^Braunschweig Integrated Centre of Systems Biology (BRICS), Braunschweig, Germany

**Keywords:** *Clostridium difficile*, genome stability, repetitive DNA, transporter specificity, metabolism, metabolome, tyrosine, phenylalanine

## Abstract

We report the frequent, convergent loss of two genes encoding the substrate-binding protein and the ATP-binding protein of an ATP-binding cassette (ABC) transporter from the genomes of unrelated *Clostridioides difficile* strains. This specific genomic deletion was strongly associated with the reduced uptake of tyrosine and phenylalanine and production of derived Stickland fermentation products, including *p*-cresol, suggesting that the affected ABC transporter had been responsible for the import of aromatic amino acids. In contrast, the transporter gene loss did not measurably affect bacterial growth or production of enterotoxins. Phylogenomic analysis of publically available genome sequences indicated that this transporter gene deletion had occurred multiple times in diverse clonal lineages of *C. difficile*, with a particularly high prevalence in ribotype *027* isolates, where 48 of 195 genomes (25%) were affected. The transporter gene deletion likely was facilitated by the repetitive structure of its genomic location. While at least some of the observed transporter gene deletions are likely to have occurred during the natural life cycle of *C. difficile*, we also provide evidence for the emergence of this mutation during long-term laboratory cultivation of reference strain R20291.

## Introduction

*Clostridioides difficile* (Lawson et al., 2016) is an anaerobic gut bacterium and the leading cause of antibiotic-associated diarrhea (Martin et al., 2016). This pathogen causes a high burden of disease in Europe, with 153,000 healthcare-associated *C. difficile* infections and 8,400 ascribed deaths annually (Cassini et al., 2016). The incidence rate is similar in the United States (Martin et al., 2016).

The primary virulence factors of *C. difficile* are two enterotoxins, toxins *A* and *B*, both of which may induce inflammation and apoptosis of the host’s colonic epithelium (Aktories et al., 2017).The synthesis of these toxins is controlled by metabolic regulators that sense the bacterium’s nutritional status, suggesting that damaging the host tissue is a strategy for improving nutrient availability (Bouillaut et al., 2015). Intracellular excess of specific metabolites (glucose, amino acids) may repress toxin synthesis altogether, indicating tight regulatory linkages between *C. difficile* pathogenicity and metabolism (Karlsson et al., 2008). Recent genome-scale metabolism modeling predicted that glucose degradation and oxidative Stickland reactions may be the main sources of energy for *C. difficile* (Dannheim et al., 2017b). In Stickland fermentation, the oxidative deamination and decarboxylation of an amino acid is coupled to the reductive deamination of another amino acid molecule (Neumann-Schaal et al., 2015). However, *C. difficile* is a genetically diverse species (Knight et al., 2015), and fermentation profiles even from closely related strains may vary widely under identical growth conditions (Riedel et al., 2017). The complex relationships between genomic variation and the *C. difficile* metabolome are very little understood, even though high-throughput DNA sequencing has provided abundant genomic data in recent years. The genomes from 21 *C. difficile* strains have been fully sequenced to date (“complete” genome sequences listed at https://www.ncbi.nlm.nih.gov/genome/genomes/535). In addition, draft genome sequences from several thousand *C. difficile* isolates are available from public databases (e.g., see http://enterobase.warwick.ac.uk/). This short-read data was generated by using Illumina sequencing technology, which is widely applied for bacterial strain characterization in epidemiological investigations (Eyre et al., 2013; He et al., 2013; Steglich et al., 2015). Large-scale bacterial genome sequencing data may be used to identify correlations of specific genomic mutations with phenotypic traits of interest, provided that suitable phenotypic data is available (Laabei et al., 2014; Lees et al., 2016). Such association studies require sufficient levels of either evolutionary convergence, driven by Darwinian selection, or genetic recombination, to reduce linkage disequilibrium among genetic loci (Lees et al., 2016). Association-based discoveries could provide multiple novel insights into genome function, but to the best of our knowledge, they have not yet been reported for *C. difficile*.

Here, we report the frequent, convergent loss of specific genes encoding components of an ATP-binding cassette (ABC) transporter from the genomes of unrelated *C. difficile* strains. Our metabolomic analyses also demonstrate that these deletion mutations are associated with an impaired uptake of tyrosine and production of *p*-cresol, suggesting this ABC transporter is specifically used for the import of aromatic amino acids. Prokaryotic ABC transporters are integral membrane proteins that translocate a variety of substrates ranging from ions to macromolecules, either into the cytosol (uptake) or out of it (efflux) (Locher, 2009). They consist of a transmembrane domain protein, which forms a substrate translocation pathway across the membrane, and an ATP binding protein that couples the transport to ATP hydrolysis. In addition, ABC transporters for substrate import commonly require extracellular substrate-binding proteins, which in Gram-positive bacteria such as *C. difficile* are anchored to the cell membrane via lipid residues (Davidson et al., 2008). The substrate-binding protein determines the substrate specificity and affinity of the transporter (Locher, 2009). The genes encoding the components of ABC transporters are usually organized in operons (Davidson et al., 2008). The genome from *C. difficile* strain R20291 [sequence accession number FN545816; (Stabler et al., 2009)] carries operons for 25 binding-protein-dependent ABC transporters, seven of which currently have no specific substrate assigned. Generally, ABC transporters have important functions for bacterial physiology, viability and virulence, since they link the cellular metabolism to the extracellular environment (Davidson et al., 2008). Revealing transporter specificities and activities will ultimately improve our understanding of *C. difficile* metabolism and its interaction with the host.

## Materials and Methods

### Bacteriology

We investigated clinical *C. difficile* isolates collected from various hospitals in Germany as reported previously (Steglich et al., 2015). In addition, we used *C. difficile* strains CD-17-01474 and DSM 27147, which are independent descendants of strain NCTC 13366 (NCTC, National Collection of Type Cultures, Public Health England, United Kingdom). NCTC 13366 is a clone of ribotype *027* strain R20291, representing a large outbreak that occurred at Stoke Mandeville hospital (United Kingdom) in 2005 (Anonymous, 2006). Strain CD-17-01474 had been purchased from NCTC in 2007 and since been alternately passaged on laboratory media and stored as a glycerol stock at -80°C. In contrast, DSM 27147 was received from NCTC in 2013 through a mutual culture collection exchange between DSMZ and NCTC and deposited in the open collection at DSMZ^1^ as a freeze-dried stock.

Bacteria were cultivated on Columbia blood agar (Oxoid) plates, which were incubated anaerobically with Anaerogen packets (Oxoid) in gas tight jars. Bacterial growth curves were measured photometrically at 600 nm in 10-mL liquid cultures applying the Hungate technique and using either Wilkins Chalgren (WIC) broth (Oxoid), or yeast peptone (YP) broth, containing 5 g/L yeast (Becton Dickinson), 16 g/L peptone (Serva), and 5 g/L NaCl (Sigma) (Dawson et al., 2011). To increase the production of *p*-cresol or to enhance the effect of *p*-cresol on *C. difficile* growth, respectively, 0.1% (4-hydroxyphenyl)acetate (*p*-hydroxyphenylacetate; Sigma) and 0.1% *p*-cresol (Sigma) were added in specific experiments as indicated (Dawson et al., 2011). For intracellular and extracellular metabolome analyses, *C. difficile* was cultivated in defined casamino acids containing medium (CDMM) as described previously and harvested at half-maximal growth (Neumann-Schaal et al., 2015).

Antibiotic susceptibility was assessed by applying Etest strips (Biomérieux). For separate detection of *C. difficile* toxins *A* and *B* in supernatants from liquid cultures (WIC broth), enzyme-linked immunosorbent assays were used according to the manufacturer’s instructions (tgcBiomics). Levels of antibiotic susceptibility (minimum inhibitory concentration) and toxin production (toxin concentration in culture supernatant) were compared between isolates with and without the transporter gene deletion by applying a Mann Whitney rank sum test, implemented in SigmaPlot (Systat).

### Quantification of Amino Acids and Analyses of Metabolites

Inactivation of the bacterial metabolism (quenching) and metabolite extraction (Zech et al., 2009; Dannheim et al., 2017b), gas chromatography/mass spectrometry (GC/MS) measurements of polar metabolites, substrate uptake and fermentation products, and data processing (Neumann-Schaal et al., 2015) were performed as described previously. Statistical significance of differences between metabolite levels from two isolate groups (isolates with the ABC transporter deletion vs. isolates carrying the transporter genes) was evaluated by non-parametric Wilcoxon–Mann–Whitney test using Benjamini–Hochberg correction to control the false discovery rate (Mann and Whitney, 1947; Benjamini and Hochberg, 1995). Metabolite levels (normalized peak values) from individual isolates were compared by applying Tukey procedures implemented in the *R* package *multcomp*, version 1.4-6 (Herberich et al., 2010). The *R* function *t*-test() (R3.3.1) was used to determine mean values and 95% confidence intervals. Tyrosine and phenylalanine in the culture supernatant were quantified by liquid chromatography (HPLC) as described previously (Dannheim et al., 2017b).

### Genome Sequencing

**FIGURE 1:**
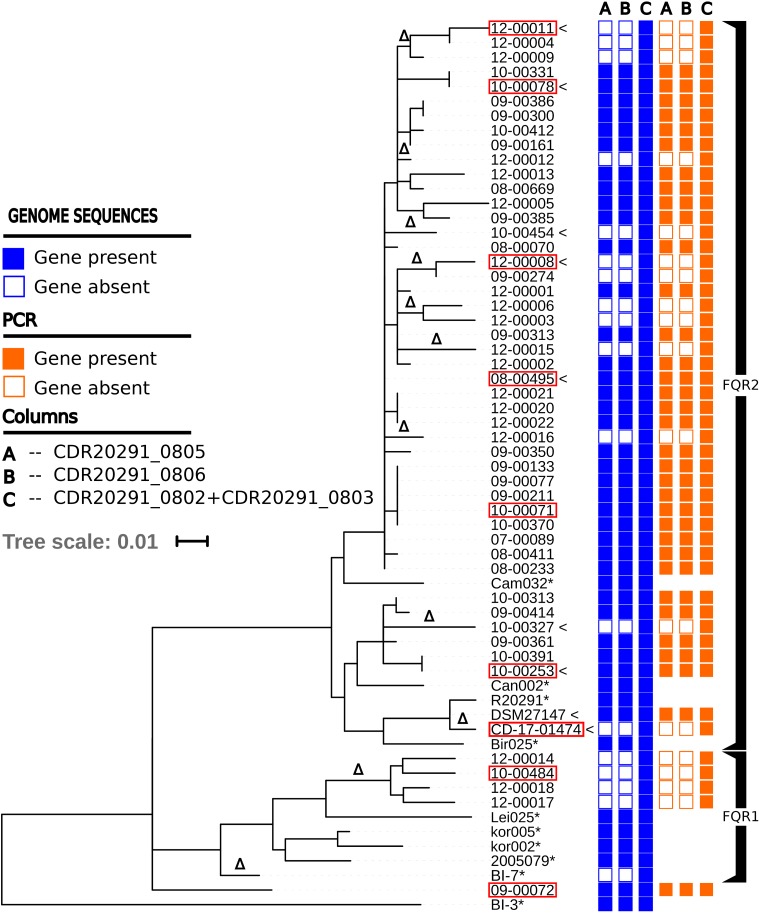
Maximum-likelihood phylogenetic tree based on core-genome SNPs from 60 ribotype *027* isolates and from strain BI-3 (He et al., 2013), which was included as an outgroup for rooting the tree. Columns on the right indicate the presence of genes encoding ABC transporter components as inferred by genome sequencing or PCR, respectively. Isolates used for metabolome analyses and growth experiments are labeled with pointy brackets. Isolates fully sequenced by a combination of SMRT and Illumina technologies are labeled with red frames. Genome sequences labeled with asterisks (^∗^) are from a previous study (He et al., 2013); they were included here for reference and were not available for PCR analysis. The symbol Δ indicates the occurrence of deletions of ABC transporter genes CDR20291_0805 and CDR20291_0806.

To generate complete genome sequences from nine *C. difficile* isolates (**Figure 1**), we applied SMRT long-read sequencing (Pacific Biosciences, United States) in combination with Illumina short-read sequencing (Illumina, United States). For preparation of SMRT sequencing libraries, 8 μg genomic DNA was sheared using g-tubes (Covaris, United States), and end-repaired and ligated to hairpin adapters applying components from the DNA Polymerase Binding Kit P6 (Pacific Biosciences, United States). Size selection to 7,000 base pairs was performed on a Blue Pippin instrument (Sage Science, United States) and libraries were sequenced on an RSII instrument (Pacific Biosciences, United States), using one SMRT cell per strain. Illumina sequencing libraries were prepared according to a previously published protocol (Baym et al., 2015), except that the concentration of TDE1 (from Illumina kit FC-121-1030) in the tagmentation reaction was reduced to 1/3. Illumina libraries were sequenced on an Illumina MiSeq machine applying a v3 reagent kit (Illumina) with 600 cycles.

For each of the genomes, 19,014–108,825 SMRT reads with mean read lengths of 7,226–10,603 base pairs were assembled using the RS_HGAP_Assembly.3 protocol implemented in SMRT Portal version 2.3.0. Illumina reads with >100-fold coverage were mapped onto the assembled sequence contigs by using BWA (Li and Durbin, 2009) to improve sequence quality to QV60. Genomes were annotated by using Prokka 1.8 software (Seemann, 2014), and annotation was corrected manually.

Fully closed genome sequences were submitted to NCBI GenBank under accession number PRJNA432093.

### Bioinformatic Analyses

Illumina sequencing read data from a total of 386 *C. difficile* genomes (**Supplementary Table S1**) were mapped to the reference genome sequence from R20291 (sequence accession number, FN545816), using BWA-MEM version 0.7.12 at default settings (Li, 2013). BAM file processing was done using Samtools version 0.1.19 (Li et al., 2009), adjusting minimum mapping quality (-Q) to 30. Samtools was also used to screen for presence or absence of the ABC transporter genes within the Illumina data sets by analyzing the mapping coverage in specific genomic regions. Consensus sequences were obtained by applying VarScan2 (v2.3) calling method mpileup2cns to the resulting BAM files (Koboldt et al., 2012), with the following parameter settings: mincoverage = 10, minfreqforhom = 0.75, minvarfrequency = 0.8, minreads2 = 6, *p*-value = 0.01, minavgqual = 20 and strandfilter = 1. Indels were detected by using ScanIndel (Yang et al., 2015; Steglich and Nübel, 2017). For discovery of insertions and deletions in fully closed genome sequences, the alignment tool Mauve was used (Darling et al., 2010). To reveal detailed structural properties of the genomic region encoding the ABC transporter in fully closed genomes, we applied MultiGeneBlast (Medema et al., 2013).

Phylogeny reconstruction was based on core-genome SNP alignment matrices generated from the mapping-based consensus sequences, excluding repetitive DNA and mobile genetic elements (Steglich et al., 2015). Maximum-likelihood phylogenetic trees were calculated under GTR-model assumption using the PhyML algorithm implemented in Seaview 4 (Guindon and Gascuel, 2003). Trees were visualized with iTOL version 4.0.3 (Letunic and Bork, 2016).

Tertiary structures of substrate-binding proteins encoded by genes CDR20291_0805 and CDR20291_0802 were predicted by applying SWISS-MODEL (Biasini et al., 2014). A substrate-binding protein from *Streptococcus pneumoniae* with the bound ligand L-tryptophan was used as template, since it displayed the highest sequence identity among proteins in the Protein Databank (PDB, available at http://www.rcsb.org/; 38% amino acid sequence identity, PDB-ID 3LFT). Structural alignment to all 501 proteins in the dataset used by Scheepers et al. (2016) was performed by using FatCat (available at http://fatcat.burnham.org/fatcat/) (Ye and Godzik, 2003). Docking of ligand L-tyrosine to substrate-binding proteins was computed by using AutoDock Vina (Li et al., 2015) and 3D molecular graphics and analyses were performed with the UCSF Chimera package (Pettersen et al., 2004).

### Polymerase Chain Reaction

Presence or absence of the specific deletion encompassing open reading frames CDR20291_0805 and CDR20291_0806 was confirmed by gene-specific PCR reactions. PCR primers were designed by using *Primer3* software, version 0.4.0^2^. Sequences of PCR primers are provided in **Supplementary Table S2**. The DreamTaq Green PCR Master Mix (Thermo Fisher Scientific) was used, and the PCR program executed 35 amplification cycles each consisting of 30 s at 95°C, 30 s at 56°C (PCR 0805, PCR 0806) or 62°C (PCR 0802-0803), respectively, and one min at 72°C.

## Results

### Convergent Loss of Transporter Genes

**Figure 1** shows a maximum-likelihood phylogenetic tree based on single-nucleotide polymorphisms in the core genomes from 61 *C. difficile* ribotype *027* isolates. The dataset includes genome sequences from 49 isolates collected in Germany (Steglich et al., 2015), two derivatives of reference strain R20291 (DSM 27147, CD-17-01474), and ten isolates from a global collection (He et al., 2013). Both fluoroquinolone-resistant phylogenetic lineages within ribotype *027*, i.e., *FQR1* and *FQR2* (He et al., 2013), are represented (**Figure 1**). Mapping of Illumina sequencing reads from 61 ribotype *027* genomes to the reference genome sequence from strain R20291 (FN545816) indicated that 18 isolates (30%) shared a specific deletion of approximately 1,889 base pairs, encompassing open reading frames CDR20291_0805 and CDR20291_0806 (**Figure 1**). Gene-specific PCR subsequently confirmed the absence of these sequences (100% consistency with Illumina sequencing results; **Figure 1**).

**FIGURE 2:**
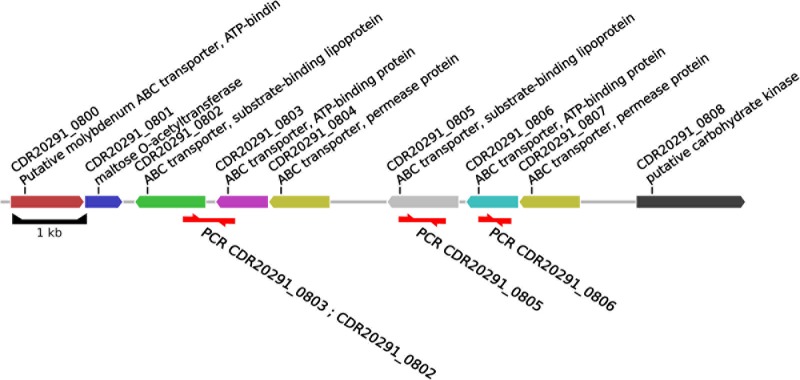
Structure of the genomic region investigated. The annotation was adopted from the genome sequence from ribotype *027* strain R20291 (accession number FN545816), position 972,100–983,373.

**FIGURE 3:**
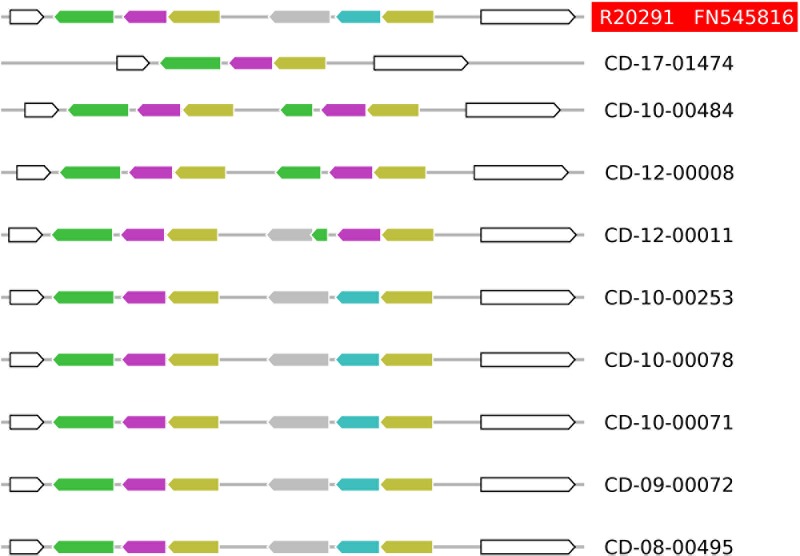
Structural variation of the ABC transporter operons in ten fully sequenced genomes from *C. difficile* ribotype *027* isolates. The color code is the same as in **Figure 2**. Five of the sequenced isolates share the structure of this genomic region with the reference strain R20291 (sequence accession number FN545816). In four isolates, genes CDR20291_0805 and CDR20291_0806 are deleted, and structural variation in this region suggested multiple, independent molecular deletion events.

According to the annotation of the R20291 genome sequence (FN545816), these deleted open reading frames encode components of an ABC transporter of unknown substrate specificity, including its substrate-binding protein (CDR20291_0805) and its ATP-binding protein (CDR20291_0806; **Figure 2**). Sequencing to completion of the genomes from nine selected *C. difficile* isolates by combining SMRT and Illumina technologies resolved the structures at this genomic region at full detail (**Figure 3**). This data confirmed the absence of open reading frames CDR20291_0805 and CDR20291_0806, and furthermore indicated replacement of these genes by duplicated copies of genes CDR20291_0802 and CDR20291_0803 in several isolates (**Figure 3**). The genes CDR20291_0802 and CDR20291_0803 are paralogous to CDR20291_0805 and CDR20291_0806, with 69 and 88% DNA sequence similarity, respectively (in R20291; **Supplementary Figure S4**). In the R20291 genome, they are located directly adjacent to CDR20291_0805 and CDR20291_0806, and, together with the gene for a permease protein (CDR20291_0804; 100% sequence identical to CDR20291_0807), they encode a highly similar ABC transporter (**Figure 2**).

**FIGURE 4:**
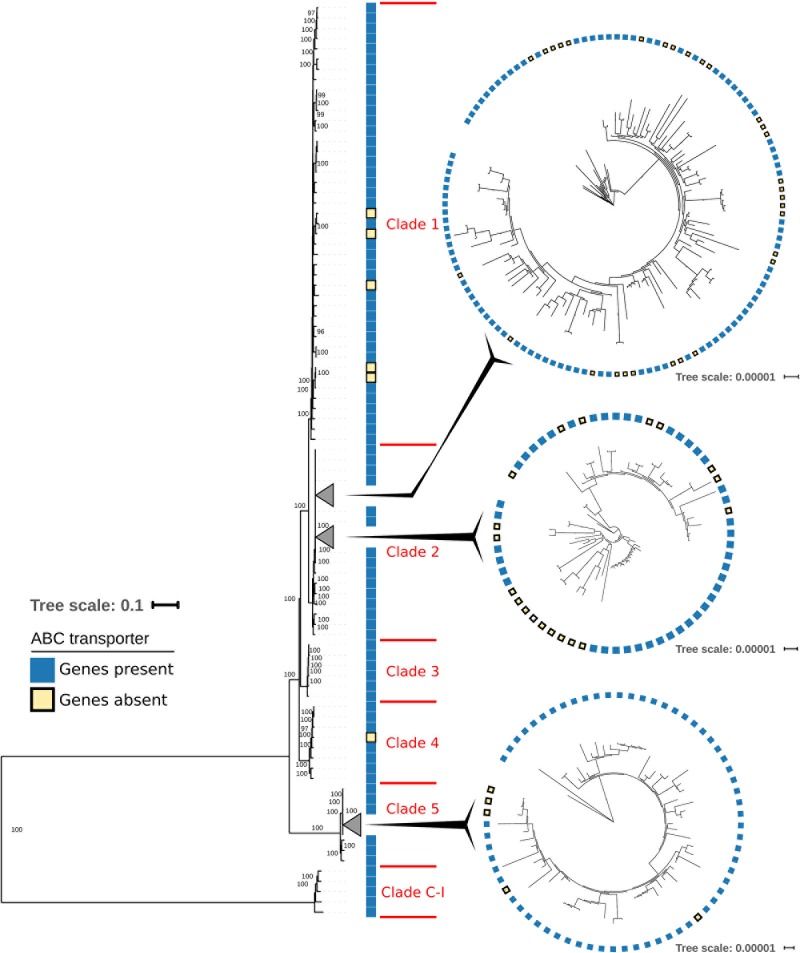
Maximum-likelihood phylogenetic tree based on core-genome SNPs from 339 *C. difficile* isolates. Sequence data had been published previously (Dingle et al., 2013; He et al., 2013; Knetsch et al., 2014) and was retrieved from GenBank (https://www.ncbi.nlm.nih.gov/genbank/). Enlargements show details of collapsed branches. Blue squares indicate the presence of ABC transporter genes CDR20291_0805 and CDR20291_0806, and yellow squares indicate the lack of these two genes.

The distribution across the phylogenetic tree of isolates lacking CDR20291_0805 and CDR20291_0806 suggests that loss of these open reading frames had occurred at least 11 times independently (**Figure 1**). Confirming this notion, sequence variation in the affected genomic region (**Figure 3**) also suggested that deletions had been generated through multiple independent molecular events. In an extended analysis, we screened previously published genome sequences from 339 *C. difficile* isolates, including international isolates affiliated to PCR ribotypes *027* (He et al., 2013) and *078* (Knetsch et al., 2014), and a recently reported dataset encompassing all phylogenetic clades within the species (Dingle et al., 2013) (**Figure 4** and **Supplementary Table S1**). Fifty-nine (17%) of these genomes lacked the genes CDR20291_0805 and CDR20291_0806, including 48 (25%) of 195 ribotype *027* isolates (**Figure 4**). This specific deletion was found in all major phylogenetic lineages except in clades 3 and C-I, which were represented by only six or five genomes each, respectively (**Figure 4**). Hence, phylogenetic analyses again indicated multiple independent loss events in distinct clonal lineages (**Figure 4**).

In some cases, isolates lacking or carrying the genes CDR20291_0805 and CDR20291_0806, respectively, were very closely related (**Figure 1**). The most extreme case is represented by the two derivatives of reference strain R20291, which differed with respect to the presence of these genes (**Figure 1**). In isolate DSM 27147 (NCTC 13366), received from NCTC in 2013, the sequence of this genomic region was identical to the published genome sequence from R20291 [FN545816; (Stabler et al., 2009)]. In contrast, in isolate CD-17-01474, purchased from NCTC in 2007 and propagated in our laboratories since then, genes CDR20291_0805 and CDR20291_0806 were deleted (**Figure 3**). In this case, obviously, gene loss had occurred during laboratory cultivation over 10 years.

### Associated Phenotypes

To identify phenotypes that may be associated with the observed gene loss, we compared a number of characteristics between *C. difficile* isolates with and without the presumptive transporter genes CDR20291_0805 and CDR20291_0806. Susceptibility to therapeutically relevant antibiotics varied only slightly among those 51 ribotype *027* isolates available to us, with minimum inhibitory concentrations ranging from 0.2 mg/L to 0.4 mg/L for metronidazole, and from 0.5 mg/L to 1.5 mg/L for vancomycin, respectively (data not shown). Hence, all isolates were fully susceptible to these drugs according to EUCAST guidelines, and the level of susceptibility was independent from the presence of those transporter genes (*P* > 0.1). Similarly, the amounts of *C. difficile* toxins *A* and *B* produced in liquid culture were unaffected by the transporter gene deletion (*P* > 0.1; not shown).

**FIGURE 5:**
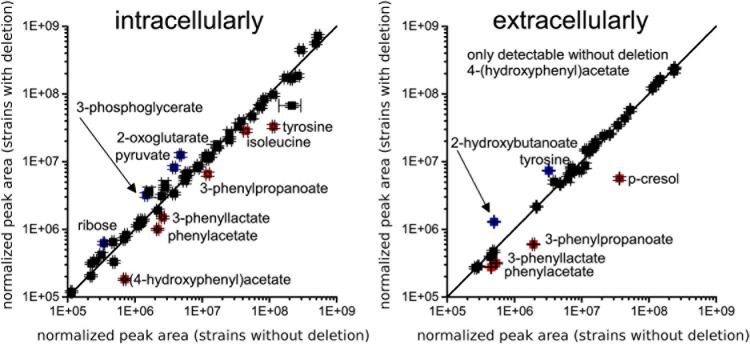
Comparison of metabolic profiles from four isolates lacking the ABC transporter genes CDR20291_0805 and CDR20291_0806 and three wildtype isolates. Scatter plots of normalized peak areas are shown; left panel, intracellular metabolome; right panel, extracellular metabolome. Metabolites depleted or increased in deletion mutants by at least 1.5-fold with adjusted *p*-values < 0.01 are labeled in red and blue, respectively. (4-hydroxyphenyl)acetate and *p*-cresol are products from fermentation of tyrosine, and phenylacetate, 3-phenyllactate and 3-phenylpropanoate are products from fermentation of phenylalanine. All metabolomic data are provided in **Supplementary Table S5**.

**FIGURE 6:**
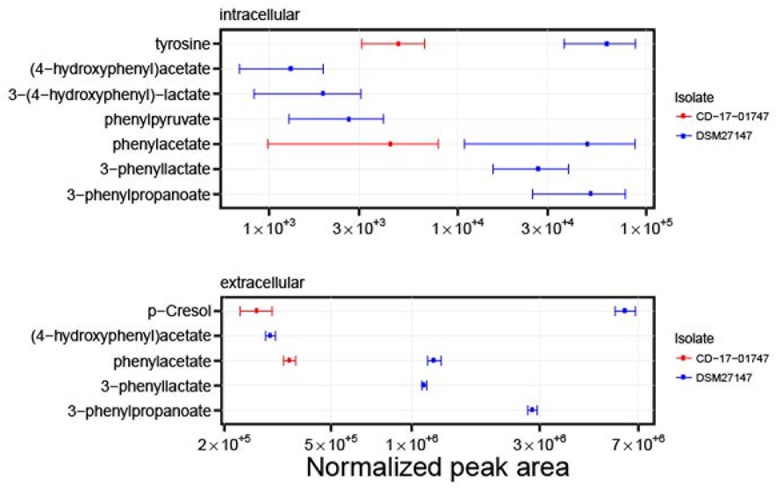
Differences in metabolite profiles from isolates CD-17-01474 and DSM 27147. Both isolates are derivatives from strain R20291, but CD-17-01474 lacks the ABC transporter genes CDR20291_0805 and CDR20291_0806. The graph shows levels of metabolites in the intracellular metabolome (three replicates, upper panel) and the extracellular metabolome (eight replicates, lower panel). Mean normalized peak areas and 95% confidence intervals are indicated. Some metabolites were not detected for isolate CD-17-01474. The results on all metabolites are shown in **Supplementary Figures S5**, **S6**.

We compared intracellular and extracellular metabolic profiles from isolates which had lost the transporter genes to those from wildtype isolates. Because our metabolomic measurements and associated data analyses were run at low throughput, we had to restrict these analyses to a total of five deletion mutants and four wildtype isolates (indicated in **Figure 1**). Aside from the transporter gene deletion, the deletion mutants displayed a limited number of additional mutations, none of which was associated with the transporter gene deletion, however (**Supplementary Tables S3**, **S4**). We found that the fermentation profiles from mutants lacking the transporter genes displayed several peculiarities (**Figure 5**). Stickland fermentation products derived from aromatic amino acids tyrosine and phenylalanine all were depleted (adjusted *P* ≤ 0.001) in deletion mutants, both intracellularly and extracellularly (**Table 1** and **Figure 5**). For example, the production of the tyrosine catabolic end product *p*-cresol was decreased by sixfold, and the intermediate fermentation product (4-hydroxyphenyl)acetate was not detectable at all (**Table 1** and **Figure 5**). At the same time, extracellular concentrations of tyrosine and phenylalanine were increased, indicating their reduced uptake (**Table 1**; absolute concentrations: tyrosine, 133 ± 12 μM vs. 83 ± 7 μM; phenylalanine, 430 ± 21 μM vs. 294 ± 36 μM; medium initially had 155 μM tyrosine, 993 μM phenylalanine). These differences were consistent and statistically significant, both in group-wise and in all pair-wise isolate comparisons (**Supplementary Figure S1**). Of note, these differences were also observed when comparing the two isolates derived from R20291, i.e., CD-17-01474 and DSM 27147, which were isogenic except for the transporter genes of interest (**Figure 6** and **Supplementary Tables S3**, **S4**). These observations indicated a strong association of the genes CDR20291_0805 and CDR20291_0806 with the uptake and fermentation of aromatic amino acids, including the production of *p*-cresol. In addition, some effects on the central carbon metabolism were observed (**Table 1**). These may be attributed to the reduced activity of both oxidative and reductive Stickland pathways for aromatic amino acids and concomitant alterations in the production and consumption of reduction equivalents.

**Table 1:** Metabolic changes in isolates lacking ABC transporter genes CDR20291_0805 and CDR20291_0806 in comparison to “wildtype isolates.”

	Fold-change	
	Extracellular	Intracellular
Tyrosine degradation		
Tyrosine	2.28	0.46
(4-hydroxyphenyl)acetate	0	0.26
*p*-cresol	0.15	0.54
**Phenylalanine degradation**		
Phenylalanine	(1.22)^∗^	(0.92)^∗^
Phenylacetate	0.61	0.46
3-Phenyllactate	0.59	0.58
3-Phenylpropanoate	0.31	0.29
**Central carbon metabolism/other fermentation pathways**		
2-Hydroxybutanoate	2.60	(0.91)^∗^
Pyruvate	ND	2.14
Ribose	ND	1.81
2-Oxoglutarate	ND	2.64
3-Phosphoglycerate	ND	2.18
Isoleucine	(1.01)^∗^	0.65

*Metabolites with fold-changes > 1.5 and adjusted p-values < 0.01 are shown. ND, not determined; ^∗^fold-change < 1.5.*

**FIGURE 7:**
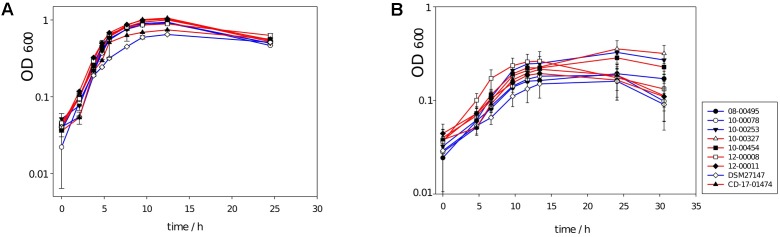
Growth curves of nine *C. difficile* isolates, four of which carry the ABC transporter genes CDR20291_0805 and CDR20291_0806 in their genomes (blue lines), and five of which lack these two genes (red lines). **(A)** Growth in WIC broth with 0.1% (4-hydroxyphenyl)acetate. **(B)** Growth in WIC broth with 0.1% (4-hydroxyphenyl)acetate and 0.1% *p*-cresol. Lowered growth rates and lowered final cell densities 0.1% *p*-cresol had been reported previously (Dawson et al., 2008).

The growth curves of deletion mutants were not different from those of wildtype isolates with respect to exponential growth rates or final cell densities after 25–30 h (*t*-test, *P* > 0.05; **Figure 7**). This result was independent from the cultivation medium used (WIC or YP broth), or whether 0.1% *p*-cresol had been added to the medium or not (**Figure 7**).

### *In Silico* Analyses of Protein Structure

The tertiary structures of substrate-binding proteins encoded by genes CDR20291_0805 and CDR20291_0802 were modeled by applying the SWISS-MODEL web server (Biasini et al., 2014) and compared to the full dataset of 501 protein structures that had previously been used for structural classification of substrate-binding proteins (Scheepers et al., 2016). Both *C. difficile* proteins were structurally most similar to proteins in Cluster B, which includes many substrate-binding proteins from amino-acid transporters (Scheepers et al., 2016). The assignment to subclusters within Cluster B was less straightforward, however, since structural similarity to multiple proteins in subclusters B-I and B-II was in a similar range of 25–27% (*P*-values < 10^-8^). Scheepers et al. (2016) had attempted to assign some substrate specificity to subclusters, but their dataset did not include any substrate-binding proteins from ABC transporters of tyrosine or any other aromatic amino acids. The substrate specificity cannot as yet be reliably predicted from the modeled protein structures, since it may depend strongly on subtle differences in the ligand binding site (Maqbool et al., 2015; Scheepers et al., 2016). Our docking analysis showed that L-tyrosine fits into the binding site of both *C. difficile* substrate-binding proteins analyzed here (RMSD = 0). The binding site is formed by nine amino acids, two of which differ between the two proteins encoded by genes CDR20291_0805 and CDR20291_0802, respectively, possibly leading to differential substrate specificity (**Supplementary Figures S2**, **S3**).

## Discussion

### Analysis of Natural Deletion Mutants Unveiled Transporter Specificity

We discovered a natural mutation that had occurred very frequently and convergently among unrelated strains of *C. difficile* (**Figures 1**, **4**). This mutation involved the deletion of two genes, encoding the substrate-binding protein and the ATP-binding protein of a putative ABC transporter, and – in a subset of genomes – their simultaneous replacement by two paralogous genes, which got duplicated in the process (**Figure 3**). The observed deletion and duplication mutation very likely got facilitated by the repetitive structure of this genomic region, formed by two directly adjacent sets of three paralogous genes each (**Figure 2** and **Supplementary Figure S4**). Such repeat elements may form DNA secondary structures, such as hairpin loops, which easily lead to strand breaks during DNA replication. During subsequent DNA repair, recombination frequently causes the removal or addition of repeat elements (Polleys et al., 2017).

The production of *p*-cresol is a unique feature of *C. difficile* and closely related organisms, and has been exploited for diagnostic purposes in the past (Sivsammye and Sims, 1990; Kuppusami et al., 2015). The frequent loss of *p*-cresol production observed here, however, questions the value of this trait for microbiological diagnostics.

It is important to note that the observed convergent nature of this mutation – the repeated, independent deletion of the same genes in unrelated isolates – enabled the identification of its association with specific phenotypes. We measured consistent phenotypic differences between deletion mutants *versus* “wildtype” isolates, even though each of these isolates carried a few additional, individual genomic peculiarities (**Supplementary Tables S3**, **S4**). Results were confirmed by comparing two isolates derived from R20291, which were isogenic except for the deletion mutation of interest plus five SNPs (four of which were either not having any effect on the encoded amino acid sequence or were located in non-coding regions) and four additional short indels, all in non-coding regions (three single-nucleotide indels, one 13-bp deletion in DSM 27147; **Supplementary Tables S3**, **S4**).

Most strikingly, the observed loss of genes encoding the substrate-binding protein and the ATP-binding protein was strongly associated with the reduced uptake of tyrosine and production of derived Stickland fermentation products, including *p*-cresol (**Figure 5** and **Table 1**). Therefore, we conclude that the ABC transporter in its wildtype form is responsible for the translocation of tyrosine from the exterior into the bacterial cytoplasm.

The ABC transporter encoded by the neighboring genes (CDR20291_0802 to CDR20291_0804) seems to have different, as yet unknown substrate specificity, which is consistent with differences among amino acids forming the ligand-binding site of the substrate-binding proteins. Overall amino-acid sequence similarity of substrate-binding proteins CDR20291_0805 and CDR20291_0802 was 69%, and interestingly, these proteins are associated with sequence-identical permease proteins in strain R20291 (**Supplementary Figure S4**). It was only recently demonstrated experimentally that the coupling of different substrate-binding proteins (with comparable sequence identity, 71%) to their reciprocal permease proteins could yield functional ABC transporters, and that the transporters’ substrate specificities were determined by the substrate-binding proteins (Teichmann et al., 2017).

Hence, our analyses of natural genomic and metabolomic variation among clinical *C. difficile* isolates revealed the substrate specificity of a binding-protein-dependent ABC transporter. Even though low-throughput, non-targeted metabolomics allowed for small sample sizes only (i.e., altogether nine isolates), the phylogeny-guided selection of isolates and relatively large effect sizes [e.g., sixfold decreased production of *p*-cresol, complete cessation of production of (4-hydroxyphenyl)acetate; **Table 1**] enabled the discovery of phenotypic effects associated with the transporter gene deletion.

### Drivers of Selection

Evolutionary convergence is considered a hallmark of Darwinian selection. Hence, the repeated, independent emergence of the observed gene loss suggested it may confer a selective advantage to the bacteria. It is unclear, however, whether this mutation may reflect an adaptation to the clinical environment or to *in vitro* growth conditions. Bacterial genome stability upon continued laboratory cultivation and long-term storage has not been explored systematically. Both, gene losses and gene duplications are frequently observed in evolution experiments with bacteria, however, suggesting that such genetic changes may provide a rapid means for adaptation to life in laboratory flasks (Tenaillon et al., 2016). Indeed, the genetic differences detected here between two isolates derived from the same parental strain R20291 obviously arose during passaging of the bacteria on laboratory culture media (**Figure 3** and **Supplementary Tables S3**, **S4**). Similarly, some genetic changes were reported for *C. difficile* strain 630, which got isolated into pure culture in 1982 and of which several variants have been reported since, including an erythromycin-susceptible mutant that can be genetically manipulated (Collery et al., 2017; Dannheim et al., 2017a). We cannot exclude that a larger proportion of the transporter gene deletions found here were laboratory-selected. In several cases, however, isolates carrying the deletion are clustered in the phylogenetic tree (**Figures 1**, **4**), even though they had been isolated in distant places and several years apart (Steglich et al., 2015), which suggests that the mutation was present already in their most recent common ancestor, and hence, that it had been formed naturally, several years before the isolates got cultivated. Therefore, at least some of the observed transporter gene deletions likely had occurred during the life cycle of *C. difficile* in the human gut.

A selective advantage may be achieved through the loss of tyrosine uptake and *p*-cresol production, or through duplication of the neighboring transporter of unknown specificity, or both. Tyrosine and phenylalanine are non-essential amino acids and among the least favored substrates for Stickland fermentation (Neumann-Schaal et al., 2015), and therefore, their uptake may not be favorable in nutrient-rich growth media. Similar constraints may apply under conditions of *C. difficile* infection, however, when nutrients are more abundant than during asymptomatic colonization due to the prior elimination of competing bacteria from the intestinal flora (Ng et al., 2013). The end product of tyrosine fermentation *p*-cresol has previously been considered useful against competitors due to its bacteriostatic properties, yet it is also self-inhibiting against *C. difficile* (Dawson et al., 2011). For a pure culture of *C. difficile*, therefore, loss of *p*-cresol formation ability could plausibly be an advantage. However, we did not observe any effect of the deletion mutation on growth rate or density in liquid cultures (**Figure 7**). Neither did the transporter-gene loss affect enterotoxin production, suggesting little effect on the bacterium’s overall nutritional status (Bouillaut et al., 2015), at least under the conditions tested.

## Author Contributions

MS and UN conceived the idea. MS, JDH, MN-S, CS, and UN performed the experiments. MS, JS, JH, TR, BB, JO, MN-S, and UN analyzed the data. MS, MN-S, and UN wrote the manuscript. All authors edited the manuscript and approved the final version.

## Conflict of Interest Statement

The authors declare that the research was conducted in the absence of any commercial or financial relationships that could be construed as a potential conflict of interest.

## Footnotes

1 www.dsmz.de

2 http://bioinfo.ut.ee/primer3-0.4.0/primer3/

## Supplementary Material

FIGURE S1 Multiple comparisons of metabolom values across strains. The filled black circles indicate the point estimators of difference between the mean of groups. 95% confidence intervals are indicated by horizontal bars and parentheses. In pairwise comparisons, if the 95% confidence interval includes zero (dashed vertical line) there is no significant difference between the group means. Conversely, if zero is not included, a significant difference is indicated. Furthermore, the more distant the 95% confidence interval is from zero, the larger the biological effect size, i.e., the real difference between the groups.

FIGURE S2 Modeled tertiary structures of the substrate-binding proteins encoded by genes CDR20291_0805 (orange) and CDR20291_0802 (blue), with L-tyrosine as ligand. The substrate-binding site is enlarged in the lower panel. Labeled amino acids differ between the two proteins and may affect their differential substrate specificity.

FIGURE S3 Alignment of the proteins encoded by *C. difficile* genes CDR20291_0805 and CDR20291_0802 with the substrate-binding protein from *Streptococcus pneumoniae* (PDB-ID, 3LFT). Shading colors indicate the hydropathy of each amino acid. Blue boxes highlight the amino acids that build the substrate-binding site. Black triangles indicate differences among amino acids within the substrate-binding site.

FIGURE S4 Alignment of genes CDR20291_0802 to CDR20291_0804 vs. CDR20291_0805 to CDR20291_0807, respectively, indicating sequence similarities.

FIGURE S5 A comparison of intracellular metabolite profiles from isolates CD-17-01474 and DSM 27147. Mean normalized peak areas and 95% confidence intervals are indicated. Some metabolites were not detected for isolate CD-17-01474.

FIGURE S6 A comparison of extracellular metabolite profiles from isolates CD-17-01474 and DSM 27147. Mean normalized peak areas and 95% confidence intervals are indicated. Some metabolites were not detected for isolate CD-17-01474.

TABLE S1 Genome sequences.

TABLE S2 PCR primers.

TABLE S3 SNP variation among genomes from nine *C. difficile* isolates used for metabolome and growth experiments.

TABLE S4 Indel variation among genomes from nine *C. difficile* isolates used for metabolome and growth experiments.

TABLE S5 Metabolome data for experiments represented in **Figures 5**, **6**.
