# Supplementary material for: Convergent Loss of ABC Transporter Genes From Clostridioides difficile Genomes Is Associated With Impaired Tyrosine Uptake and p-Cresol Production
Source: Front Microbiol. 2018 May 8;9:901. doi: 10.3389/fmicb.2018.00901 (PMC5951980; doi:10.3389/fmicb.2018.00901)
Supplement: FIGURE S1 — Multiple comparisons of metabolom values across strains. The filled black circles indicate the point estimators of difference between the mean of groups. 95% confidence intervals are indicated by horizontal bars and parentheses. In pairwise comparisons, if the 95% confidence interval includes zero (dashed vertical line) there is no significant difference between the group means. Conversely, if zero is not included, a significant difference is indicated. Furthermore, the more distant the 95% confidence interval is from zero, the larger the biological effect size, i.e., the real difference between the groups. [file Image_1.PDF]

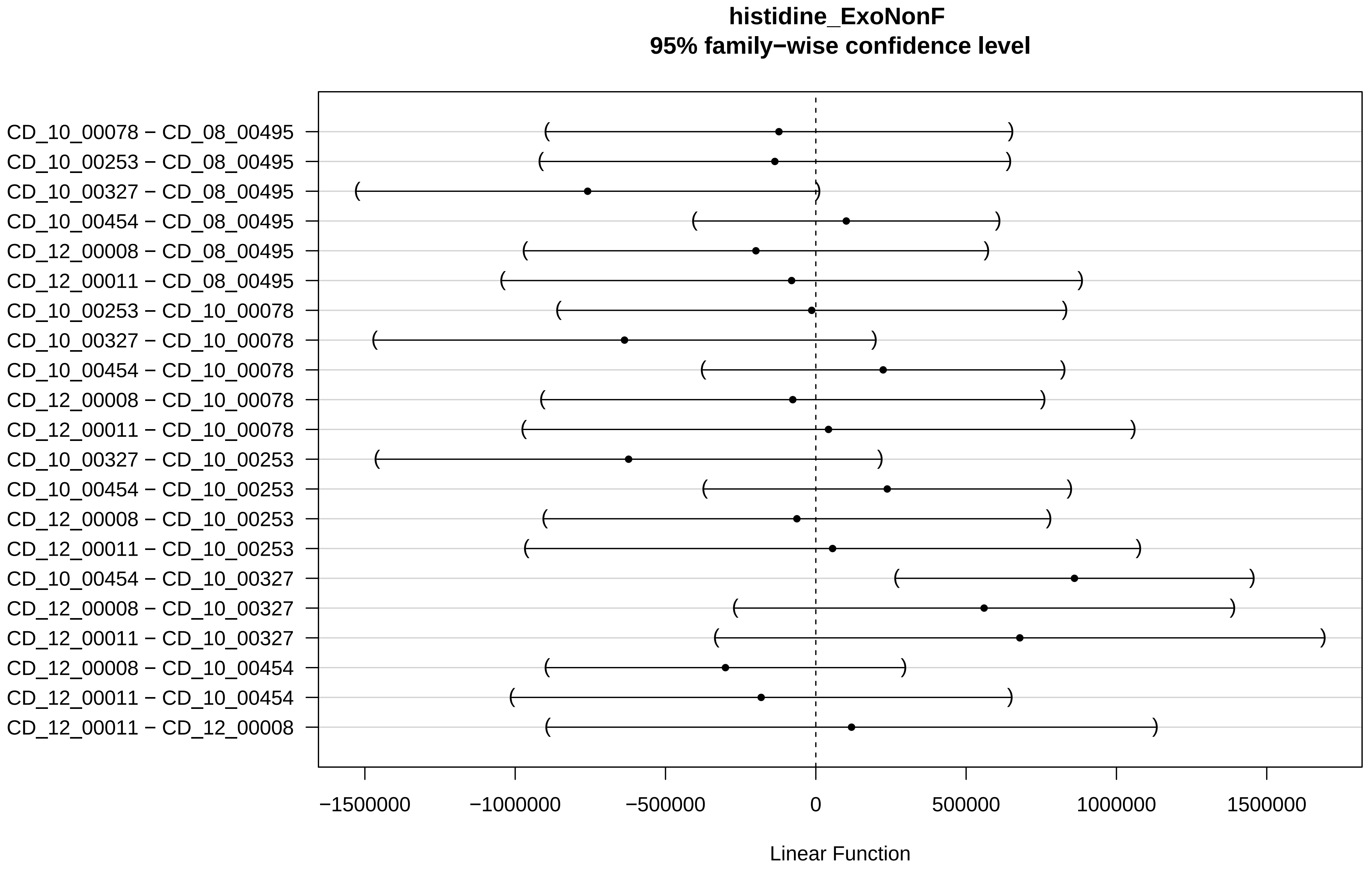
histidine_ExoNonF
95% family−wise confidence level
CD_10_00078 − CD_08_00495
CD_10_00253 − CD_08_00495
CD_10_00327 − CD_08_00495
CD_10_00454 − CD_08_00495
CD_12_00008 − CD_08_00495
CD_12_00011 − CD_08_00495
CD_10_00253 − CD_10_00078
CD_10_00327 − CD_10_00078
CD_10_00454 − CD_10_00078
CD_12_00008 − CD_10_00078
CD_12_00011 − CD_10_00078
CD_10_00327 − CD_10_00253
CD_10_00454 − CD_10_00253
CD_12_00008 − CD_10_00253
CD_12_00011 − CD_10_00253
CD_10_00454 − CD_10_00327
CD_12_00008 − CD_10_00327
CD_12_00011 − CD_10_00327
CD_12_00008 − CD_10_00454
CD_12_00011 − CD_10_00454
CD_12_00011 − CD_12_00008
−1500000
−1000000
−500000
0
500000
1000000
1500000
Linear Function

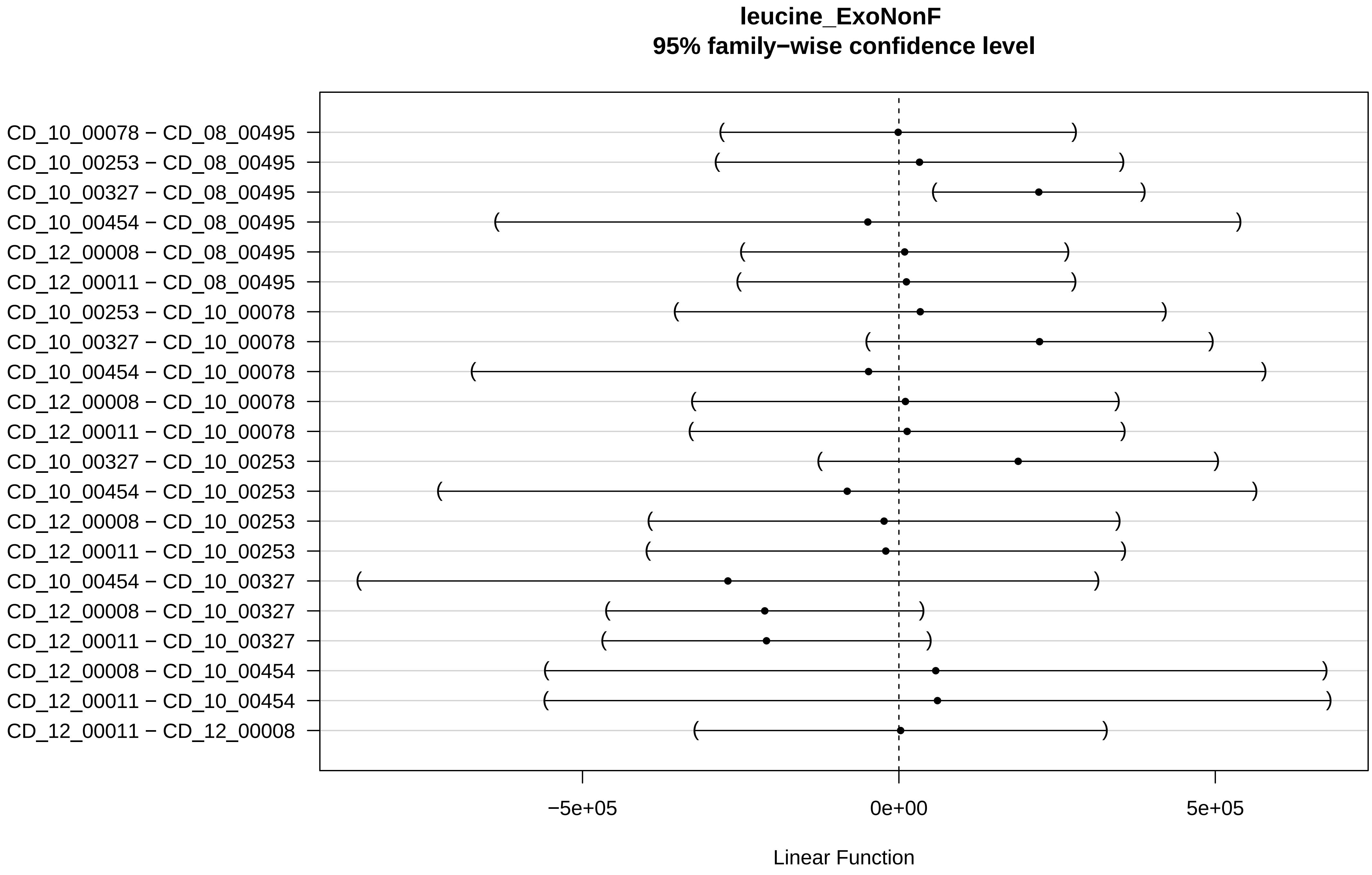

leucine_ExoNonF
95% family−wise confidence level
CD_10_00078 − CD_08_00495
CD_10_00253 − CD_08_00495
CD_10_00327 − CD_08_00495
CD_10_00454 − CD_08_00495
CD_12_00008 − CD_08_00495
CD_12_00011 − CD_08_00495
CD_10_00253 − CD_10_00078
CD_10_00327 − CD_10_00078
CD_10_00454 − CD_10_00078
CD_12_00008 − CD_10_00078
CD_12_00011 − CD_10_00078
CD_10_00327 − CD_10_00253
CD_10_00454 − CD_10_00253
CD_12_00008 − CD_10_00253
CD_12_00011 − CD_10_00253
CD_10_00454 − CD_10_00327
CD_12_00008 − CD_10_00327
CD_12_00011 − CD_10_00327
CD_12_00008 − CD_10_00454
CD_12_00011 − CD_10_00454
CD_12_00011 − CD_12_00008
−5e+05
0e+00
5e+05
Linear Function

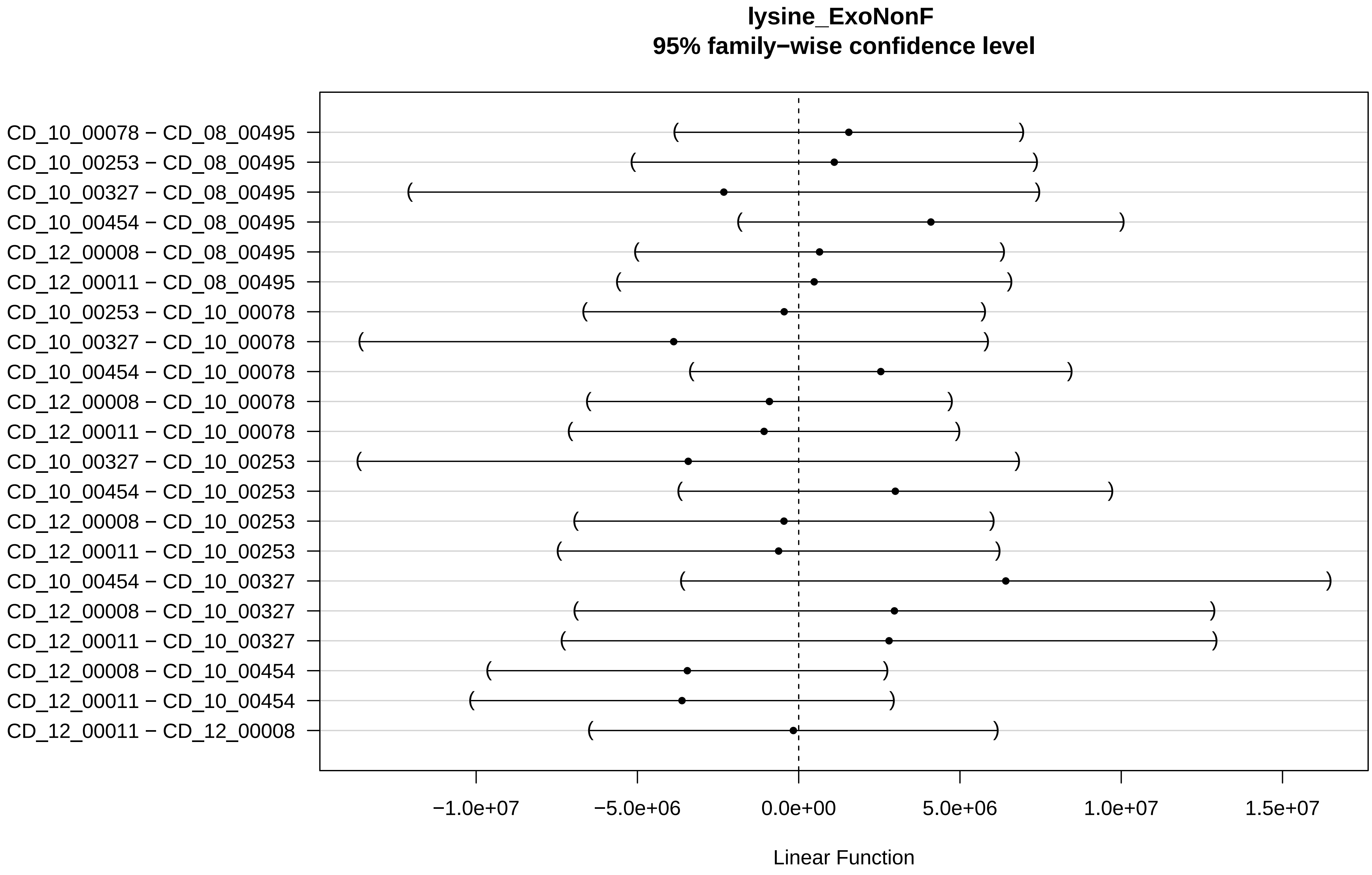
lysine_ExoNonF
95% family−wise confidence level
CD_10_00078 − CD_08_00495
CD_10_00253 − CD_08_00495
CD_10_00327 − CD_08_00495
CD_10_00454 − CD_08_00495
CD_12_00008 − CD_08_00495
CD_12_00011 − CD_08_00495
CD_10_00253 − CD_10_00078
CD_10_00327 − CD_10_00078
CD_10_00454 − CD_10_00078
CD_12_00008 − CD_10_00078
CD_12_00011 − CD_10_00078
CD_10_00327 − CD_10_00253
CD_10_00454 − CD_10_00253
CD_12_00008 − CD_10_00253
CD_12_00011 − CD_10_00253
CD_10_00454 − CD_10_00327
CD_12_00008 − CD_10_00327
CD_12_00011 − CD_10_00327
CD_12_00008 − CD_10_00454
CD_12_00011 − CD_10_00454
CD_12_00011 − CD_12_00008
−1.0e+07
−5.0e+06
0.0e+00
5.0e+06
1.0e+07
1.5e+07
Linear Function

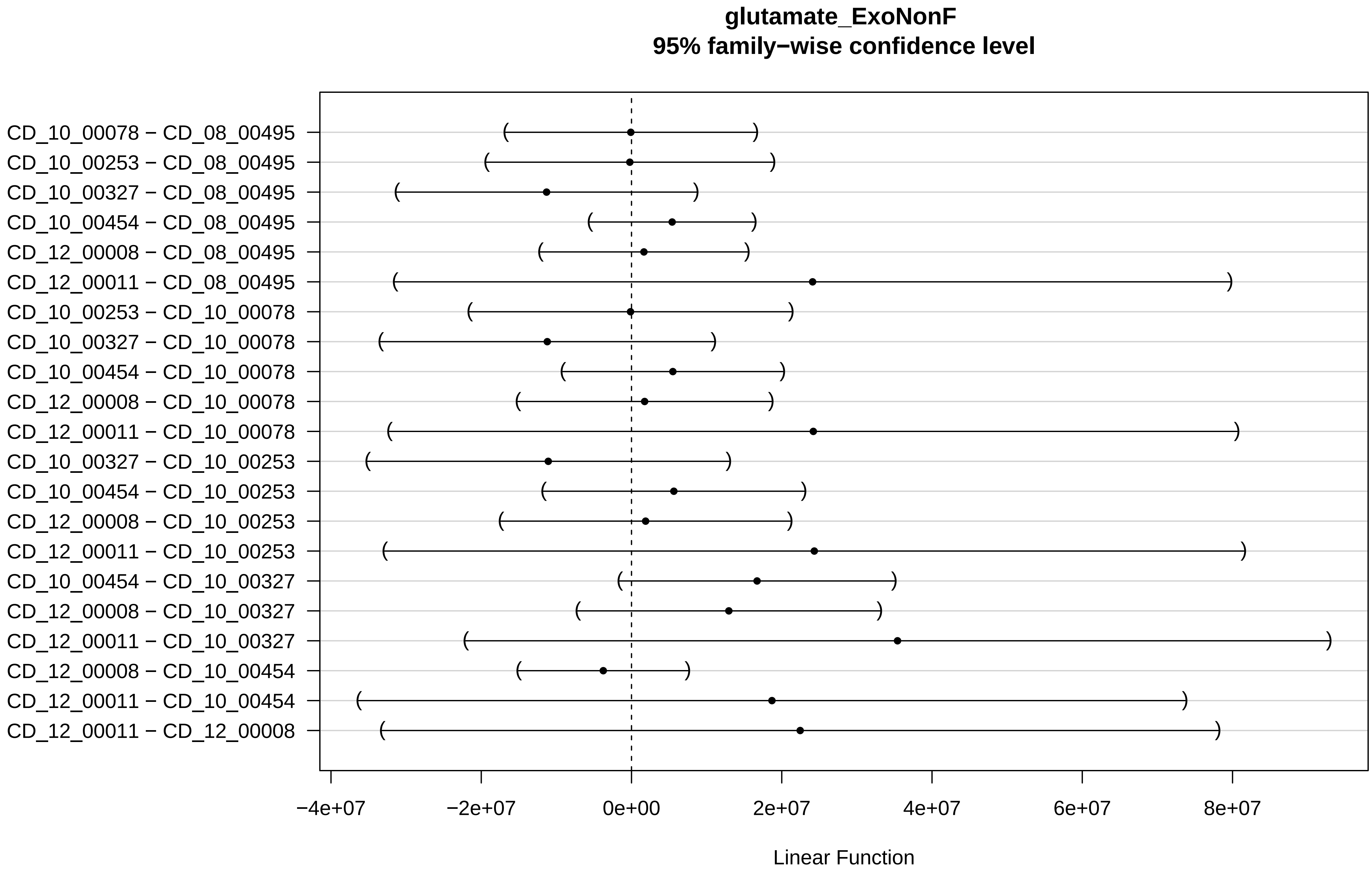
glutamate_ExoNonF
95% family−wise confidence level
CD_10_00078 − CD_08_00495
CD_10_00253 − CD_08_00495
CD_10_00327 − CD_08_00495
CD_10_00454 − CD_08_00495
CD_12_00008 − CD_08_00495
CD_12_00011 − CD_08_00495
CD_10_00253 − CD_10_00078
CD_10_00327 − CD_10_00078
CD_10_00454 − CD_10_00078
CD_12_00008 − CD_10_00078
CD_12_00011 − CD_10_00078
CD_10_00327 − CD_10_00253
CD_10_00454 − CD_10_00253
CD_12_00008 − CD_10_00253
CD_12_00011 − CD_10_00253
CD_10_00454 − CD_10_00327
CD_12_00008 − CD_10_00327
CD_12_00011 − CD_10_00327
CD_12_00008 − CD_10_00454
CD_12_00011 − CD_10_00454
CD_12_00011 − CD_12_00008
−4e+07
−2e+07
0e+00
2e+07
4e+07
6e+07
8e+07
Linear Function

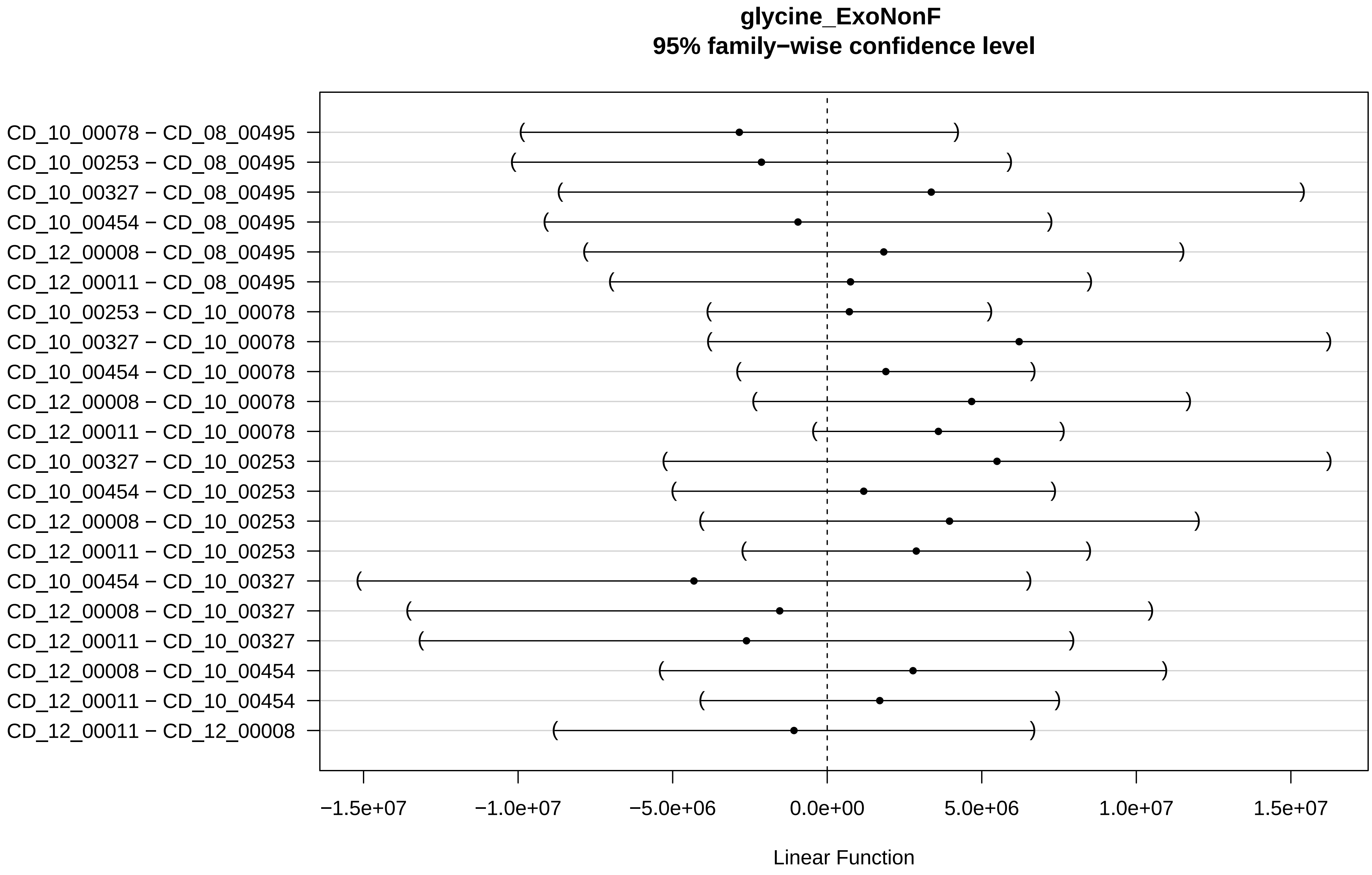
glycine_ExoNonF
95% family−wise confidence level
CD_10_00078 − CD_08_00495
CD_10_00253 − CD_08_00495
CD_10_00327 − CD_08_00495
CD_10_00454 − CD_08_00495
CD_12_00008 − CD_08_00495
CD_12_00011 − CD_08_00495
CD_10_00253 − CD_10_00078
CD_10_00327 − CD_10_00078
CD_10_00454 − CD_10_00078
CD_12_00008 − CD_10_00078
CD_12_00011 − CD_10_00078
CD_10_00327 − CD_10_00253
CD_10_00454 − CD_10_00253
CD_12_00008 − CD_10_00253
CD_12_00011 − CD_10_00253
CD_10_00454 − CD_10_00327
CD_12_00008 − CD_10_00327
CD_12_00011 − CD_10_00327
CD_12_00008 − CD_10_00454
CD_12_00011 − CD_10_00454
CD_12_00011 − CD_12_00008
−1.5e+07
−1.0e+07
−5.0e+06
0.0e+00
5.0e+06
1.0e+07
1.5e+07
Linear Function

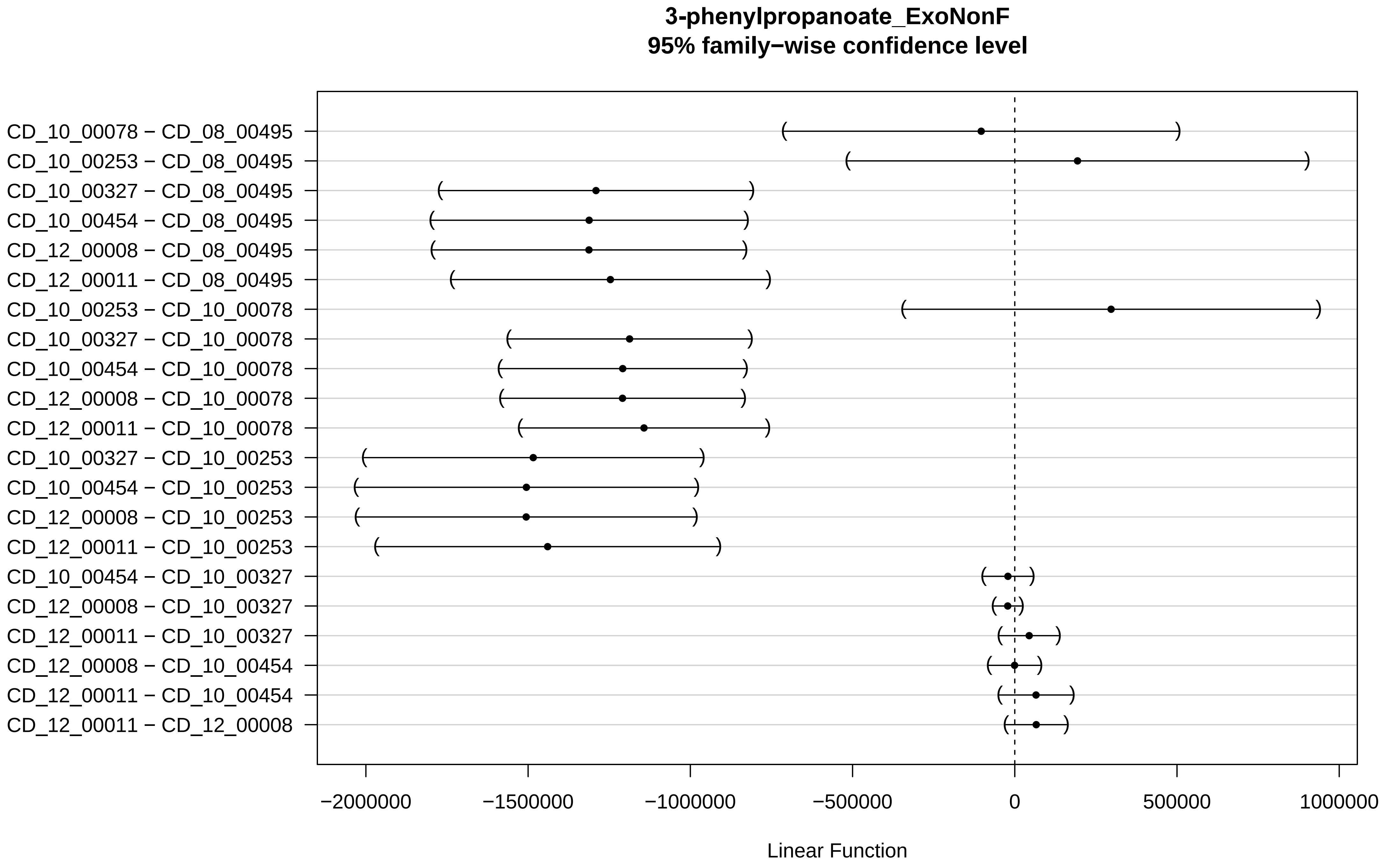
3-phenylpropanoate_ExoNonF
95% family−wise confidence level
CD_10_00078 − CD_08_00495
CD_10_00253 − CD_08_00495
CD_10_00327 − CD_08_00495
CD_10_00454 − CD_08_00495
CD_12_00008 − CD_08_00495
CD_12_00011 − CD_08_00495
CD_10_00253 − CD_10_00078
CD_10_00327 − CD_10_00078
CD_10_00454 − CD_10_00078
CD_12_00008 − CD_10_00078
CD_12_00011 − CD_10_00078
CD_10_00327 − CD_10_00253
CD_10_00454 − CD_10_00253
CD_12_00008 − CD_10_00253
CD_12_00011 − CD_10_00253
CD_10_00454 − CD_10_00327
CD_12_00008 − CD_10_00327
CD_12_00011 − CD_10_00327
CD_12_00008 − CD_10_00454
CD_12_00011 − CD_10_00454
CD_12_00011 − CD_12_00008
−2000000
−1500000
−1000000
−500000
0
500000
1000000
Linear Function

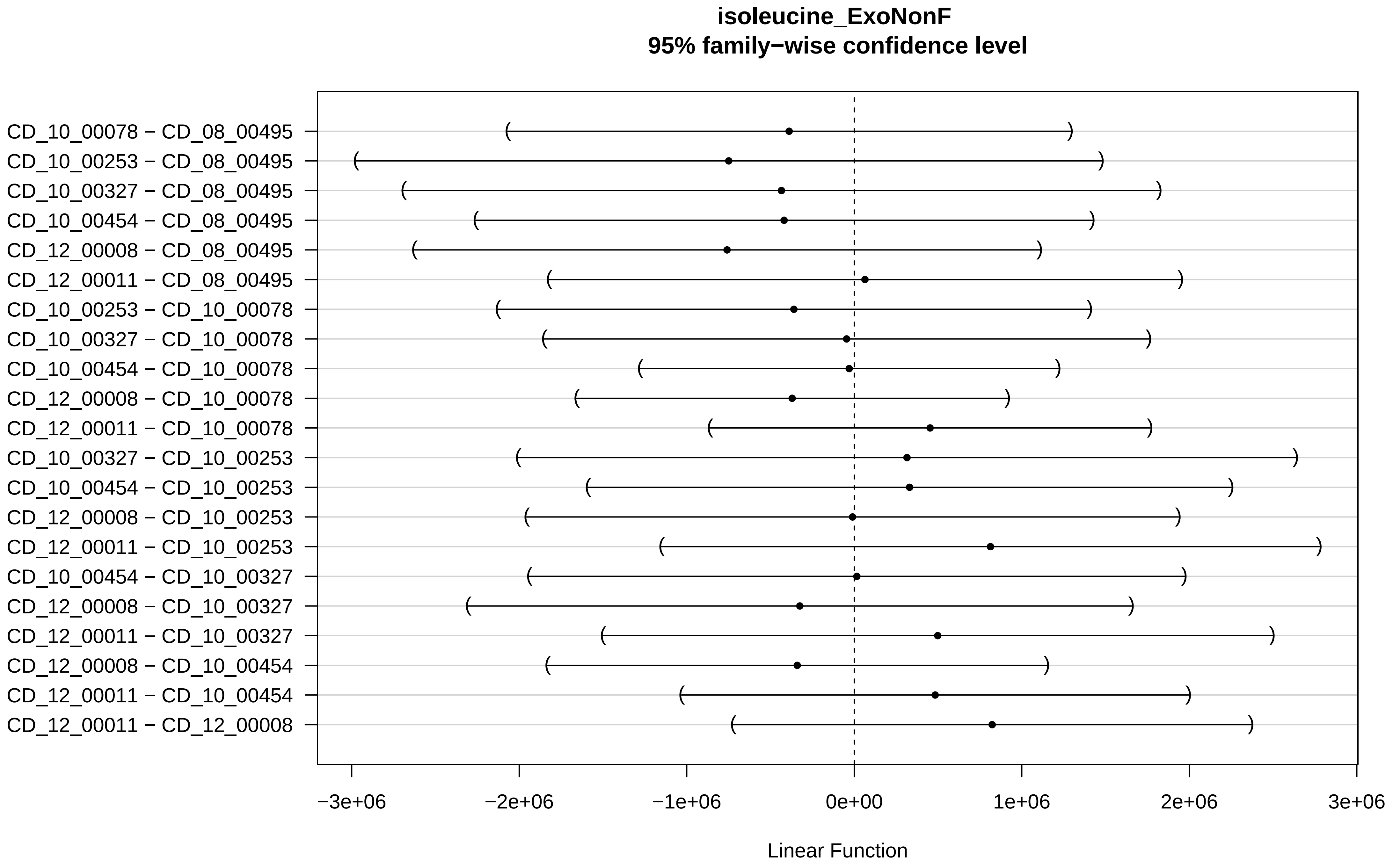

isoleucine_ExoNonF
95% family−wise confidence level
CD_10_00078 − CD_08_00495
CD_10_00253 − CD_08_00495
CD_10_00327 − CD_08_00495
CD_10_00454 − CD_08_00495
CD_12_00008 − CD_08_00495
CD_12_00011 − CD_08_00495
CD_10_00253 − CD_10_00078
CD_10_00327 − CD_10_00078
CD_10_00454 − CD_10_00078
CD_12_00008 − CD_10_00078
CD_12_00011 − CD_10_00078
CD_10_00327 − CD_10_00253
CD_10_00454 − CD_10_00253
CD_12_00008 − CD_10_00253
CD_12_00011 − CD_10_00253
CD_10_00454 − CD_10_00327
CD_12_00008 − CD_10_00327
CD_12_00011 − CD_10_00327
CD_12_00008 − CD_10_00454
CD_12_00011 − CD_10_00454
CD_12_00011 − CD_12_00008
−3e+06
−2e+06
−1e+06
0e+00
1e+06
2e+06
3e+06
Linear Function

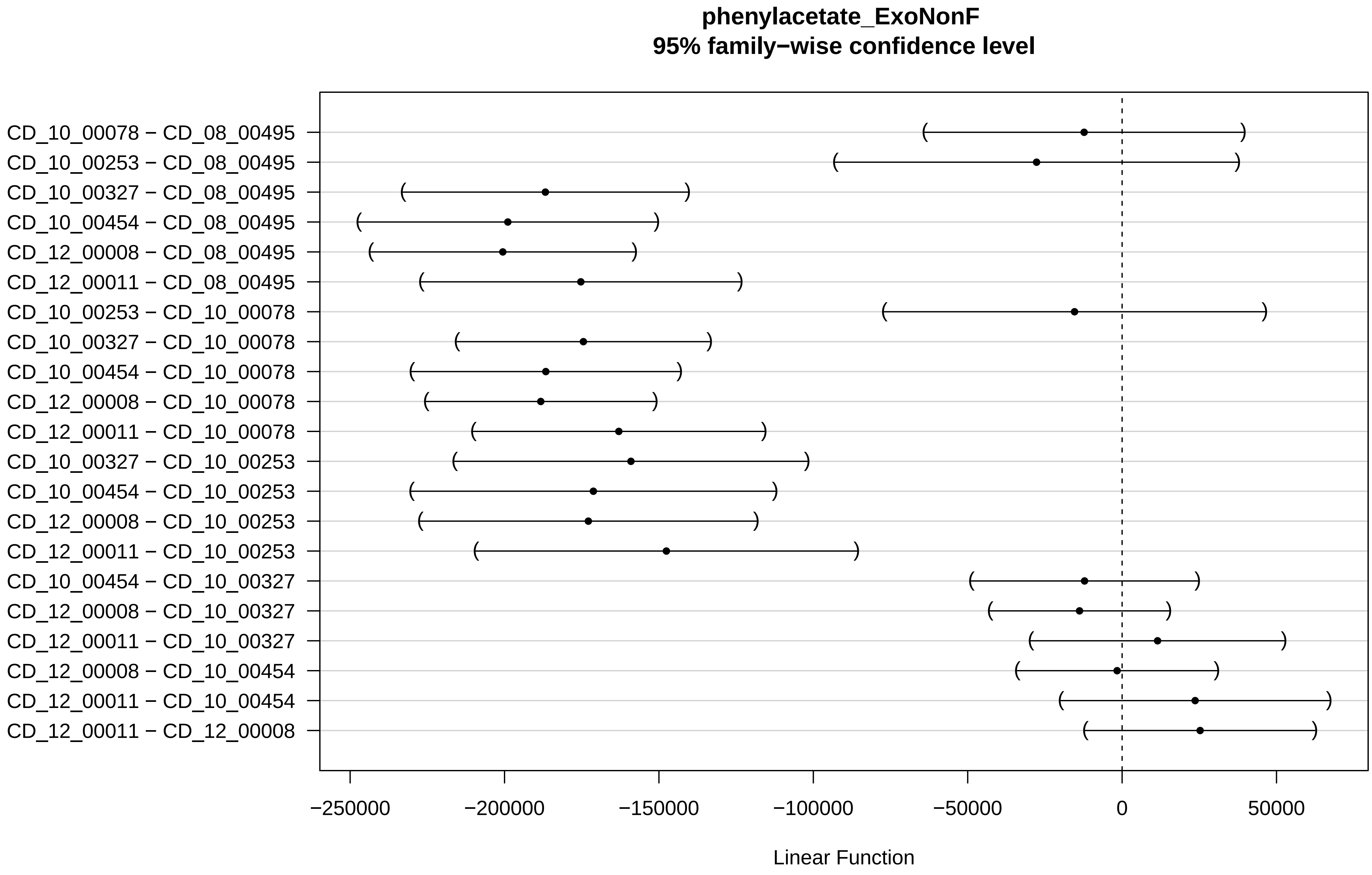
phenylacetate_ExoNonF
95% family−wise confidence level
CD_10_00078 − CD_08_00495
CD_10_00253 − CD_08_00495
CD_10_00327 − CD_08_00495
CD_10_00454 − CD_08_00495
CD_12_00008 − CD_08_00495
CD_12_00011 − CD_08_00495
CD_10_00253 − CD_10_00078
CD_10_00327 − CD_10_00078
CD_10_00454 − CD_10_00078
CD_12_00008 − CD_10_00078
CD_12_00011 − CD_10_00078
CD_10_00327 − CD_10_00253
CD_10_00454 − CD_10_00253
CD_12_00008 − CD_10_00253
CD_12_00011 − CD_10_00253
CD_10_00454 − CD_10_00327
CD_12_00008 − CD_10_00327
CD_12_00011 − CD_10_00327
CD_12_00008 − CD_10_00454
CD_12_00011 − CD_10_00454
CD_12_00011 − CD_12_00008
−250000
−200000
−150000
−100000
−50000
0
50000
Linear Function

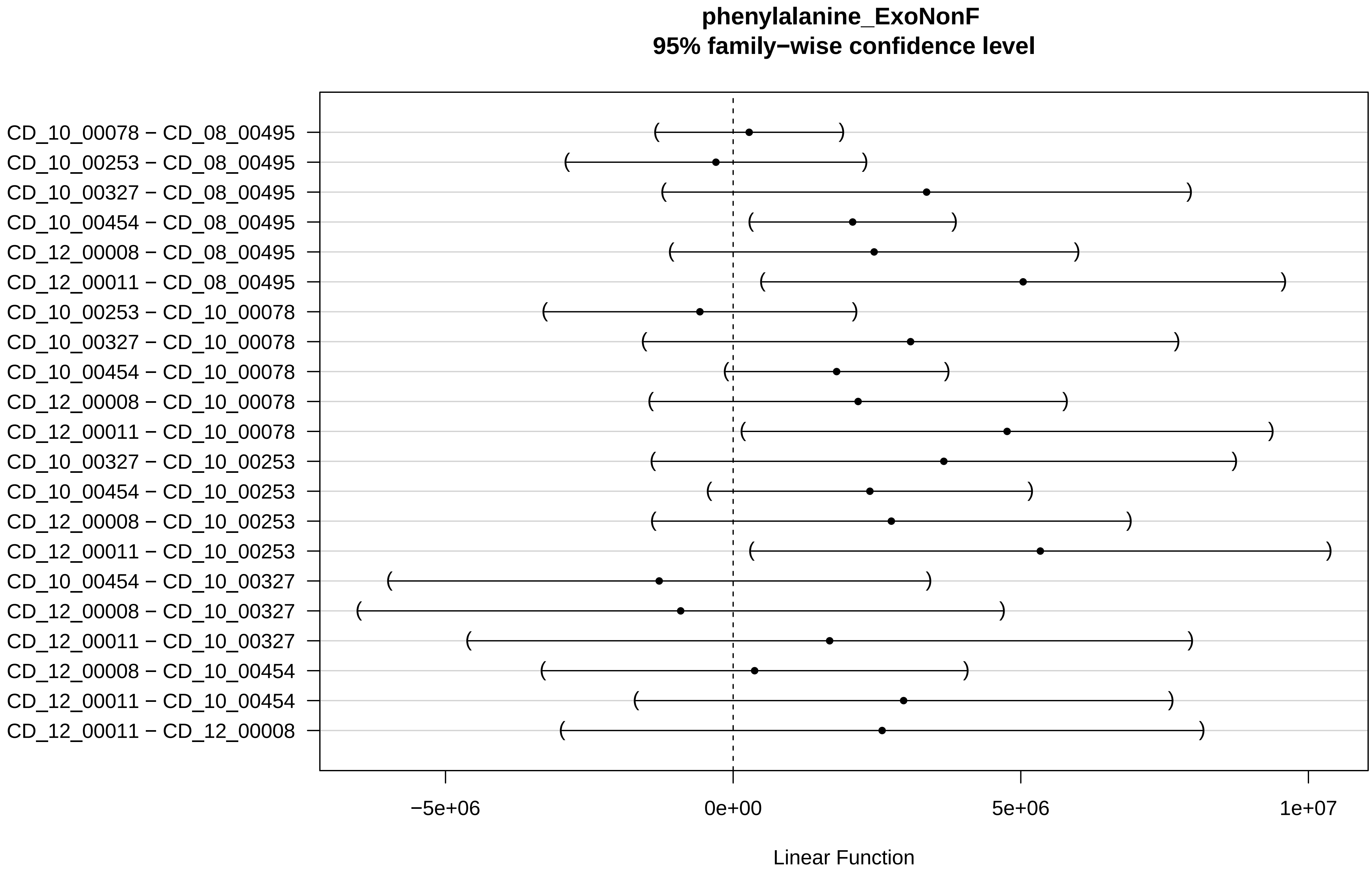
phenylalanine_ExoNonF
95% family−wise confidence level
CD_10_00078 − CD_08_00495
CD_10_00253 − CD_08_00495
CD_10_00327 − CD_08_00495
CD_10_00454 − CD_08_00495
CD_12_00008 − CD_08_00495
CD_12_00011 − CD_08_00495
CD_10_00253 − CD_10_00078
CD_10_00327 − CD_10_00078
CD_10_00454 − CD_10_00078
CD_12_00008 − CD_10_00078
CD_12_00011 − CD_10_00078
CD_10_00327 − CD_10_00253
CD_10_00454 − CD_10_00253
CD_12_00008 − CD_10_00253
CD_12_00011 − CD_10_00253
CD_10_00454 − CD_10_00327
CD_12_00008 − CD_10_00327
CD_12_00011 − CD_10_00327
CD_12_00008 − CD_10_00454
CD_12_00011 − CD_10_00454
CD_12_00011 − CD_12_00008
−5e+06
0e+00
5e+06
1e+07
Linear Function

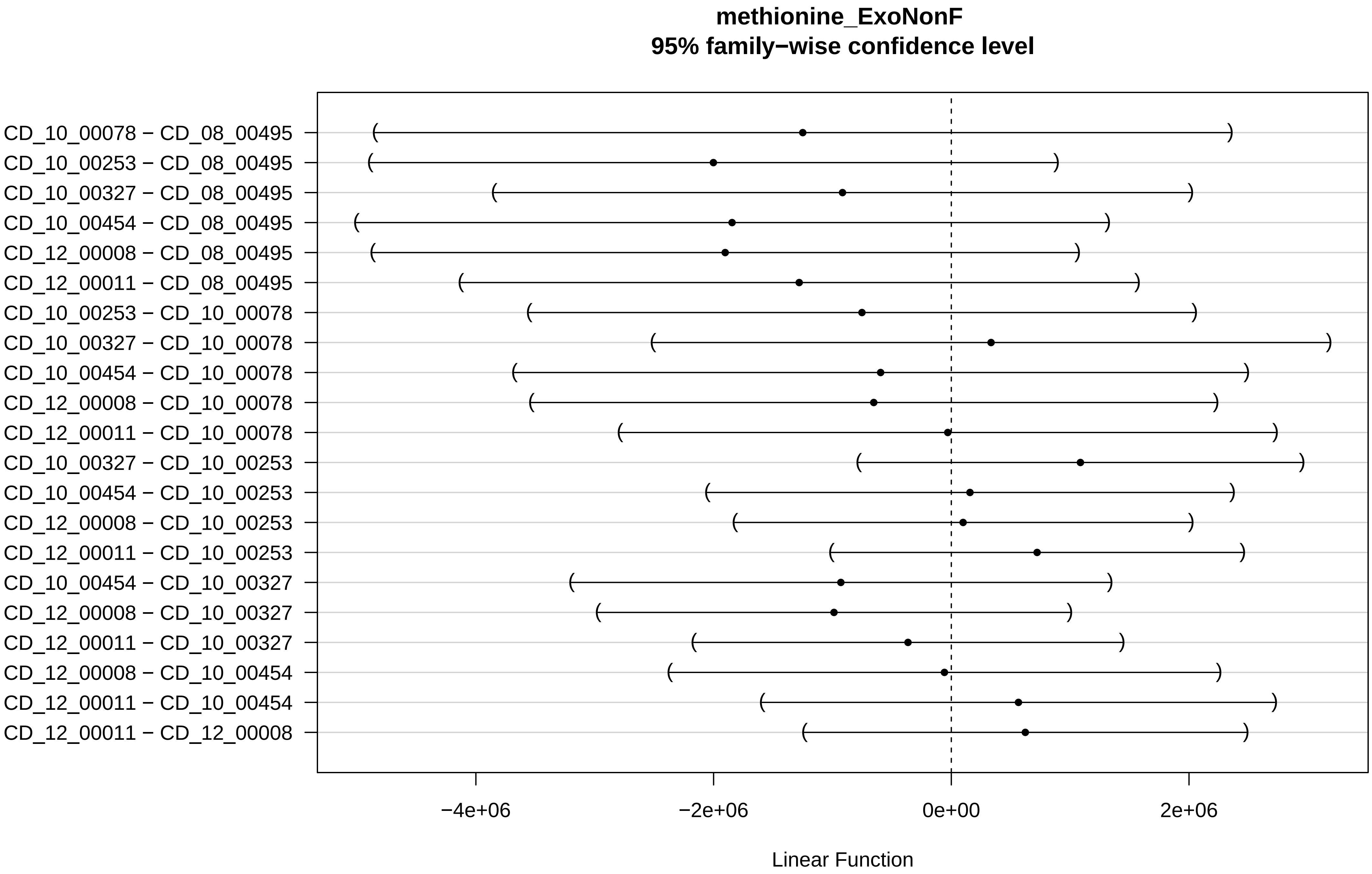
methionine_ExoNonF
95% family−wise confidence level
CD_10_00078 − CD_08_00495
CD_10_00253 − CD_08_00495
CD_10_00327 − CD_08_00495
CD_10_00454 − CD_08_00495
CD_12_00008 − CD_08_00495
CD_12_00011 − CD_08_00495
CD_10_00253 − CD_10_00078
CD_10_00327 − CD_10_00078
CD_10_00454 − CD_10_00078
CD_12_00008 − CD_10_00078
CD_12_00011 − CD_10_00078
CD_10_00327 − CD_10_00253
CD_10_00454 − CD_10_00253
CD_12_00008 − CD_10_00253
CD_12_00011 − CD_10_00253
CD_10_00454 − CD_10_00327
CD_12_00008 − CD_10_00327
CD_12_00011 − CD_10_00327
CD_12_00008 − CD_10_00454
CD_12_00011 − CD_10_00454
CD_12_00011 − CD_12_00008
−4e+06
−2e+06
0e+00
2e+06
Linear Function

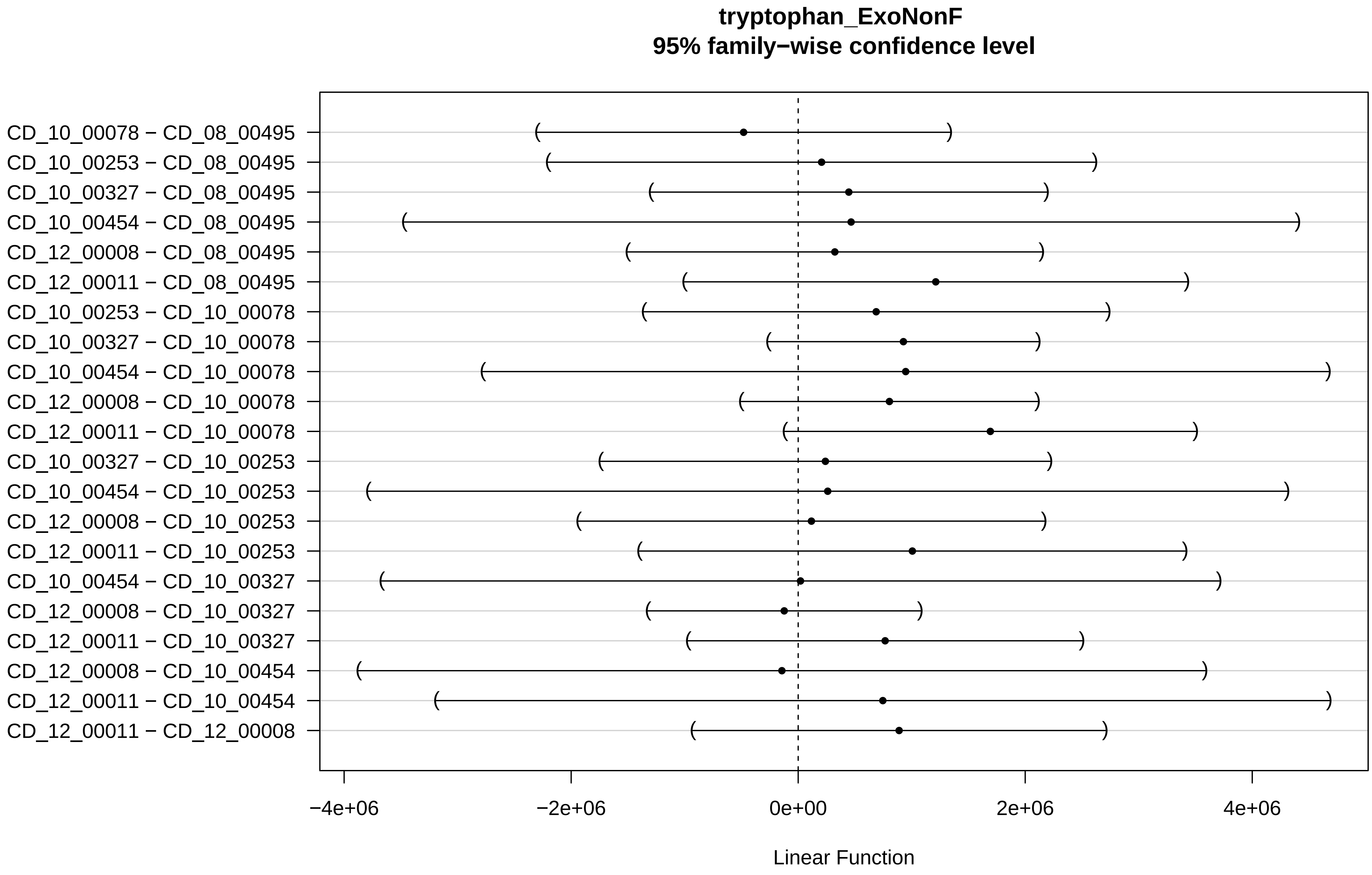
tryptophan_ExoNonF
95% family−wise confidence level
CD_10_00078 − CD_08_00495
CD_10_00253 − CD_08_00495
CD_10_00327 − CD_08_00495
CD_10_00454 − CD_08_00495
CD_12_00008 − CD_08_00495
CD_12_00011 − CD_08_00495
CD_10_00253 − CD_10_00078
CD_10_00327 − CD_10_00078
CD_10_00454 − CD_10_00078
CD_12_00008 − CD_10_00078
CD_12_00011 − CD_10_00078
CD_10_00327 − CD_10_00253
CD_10_00454 − CD_10_00253
CD_12_00008 − CD_10_00253
CD_12_00011 − CD_10_00253
CD_10_00454 − CD_10_00327
CD_12_00008 − CD_10_00327
CD_12_00011 − CD_10_00327
CD_12_00008 − CD_10_00454
CD_12_00011 − CD_10_00454
CD_12_00011 − CD_12_00008
−4e+06
−2e+06
0e+00
2e+06
4e+06
Linear Function

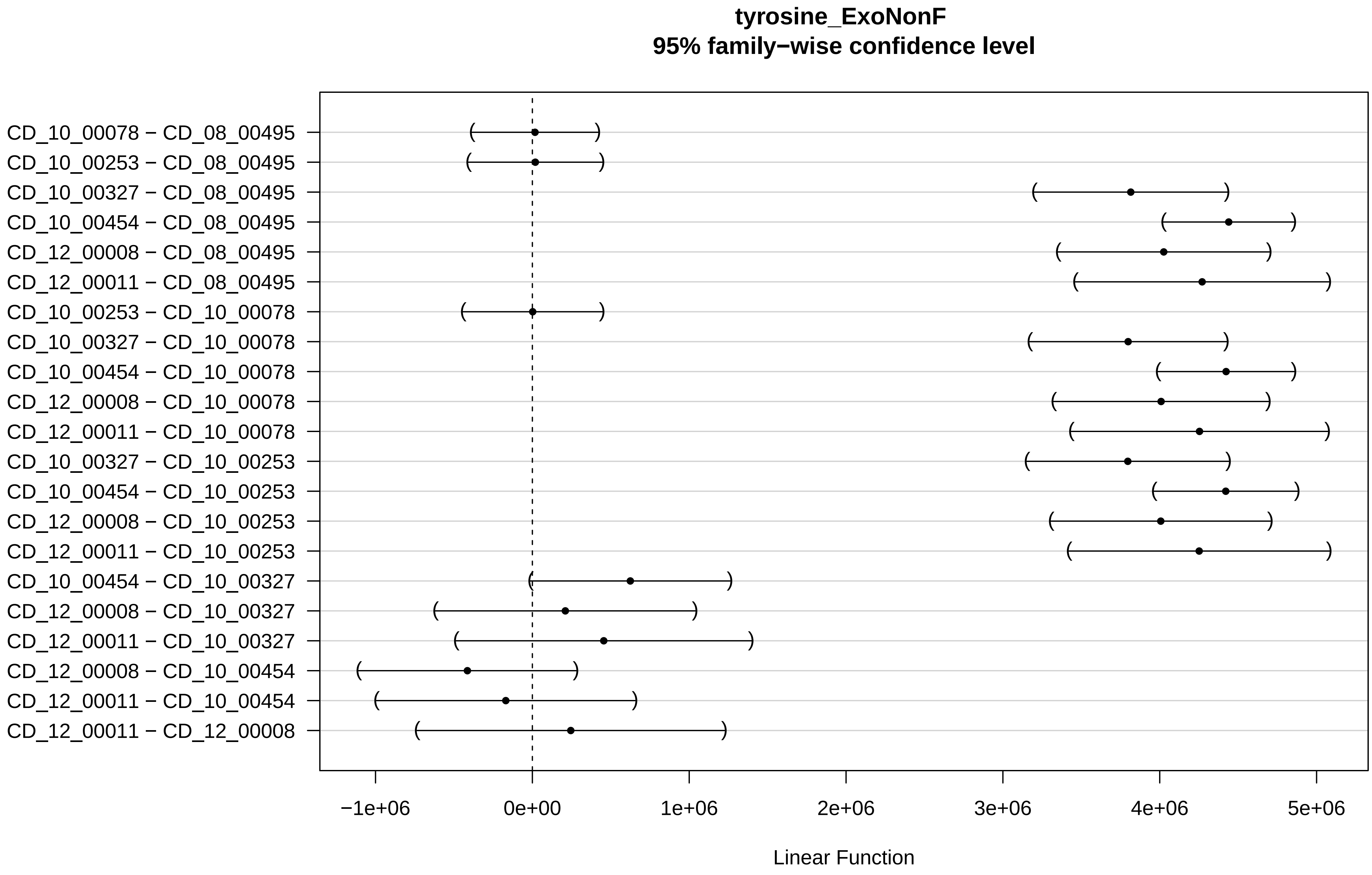
tyrosine_ExoNonF
95% family−wise confidence level
CD_10_00078 − CD_08_00495
CD_10_00253 − CD_08_00495
CD_10_00327 − CD_08_00495
CD_10_00454 − CD_08_00495
CD_12_00008 − CD_08_00495
CD_12_00011 − CD_08_00495
CD_10_00253 − CD_10_00078
CD_10_00327 − CD_10_00078
CD_10_00454 − CD_10_00078
CD_12_00008 − CD_10_00078
CD_12_00011 − CD_10_00078
CD_10_00327 − CD_10_00253
CD_10_00454 − CD_10_00253
CD_12_00008 − CD_10_00253
CD_12_00011 − CD_10_00253
CD_10_00454 − CD_10_00327
CD_12_00008 − CD_10_00327
CD_12_00011 − CD_10_00327
CD_12_00008 − CD_10_00454
CD_12_00011 − CD_10_00454
CD_12_00011 − CD_12_00008
−1e+06
0e+00
1e+06
2e+06
3e+06
4e+06
5e+06
Linear Function

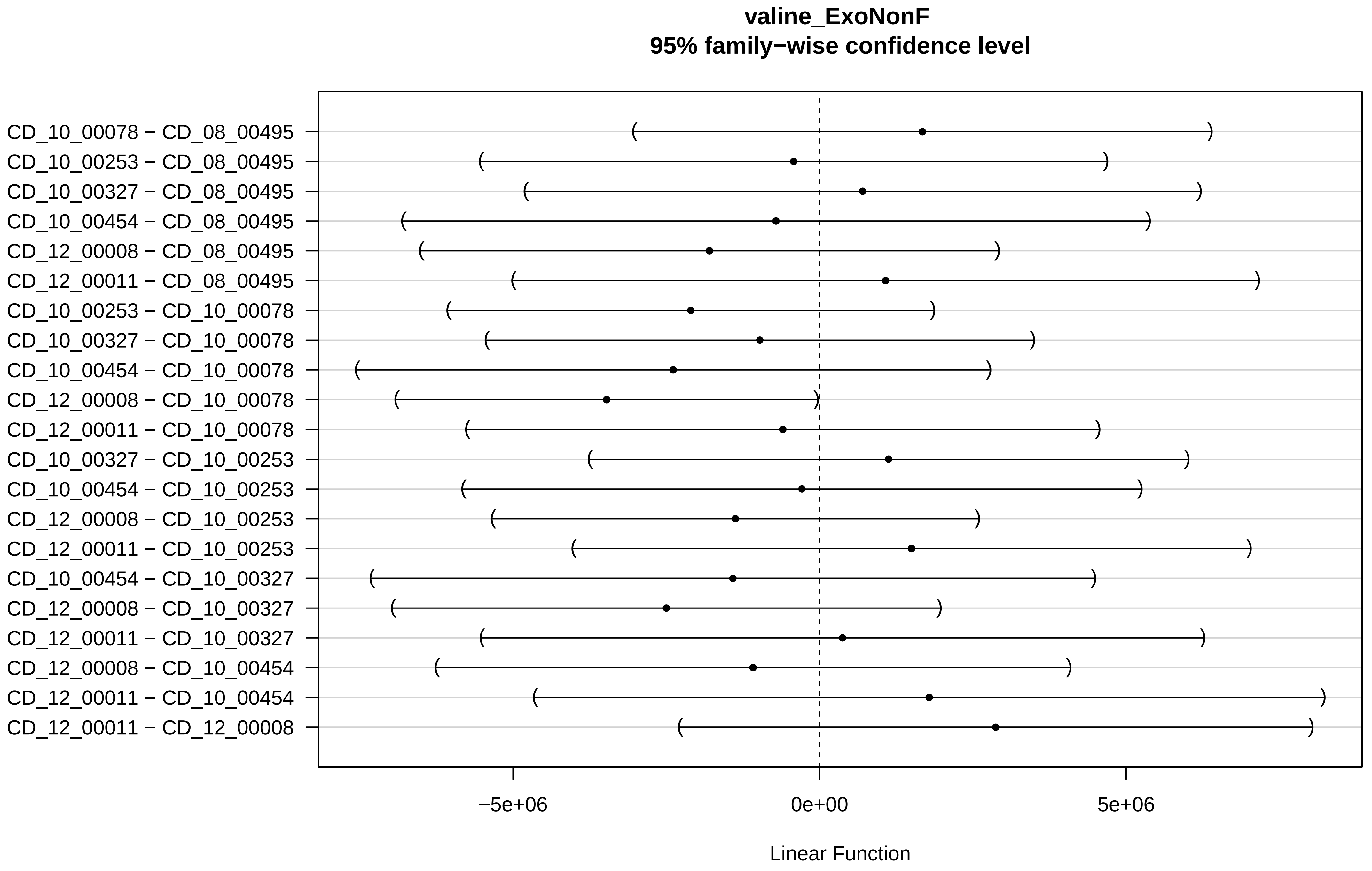
valine_ExoNonF
95% family−wise confidence level
CD_10_00078 − CD_08_00495
CD_10_00253 − CD_08_00495
CD_10_00327 − CD_08_00495
CD_10_00454 − CD_08_00495
CD_12_00008 − CD_08_00495
CD_12_00011 − CD_08_00495
CD_10_00253 − CD_10_00078
CD_10_00327 − CD_10_00078
CD_10_00454 − CD_10_00078
CD_12_00008 − CD_10_00078
CD_12_00011 − CD_10_00078
CD_10_00327 − CD_10_00253
CD_10_00454 − CD_10_00253
CD_12_00008 − CD_10_00253
CD_12_00011 − CD_10_00253
CD_10_00454 − CD_10_00327
CD_12_00008 − CD_10_00327
CD_12_00011 − CD_10_00327
CD_12_00008 − CD_10_00454
CD_12_00011 − CD_10_00454
CD_12_00011 − CD_12_00008
−5e+06
0e+00
5e+06
Linear Function

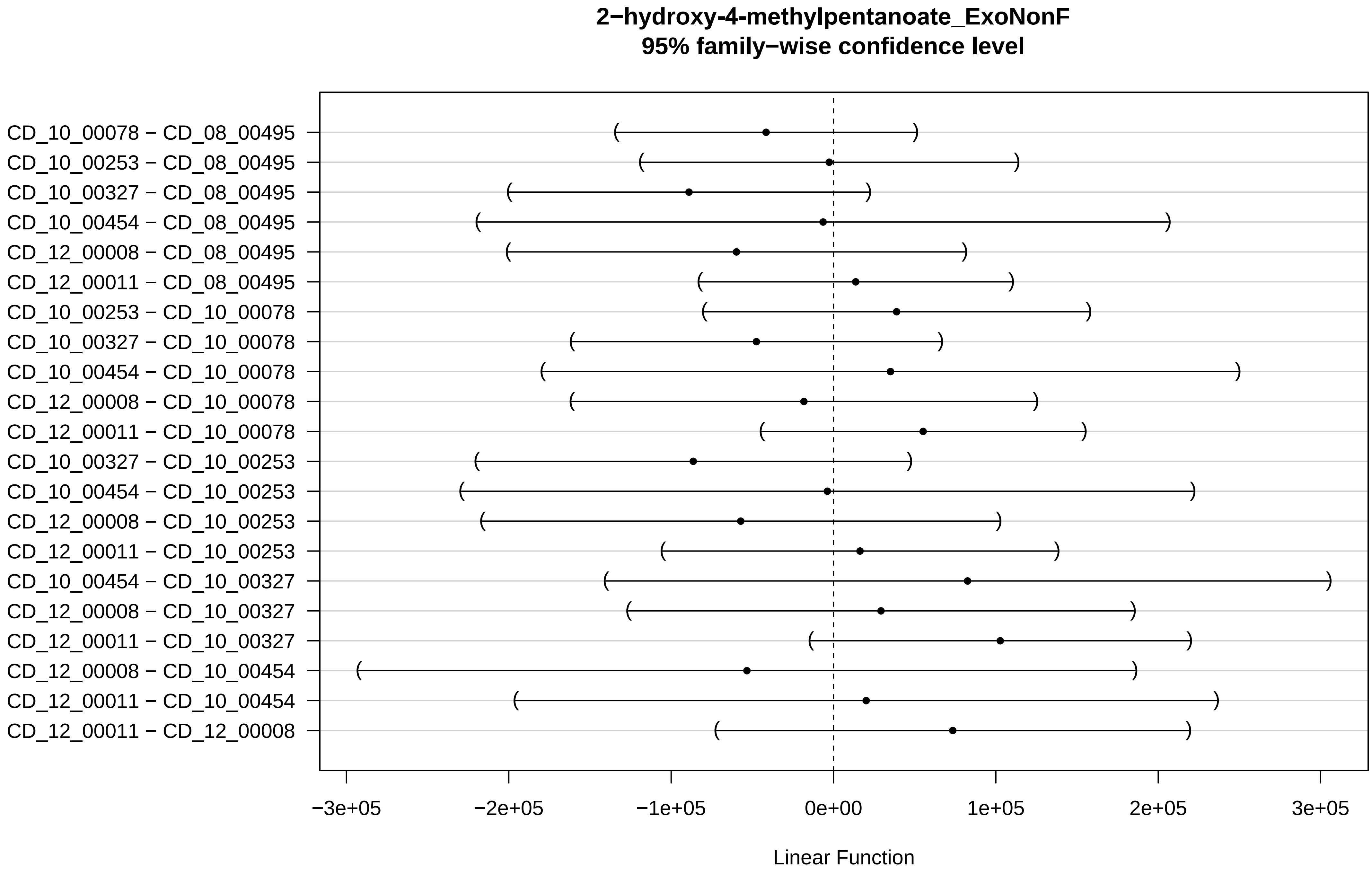

2−hydroxy-4-methylpentanoate_ExoNonF
95% family−wise confidence level
CD_10_00078 − CD_08_00495
CD_10_00253 − CD_08_00495
CD_10_00327 − CD_08_00495
CD_10_00454 − CD_08_00495
CD_12_00008 − CD_08_00495
CD_12_00011 − CD_08_00495
CD_10_00253 − CD_10_00078
CD_10_00327 − CD_10_00078
CD_10_00454 − CD_10_00078
CD_12_00008 − CD_10_00078
CD_12_00011 − CD_10_00078
CD_10_00327 − CD_10_00253
CD_10_00454 − CD_10_00253
CD_12_00008 − CD_10_00253
CD_12_00011 − CD_10_00253
CD_10_00454 − CD_10_00327
CD_12_00008 − CD_10_00327
CD_12_00011 − CD_10_00327
CD_12_00008 − CD_10_00454
CD_12_00011 − CD_10_00454
CD_12_00011 − CD_12_00008
−3e+05
−2e+05
−1e+05
0e+00
1e+05
2e+05
3e+05
Linear Function

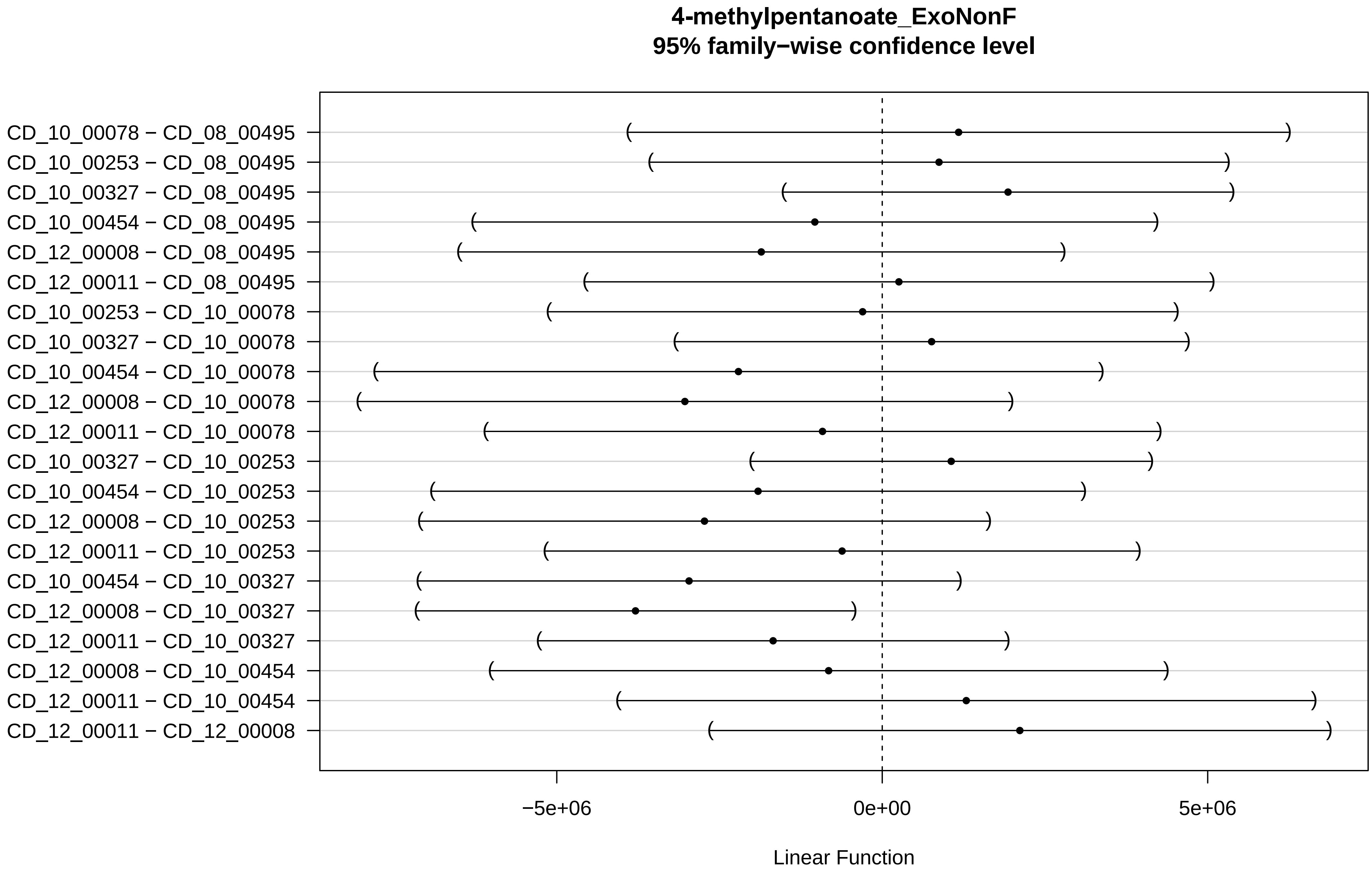
4-methylpentanoate_ExoNonF
95% family−wise confidence level
CD_10_00078 − CD_08_00495
CD_10_00253 − CD_08_00495
CD_10_00327 − CD_08_00495
CD_10_00454 − CD_08_00495
CD_12_00008 − CD_08_00495
CD_12_00011 − CD_08_00495
CD_10_00253 − CD_10_00078
CD_10_00327 − CD_10_00078
CD_10_00454 − CD_10_00078
CD_12_00008 − CD_10_00078
CD_12_00011 − CD_10_00078
CD_10_00327 − CD_10_00253
CD_10_00454 − CD_10_00253
CD_12_00008 − CD_10_00253
CD_12_00011 − CD_10_00253
CD_10_00454 − CD_10_00327
CD_12_00008 − CD_10_00327
CD_12_00011 − CD_10_00327
CD_12_00008 − CD_10_00454
CD_12_00011 − CD_10_00454
CD_12_00011 − CD_12_00008
−5e+06
0e+00
5e+06
Linear Function

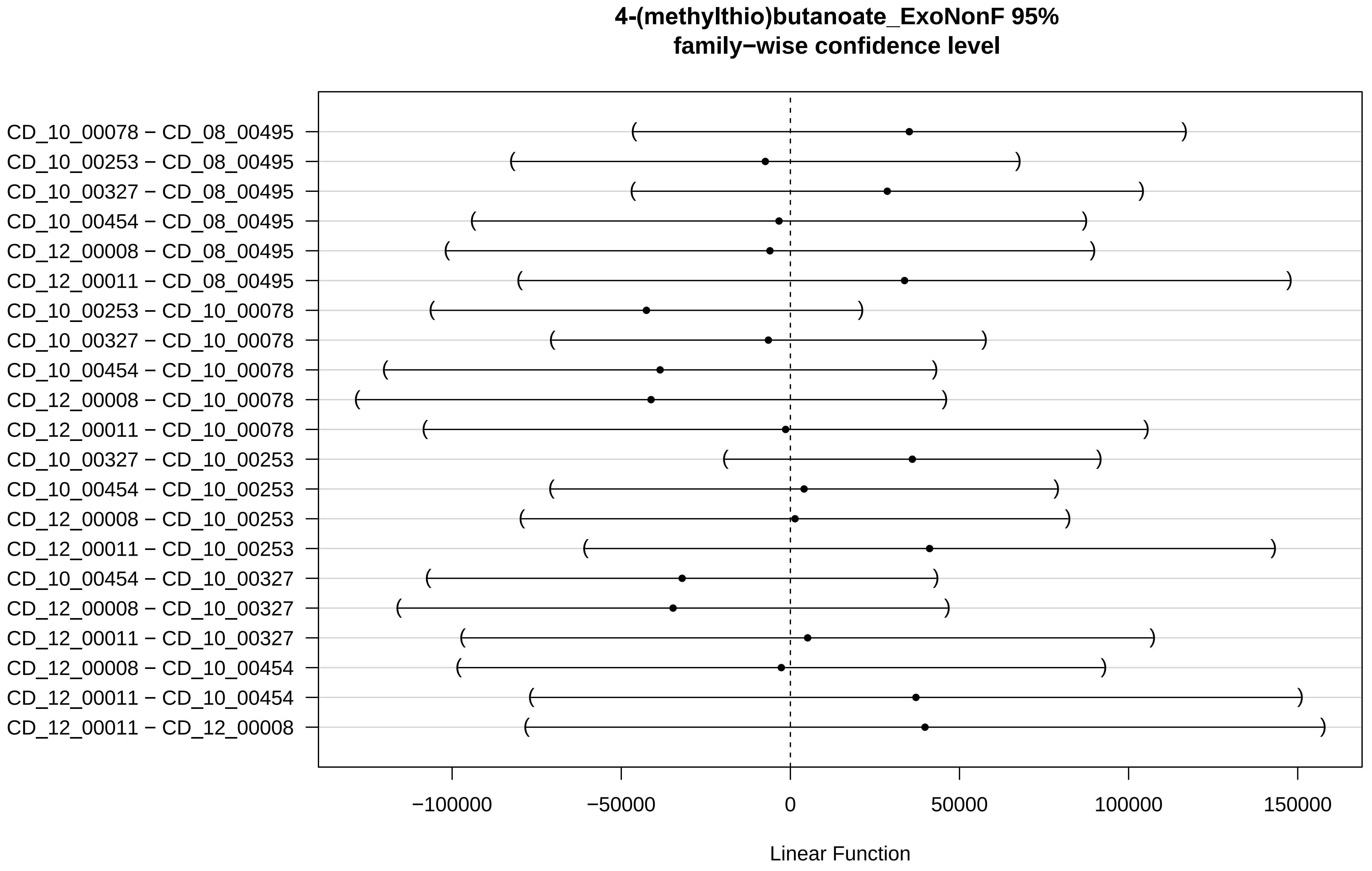
4-(methylthio)butanoate_ExoNonF 95%
family−wise confidence level
CD_10_00078 − CD_08_00495
CD_10_00253 − CD_08_00495
CD_10_00327 − CD_08_00495
CD_10_00454 − CD_08_00495
CD_12_00008 − CD_08_00495
CD_12_00011 − CD_08_00495
CD_10_00253 − CD_10_00078
CD_10_00327 − CD_10_00078
CD_10_00454 − CD_10_00078
CD_12_00008 − CD_10_00078
CD_12_00011 − CD_10_00078
CD_10_00327 − CD_10_00253
CD_10_00454 − CD_10_00253
CD_12_00008 − CD_10_00253
CD_12_00011 − CD_10_00253
CD_10_00454 − CD_10_00327
CD_12_00008 − CD_10_00327
CD_12_00011 − CD_10_00327
CD_12_00008 − CD_10_00454
CD_12_00011 − CD_10_00454
CD_12_00011 − CD_12_00008
−100000
−50000
0
50000
100000
150000
Linear Function

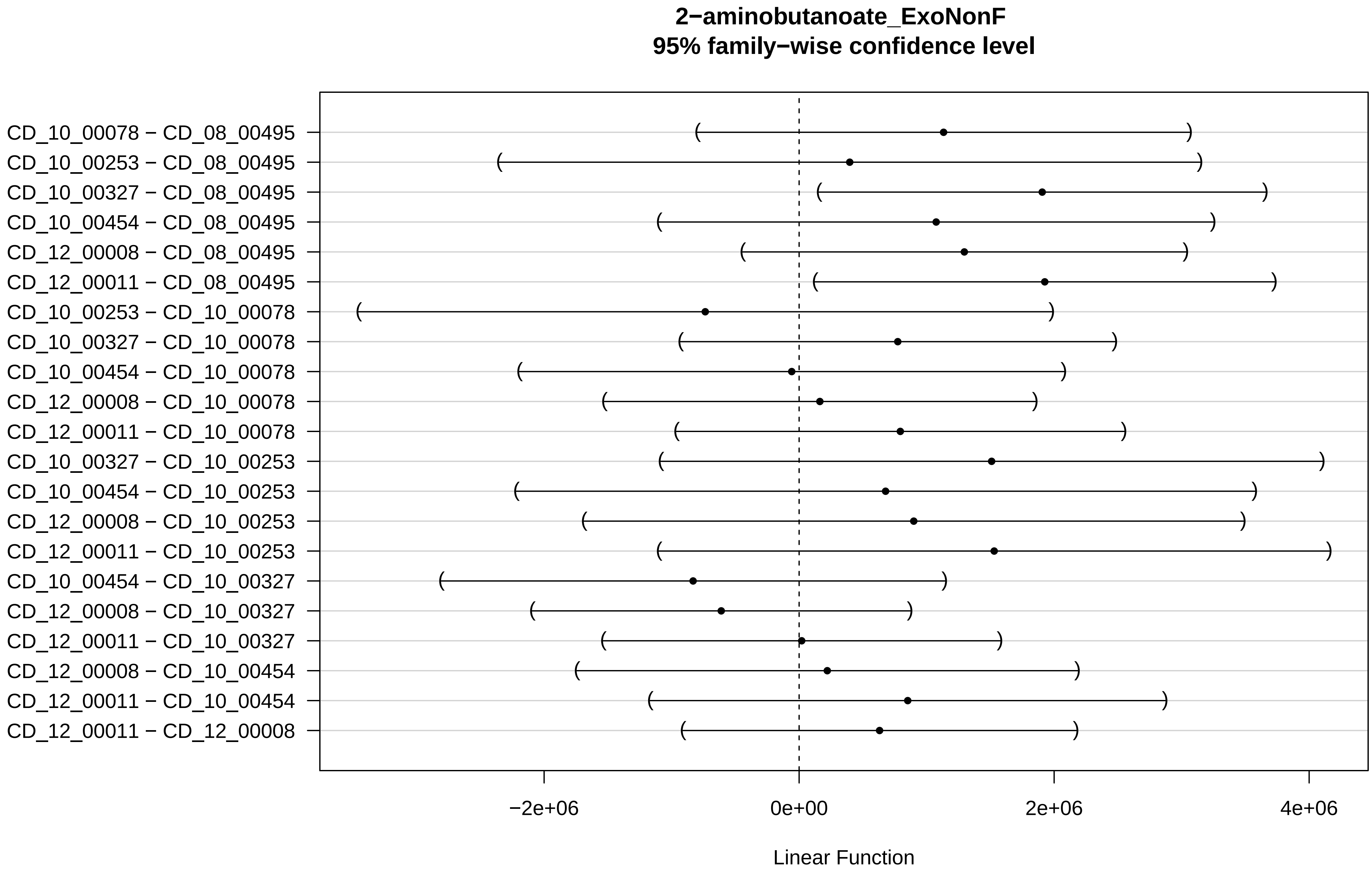
2−aminobutanoate_ExoNonF
95% family−wise confidence level
CD_10_00078 − CD_08_00495
CD_10_00253 − CD_08_00495
CD_10_00327 − CD_08_00495
CD_10_00454 − CD_08_00495
CD_12_00008 − CD_08_00495
CD_12_00011 − CD_08_00495
CD_10_00253 − CD_10_00078
CD_10_00327 − CD_10_00078
CD_10_00454 − CD_10_00078
CD_12_00008 − CD_10_00078
CD_12_00011 − CD_10_00078
CD_10_00327 − CD_10_00253
CD_10_00454 − CD_10_00253
CD_12_00008 − CD_10_00253
CD_12_00011 − CD_10_00253
CD_10_00454 − CD_10_00327
CD_12_00008 − CD_10_00327
CD_12_00011 − CD_10_00327
CD_12_00008 − CD_10_00454
CD_12_00011 − CD_10_00454
CD_12_00011 − CD_12_00008
−2e+06
0e+00
2e+06
4e+06
Linear Function

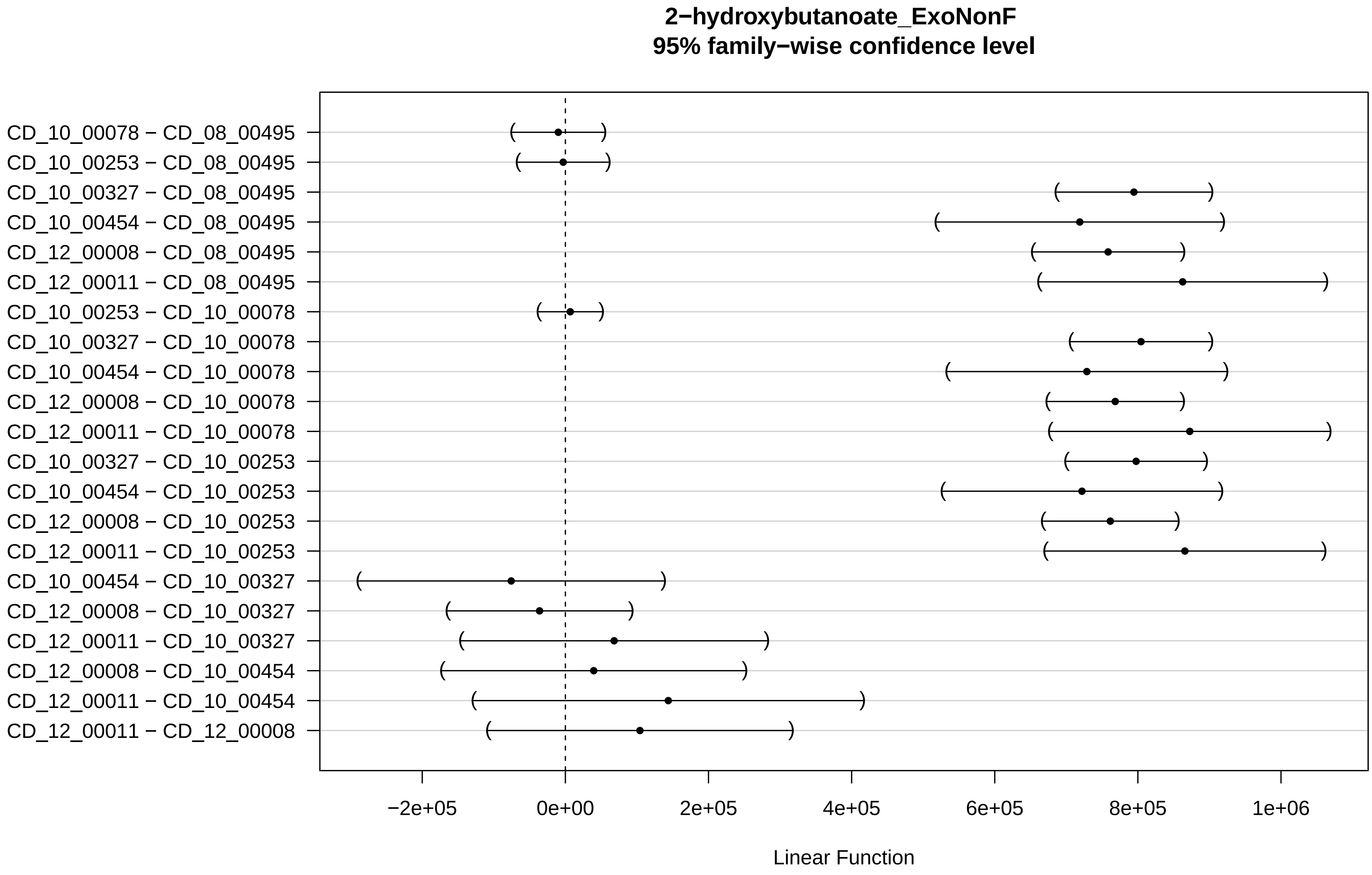

2−hydroxybutanoate_ExoNonF
95% family−wise confidence level
CD_10_00078 − CD_08_00495
CD_10_00253 − CD_08_00495
CD_10_00327 − CD_08_00495
CD_10_00454 − CD_08_00495
CD_12_00008 − CD_08_00495
CD_12_00011 − CD_08_00495
CD_10_00253 − CD_10_00078
CD_10_00327 − CD_10_00078
CD_10_00454 − CD_10_00078
CD_12_00008 − CD_10_00078
CD_12_00011 − CD_10_00078
CD_10_00327 − CD_10_00253
CD_10_00454 − CD_10_00253
CD_12_00008 − CD_10_00253
CD_12_00011 − CD_10_00253
CD_10_00454 − CD_10_00327
CD_12_00008 − CD_10_00327
CD_12_00011 − CD_10_00327
CD_12_00008 − CD_10_00454
CD_12_00011 − CD_10_00454
CD_12_00011 − CD_12_00008
−2e+05
0e+00
2e+05
4e+05
6e+05
8e+05
1e+06
Linear Function

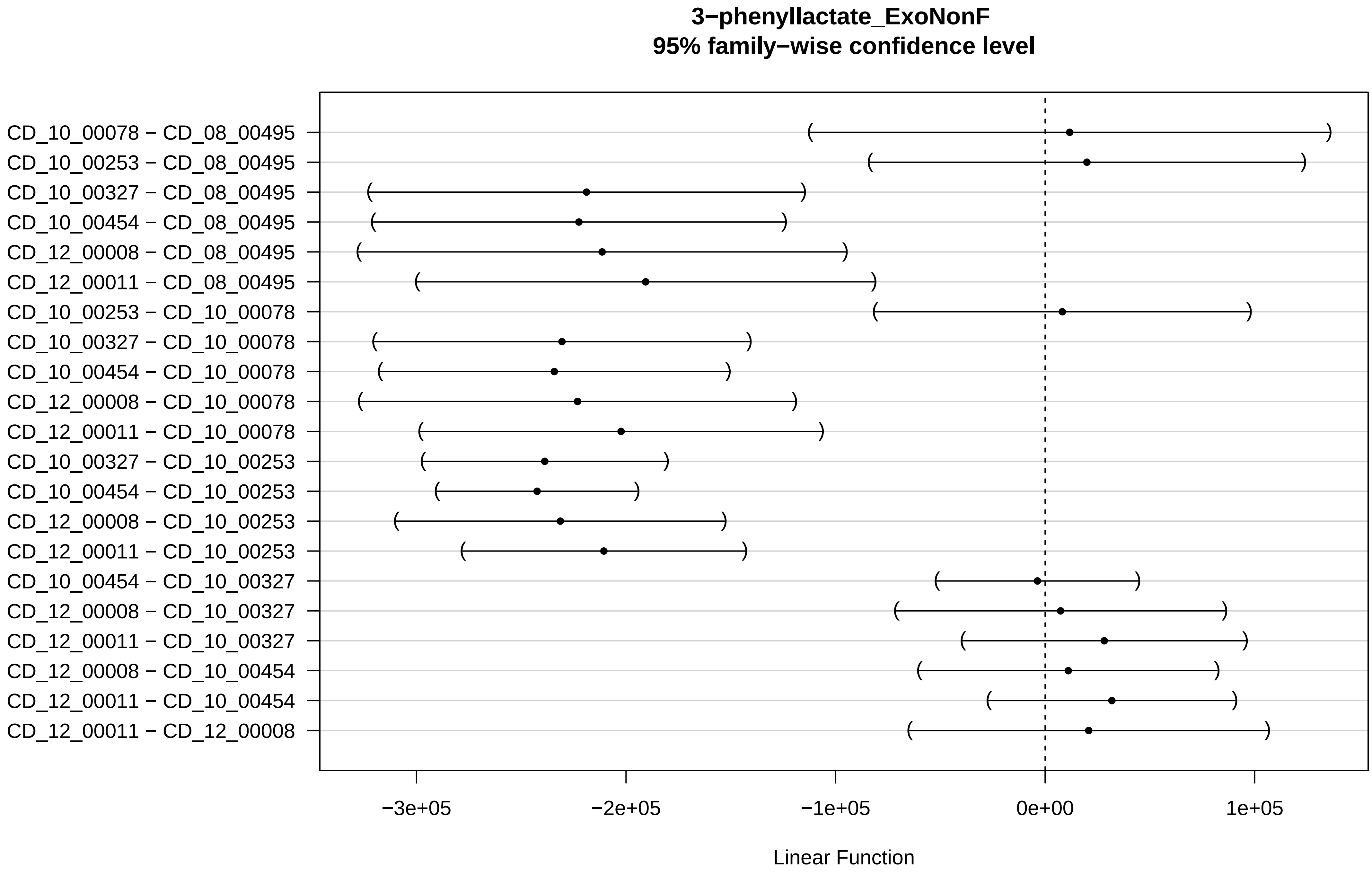
3−phenyllactate_ExoNonF
95% family−wise confidence level
CD_10_00078 − CD_08_00495
CD_10_00253 − CD_08_00495
CD_10_00327 − CD_08_00495
CD_10_00454 − CD_08_00495
CD_12_00008 − CD_08_00495
CD_12_00011 − CD_08_00495
CD_10_00253 − CD_10_00078
CD_10_00327 − CD_10_00078
CD_10_00454 − CD_10_00078
CD_12_00008 − CD_10_00078
CD_12_00011 − CD_10_00078
CD_10_00327 − CD_10_00253
CD_10_00454 − CD_10_00253
CD_12_00008 − CD_10_00253
CD_12_00011 − CD_10_00253
CD_10_00454 − CD_10_00327
CD_12_00008 − CD_10_00327
CD_12_00011 − CD_10_00327
CD_12_00008 − CD_10_00454
CD_12_00011 − CD_10_00454
CD_12_00011 − CD_12_00008
−3e+05
−2e+05
−1e+05
0e+00
1e+05
Linear Function

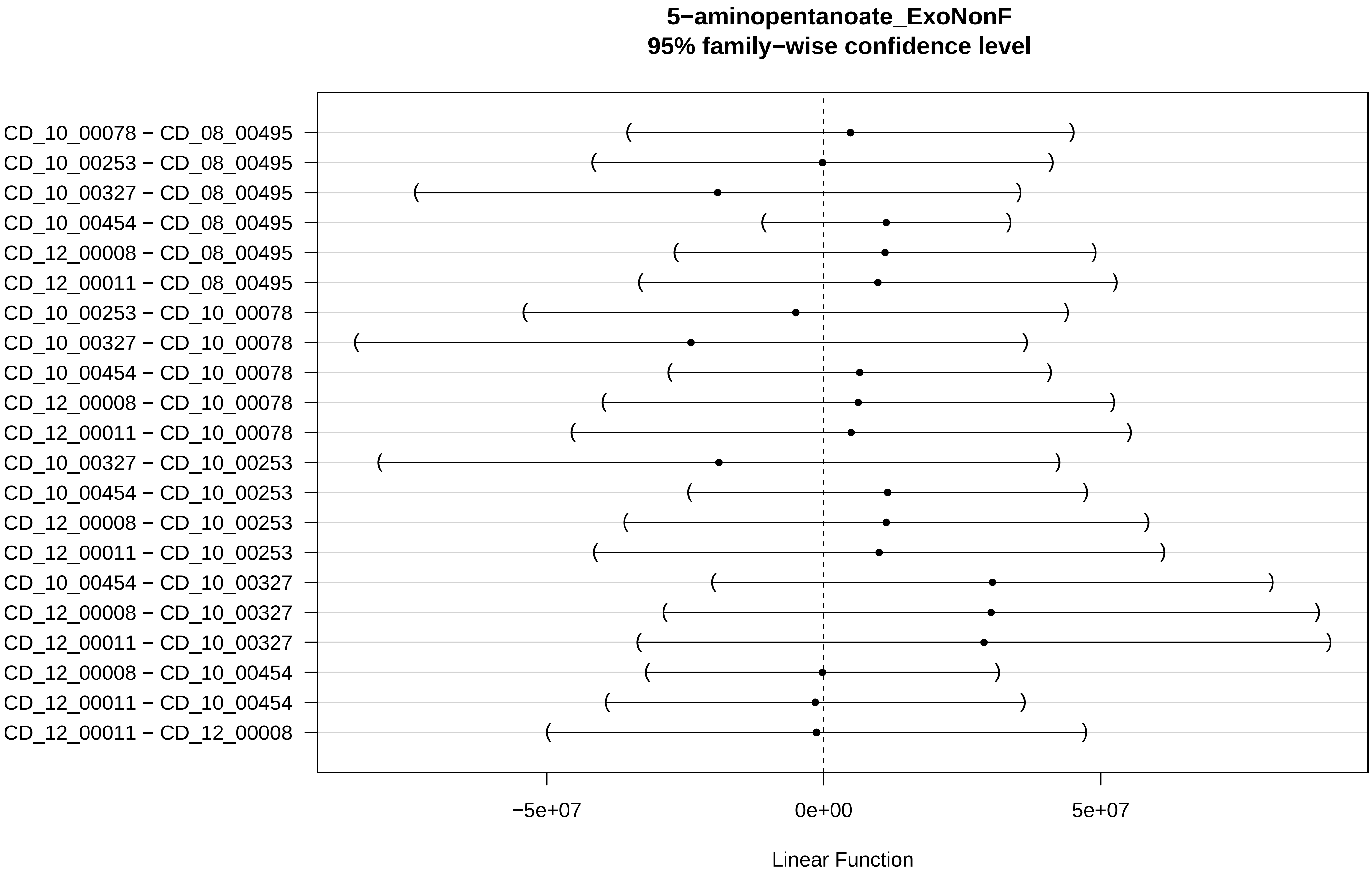

5−aminopentanoate_ExoNonF
95% family−wise confidence level
CD_10_00078 − CD_08_00495
CD_10_00253 − CD_08_00495
CD_10_00327 − CD_08_00495
CD_10_00454 − CD_08_00495
CD_12_00008 − CD_08_00495
CD_12_00011 − CD_08_00495
CD_10_00253 − CD_10_00078
CD_10_00327 − CD_10_00078
CD_10_00454 − CD_10_00078
CD_12_00008 − CD_10_00078
CD_12_00011 − CD_10_00078
CD_10_00327 − CD_10_00253
CD_10_00454 − CD_10_00253
CD_12_00008 − CD_10_00253
CD_12_00011 − CD_10_00253
CD_10_00454 − CD_10_00327
CD_12_00008 − CD_10_00327
CD_12_00011 − CD_10_00327
CD_12_00008 − CD_10_00454
CD_12_00011 − CD_10_00454
CD_12_00011 − CD_12_00008
−5e+07
0e+00
5e+07
Linear Function

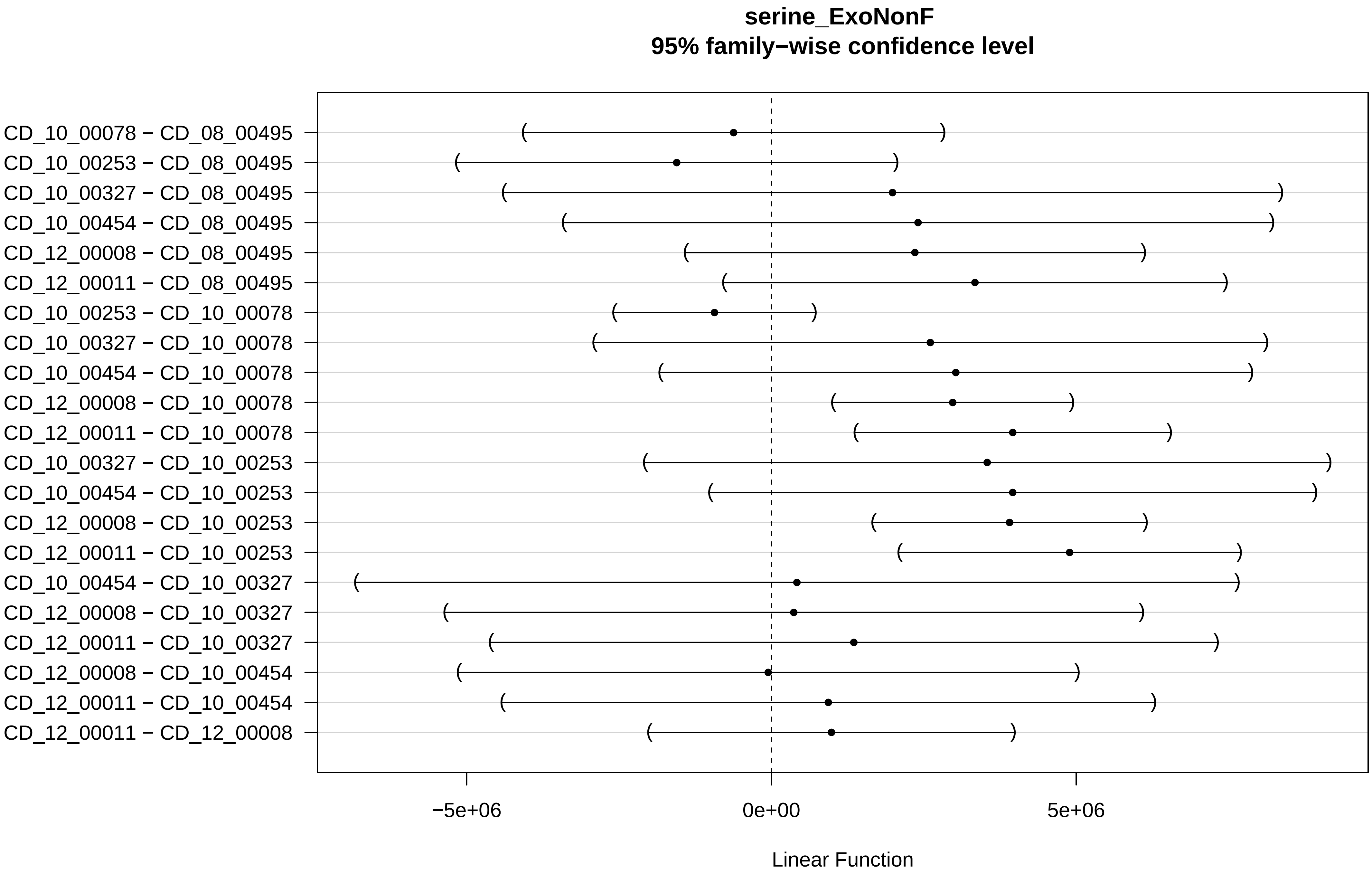
serine_ExoNonF
95% family−wise confidence level
CD_10_00078 − CD_08_00495
CD_10_00253 − CD_08_00495
CD_10_00327 − CD_08_00495
CD_10_00454 − CD_08_00495
CD_12_00008 − CD_08_00495
CD_12_00011 − CD_08_00495
CD_10_00253 − CD_10_00078
CD_10_00327 − CD_10_00078
CD_10_00454 − CD_10_00078
CD_12_00008 − CD_10_00078
CD_12_00011 − CD_10_00078
CD_10_00327 − CD_10_00253
CD_10_00454 − CD_10_00253
CD_12_00008 − CD_10_00253
CD_12_00011 − CD_10_00253
CD_10_00454 − CD_10_00327
CD_12_00008 − CD_10_00327
CD_12_00011 − CD_10_00327
CD_12_00008 − CD_10_00454
CD_12_00011 − CD_10_00454
CD_12_00011 − CD_12_00008
−5e+06
0e+00
5e+06
Linear Function

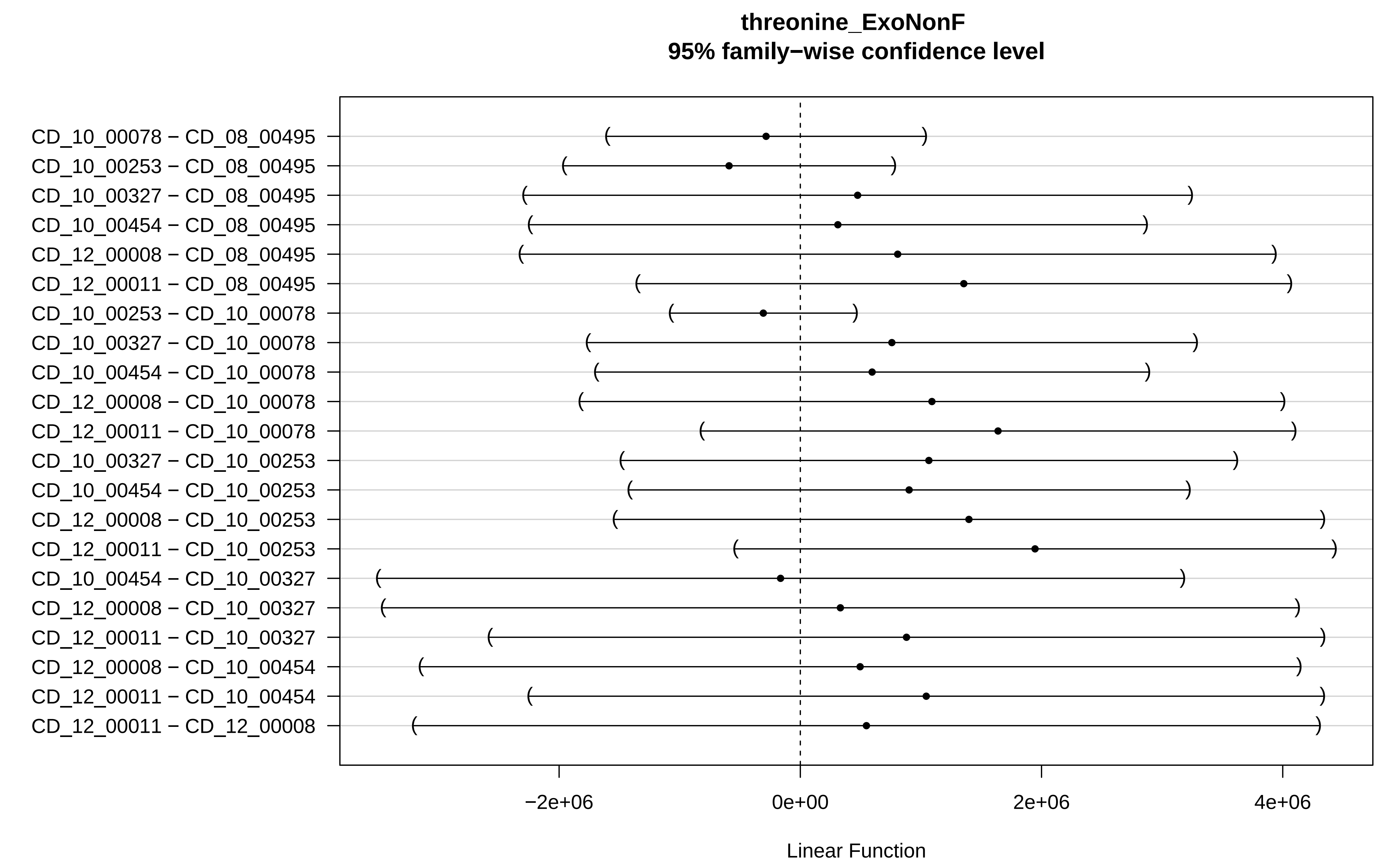
threonine_ExoNonF
95% family−wise confidence level
CD_10_00078 − CD_08_00495
CD_10_00253 − CD_08_00495
CD_10_00327 − CD_08_00495
CD_10_00454 − CD_08_00495
CD_12_00008 − CD_08_00495
CD_12_00011 − CD_08_00495
CD_10_00253 − CD_10_00078
CD_10_00327 − CD_10_00078
CD_10_00454 − CD_10_00078
CD_12_00008 − CD_10_00078
CD_12_00011 − CD_10_00078
CD_10_00327 − CD_10_00253
CD_10_00454 − CD_10_00253
CD_12_00008 − CD_10_00253
CD_12_00011 − CD_10_00253
CD_10_00454 − CD_10_00327
CD_12_00008 − CD_10_00327
CD_12_00011 − CD_10_00327
CD_12_00008 − CD_10_00454
CD_12_00011 − CD_10_00454
CD_12_00011 − CD_12_00008
−2e+06
0e+00
2e+06
4e+06
Linear Function

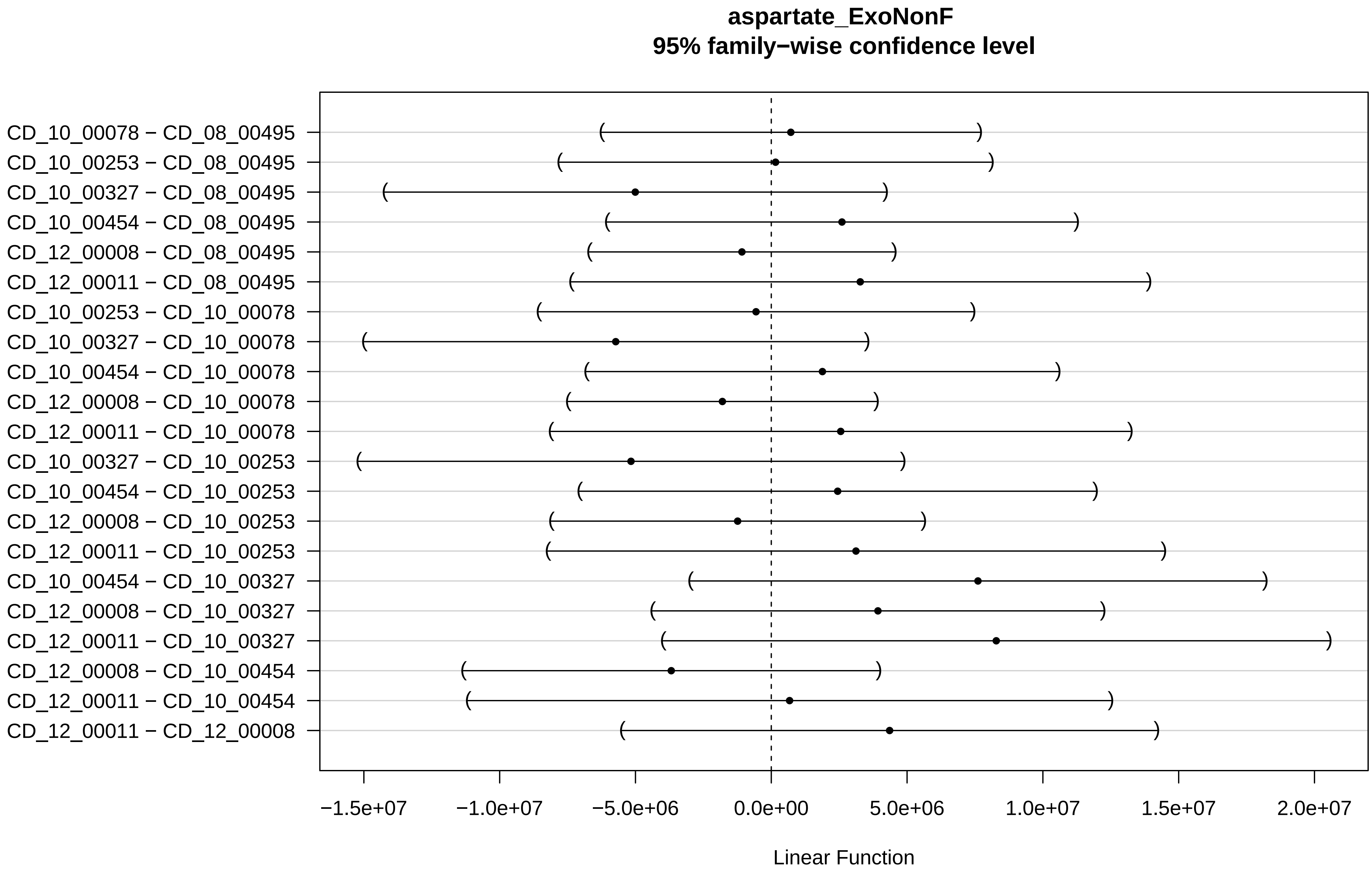
aspartate_ExoNonF
95% family−wise confidence level
CD_10_00078 − CD_08_00495
CD_10_00253 − CD_08_00495
CD_10_00327 − CD_08_00495
CD_10_00454 − CD_08_00495
CD_12_00008 − CD_08_00495
CD_12_00011 − CD_08_00495
CD_10_00253 − CD_10_00078
CD_10_00327 − CD_10_00078
CD_10_00454 − CD_10_00078
CD_12_00008 − CD_10_00078
CD_12_00011 − CD_10_00078
CD_10_00327 − CD_10_00253
CD_10_00454 − CD_10_00253
CD_12_00008 − CD_10_00253
CD_12_00011 − CD_10_00253
CD_10_00454 − CD_10_00327
CD_12_00008 − CD_10_00327
CD_12_00011 − CD_10_00327
CD_12_00008 − CD_10_00454
CD_12_00011 − CD_10_00454
CD_12_00011 − CD_12_00008
−1.5e+07
−1.0e+07
−5.0e+06
0.0e+00
5.0e+06
1.0e+07
1.5e+07
2.0e+07
Linear Function

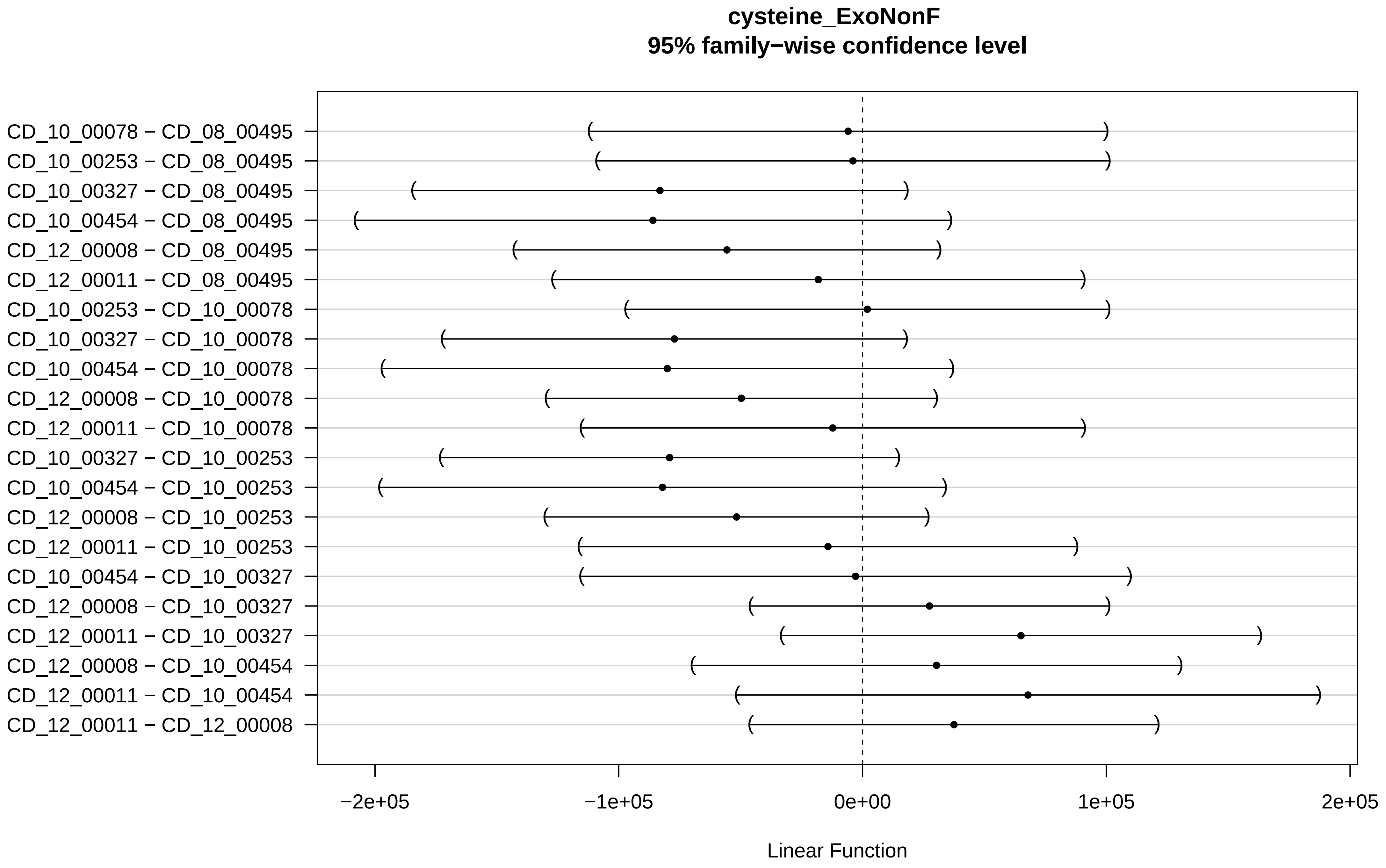

cysteine_ExoNonF
95% family−wise confidence level
CD_10_00078 − CD_08_00495
CD_10_00253 − CD_08_00495
CD_10_00327 − CD_08_00495
CD_10_00454 − CD_08_00495
CD_12_00008 − CD_08_00495
CD_12_00011 − CD_08_00495
CD_10_00253 − CD_10_00078
CD_10_00327 − CD_10_00078
CD_10_00454 − CD_10_00078
CD_12_00008 − CD_10_00078
CD_12_00011 − CD_10_00078
CD_10_00327 − CD_10_00253
CD_10_00454 − CD_10_00253
CD_12_00008 − CD_10_00253
CD_12_00011 − CD_10_00253
CD_10_00454 − CD_10_00327
CD_12_00008 − CD_10_00327
CD_12_00011 − CD_10_00327
CD_12_00008 − CD_10_00454
CD_12_00011 − CD_10_00454
CD_12_00011 − CD_12_00008
−2e+05
−1e+05
0e+00
1e+05
2e+05
Linear Function

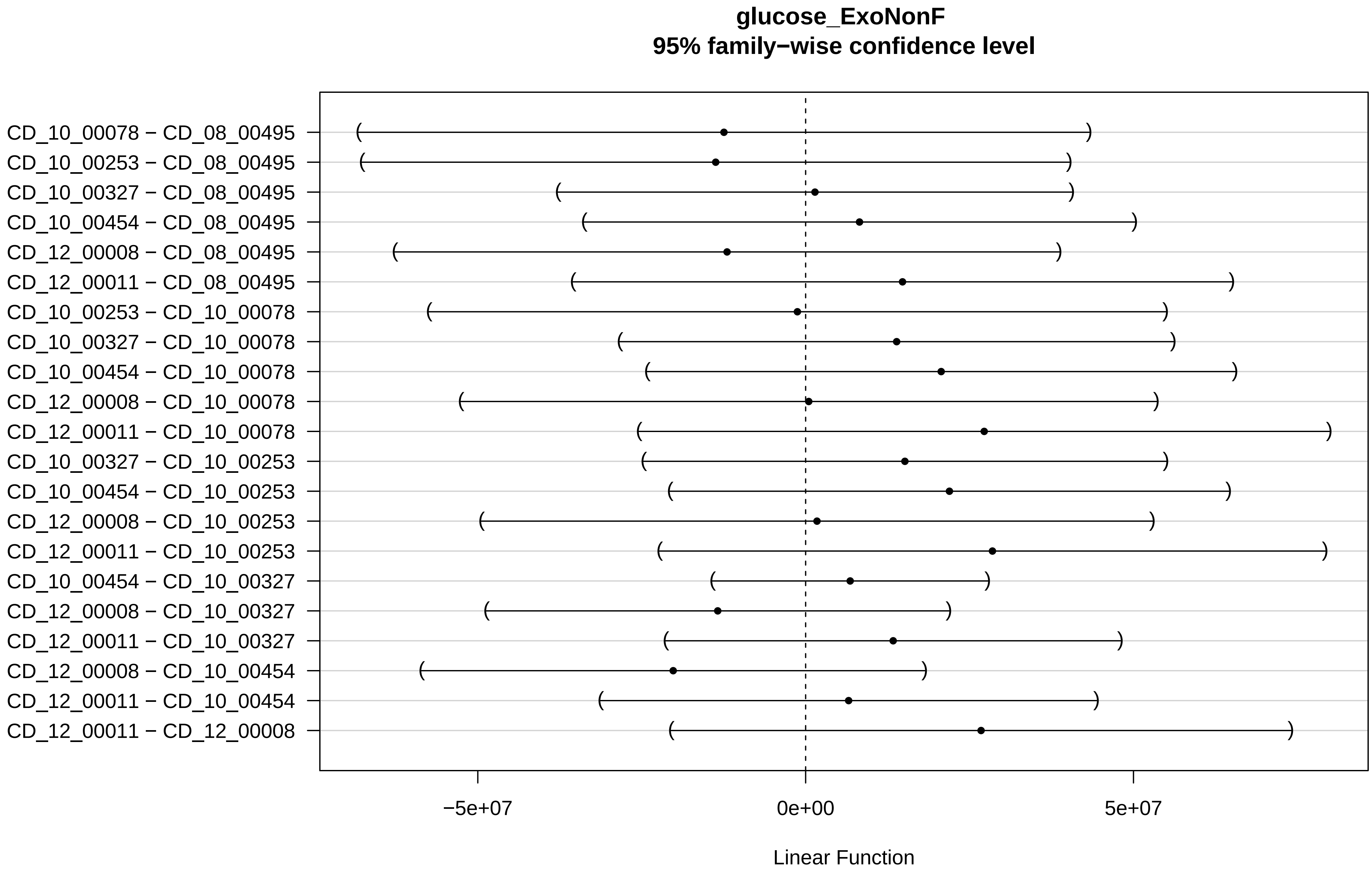
glucose_ExoNonF
95% family−wise confidence level
CD_10_00078 − CD_08_00495
CD_10_00253 − CD_08_00495
CD_10_00327 − CD_08_00495
CD_10_00454 − CD_08_00495
CD_12_00008 − CD_08_00495
CD_12_00011 − CD_08_00495
CD_10_00253 − CD_10_00078
CD_10_00327 − CD_10_00078
CD_10_00454 − CD_10_00078
CD_12_00008 − CD_10_00078
CD_12_00011 − CD_10_00078
CD_10_00327 − CD_10_00253
CD_10_00454 − CD_10_00253
CD_12_00008 − CD_10_00253
CD_12_00011 − CD_10_00253
CD_10_00454 − CD_10_00327
CD_12_00008 − CD_10_00327
CD_12_00011 − CD_10_00327
CD_12_00008 − CD_10_00454
CD_12_00011 − CD_10_00454
CD_12_00011 − CD_12_00008
−5e+07
0e+00
5e+07
Linear Function

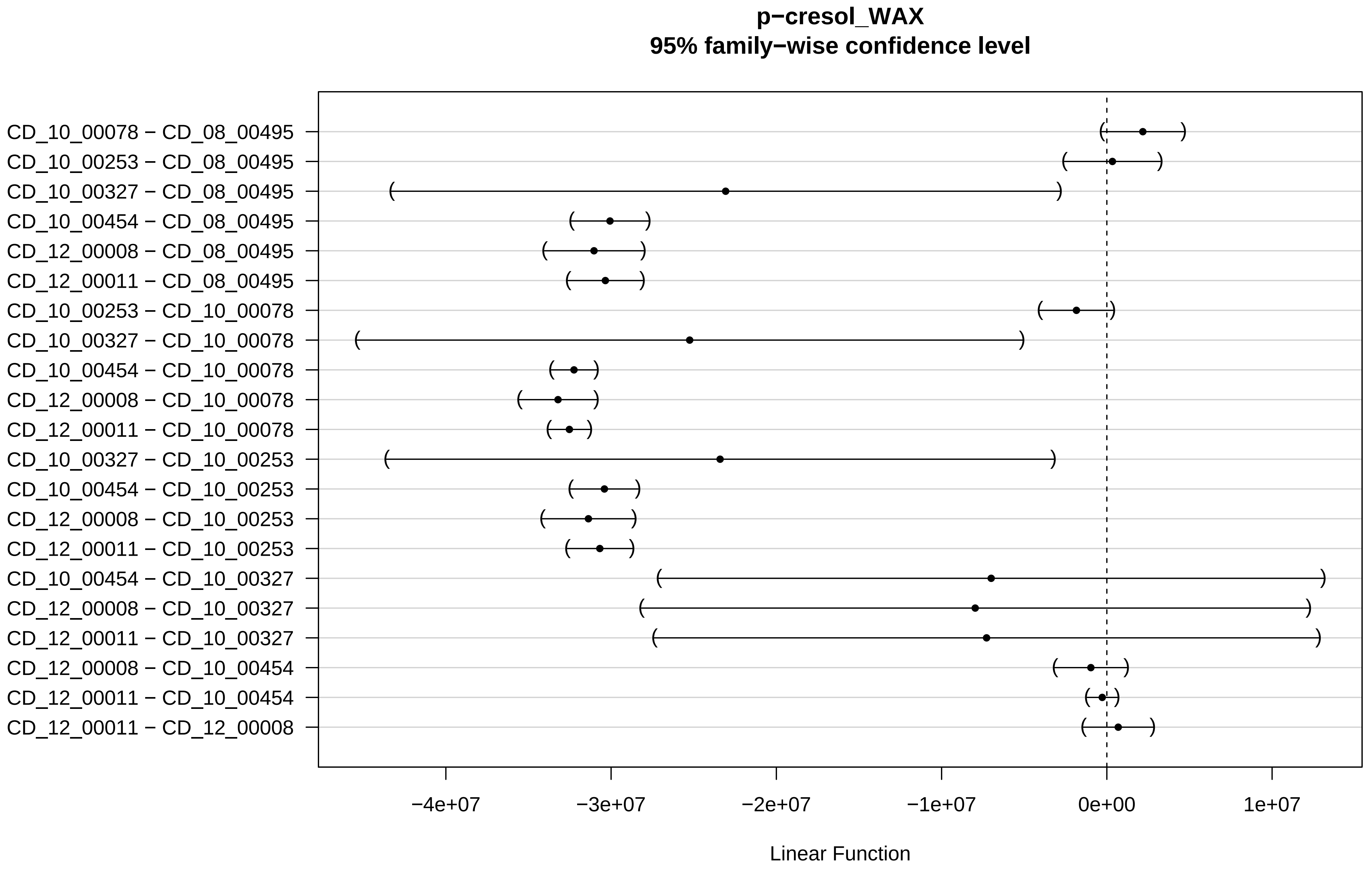

p−cresol_WAX
95% family−wise confidence level
CD_10_00078 − CD_08_00495
CD_10_00253 − CD_08_00495
CD_10_00327 − CD_08_00495
CD_10_00454 − CD_08_00495
CD_12_00008 − CD_08_00495
CD_12_00011 − CD_08_00495
CD_10_00253 − CD_10_00078
CD_10_00327 − CD_10_00078
CD_10_00454 − CD_10_00078
CD_12_00008 − CD_10_00078
CD_12_00011 − CD_10_00078
CD_10_00327 − CD_10_00253
CD_10_00454 − CD_10_00253
CD_12_00008 − CD_10_00253
CD_12_00011 − CD_10_00253
CD_10_00454 − CD_10_00327
CD_12_00008 − CD_10_00327
CD_12_00011 − CD_10_00327
CD_12_00008 − CD_10_00454
CD_12_00011 − CD_10_00454
CD_12_00011 − CD_12_00008
−4e+07
−3e+07
−2e+07
−1e+07
0e+00
1e+07
Linear Function

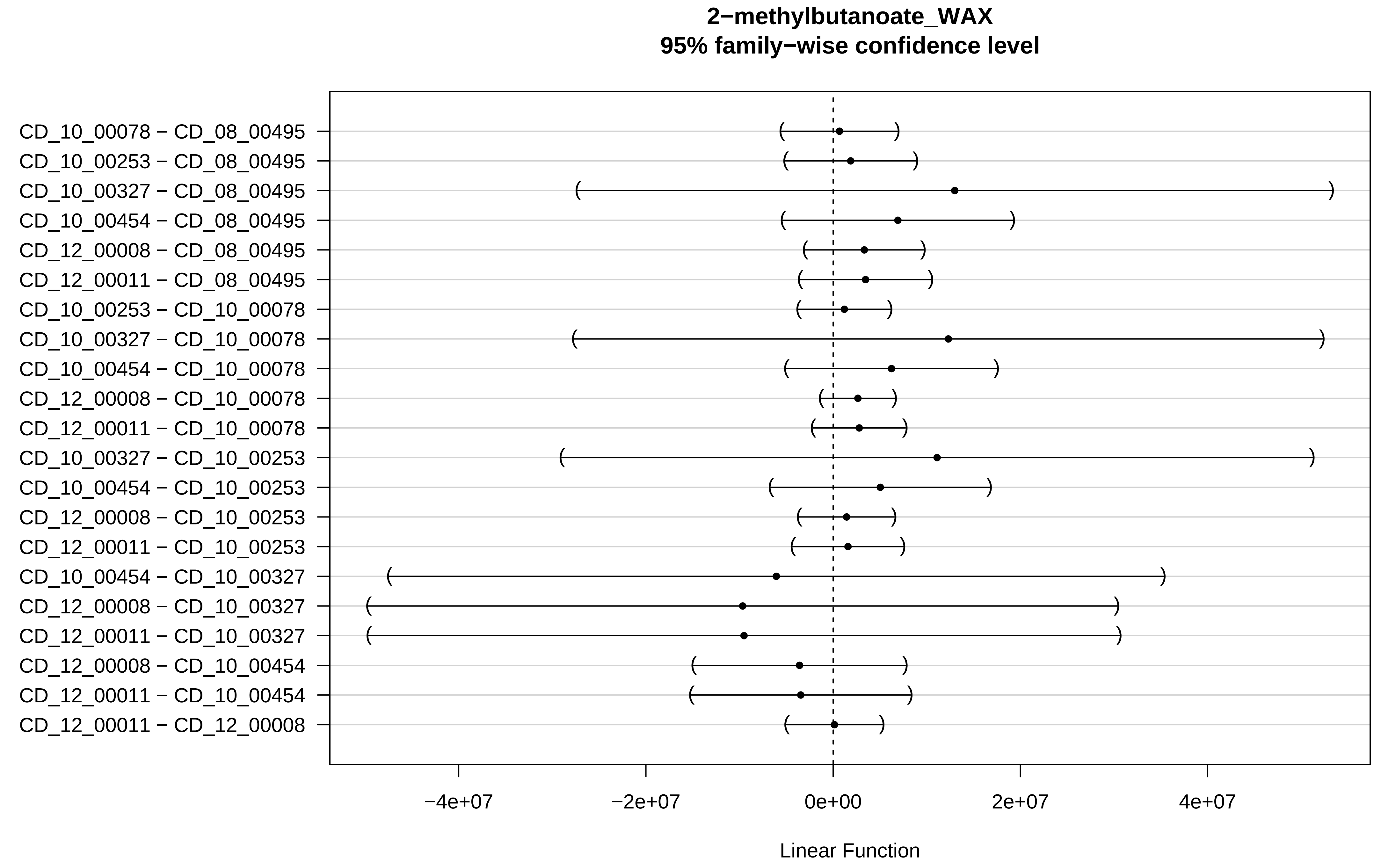
2−methylbutanoate_WAX
95% family−wise confidence level
CD_10_00078 − CD_08_00495
CD_10_00253 − CD_08_00495
CD_10_00327 − CD_08_00495
CD_10_00454 − CD_08_00495
CD_12_00008 − CD_08_00495
CD_12_00011 − CD_08_00495
CD_10_00253 − CD_10_00078
CD_10_00327 − CD_10_00078
CD_10_00454 − CD_10_00078
CD_12_00008 − CD_10_00078
CD_12_00011 − CD_10_00078
CD_10_00327 − CD_10_00253
CD_10_00454 − CD_10_00253
CD_12_00008 − CD_10_00253
CD_12_00011 − CD_10_00253
CD_10_00454 − CD_10_00327
CD_12_00008 − CD_10_00327
CD_12_00011 − CD_10_00327
CD_12_00008 − CD_10_00454
CD_12_00011 − CD_10_00454
CD_12_00011 − CD_12_00008
−4e+07
−2e+07
0e+00
2e+07
4e+07
Linear Function

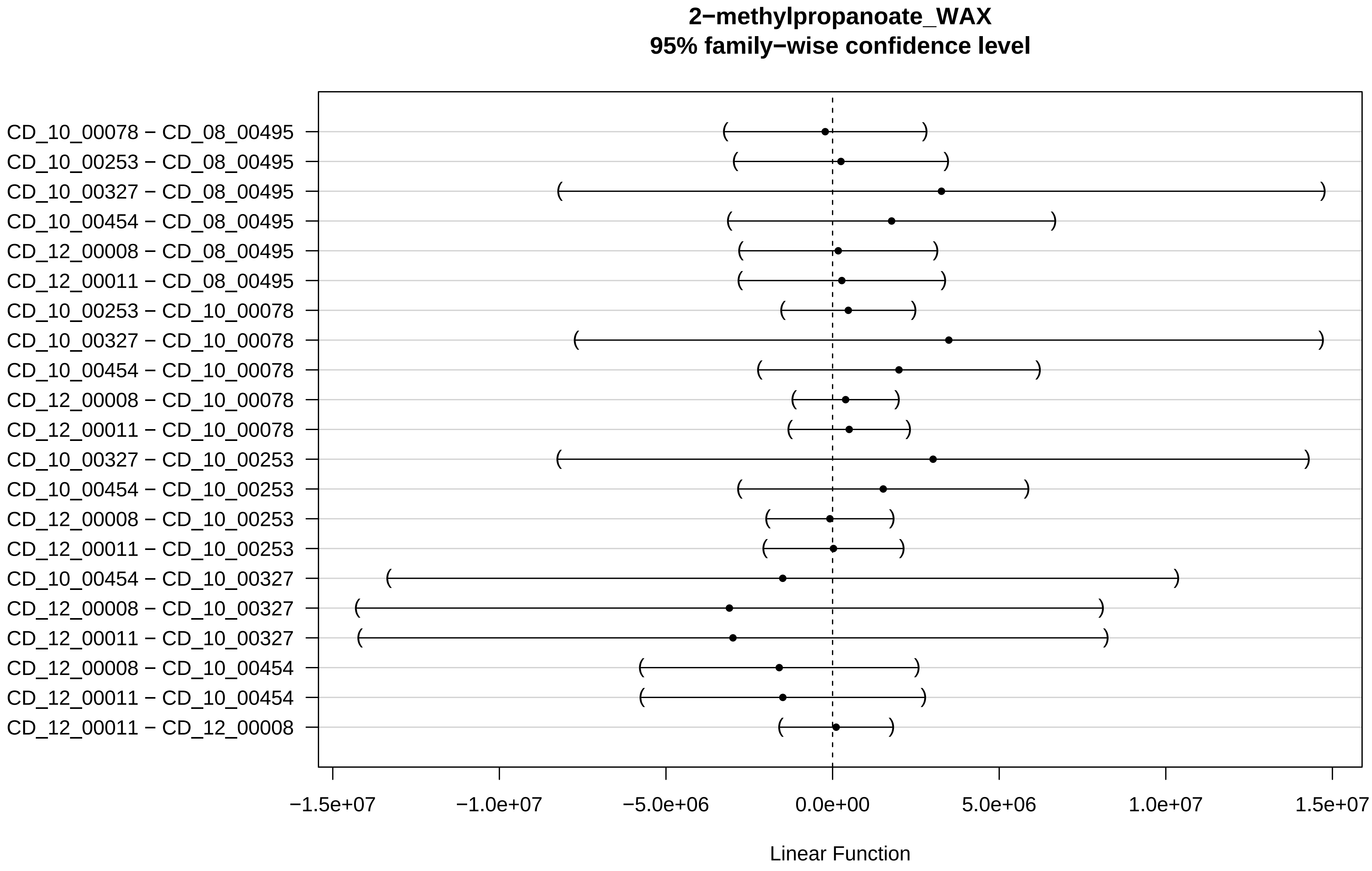

2−methylpropanoate_WAX
95% family−wise confidence level
CD_10_00078 − CD_08_00495
CD_10_00253 − CD_08_00495
CD_10_00327 − CD_08_00495
CD_10_00454 − CD_08_00495
CD_12_00008 − CD_08_00495
CD_12_00011 − CD_08_00495
CD_10_00253 − CD_10_00078
CD_10_00327 − CD_10_00078
CD_10_00454 − CD_10_00078
CD_12_00008 − CD_10_00078
CD_12_00011 − CD_10_00078
CD_10_00327 − CD_10_00253
CD_10_00454 − CD_10_00253
CD_12_00008 − CD_10_00253
CD_12_00011 − CD_10_00253
CD_10_00454 − CD_10_00327
CD_12_00008 − CD_10_00327
CD_12_00011 − CD_10_00327
CD_12_00008 − CD_10_00454
CD_12_00011 − CD_10_00454
CD_12_00011 − CD_12_00008
−1.5e+07
−1.0e+07
−5.0e+06
0.0e+00
5.0e+06
1.0e+07
1.5e+07
Linear Function

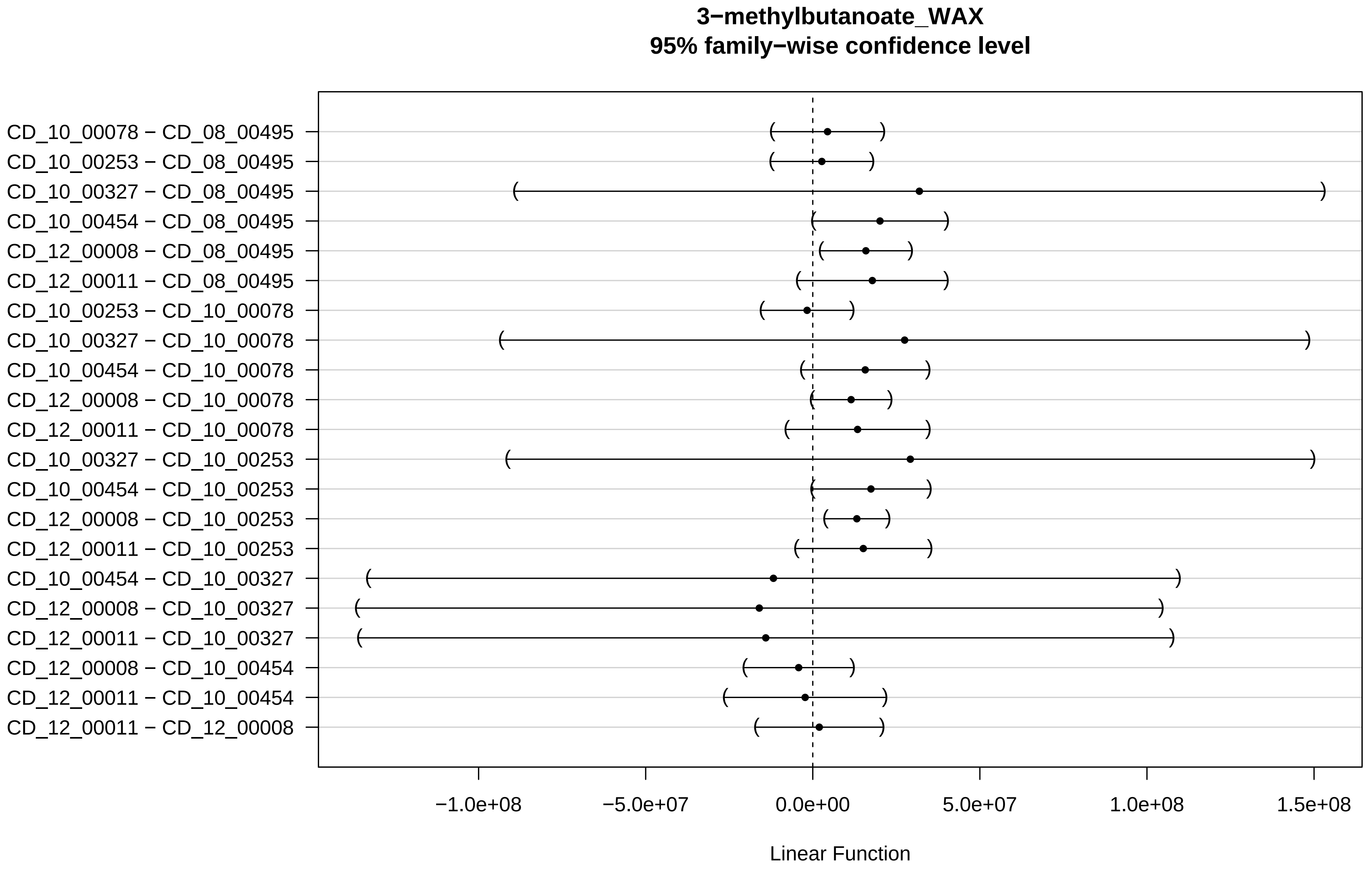

3−methylbutanoate_WAX
95% family−wise confidence level
CD_10_00078 − CD_08_00495
CD_10_00253 − CD_08_00495
CD_10_00327 − CD_08_00495
CD_10_00454 − CD_08_00495
CD_12_00008 − CD_08_00495
CD_12_00011 − CD_08_00495
CD_10_00253 − CD_10_00078
CD_10_00327 − CD_10_00078
CD_10_00454 − CD_10_00078
CD_12_00008 − CD_10_00078
CD_12_00011 − CD_10_00078
CD_10_00327 − CD_10_00253
CD_10_00454 − CD_10_00253
CD_12_00008 − CD_10_00253
CD_12_00011 − CD_10_00253
CD_10_00454 − CD_10_00327
CD_12_00008 − CD_10_00327
CD_12_00011 − CD_10_00327
CD_12_00008 − CD_10_00454
CD_12_00011 − CD_10_00454
CD_12_00011 − CD_12_00008
−1.0e+08
−5.0e+07
0.0e+00
5.0e+07
1.0e+08
1.5e+08
Linear Function

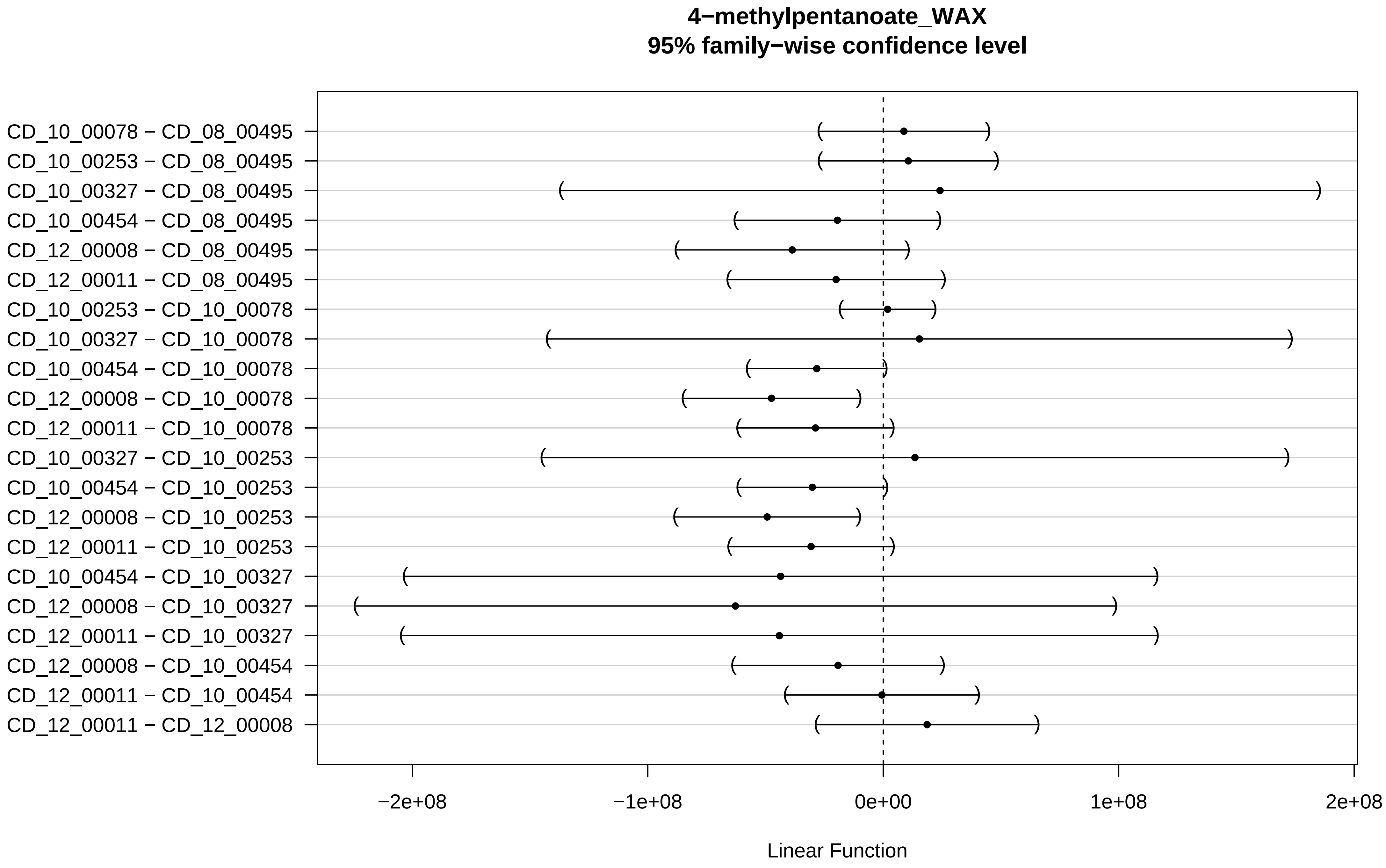
4−methylpentanoate_WAX
95% family−wise confidence level
CD_10_00078 − CD_08_00495
CD_10_00253 − CD_08_00495
CD_10_00327 − CD_08_00495
CD_10_00454 − CD_08_00495
CD_12_00008 − CD_08_00495
CD_12_00011 − CD_08_00495
CD_10_00253 − CD_10_00078
CD_10_00327 − CD_10_00078
CD_10_00454 − CD_10_00078
CD_12_00008 − CD_10_00078
CD_12_00011 − CD_10_00078
CD_10_00327 − CD_10_00253
CD_10_00454 − CD_10_00253
CD_12_00008 − CD_10_00253
CD_12_00011 − CD_10_00253
CD_10_00454 − CD_10_00327
CD_12_00008 − CD_10_00327
CD_12_00011 − CD_10_00327
CD_12_00008 − CD_10_00454
CD_12_00011 − CD_10_00454
CD_12_00011 − CD_12_00008
−2e+08
−1e+08
0e+00
1e+08
2e+08
Linear Function

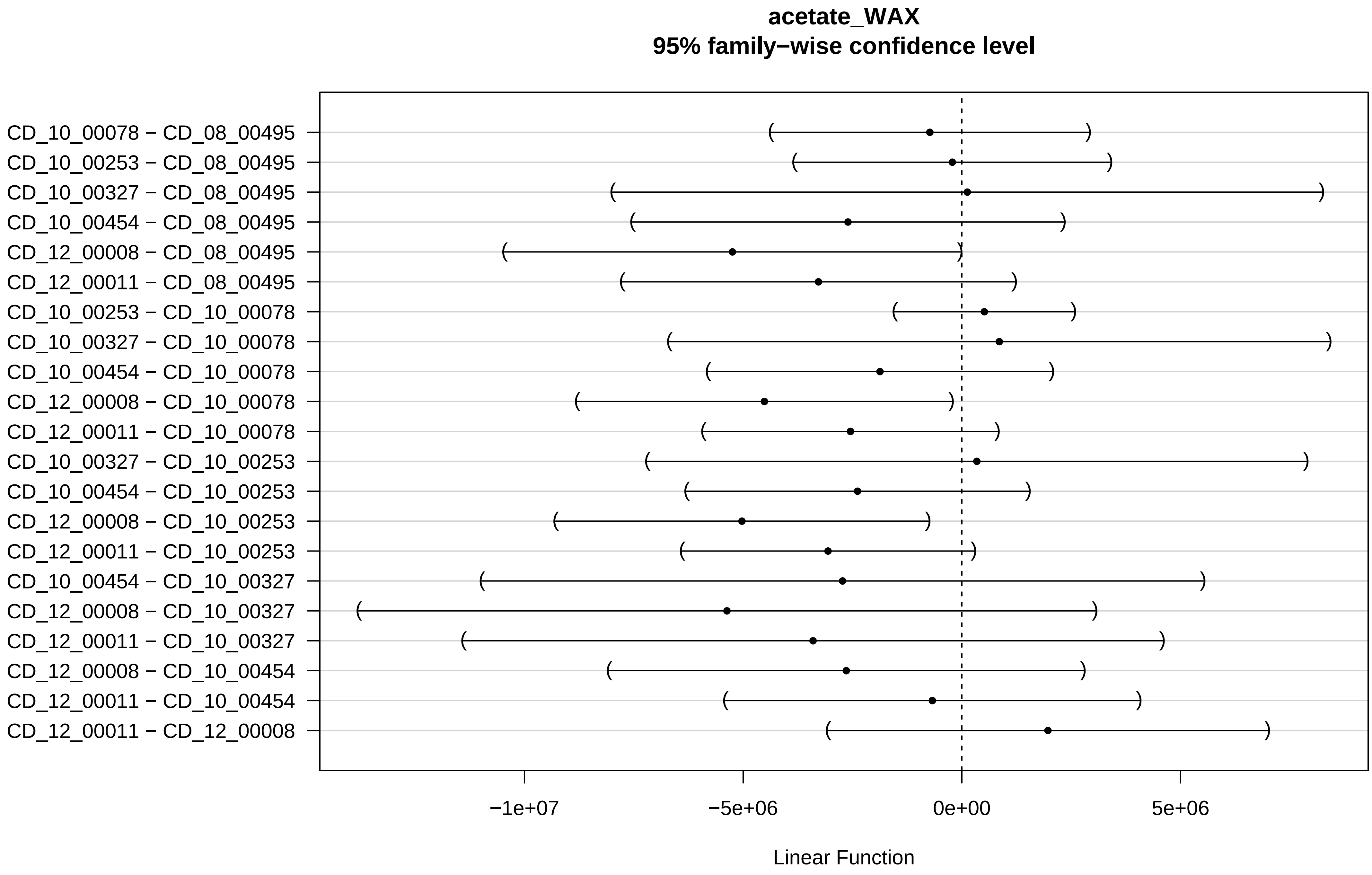
acetate_WAX
95% family−wise confidence level
CD_10_00078 − CD_08_00495
CD_10_00253 − CD_08_00495
CD_10_00327 − CD_08_00495
CD_10_00454 − CD_08_00495
CD_12_00008 − CD_08_00495
CD_12_00011 − CD_08_00495
CD_10_00253 − CD_10_00078
CD_10_00327 − CD_10_00078
CD_10_00454 − CD_10_00078
CD_12_00008 − CD_10_00078
CD_12_00011 − CD_10_00078
CD_10_00327 − CD_10_00253
CD_10_00454 − CD_10_00253
CD_12_00008 − CD_10_00253
CD_12_00011 − CD_10_00253
CD_10_00454 − CD_10_00327
CD_12_00008 − CD_10_00327
CD_12_00011 − CD_10_00327
CD_12_00008 − CD_10_00454
CD_12_00011 − CD_10_00454
CD_12_00011 − CD_12_00008
−1e+07
−5e+06
0e+00
5e+06
Linear Function

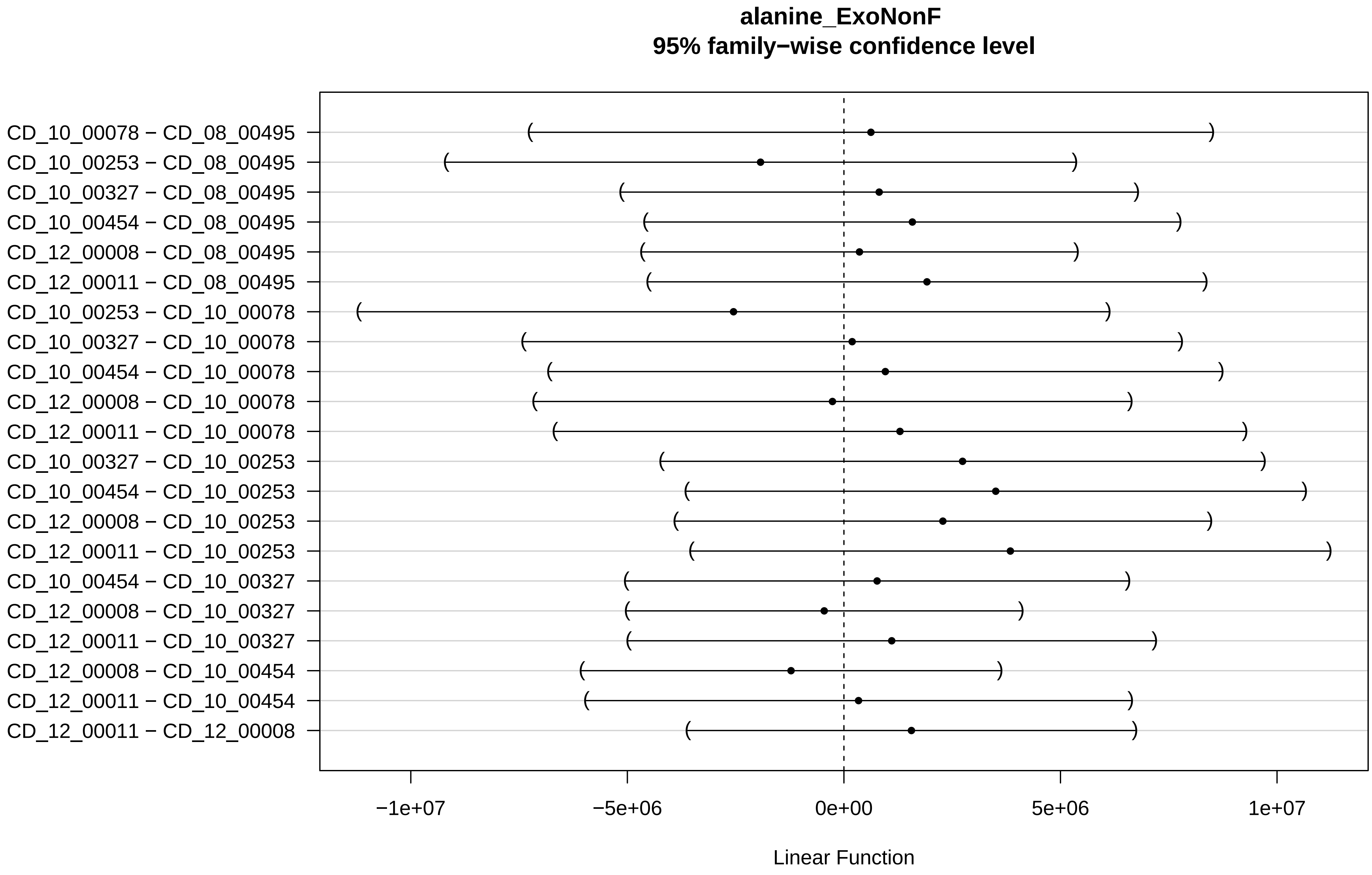
alanine_ExoNonF
95% family−wise confidence level
CD_10_00078 − CD_08_00495
CD_10_00253 − CD_08_00495
CD_10_00327 − CD_08_00495
CD_10_00454 − CD_08_00495
CD_12_00008 − CD_08_00495
CD_12_00011 − CD_08_00495
CD_10_00253 − CD_10_00078
CD_10_00327 − CD_10_00078
CD_10_00454 − CD_10_00078
CD_12_00008 − CD_10_00078
CD_12_00011 − CD_10_00078
CD_10_00327 − CD_10_00253
CD_10_00454 − CD_10_00253
CD_12_00008 − CD_10_00253
CD_12_00011 − CD_10_00253
CD_10_00454 − CD_10_00327
CD_12_00008 − CD_10_00327
CD_12_00011 − CD_10_00327
CD_12_00008 − CD_10_00454
CD_12_00011 − CD_10_00454
CD_12_00011 − CD_12_00008
−1e+07
−5e+06
0e+00
5e+06
1e+07
Linear Function

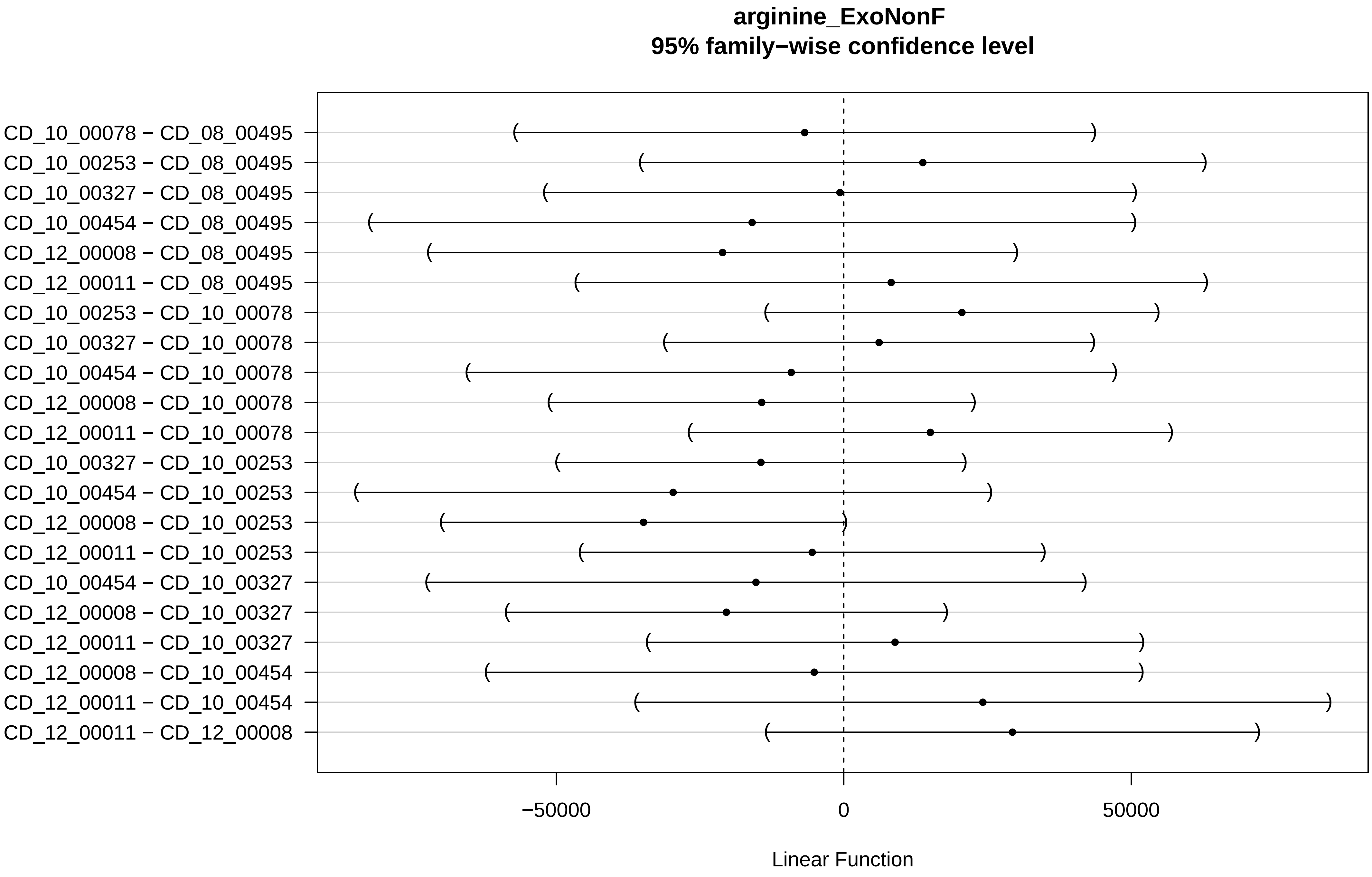
arginine_ExoNonF
95% family−wise confidence level
CD_10_00078 − CD_08_00495
CD_10_00253 − CD_08_00495
CD_10_00327 − CD_08_00495
CD_10_00454 − CD_08_00495
CD_12_00008 − CD_08_00495
CD_12_00011 − CD_08_00495
CD_10_00253 − CD_10_00078
CD_10_00327 − CD_10_00078
CD_10_00454 − CD_10_00078
CD_12_00008 − CD_10_00078
CD_12_00011 − CD_10_00078
CD_10_00327 − CD_10_00253
CD_10_00454 − CD_10_00253
CD_12_00008 − CD_10_00253
CD_12_00011 − CD_10_00253
CD_10_00454 − CD_10_00327
CD_12_00008 − CD_10_00327
CD_12_00011 − CD_10_00327
CD_12_00008 − CD_10_00454
CD_12_00011 − CD_10_00454
CD_12_00011 − CD_12_00008
−50000
0
50000
Linear Function

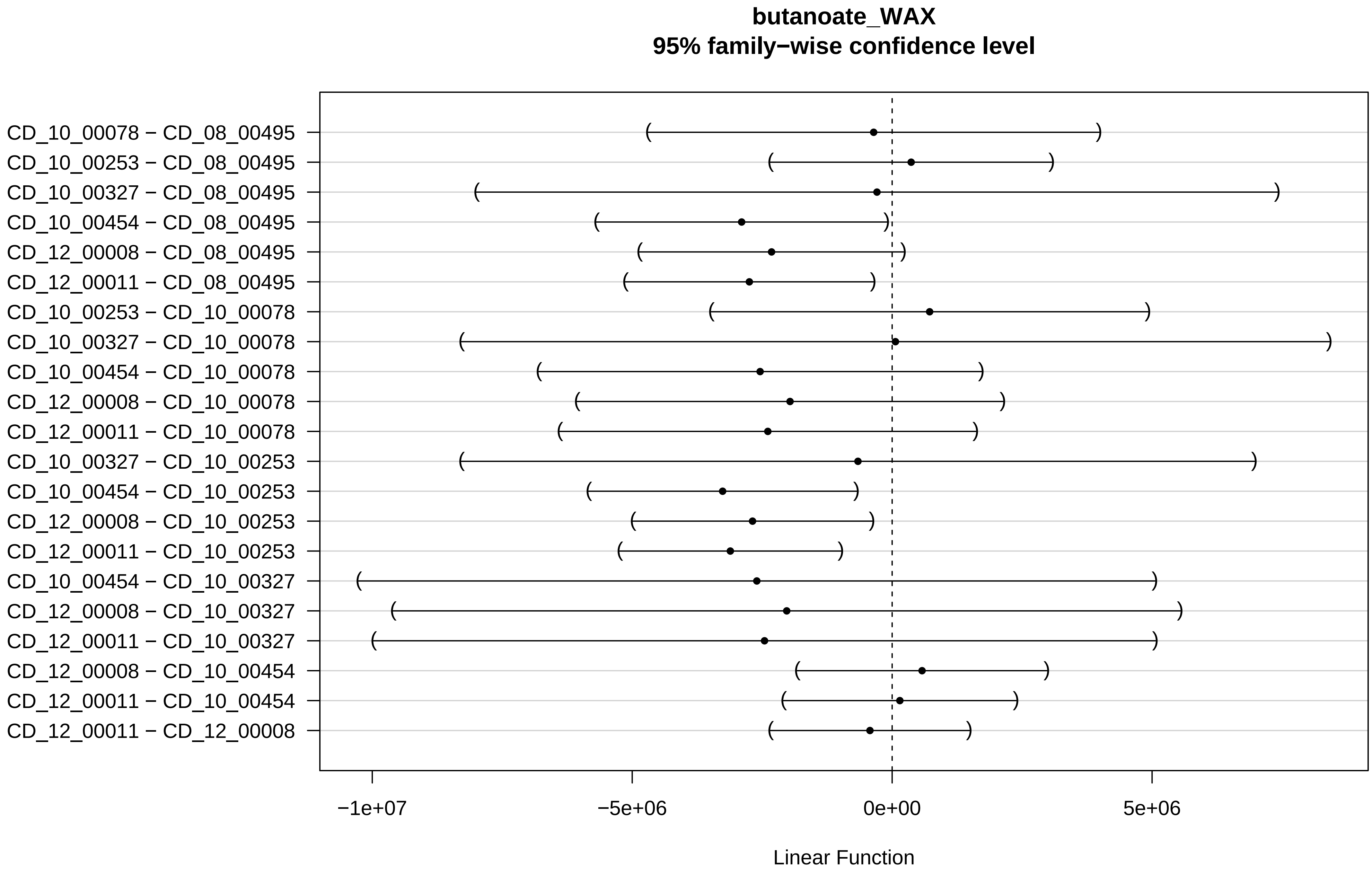

butanoate_WAX
95% family−wise confidence level
CD_10_00078 − CD_08_00495
CD_10_00253 − CD_08_00495
CD_10_00327 − CD_08_00495
CD_10_00454 − CD_08_00495
CD_12_00008 − CD_08_00495
CD_12_00011 − CD_08_00495
CD_10_00253 − CD_10_00078
CD_10_00327 − CD_10_00078
CD_10_00454 − CD_10_00078
CD_12_00008 − CD_10_00078
CD_12_00011 − CD_10_00078
CD_10_00327 − CD_10_00253
CD_10_00454 − CD_10_00253
CD_12_00008 − CD_10_00253
CD_12_00011 − CD_10_00253
CD_10_00454 − CD_10_00327
CD_12_00008 − CD_10_00327
CD_12_00011 − CD_10_00327
CD_12_00008 − CD_10_00454
CD_12_00011 − CD_10_00454
CD_12_00011 − CD_12_00008
−1e+07
−5e+06
0e+00
5e+06
Linear Function

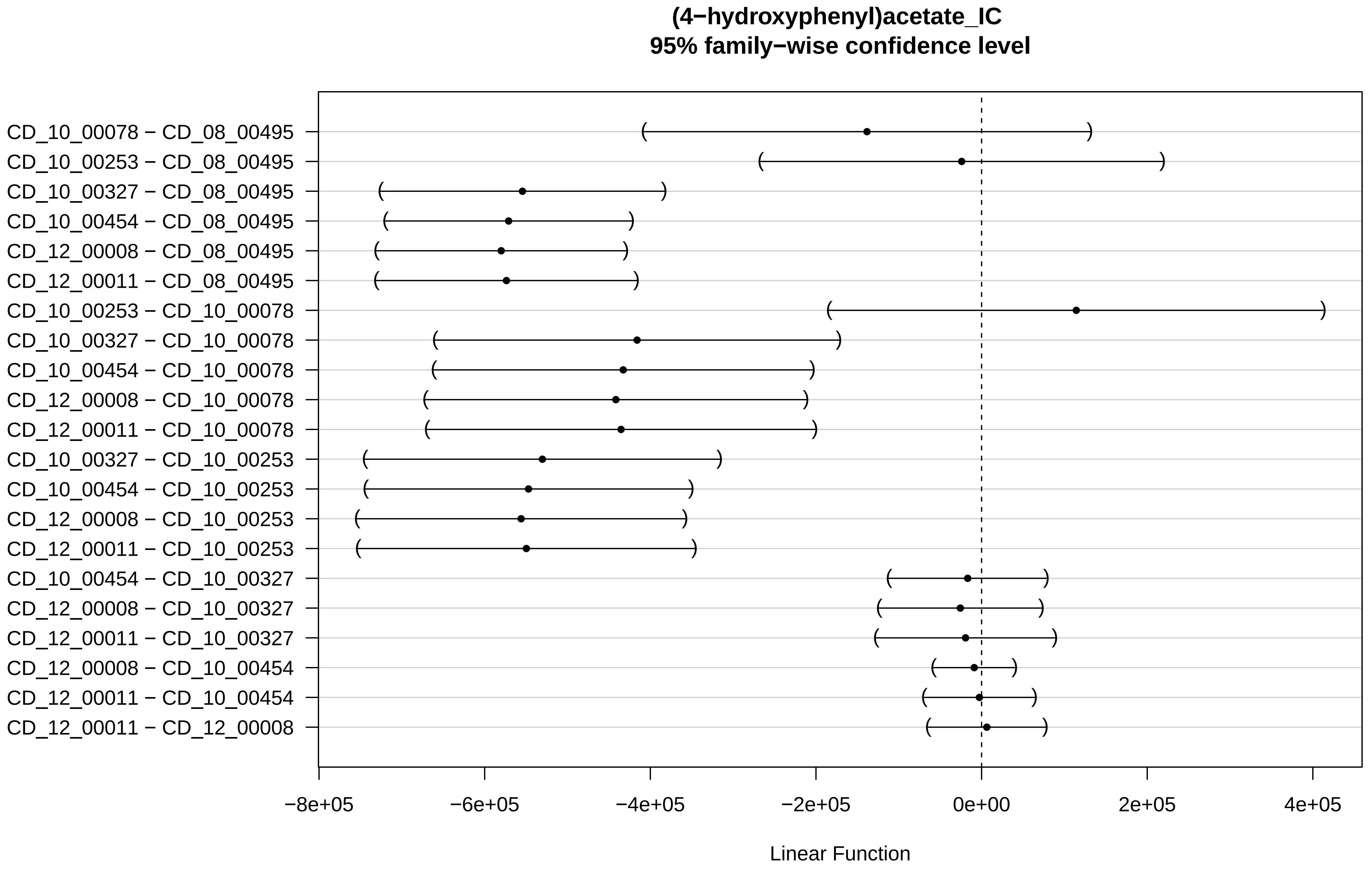
(4−hydroxyphenyl)acetate_IC
95% family−wise confidence level
CD_10_00078 − CD_08_00495
CD_10_00253 − CD_08_00495
CD_10_00327 − CD_08_00495
CD_10_00454 − CD_08_00495
CD_12_00008 − CD_08_00495
CD_12_00011 − CD_08_00495
CD_10_00253 − CD_10_00078
CD_10_00327 − CD_10_00078
CD_10_00454 − CD_10_00078
CD_12_00008 − CD_10_00078
CD_12_00011 − CD_10_00078
CD_10_00327 − CD_10_00253
CD_10_00454 − CD_10_00253
CD_12_00008 − CD_10_00253
CD_12_00011 − CD_10_00253
CD_10_00454 − CD_10_00327
CD_12_00008 − CD_10_00327
CD_12_00011 − CD_10_00327
CD_12_00008 − CD_10_00454
CD_12_00011 − CD_10_00454
CD_12_00011 − CD_12_00008
−8e+05
−6e+05
−4e+05
−2e+05
0e+00
2e+05
4e+05
Linear Function

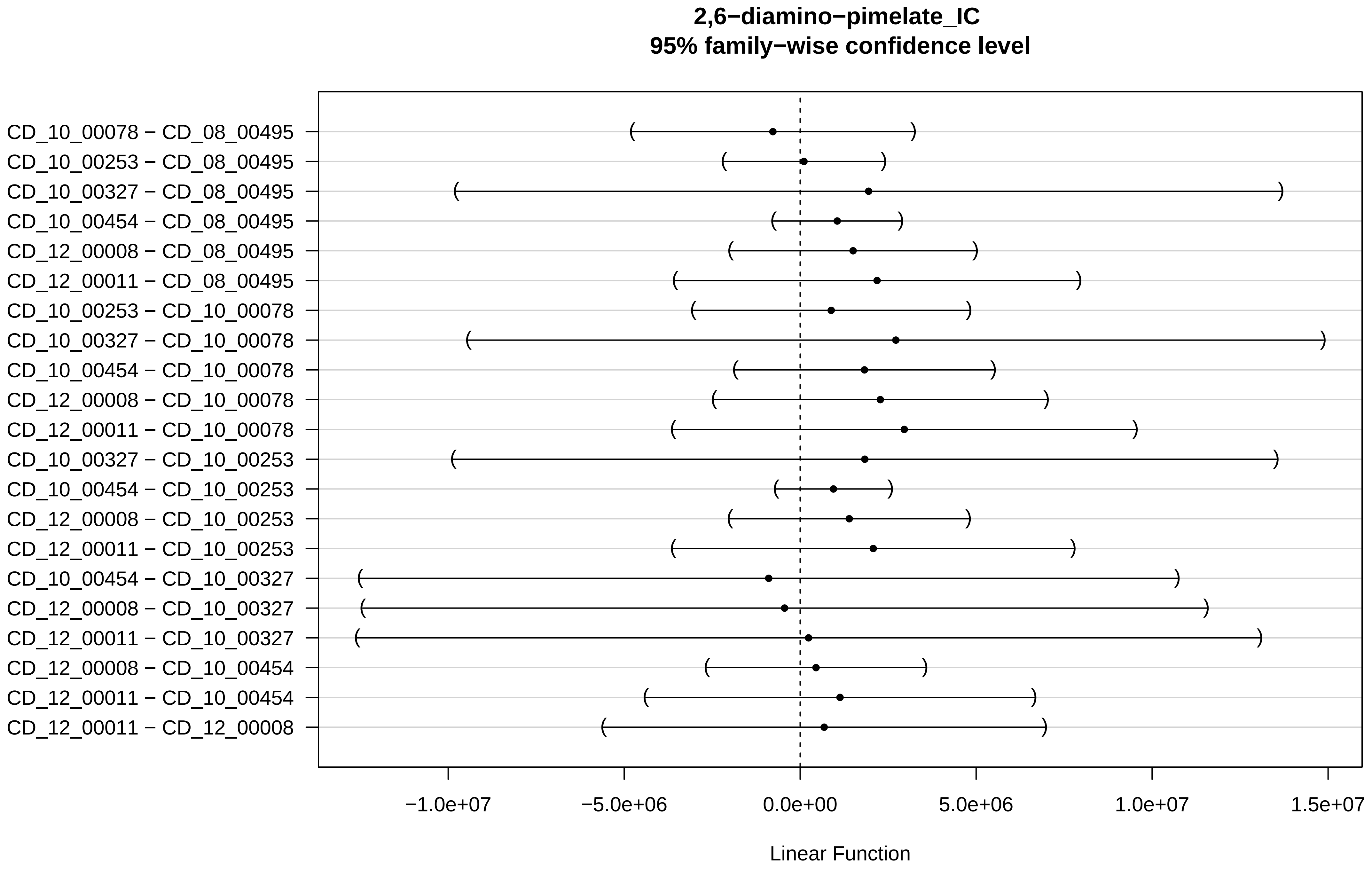
2,6−diamino−pimelate_IC
95% family−wise confidence level
CD_10_00078 − CD_08_00495
CD_10_00253 − CD_08_00495
CD_10_00327 − CD_08_00495
CD_10_00454 − CD_08_00495
CD_12_00008 − CD_08_00495
CD_12_00011 − CD_08_00495
CD_10_00253 − CD_10_00078
CD_10_00327 − CD_10_00078
CD_10_00454 − CD_10_00078
CD_12_00008 − CD_10_00078
CD_12_00011 − CD_10_00078
CD_10_00327 − CD_10_00253
CD_10_00454 − CD_10_00253
CD_12_00008 − CD_10_00253
CD_12_00011 − CD_10_00253
CD_10_00454 − CD_10_00327
CD_12_00008 − CD_10_00327
CD_12_00011 − CD_10_00327
CD_12_00008 − CD_10_00454
CD_12_00011 − CD_10_00454
CD_12_00011 − CD_12_00008
−1.0e+07
−5.0e+06
0.0e+00
5.0e+06
1.0e+07
1.5e+07
Linear Function

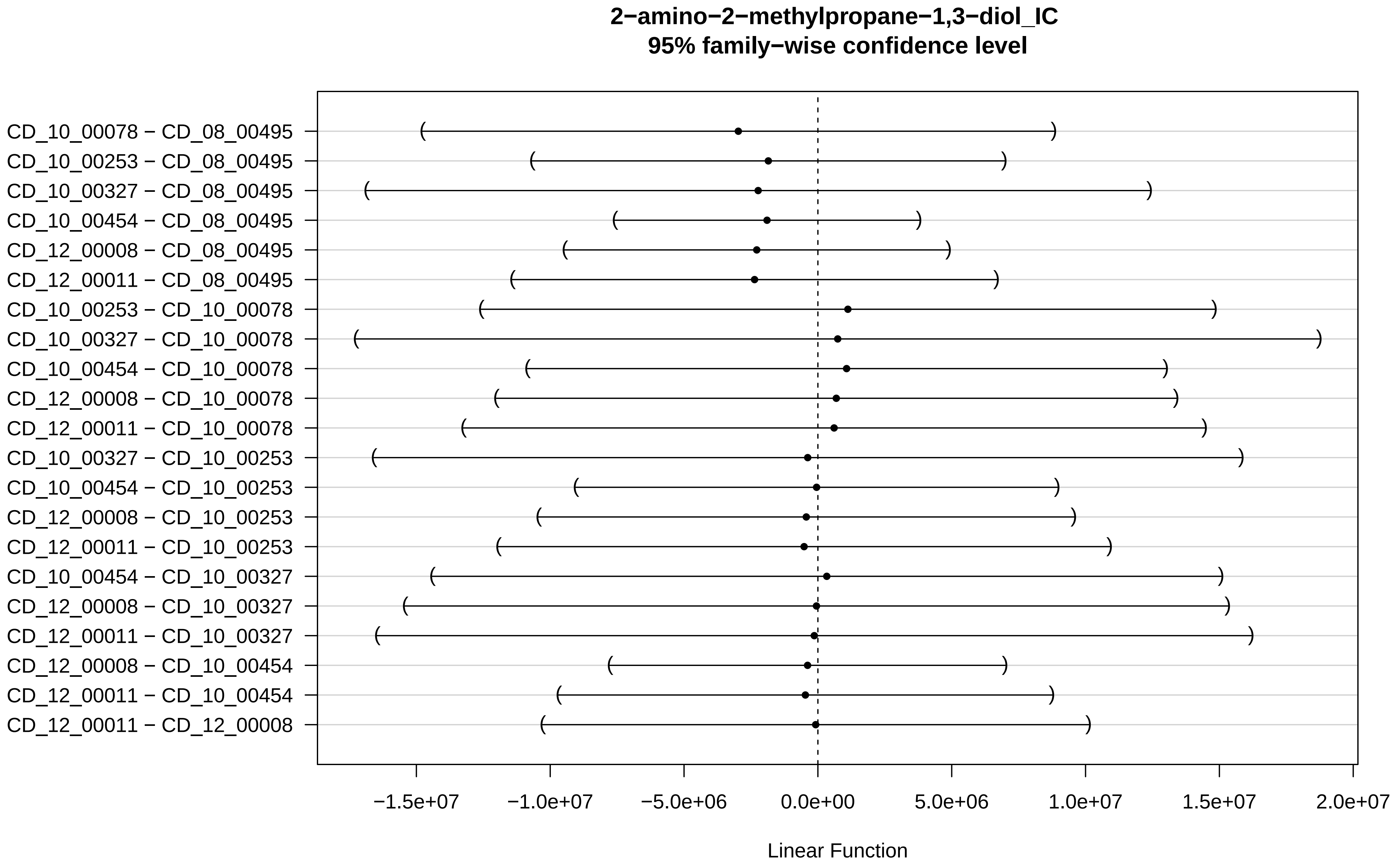
2−amino−2−methylpropane−1,3−diol_IC
95% family−wise confidence level
CD_10_00078 − CD_08_00495
CD_10_00253 − CD_08_00495
CD_10_00327 − CD_08_00495
CD_10_00454 − CD_08_00495
CD_12_00008 − CD_08_00495
CD_12_00011 − CD_08_00495
CD_10_00253 − CD_10_00078
CD_10_00327 − CD_10_00078
CD_10_00454 − CD_10_00078
CD_12_00008 − CD_10_00078
CD_12_00011 − CD_10_00078
CD_10_00327 − CD_10_00253
CD_10_00454 − CD_10_00253
CD_12_00008 − CD_10_00253
CD_12_00011 − CD_10_00253
CD_10_00454 − CD_10_00327
CD_12_00008 − CD_10_00327
CD_12_00011 − CD_10_00327
CD_12_00008 − CD_10_00454
CD_12_00011 − CD_10_00454
CD_12_00011 − CD_12_00008
−1.5e+07
−1.0e+07
−5.0e+06
0.0e+00
5.0e+06
1.0e+07
1.5e+07
2.0e+07
Linear Function

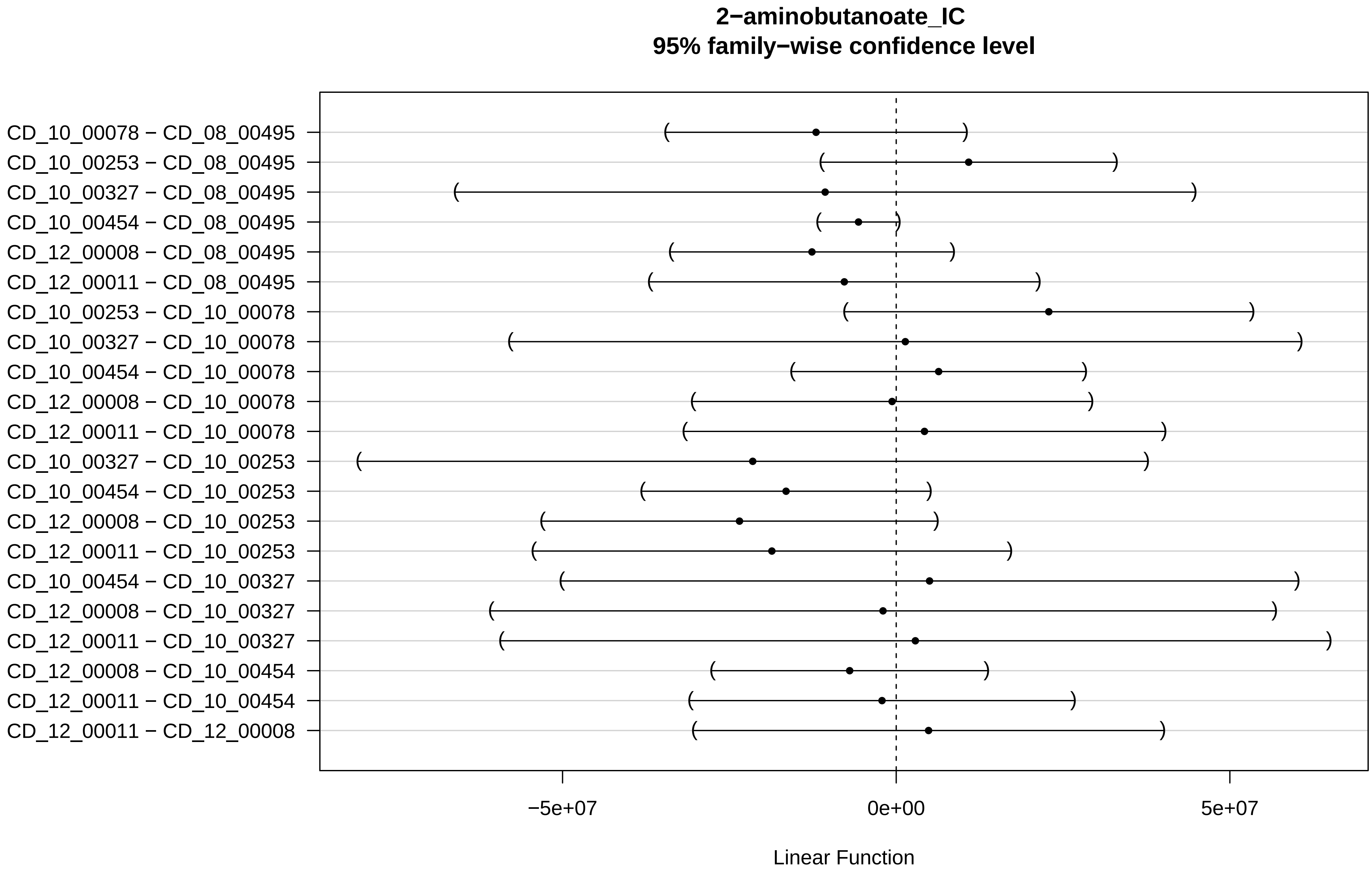
2−aminobutanoate_IC
95% family−wise confidence level
CD_10_00078 − CD_08_00495
CD_10_00253 − CD_08_00495
CD_10_00327 − CD_08_00495
CD_10_00454 − CD_08_00495
CD_12_00008 − CD_08_00495
CD_12_00011 − CD_08_00495
CD_10_00253 − CD_10_00078
CD_10_00327 − CD_10_00078
CD_10_00454 − CD_10_00078
CD_12_00008 − CD_10_00078
CD_12_00011 − CD_10_00078
CD_10_00327 − CD_10_00253
CD_10_00454 − CD_10_00253
CD_12_00008 − CD_10_00253
CD_12_00011 − CD_10_00253
CD_10_00454 − CD_10_00327
CD_12_00008 − CD_10_00327
CD_12_00011 − CD_10_00327
CD_12_00008 − CD_10_00454
CD_12_00011 − CD_10_00454
CD_12_00011 − CD_12_00008
−5e+07
0e+00
5e+07
Linear Function

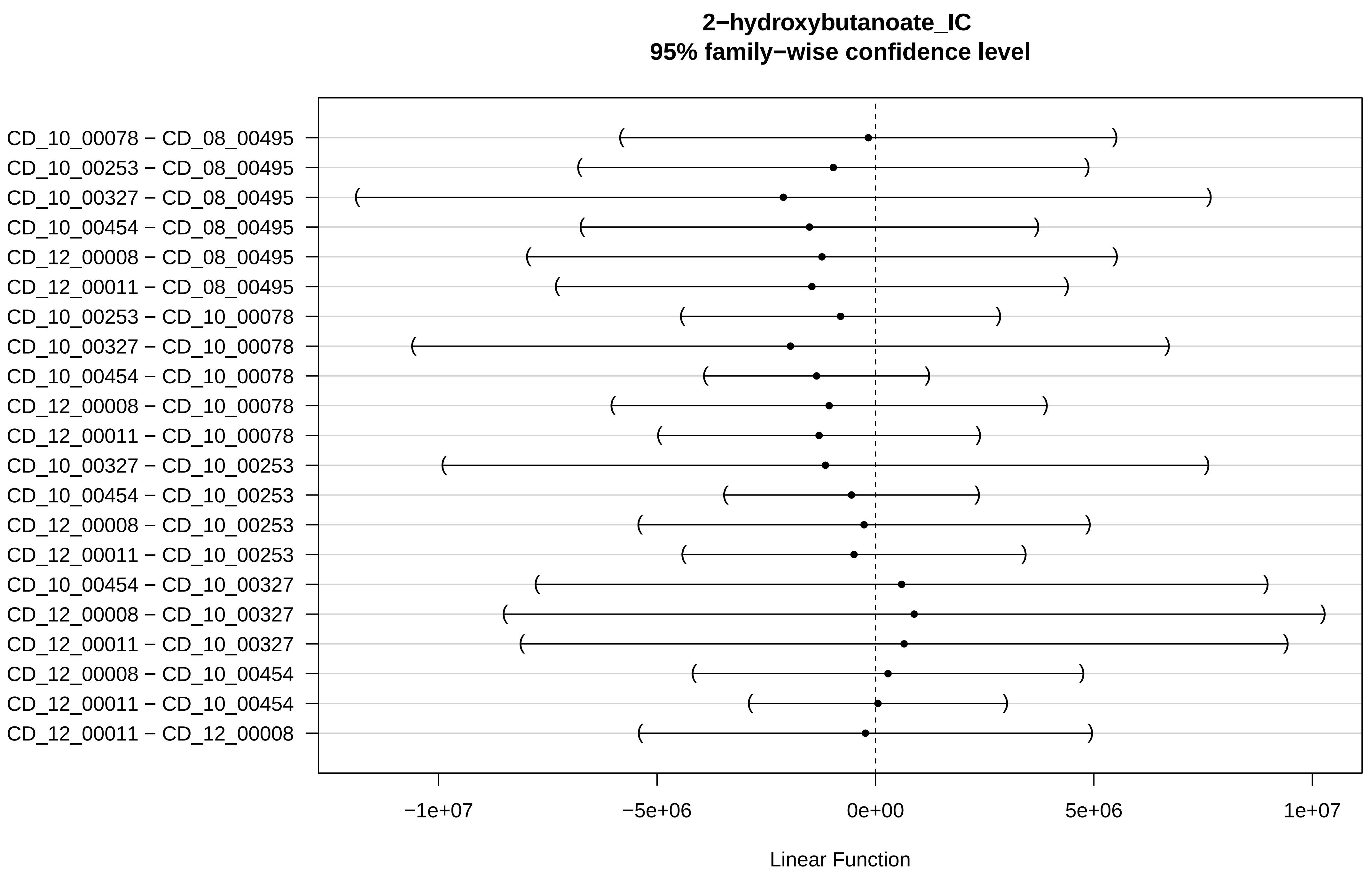

2−hydroxybutanoate_IC
95% family−wise confidence level
CD_10_00078 − CD_08_00495
CD_10_00253 − CD_08_00495
CD_10_00327 − CD_08_00495
CD_10_00454 − CD_08_00495
CD_12_00008 − CD_08_00495
CD_12_00011 − CD_08_00495
CD_10_00253 − CD_10_00078
CD_10_00327 − CD_10_00078
CD_10_00454 − CD_10_00078
CD_12_00008 − CD_10_00078
CD_12_00011 − CD_10_00078
CD_10_00327 − CD_10_00253
CD_10_00454 − CD_10_00253
CD_12_00008 − CD_10_00253
CD_12_00011 − CD_10_00253
CD_10_00454 − CD_10_00327
CD_12_00008 − CD_10_00327
CD_12_00011 − CD_10_00327
CD_12_00008 − CD_10_00454
CD_12_00011 − CD_10_00454
CD_12_00011 − CD_12_00008
−1e+07
−5e+06
0e+00
5e+06
1e+07
Linear Function

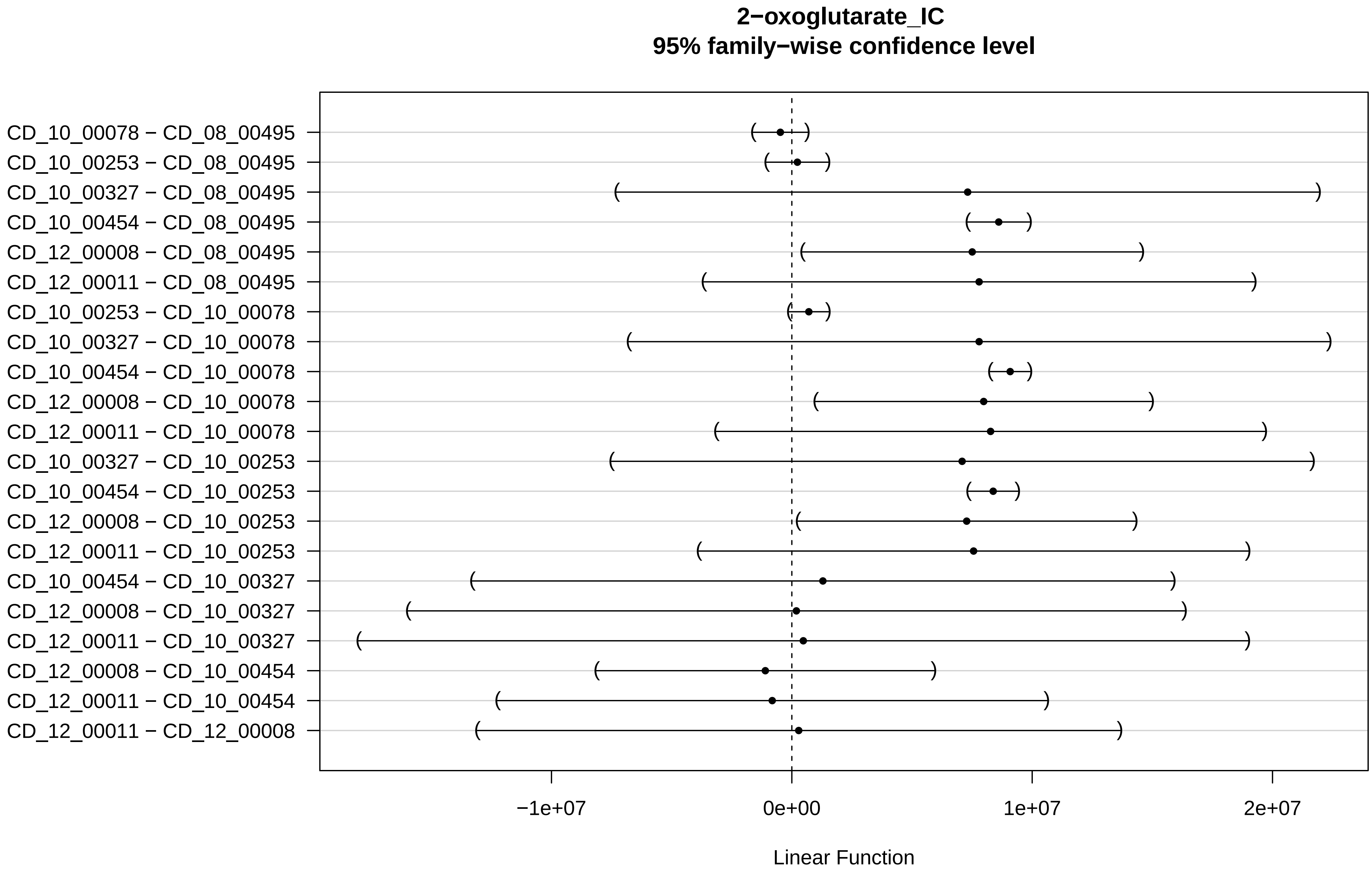

2−oxoglutarate_IC
95% family−wise confidence level
CD_10_00078 − CD_08_00495
CD_10_00253 − CD_08_00495
CD_10_00327 − CD_08_00495
CD_10_00454 − CD_08_00495
CD_12_00008 − CD_08_00495
CD_12_00011 − CD_08_00495
CD_10_00253 − CD_10_00078
CD_10_00327 − CD_10_00078
CD_10_00454 − CD_10_00078
CD_12_00008 − CD_10_00078
CD_12_00011 − CD_10_00078
CD_10_00327 − CD_10_00253
CD_10_00454 − CD_10_00253
CD_12_00008 − CD_10_00253
CD_12_00011 − CD_10_00253
CD_10_00454 − CD_10_00327
CD_12_00008 − CD_10_00327
CD_12_00011 − CD_10_00327
CD_12_00008 − CD_10_00454
CD_12_00011 − CD_10_00454
CD_12_00011 − CD_12_00008
−1e+07
0e+00
1e+07
2e+07
Linear Function

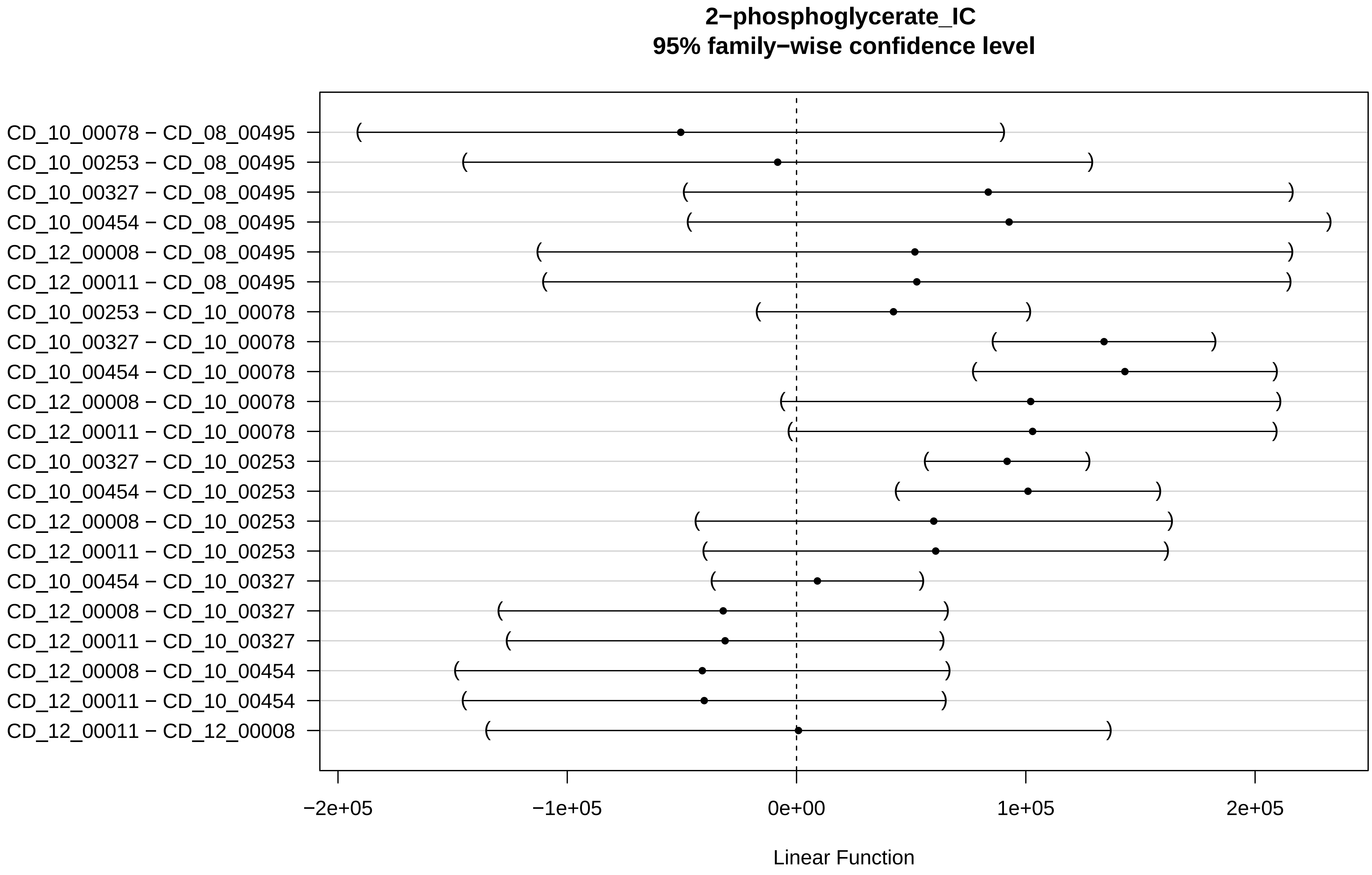
2−phosphoglycerate_IC
95% family−wise confidence level
CD_10_00078 − CD_08_00495
CD_10_00253 − CD_08_00495
CD_10_00327 − CD_08_00495
CD_10_00454 − CD_08_00495
CD_12_00008 − CD_08_00495
CD_12_00011 − CD_08_00495
CD_10_00253 − CD_10_00078
CD_10_00327 − CD_10_00078
CD_10_00454 − CD_10_00078
CD_12_00008 − CD_10_00078
CD_12_00011 − CD_10_00078
CD_10_00327 − CD_10_00253
CD_10_00454 − CD_10_00253
CD_12_00008 − CD_10_00253
CD_12_00011 − CD_10_00253
CD_10_00454 − CD_10_00327
CD_12_00008 − CD_10_00327
CD_12_00011 − CD_10_00327
CD_12_00008 − CD_10_00454
CD_12_00011 − CD_10_00454
CD_12_00011 − CD_12_00008
−2e+05
−1e+05
0e+00
1e+05
2e+05
Linear Function

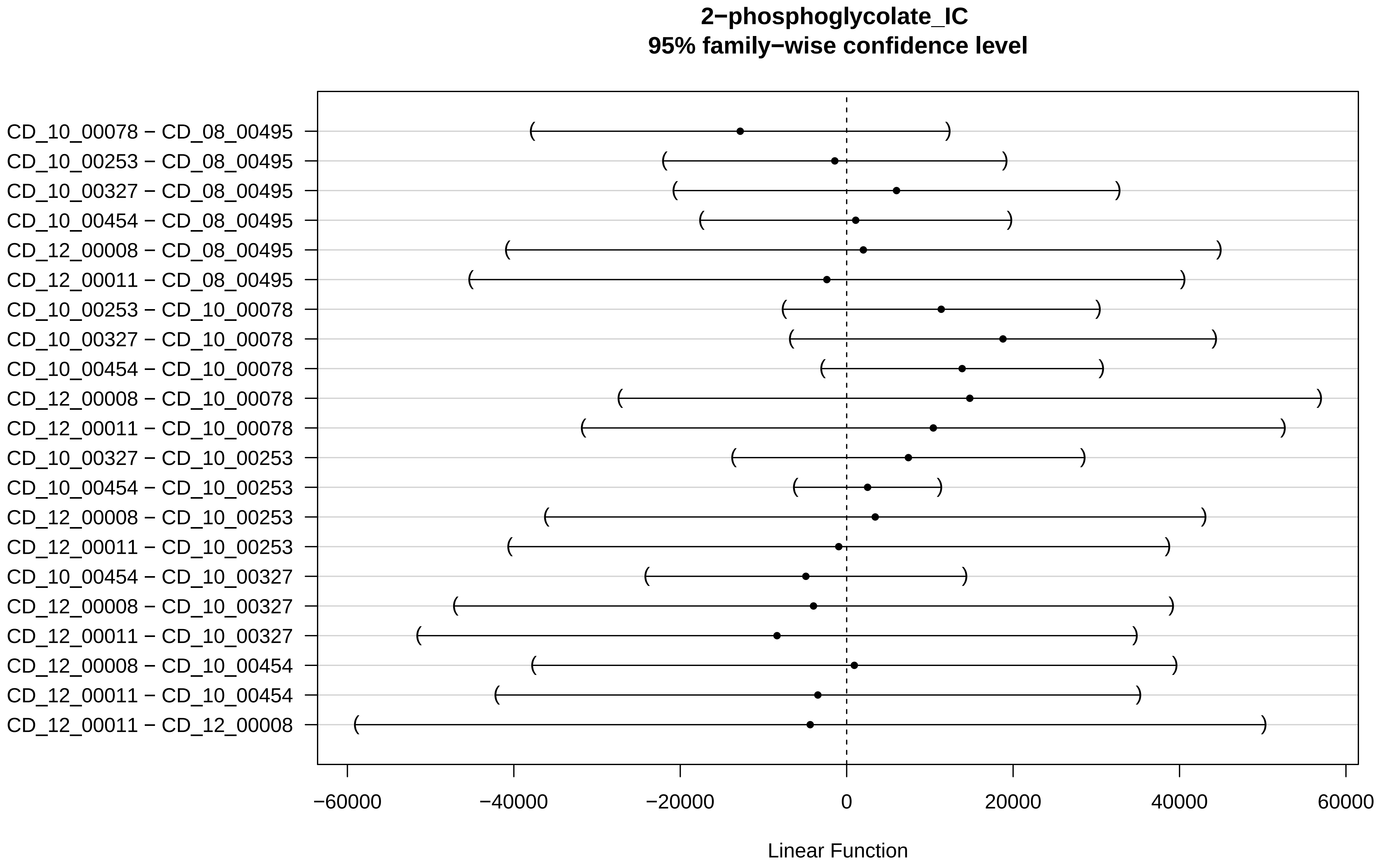

2−phosphoglycolate_IC
95% family−wise confidence level
CD_10_00078 − CD_08_00495
CD_10_00253 − CD_08_00495
CD_10_00327 − CD_08_00495
CD_10_00454 − CD_08_00495
CD_12_00008 − CD_08_00495
CD_12_00011 − CD_08_00495
CD_10_00253 − CD_10_00078
CD_10_00327 − CD_10_00078
CD_10_00454 − CD_10_00078
CD_12_00008 − CD_10_00078
CD_12_00011 − CD_10_00078
CD_10_00327 − CD_10_00253
CD_10_00454 − CD_10_00253
CD_12_00008 − CD_10_00253
CD_12_00011 − CD_10_00253
CD_10_00454 − CD_10_00327
CD_12_00008 − CD_10_00327
CD_12_00011 − CD_10_00327
CD_12_00008 − CD_10_00454
CD_12_00011 − CD_10_00454
CD_12_00011 − CD_12_00008
−60000
−40000
−20000
0
20000
40000
60000
Linear Function

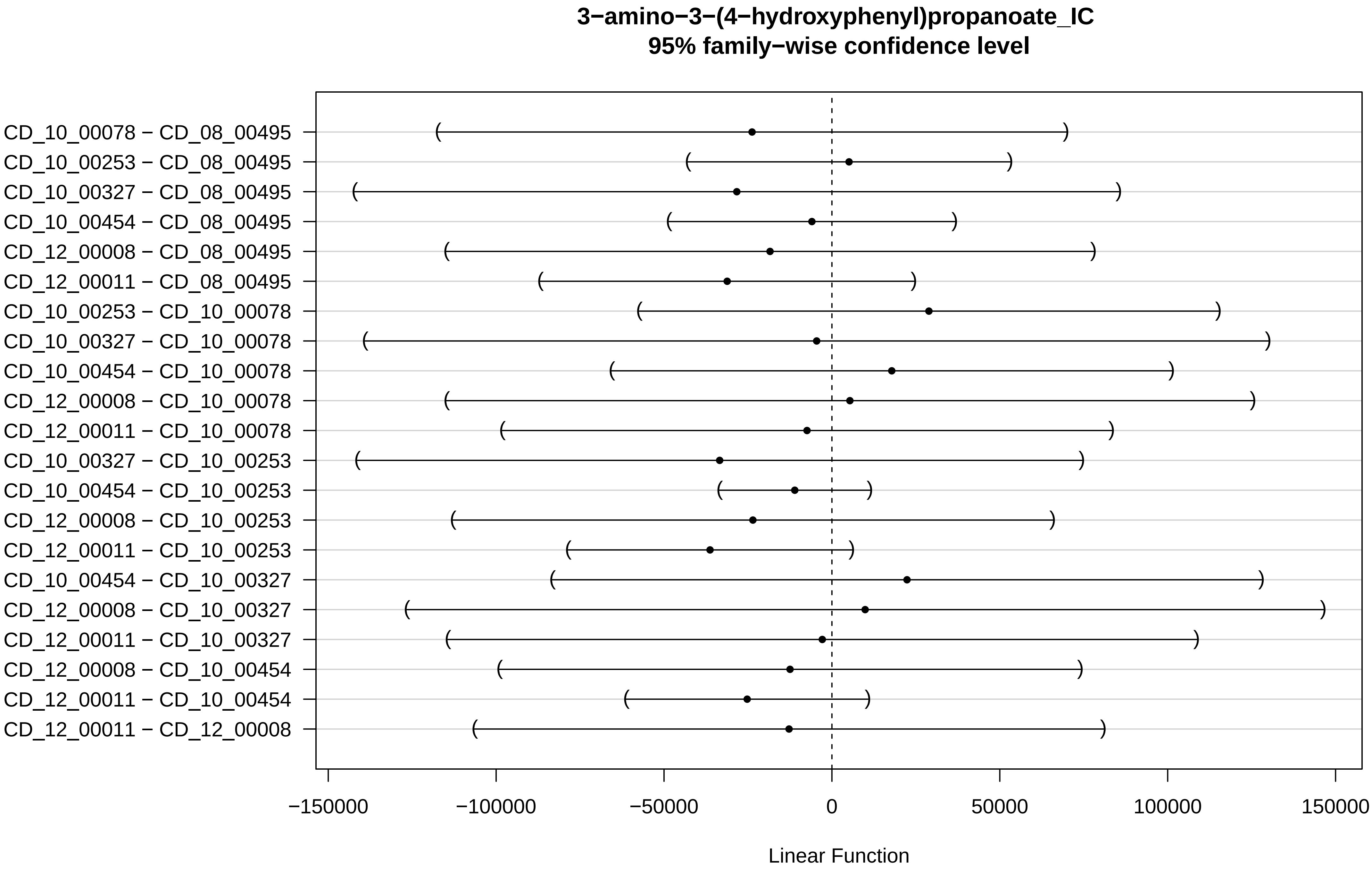
3−amino−3−(4−hydroxyphenyl)propanoate_IC
95% family−wise confidence level
CD_10_00078 − CD_08_00495
CD_10_00253 − CD_08_00495
CD_10_00327 − CD_08_00495
CD_10_00454 − CD_08_00495
CD_12_00008 − CD_08_00495
CD_12_00011 − CD_08_00495
CD_10_00253 − CD_10_00078
CD_10_00327 − CD_10_00078
CD_10_00454 − CD_10_00078
CD_12_00008 − CD_10_00078
CD_12_00011 − CD_10_00078
CD_10_00327 − CD_10_00253
CD_10_00454 − CD_10_00253
CD_12_00008 − CD_10_00253
CD_12_00011 − CD_10_00253
CD_10_00454 − CD_10_00327
CD_12_00008 − CD_10_00327
CD_12_00011 − CD_10_00327
CD_12_00008 − CD_10_00454
CD_12_00011 − CD_10_00454
CD_12_00011 − CD_12_00008
−150000
−100000
−50000
0
50000
100000
150000
Linear Function

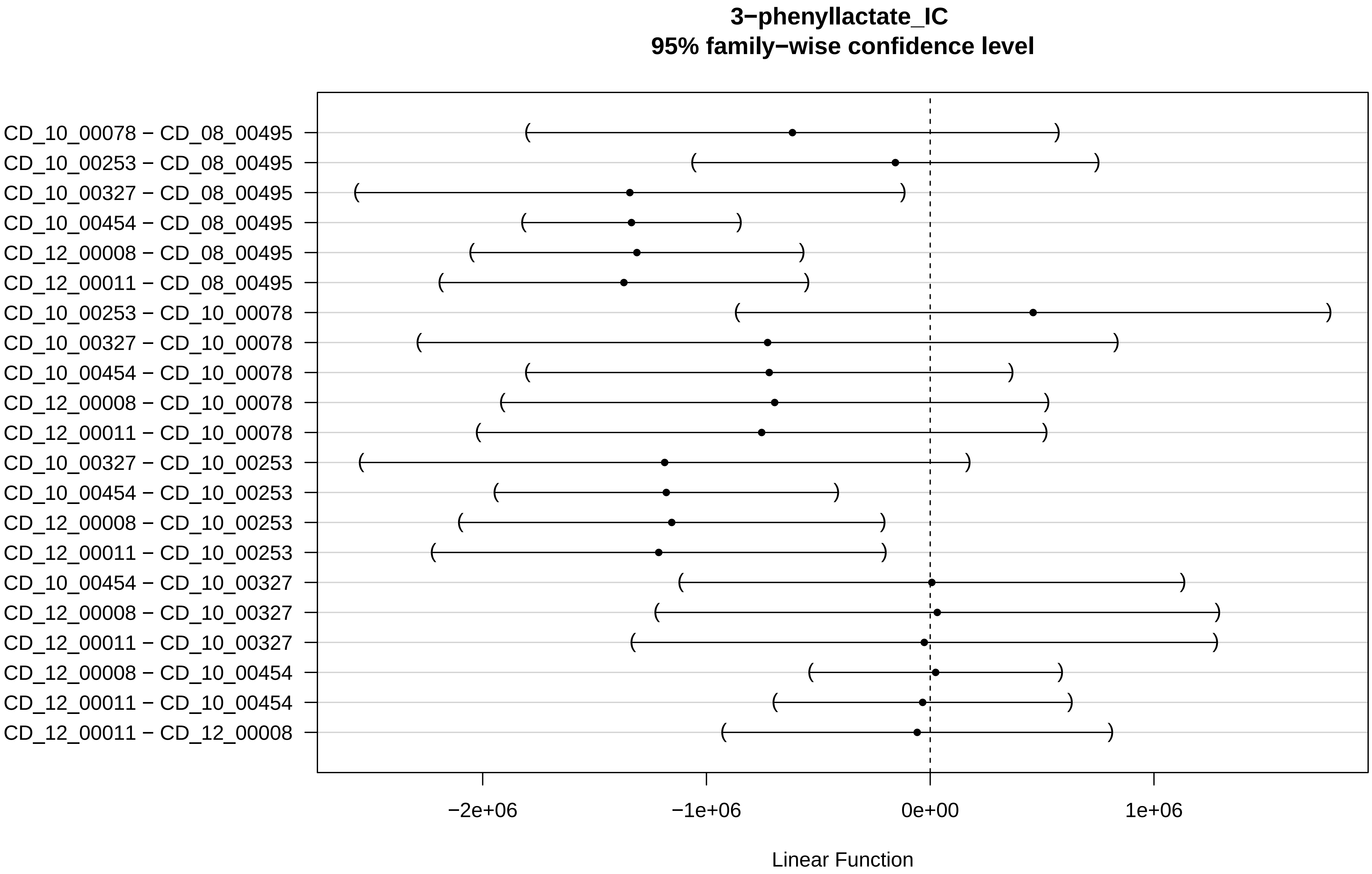
3−phenyllactate_IC
95% family−wise confidence level
CD_10_00078 − CD_08_00495
CD_10_00253 − CD_08_00495
CD_10_00327 − CD_08_00495
CD_10_00454 − CD_08_00495
CD_12_00008 − CD_08_00495
CD_12_00011 − CD_08_00495
CD_10_00253 − CD_10_00078
CD_10_00327 − CD_10_00078
CD_10_00454 − CD_10_00078
CD_12_00008 − CD_10_00078
CD_12_00011 − CD_10_00078
CD_10_00327 − CD_10_00253
CD_10_00454 − CD_10_00253
CD_12_00008 − CD_10_00253
CD_12_00011 − CD_10_00253
CD_10_00454 − CD_10_00327
CD_12_00008 − CD_10_00327
CD_12_00011 − CD_10_00327
CD_12_00008 − CD_10_00454
CD_12_00011 − CD_10_00454
CD_12_00011 − CD_12_00008
−2e+06
−1e+06
0e+00
1e+06
Linear Function

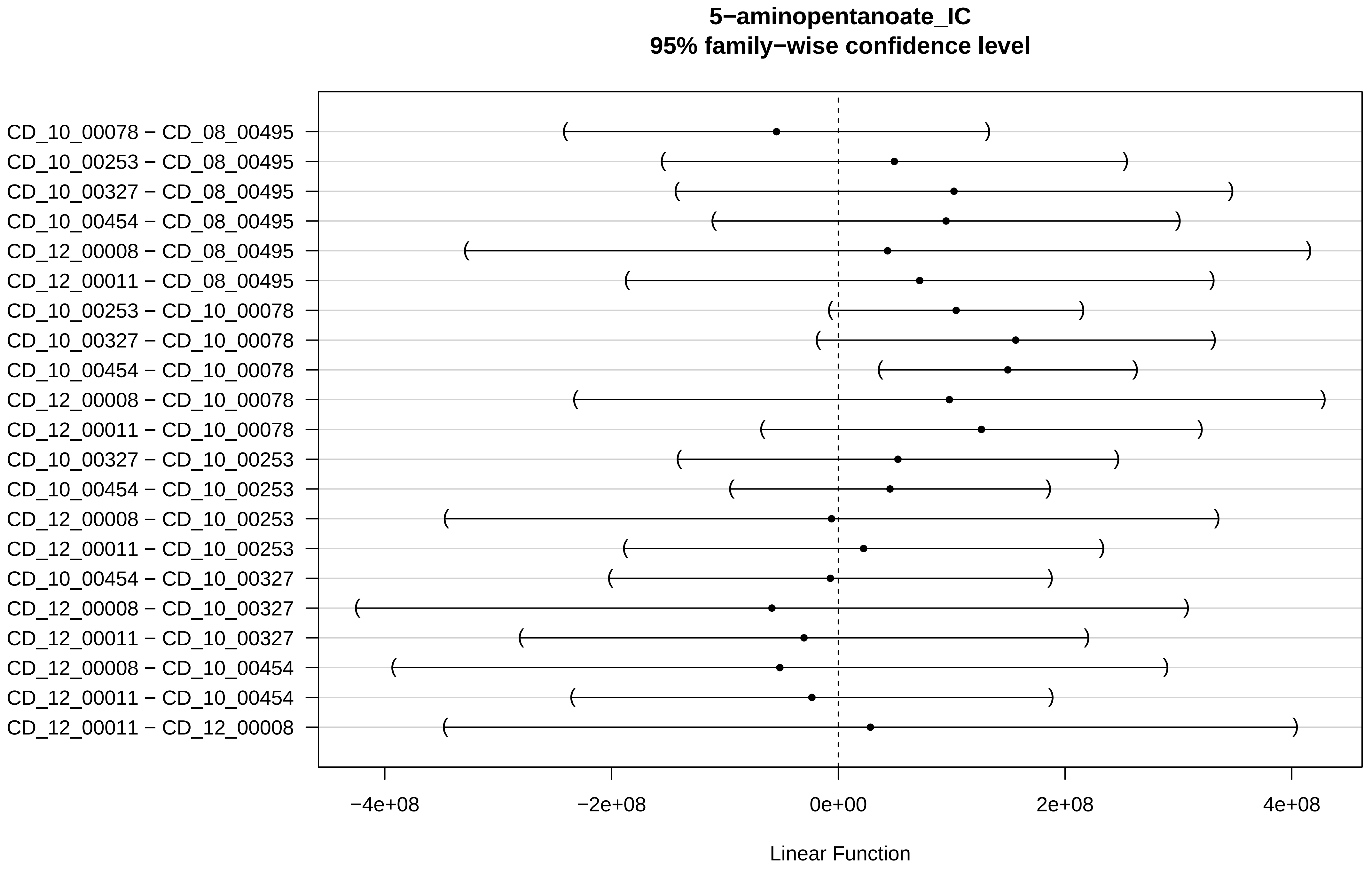
5−aminopentanoate_IC
95% family−wise confidence level
CD_10_00078 − CD_08_00495
CD_10_00253 − CD_08_00495
CD_10_00327 − CD_08_00495
CD_10_00454 − CD_08_00495
CD_12_00008 − CD_08_00495
CD_12_00011 − CD_08_00495
CD_10_00253 − CD_10_00078
CD_10_00327 − CD_10_00078
CD_10_00454 − CD_10_00078
CD_12_00008 − CD_10_00078
CD_12_00011 − CD_10_00078
CD_10_00327 − CD_10_00253
CD_10_00454 − CD_10_00253
CD_12_00008 − CD_10_00253
CD_12_00011 − CD_10_00253
CD_10_00454 − CD_10_00327
CD_12_00008 − CD_10_00327
CD_12_00011 − CD_10_00327
CD_12_00008 − CD_10_00454
CD_12_00011 − CD_10_00454
CD_12_00011 − CD_12_00008
−4e+08
−2e+08
0e+00
2e+08
4e+08
Linear Function

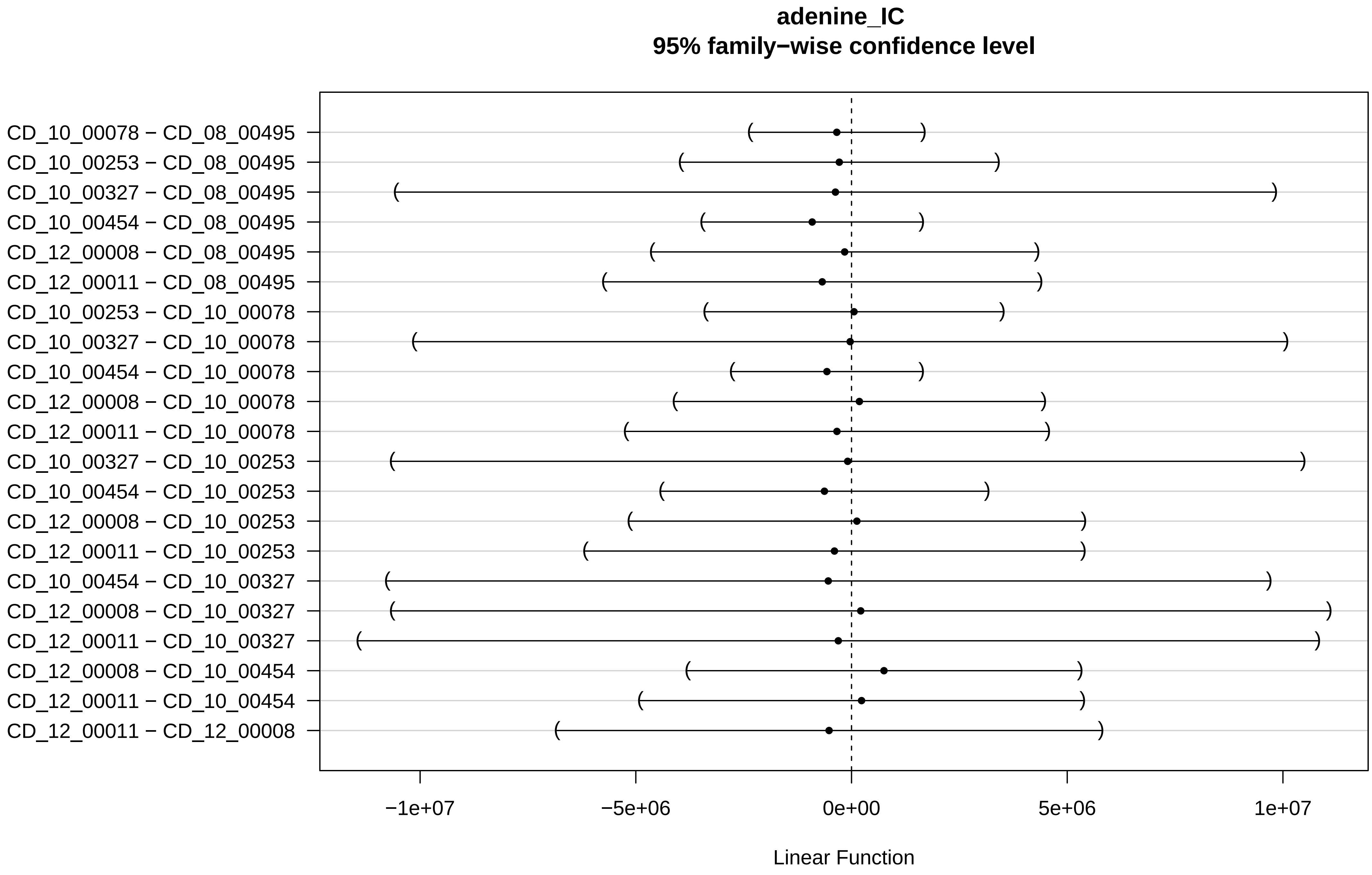
adenine_IC
95% family−wise confidence level
CD_10_00078 − CD_08_00495
CD_10_00253 − CD_08_00495
CD_10_00327 − CD_08_00495
CD_10_00454 − CD_08_00495
CD_12_00008 − CD_08_00495
CD_12_00011 − CD_08_00495
CD_10_00253 − CD_10_00078
CD_10_00327 − CD_10_00078
CD_10_00454 − CD_10_00078
CD_12_00008 − CD_10_00078
CD_12_00011 − CD_10_00078
CD_10_00327 − CD_10_00253
CD_10_00454 − CD_10_00253
CD_12_00008 − CD_10_00253
CD_12_00011 − CD_10_00253
CD_10_00454 − CD_10_00327
CD_12_00008 − CD_10_00327
CD_12_00011 − CD_10_00327
CD_12_00008 − CD_10_00454
CD_12_00011 − CD_10_00454
CD_12_00011 − CD_12_00008
−1e+07
−5e+06
0e+00
5e+06
1e+07
Linear Function

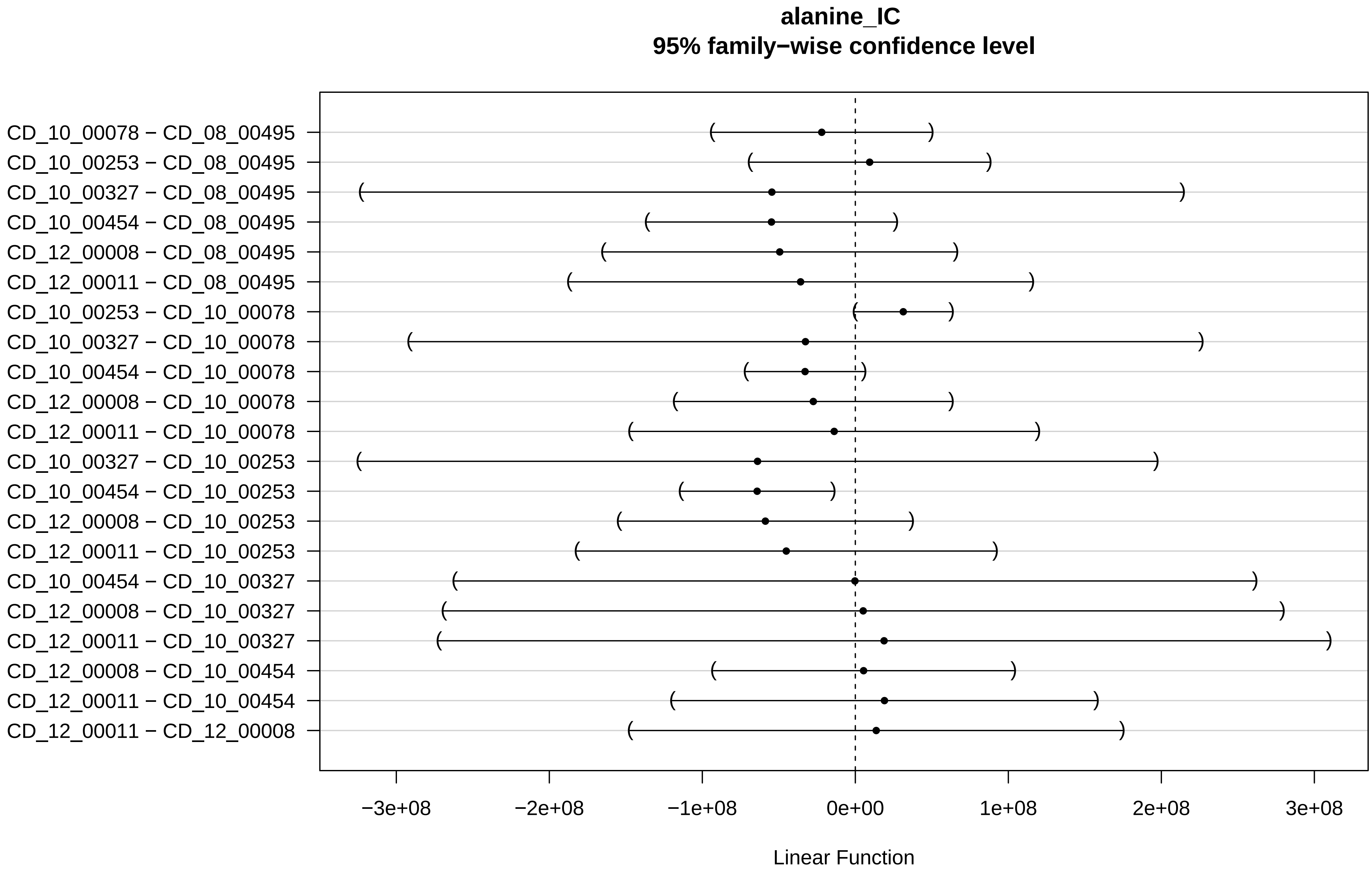
alanine_IC
95% family−wise confidence level
CD_10_00078 − CD_08_00495
CD_10_00253 − CD_08_00495
CD_10_00327 − CD_08_00495
CD_10_00454 − CD_08_00495
CD_12_00008 − CD_08_00495
CD_12_00011 − CD_08_00495
CD_10_00253 − CD_10_00078
CD_10_00327 − CD_10_00078
CD_10_00454 − CD_10_00078
CD_12_00008 − CD_10_00078
CD_12_00011 − CD_10_00078
CD_10_00327 − CD_10_00253
CD_10_00454 − CD_10_00253
CD_12_00008 − CD_10_00253
CD_12_00011 − CD_10_00253
CD_10_00454 − CD_10_00327
CD_12_00008 − CD_10_00327
CD_12_00011 − CD_10_00327
CD_12_00008 − CD_10_00454
CD_12_00011 − CD_10_00454
CD_12_00011 − CD_12_00008
−3e+08
−2e+08
−1e+08
0e+00
1e+08
2e+08
3e+08
Linear Function

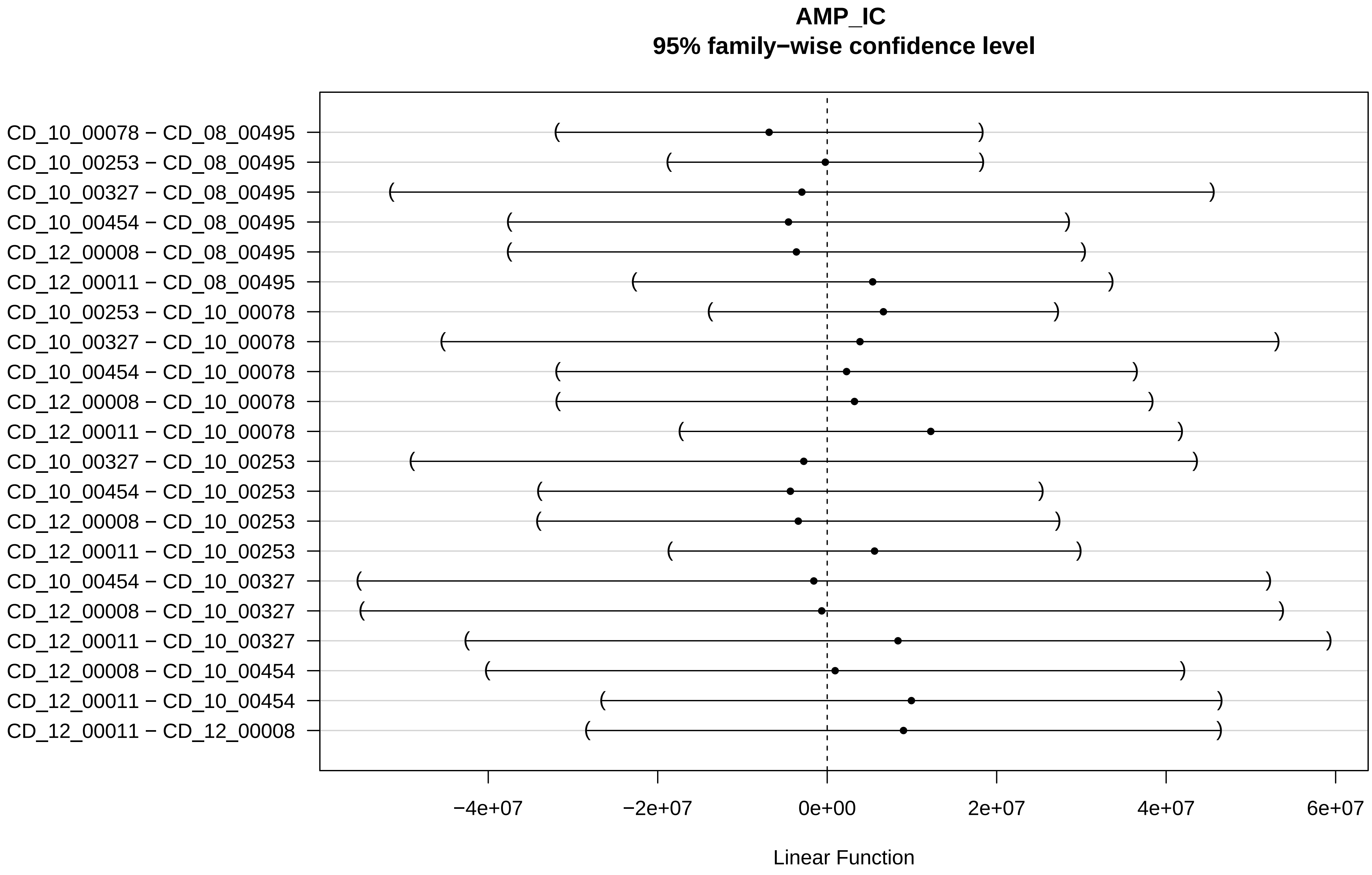
AMP_IC
95% family−wise confidence level
CD_10_00078 − CD_08_00495
CD_10_00253 − CD_08_00495
CD_10_00327 − CD_08_00495
CD_10_00454 − CD_08_00495
CD_12_00008 − CD_08_00495
CD_12_00011 − CD_08_00495
CD_10_00253 − CD_10_00078
CD_10_00327 − CD_10_00078
CD_10_00454 − CD_10_00078
CD_12_00008 − CD_10_00078
CD_12_00011 − CD_10_00078
CD_10_00327 − CD_10_00253
CD_10_00454 − CD_10_00253
CD_12_00008 − CD_10_00253
CD_12_00011 − CD_10_00253
CD_10_00454 − CD_10_00327
CD_12_00008 − CD_10_00327
CD_12_00011 − CD_10_00327
CD_12_00008 − CD_10_00454
CD_12_00011 − CD_10_00454
CD_12_00011 − CD_12_00008
−4e+07
−2e+07
0e+00
2e+07
4e+07
6e+07
Linear Function

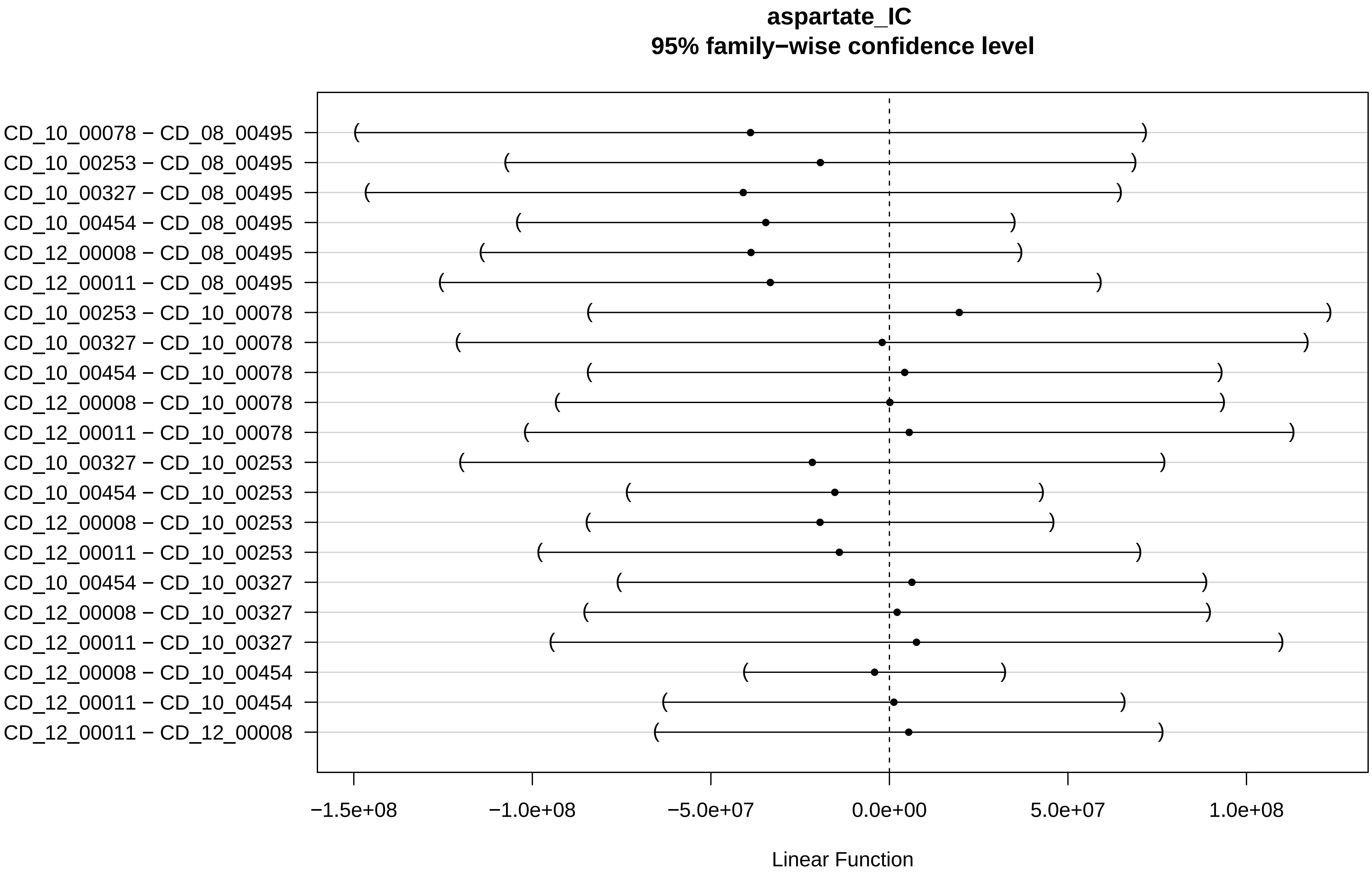
aspartate_IC
95% family−wise confidence level
CD_10_00078 − CD_08_00495
CD_10_00253 − CD_08_00495
CD_10_00327 − CD_08_00495
CD_10_00454 − CD_08_00495
CD_12_00008 − CD_08_00495
CD_12_00011 − CD_08_00495
CD_10_00253 − CD_10_00078
CD_10_00327 − CD_10_00078
CD_10_00454 − CD_10_00078
CD_12_00008 − CD_10_00078
CD_12_00011 − CD_10_00078
CD_10_00327 − CD_10_00253
CD_10_00454 − CD_10_00253
CD_12_00008 − CD_10_00253
CD_12_00011 − CD_10_00253
CD_10_00454 − CD_10_00327
CD_12_00008 − CD_10_00327
CD_12_00011 − CD_10_00327
CD_12_00008 − CD_10_00454
CD_12_00011 − CD_10_00454
CD_12_00011 − CD_12_00008
−1.5e+08
−1.0e+08
−5.0e+07
0.0e+00
5.0e+07
1.0e+08
Linear Function

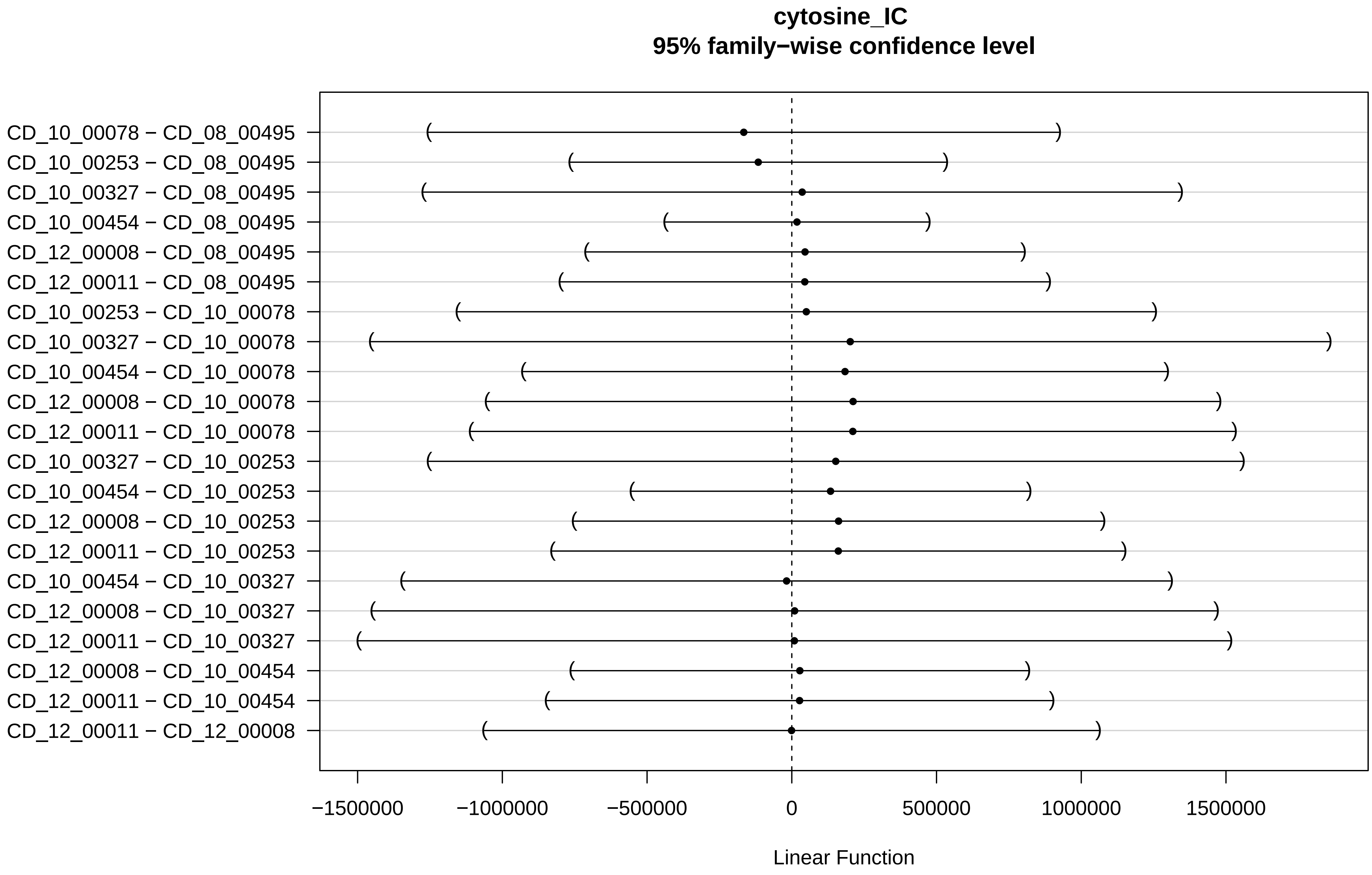
cytosine_IC
95% family−wise confidence level
CD_10_00078 − CD_08_00495
CD_10_00253 − CD_08_00495
CD_10_00327 − CD_08_00495
CD_10_00454 − CD_08_00495
CD_12_00008 − CD_08_00495
CD_12_00011 − CD_08_00495
CD_10_00253 − CD_10_00078
CD_10_00327 − CD_10_00078
CD_10_00454 − CD_10_00078
CD_12_00008 − CD_10_00078
CD_12_00011 − CD_10_00078
CD_10_00327 − CD_10_00253
CD_10_00454 − CD_10_00253
CD_12_00008 − CD_10_00253
CD_12_00011 − CD_10_00253
CD_10_00454 − CD_10_00327
CD_12_00008 − CD_10_00327
CD_12_00011 − CD_10_00327
CD_12_00008 − CD_10_00454
CD_12_00011 − CD_10_00454
CD_12_00011 − CD_12_00008
−1500000
−1000000
−500000
0
500000
1000000
1500000
Linear Function

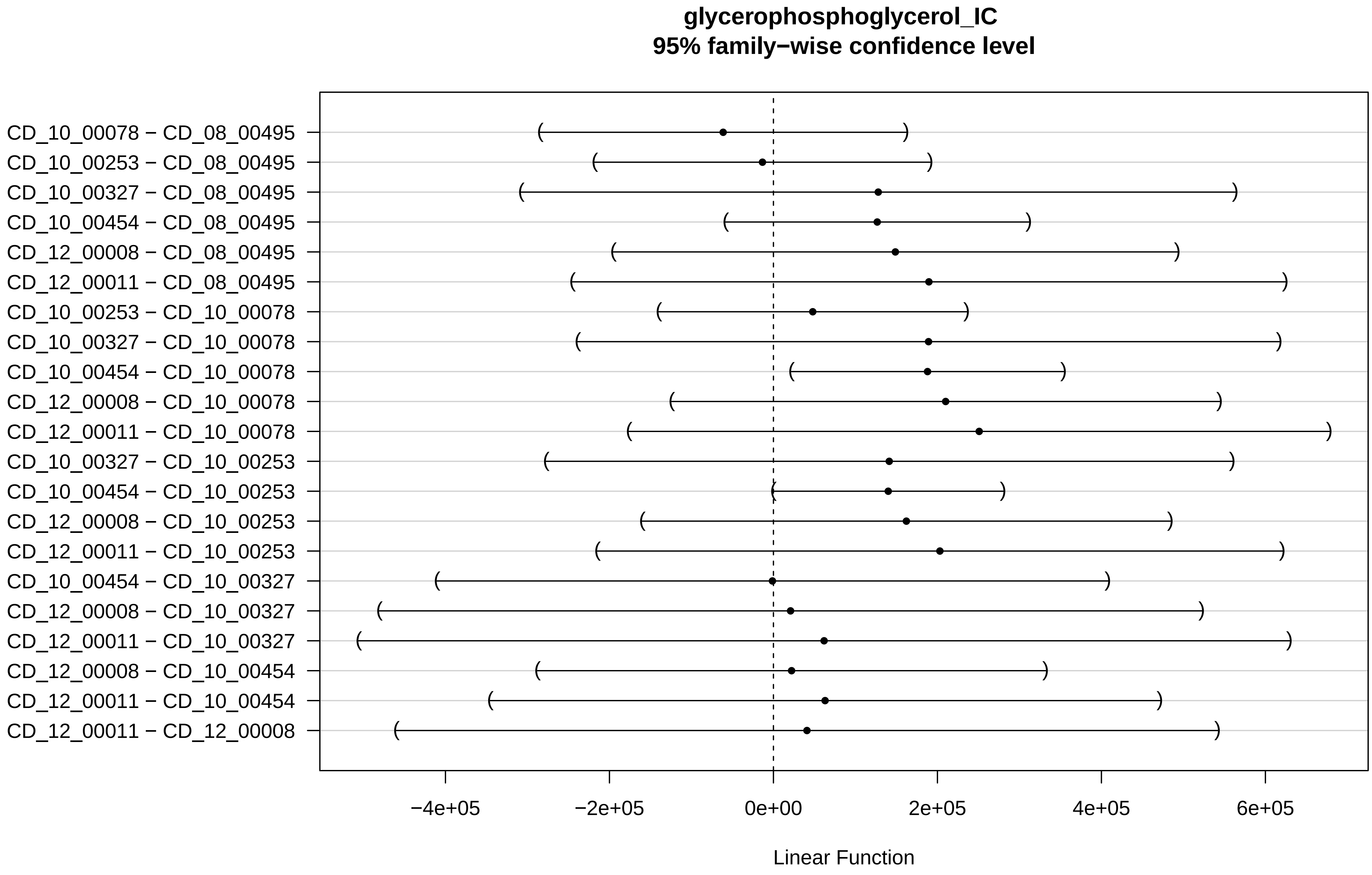

glycerophosphoglycerol_IC
95% family−wise confidence level
CD_10_00078 − CD_08_00495
CD_10_00253 − CD_08_00495
CD_10_00327 − CD_08_00495
CD_10_00454 − CD_08_00495
CD_12_00008 − CD_08_00495
CD_12_00011 − CD_08_00495
CD_10_00253 − CD_10_00078
CD_10_00327 − CD_10_00078
CD_10_00454 − CD_10_00078
CD_12_00008 − CD_10_00078
CD_12_00011 − CD_10_00078
CD_10_00327 − CD_10_00253
CD_10_00454 − CD_10_00253
CD_12_00008 − CD_10_00253
CD_12_00011 − CD_10_00253
CD_10_00454 − CD_10_00327
CD_12_00008 − CD_10_00327
CD_12_00011 − CD_10_00327
CD_12_00008 − CD_10_00454
CD_12_00011 − CD_10_00454
CD_12_00011 − CD_12_00008
−4e+05
−2e+05
0e+00
2e+05
4e+05
6e+05
Linear Function

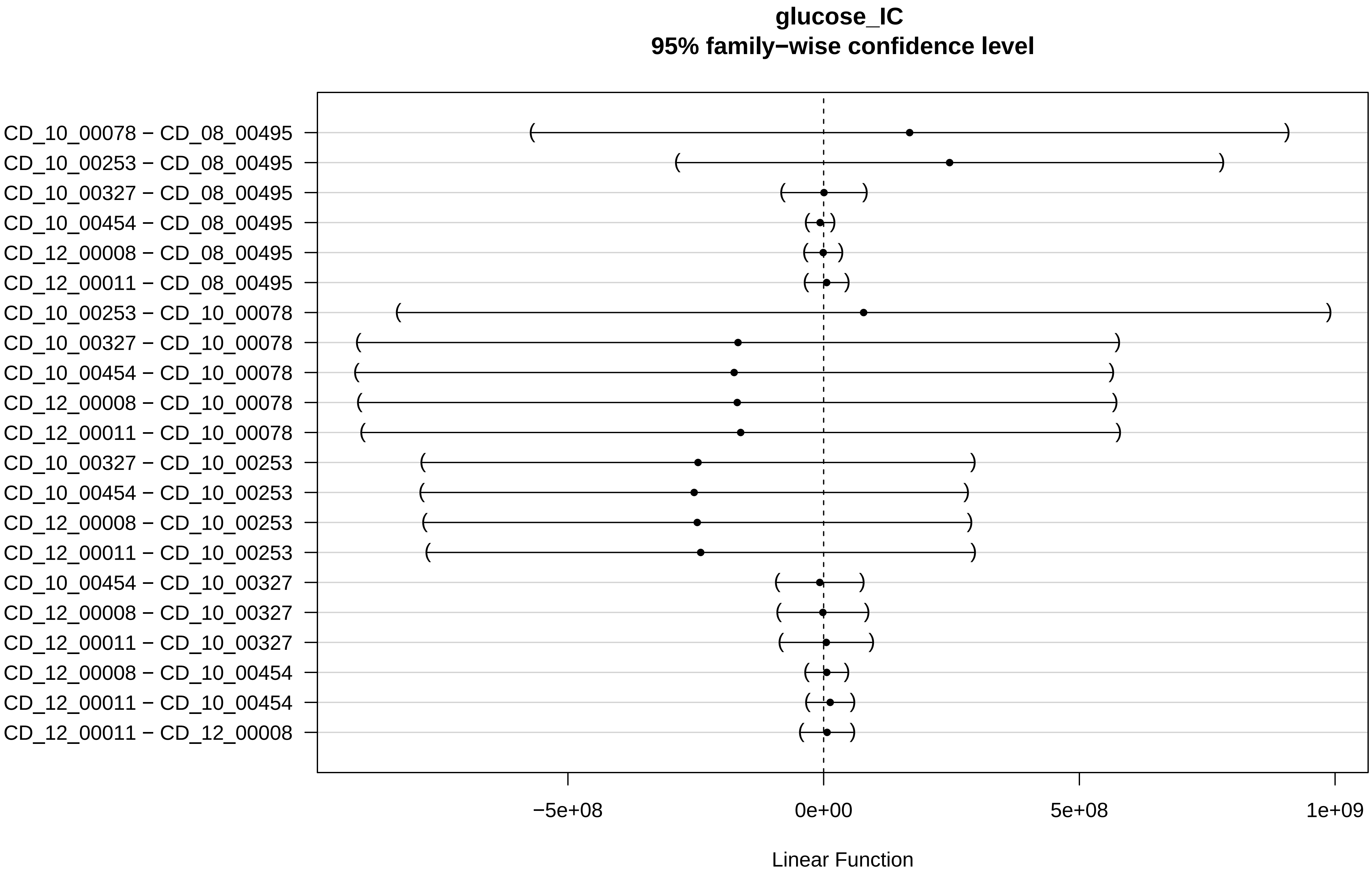
glucose_IC
95% family−wise confidence level
CD_10_00078 − CD_08_00495
CD_10_00253 − CD_08_00495
CD_10_00327 − CD_08_00495
CD_10_00454 − CD_08_00495
CD_12_00008 − CD_08_00495
CD_12_00011 − CD_08_00495
CD_10_00253 − CD_10_00078
CD_10_00327 − CD_10_00078
CD_10_00454 − CD_10_00078
CD_12_00008 − CD_10_00078
CD_12_00011 − CD_10_00078
CD_10_00327 − CD_10_00253
CD_10_00454 − CD_10_00253
CD_12_00008 − CD_10_00253
CD_12_00011 − CD_10_00253
CD_10_00454 − CD_10_00327
CD_12_00008 − CD_10_00327
CD_12_00011 − CD_10_00327
CD_12_00008 − CD_10_00454
CD_12_00011 − CD_10_00454
CD_12_00011 − CD_12_00008
−5e+08
0e+00
5e+08
1e+09
Linear Function

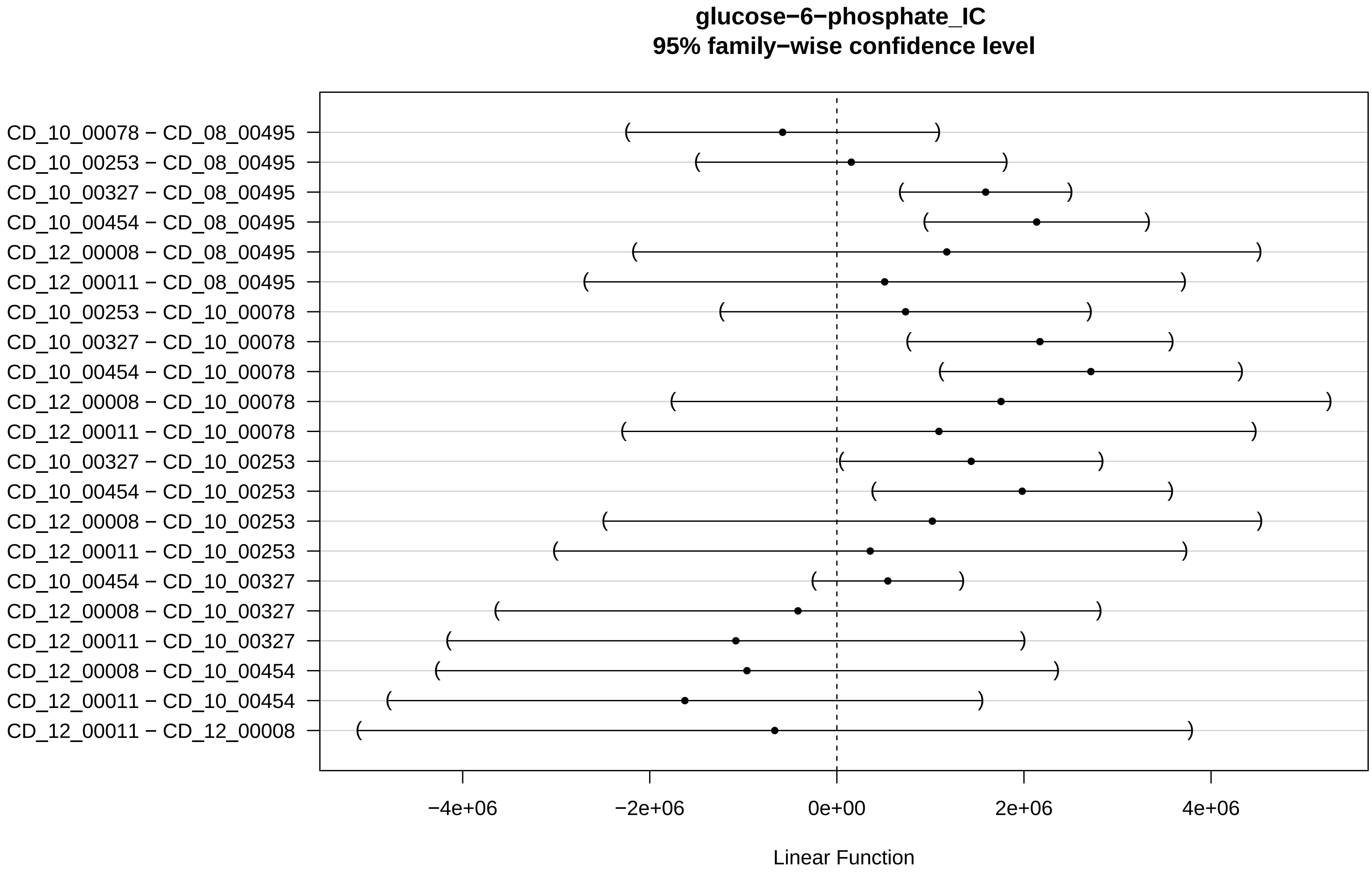

glucose−6−phosphate_IC
95% family−wise confidence level
CD_10_00078 − CD_08_00495
CD_10_00253 − CD_08_00495
CD_10_00327 − CD_08_00495
CD_10_00454 − CD_08_00495
CD_12_00008 − CD_08_00495
CD_12_00011 − CD_08_00495
CD_10_00253 − CD_10_00078
CD_10_00327 − CD_10_00078
CD_10_00454 − CD_10_00078
CD_12_00008 − CD_10_00078
CD_12_00011 − CD_10_00078
CD_10_00327 − CD_10_00253
CD_10_00454 − CD_10_00253
CD_12_00008 − CD_10_00253
CD_12_00011 − CD_10_00253
CD_10_00454 − CD_10_00327
CD_12_00008 − CD_10_00327
CD_12_00011 − CD_10_00327
CD_12_00008 − CD_10_00454
CD_12_00011 − CD_10_00454
CD_12_00011 − CD_12_00008
−4e+06
−2e+06
0e+00
2e+06
4e+06
Linear Function

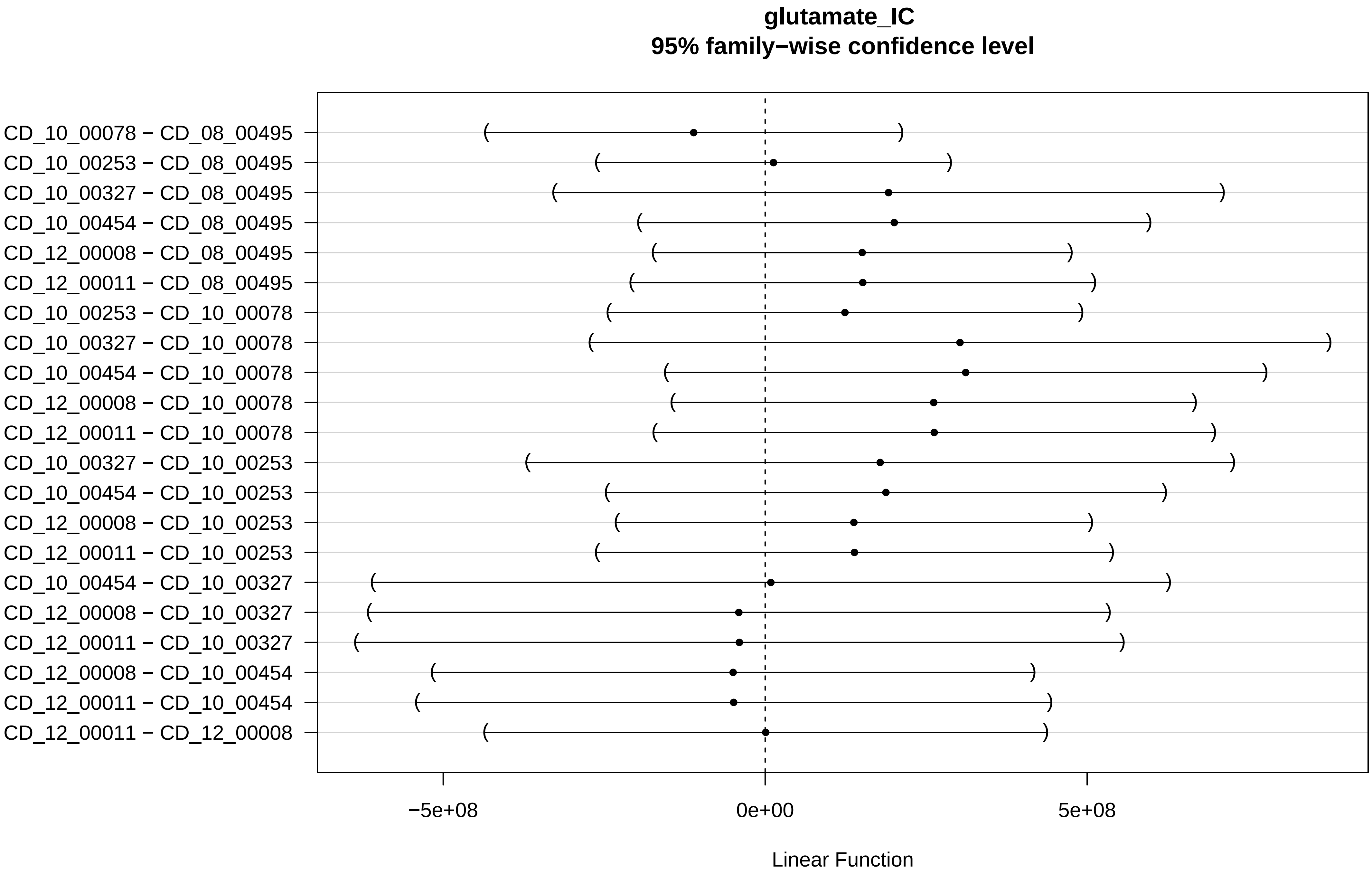
glutamate_IC
95% family−wise confidence level
CD_10_00078 − CD_08_00495
CD_10_00253 − CD_08_00495
CD_10_00327 − CD_08_00495
CD_10_00454 − CD_08_00495
CD_12_00008 − CD_08_00495
CD_12_00011 − CD_08_00495
CD_10_00253 − CD_10_00078
CD_10_00327 − CD_10_00078
CD_10_00454 − CD_10_00078
CD_12_00008 − CD_10_00078
CD_12_00011 − CD_10_00078
CD_10_00327 − CD_10_00253
CD_10_00454 − CD_10_00253
CD_12_00008 − CD_10_00253
CD_12_00011 − CD_10_00253
CD_10_00454 − CD_10_00327
CD_12_00008 − CD_10_00327
CD_12_00011 − CD_10_00327
CD_12_00008 − CD_10_00454
CD_12_00011 − CD_10_00454
CD_12_00011 − CD_12_00008
−5e+08
0e+00
5e+08
Linear Function

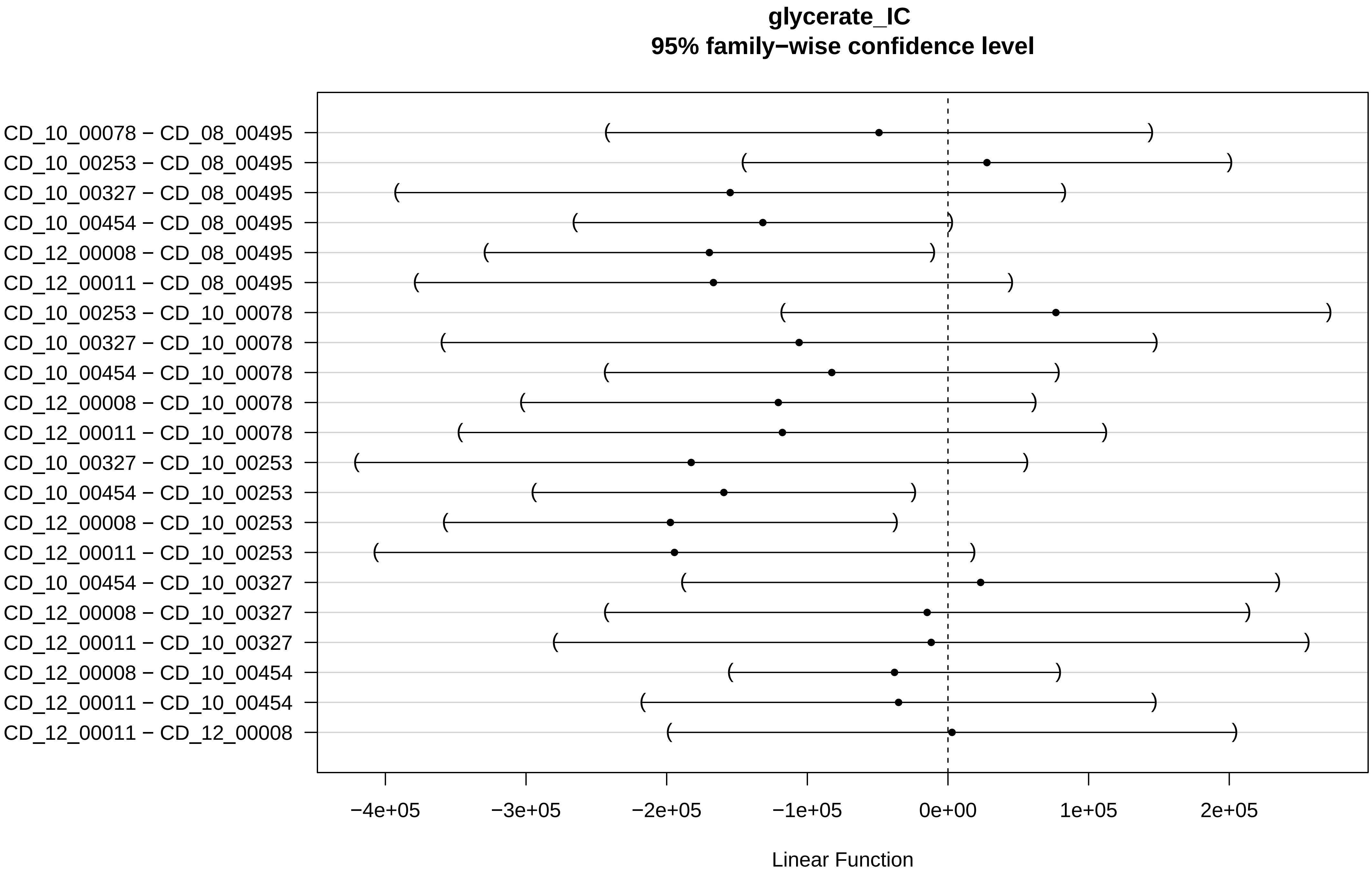
glycerate_IC
95% family−wise confidence level
CD_10_00078 − CD_08_00495
CD_10_00253 − CD_08_00495
CD_10_00327 − CD_08_00495
CD_10_00454 − CD_08_00495
CD_12_00008 − CD_08_00495
CD_12_00011 − CD_08_00495
CD_10_00253 − CD_10_00078
CD_10_00327 − CD_10_00078
CD_10_00454 − CD_10_00078
CD_12_00008 − CD_10_00078
CD_12_00011 − CD_10_00078
CD_10_00327 − CD_10_00253
CD_10_00454 − CD_10_00253
CD_12_00008 − CD_10_00253
CD_12_00011 − CD_10_00253
CD_10_00454 − CD_10_00327
CD_12_00008 − CD_10_00327
CD_12_00011 − CD_10_00327
CD_12_00008 − CD_10_00454
CD_12_00011 − CD_10_00454
CD_12_00011 − CD_12_00008
−4e+05
−3e+05
−2e+05
−1e+05
0e+00
1e+05
2e+05
Linear Function

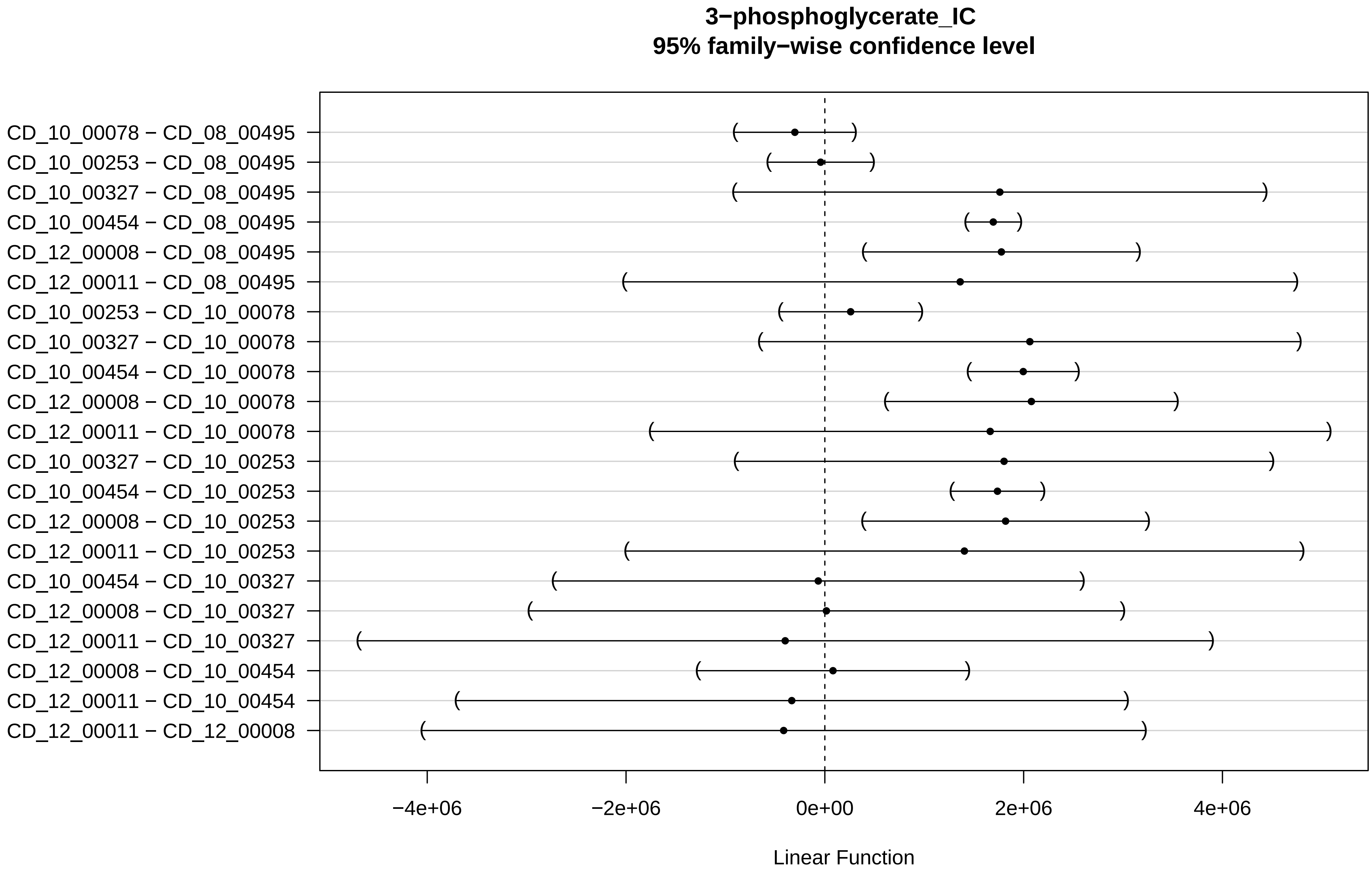
3−phosphoglycerate_IC
95% family−wise confidence level
CD_10_00078 − CD_08_00495
CD_10_00253 − CD_08_00495
CD_10_00327 − CD_08_00495
CD_10_00454 − CD_08_00495
CD_12_00008 − CD_08_00495
CD_12_00011 − CD_08_00495
CD_10_00253 − CD_10_00078
CD_10_00327 − CD_10_00078
CD_10_00454 − CD_10_00078
CD_12_00008 − CD_10_00078
CD_12_00011 − CD_10_00078
CD_10_00327 − CD_10_00253
CD_10_00454 − CD_10_00253
CD_12_00008 − CD_10_00253
CD_12_00011 − CD_10_00253
CD_10_00454 − CD_10_00327
CD_12_00008 − CD_10_00327
CD_12_00011 − CD_10_00327
CD_12_00008 − CD_10_00454
CD_12_00011 − CD_10_00454
CD_12_00011 − CD_12_00008
−4e+06
−2e+06
0e+00
2e+06
4e+06
Linear Function

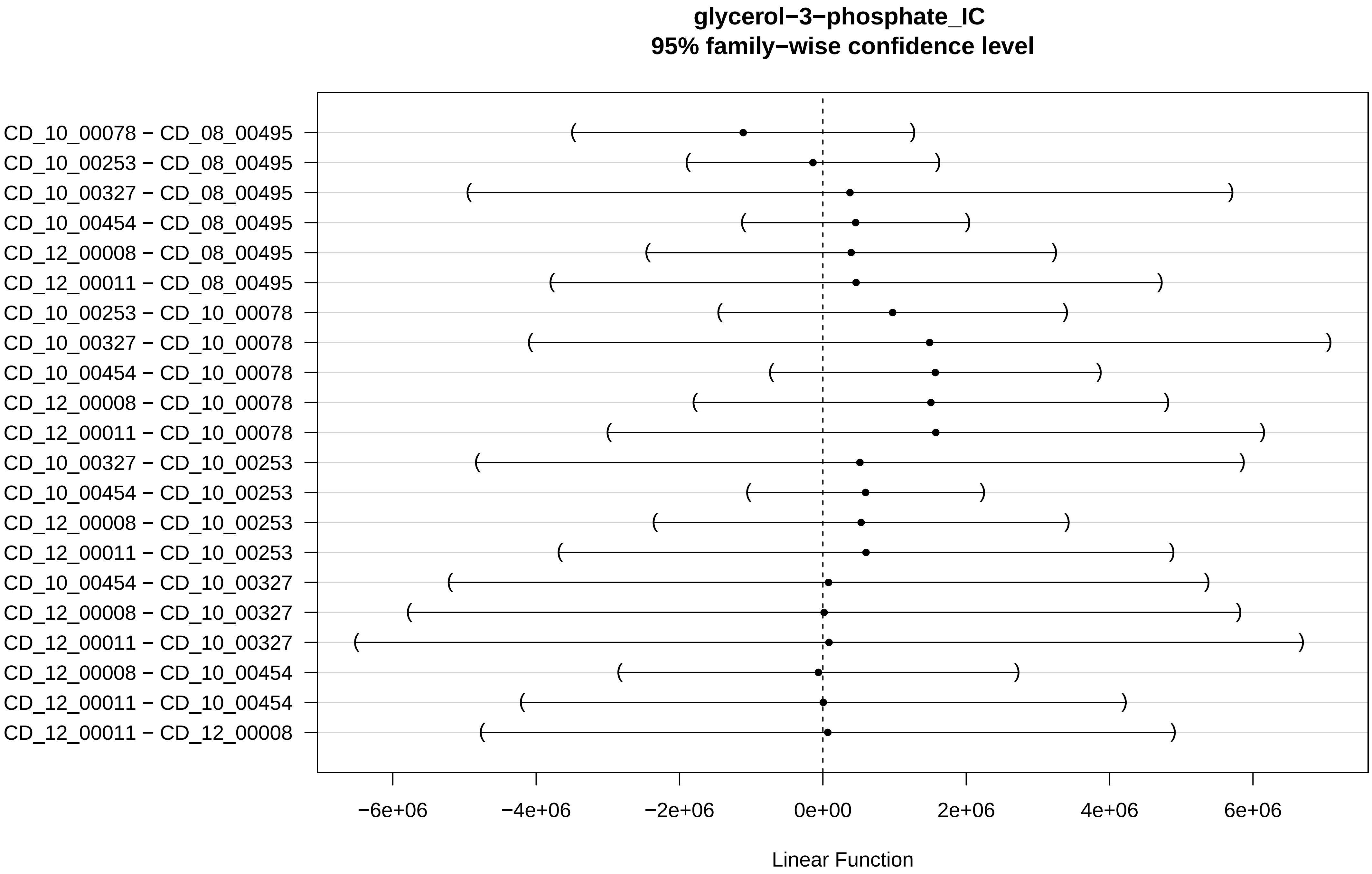
glycerol−3−phosphate_IC
95% family−wise confidence level
CD_10_00078 − CD_08_00495
CD_10_00253 − CD_08_00495
CD_10_00327 − CD_08_00495
CD_10_00454 − CD_08_00495
CD_12_00008 − CD_08_00495
CD_12_00011 − CD_08_00495
CD_10_00253 − CD_10_00078
CD_10_00327 − CD_10_00078
CD_10_00454 − CD_10_00078
CD_12_00008 − CD_10_00078
CD_12_00011 − CD_10_00078
CD_10_00327 − CD_10_00253
CD_10_00454 − CD_10_00253
CD_12_00008 − CD_10_00253
CD_12_00011 − CD_10_00253
CD_10_00454 − CD_10_00327
CD_12_00008 − CD_10_00327
CD_12_00011 − CD_10_00327
CD_12_00008 − CD_10_00454
CD_12_00011 − CD_10_00454
CD_12_00011 − CD_12_00008
−6e+06
−4e+06
−2e+06
0e+00
2e+06
4e+06
6e+06
Linear Function

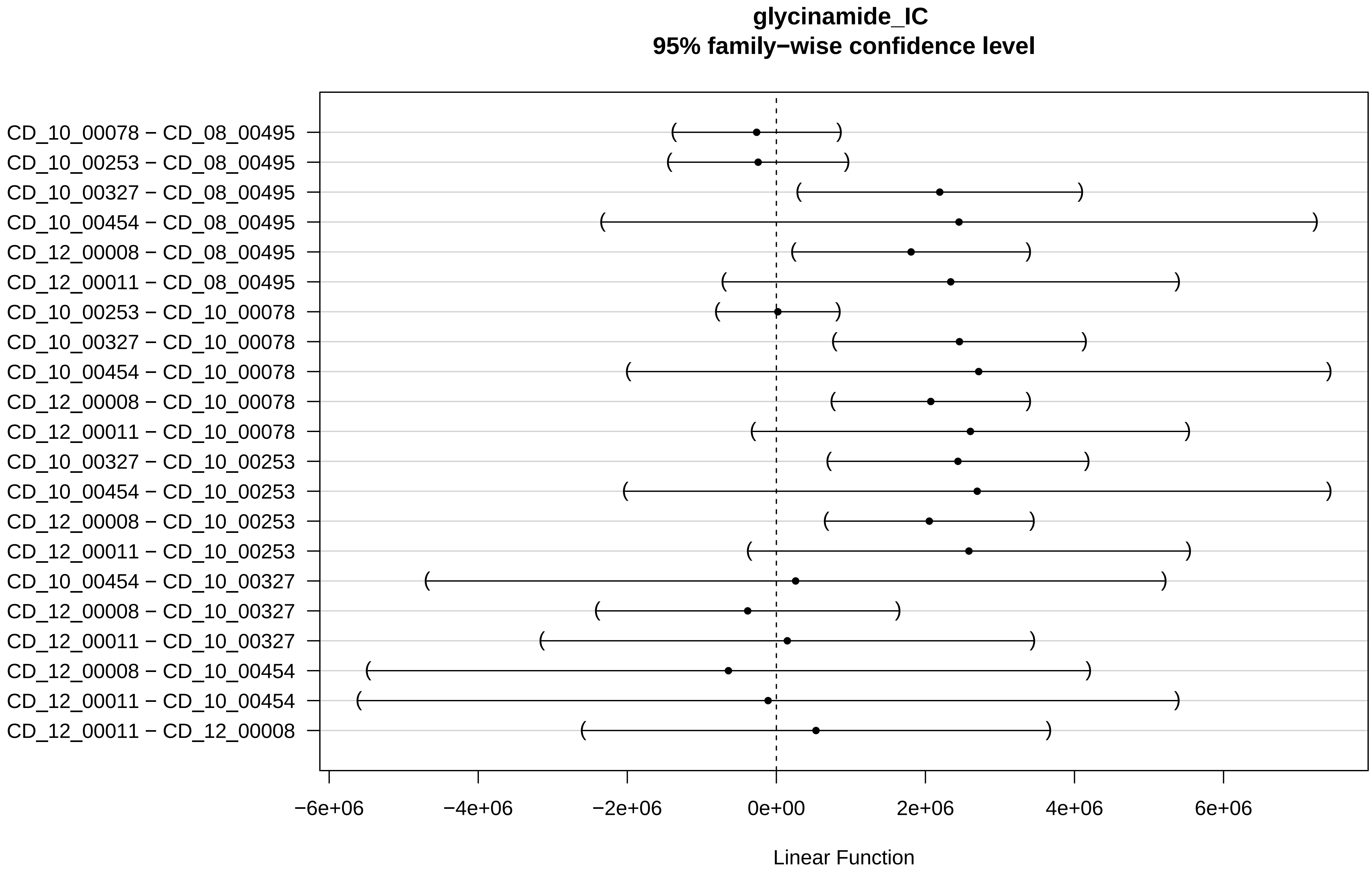

glycinamide_IC
95% family−wise confidence level
CD_10_00078 − CD_08_00495
CD_10_00253 − CD_08_00495
CD_10_00327 − CD_08_00495
CD_10_00454 − CD_08_00495
CD_12_00008 − CD_08_00495
CD_12_00011 − CD_08_00495
CD_10_00253 − CD_10_00078
CD_10_00327 − CD_10_00078
CD_10_00454 − CD_10_00078
CD_12_00008 − CD_10_00078
CD_12_00011 − CD_10_00078
CD_10_00327 − CD_10_00253
CD_10_00454 − CD_10_00253
CD_12_00008 − CD_10_00253
CD_12_00011 − CD_10_00253
CD_10_00454 − CD_10_00327
CD_12_00008 − CD_10_00327
CD_12_00011 − CD_10_00327
CD_12_00008 − CD_10_00454
CD_12_00011 − CD_10_00454
CD_12_00011 − CD_12_00008
−6e+06
−4e+06
−2e+06
0e+00
2e+06
4e+06
6e+06
Linear Function

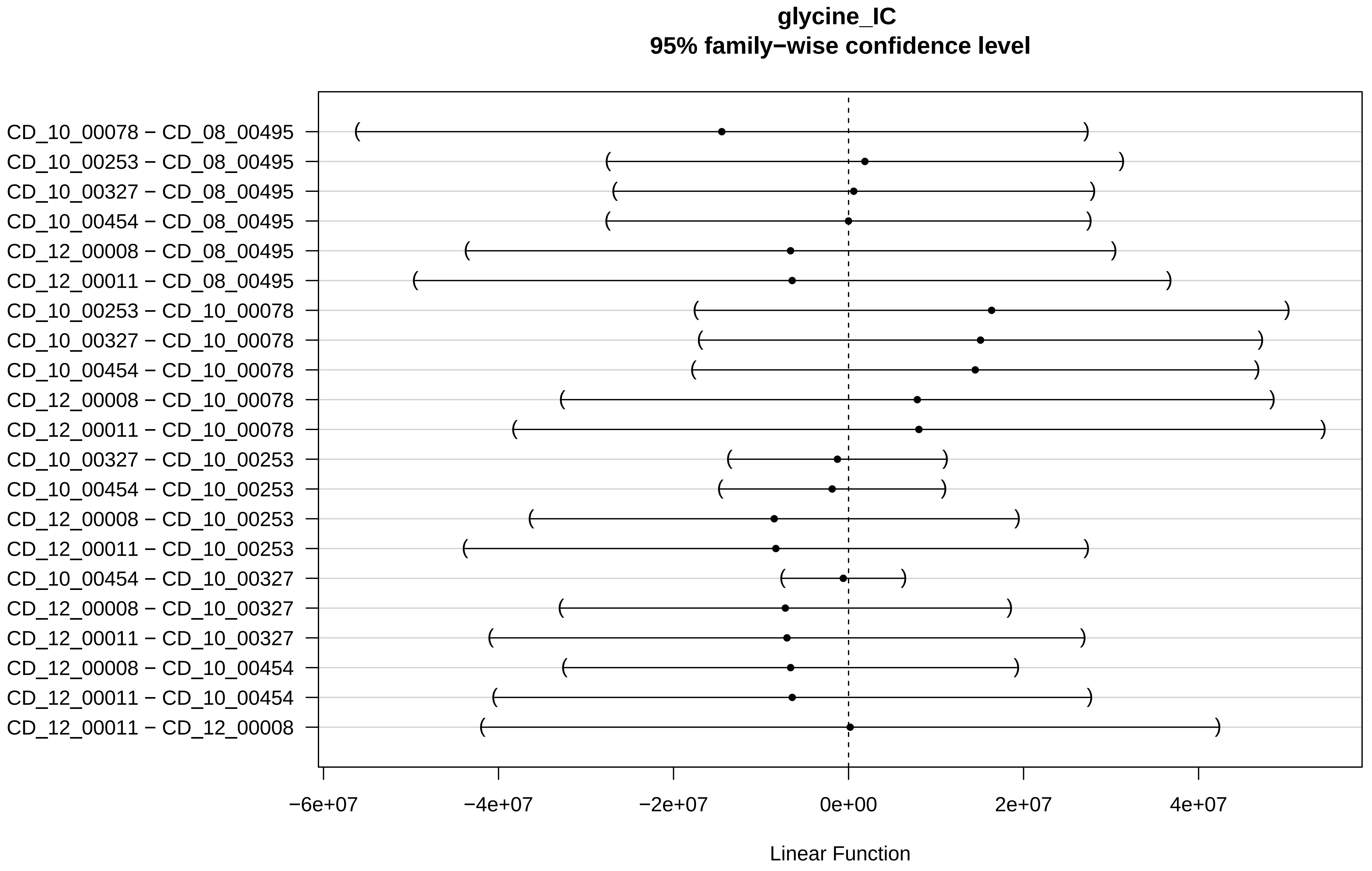
glycine_IC
95% family−wise confidence level
CD_10_00078 − CD_08_00495
CD_10_00253 − CD_08_00495
CD_10_00327 − CD_08_00495
CD_10_00454 − CD_08_00495
CD_12_00008 − CD_08_00495
CD_12_00011 − CD_08_00495
CD_10_00253 − CD_10_00078
CD_10_00327 − CD_10_00078
CD_10_00454 − CD_10_00078
CD_12_00008 − CD_10_00078
CD_12_00011 − CD_10_00078
CD_10_00327 − CD_10_00253
CD_10_00454 − CD_10_00253
CD_12_00008 − CD_10_00253
CD_12_00011 − CD_10_00253
CD_10_00454 − CD_10_00327
CD_12_00008 − CD_10_00327
CD_12_00011 − CD_10_00327
CD_12_00008 − CD_10_00454
CD_12_00011 − CD_10_00454
CD_12_00011 − CD_12_00008
−6e+07
−4e+07
−2e+07
0e+00
2e+07
4e+07
Linear Function

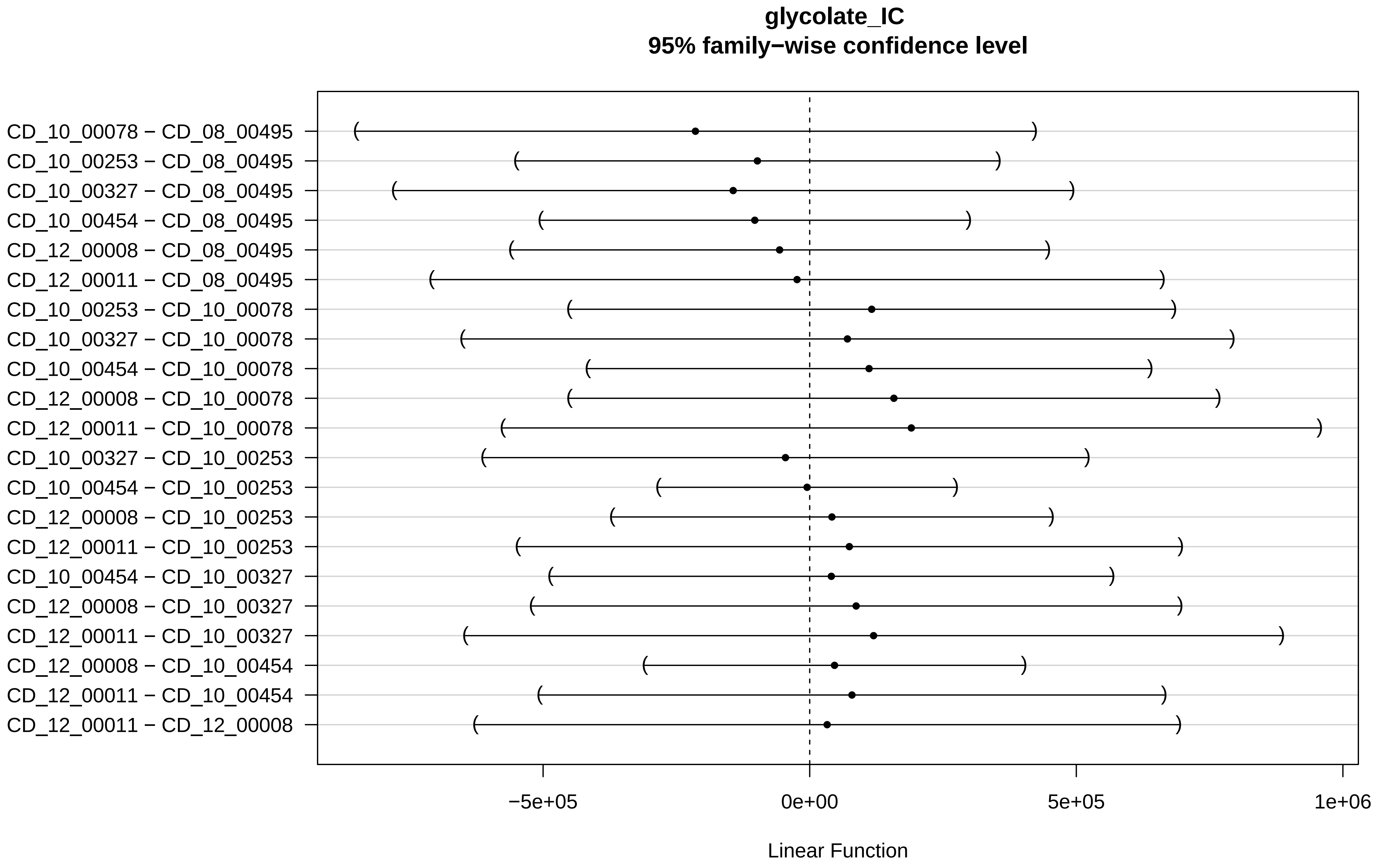
glycolate_IC
95% family−wise confidence level
CD_10_00078 − CD_08_00495
CD_10_00253 − CD_08_00495
CD_10_00327 − CD_08_00495
CD_10_00454 − CD_08_00495
CD_12_00008 − CD_08_00495
CD_12_00011 − CD_08_00495
CD_10_00253 − CD_10_00078
CD_10_00327 − CD_10_00078
CD_10_00454 − CD_10_00078
CD_12_00008 − CD_10_00078
CD_12_00011 − CD_10_00078
CD_10_00327 − CD_10_00253
CD_10_00454 − CD_10_00253
CD_12_00008 − CD_10_00253
CD_12_00011 − CD_10_00253
CD_10_00454 − CD_10_00327
CD_12_00008 − CD_10_00327
CD_12_00011 − CD_10_00327
CD_12_00008 − CD_10_00454
CD_12_00011 − CD_10_00454
CD_12_00011 − CD_12_00008
−5e+05
0e+00
5e+05
1e+06
Linear Function

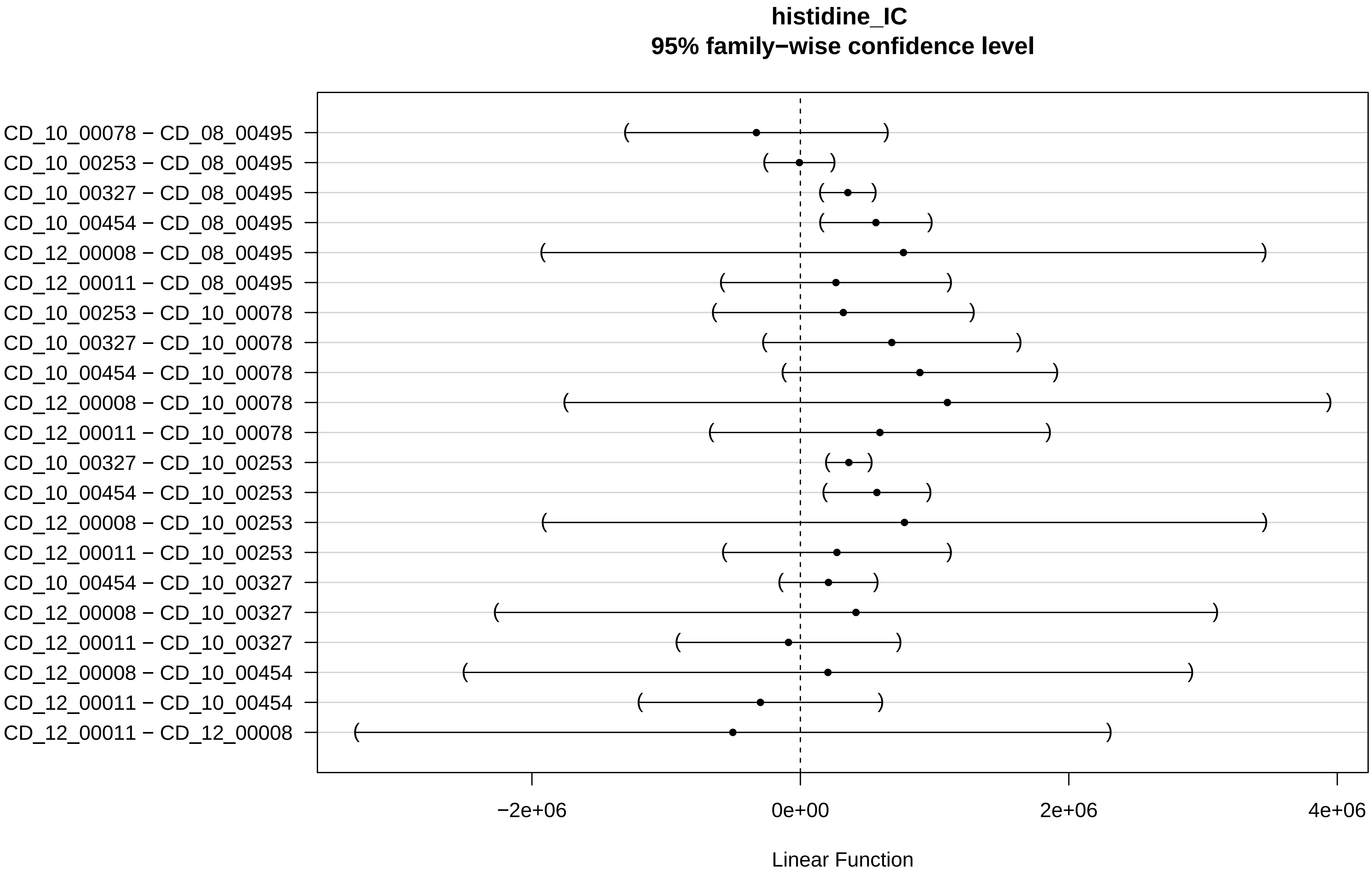
histidine_IC
95% family−wise confidence level
CD_10_00078 − CD_08_00495
CD_10_00253 − CD_08_00495
CD_10_00327 − CD_08_00495
CD_10_00454 − CD_08_00495
CD_12_00008 − CD_08_00495
CD_12_00011 − CD_08_00495
CD_10_00253 − CD_10_00078
CD_10_00327 − CD_10_00078
CD_10_00454 − CD_10_00078
CD_12_00008 − CD_10_00078
CD_12_00011 − CD_10_00078
CD_10_00327 − CD_10_00253
CD_10_00454 − CD_10_00253
CD_12_00008 − CD_10_00253
CD_12_00011 − CD_10_00253
CD_10_00454 − CD_10_00327
CD_12_00008 − CD_10_00327
CD_12_00011 − CD_10_00327
CD_12_00008 − CD_10_00454
CD_12_00011 − CD_10_00454
CD_12_00011 − CD_12_00008
−2e+06
0e+00
2e+06
4e+06
Linear Function

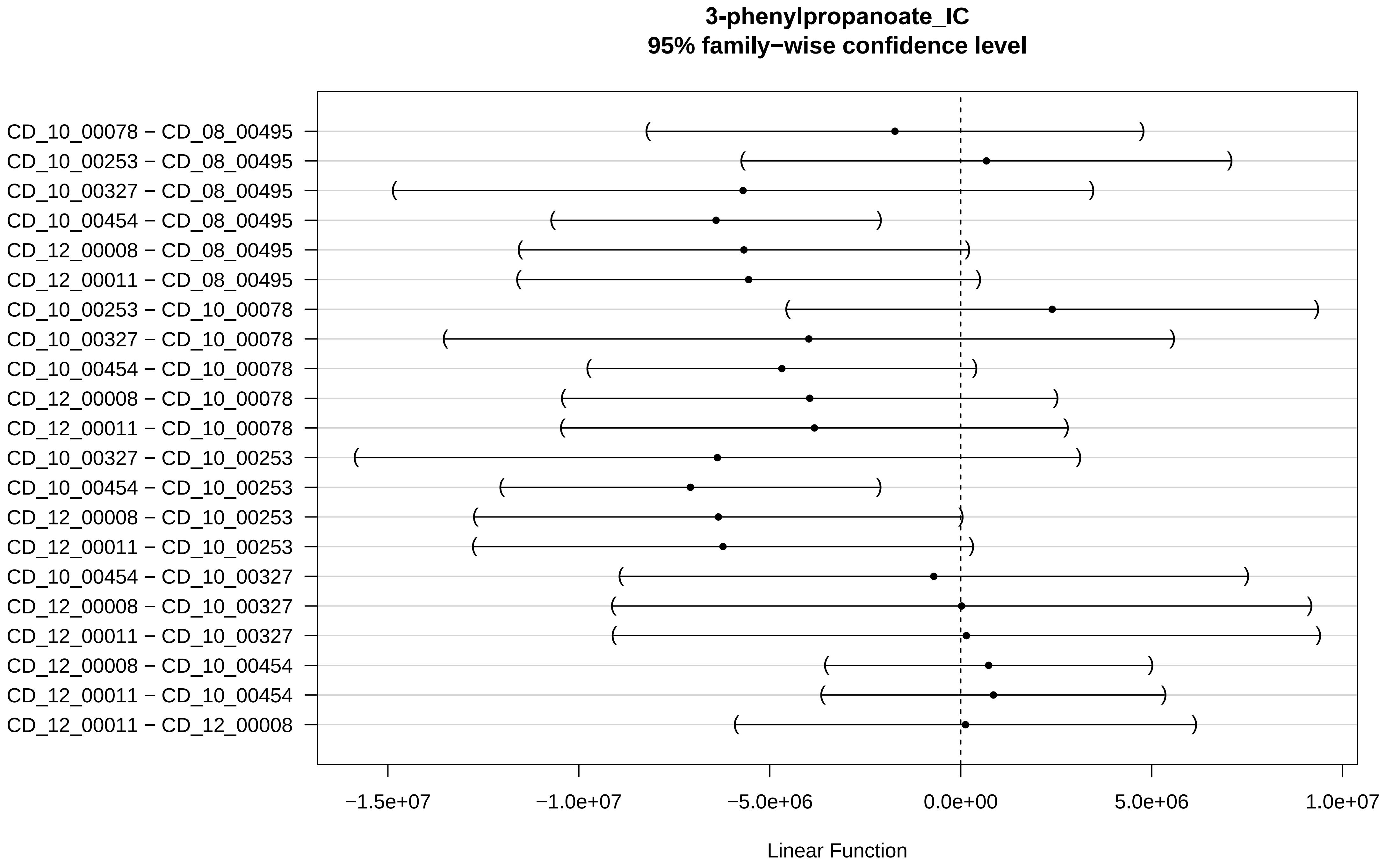

3-phenylpropanoate_IC
95% family−wise confidence level
CD_10_00078 − CD_08_00495
CD_10_00253 − CD_08_00495
CD_10_00327 − CD_08_00495
CD_10_00454 − CD_08_00495
CD_12_00008 − CD_08_00495
CD_12_00011 − CD_08_00495
CD_10_00253 − CD_10_00078
CD_10_00327 − CD_10_00078
CD_10_00454 − CD_10_00078
CD_12_00008 − CD_10_00078
CD_12_00011 − CD_10_00078
CD_10_00327 − CD_10_00253
CD_10_00454 − CD_10_00253
CD_12_00008 − CD_10_00253
CD_12_00011 − CD_10_00253
CD_10_00454 − CD_10_00327
CD_12_00008 − CD_10_00327
CD_12_00011 − CD_10_00327
CD_12_00008 − CD_10_00454
CD_12_00011 − CD_10_00454
CD_12_00011 − CD_12_00008
−1.5e+07
−1.0e+07
−5.0e+06
0.0e+00
5.0e+06
1.0e+07
Linear Function

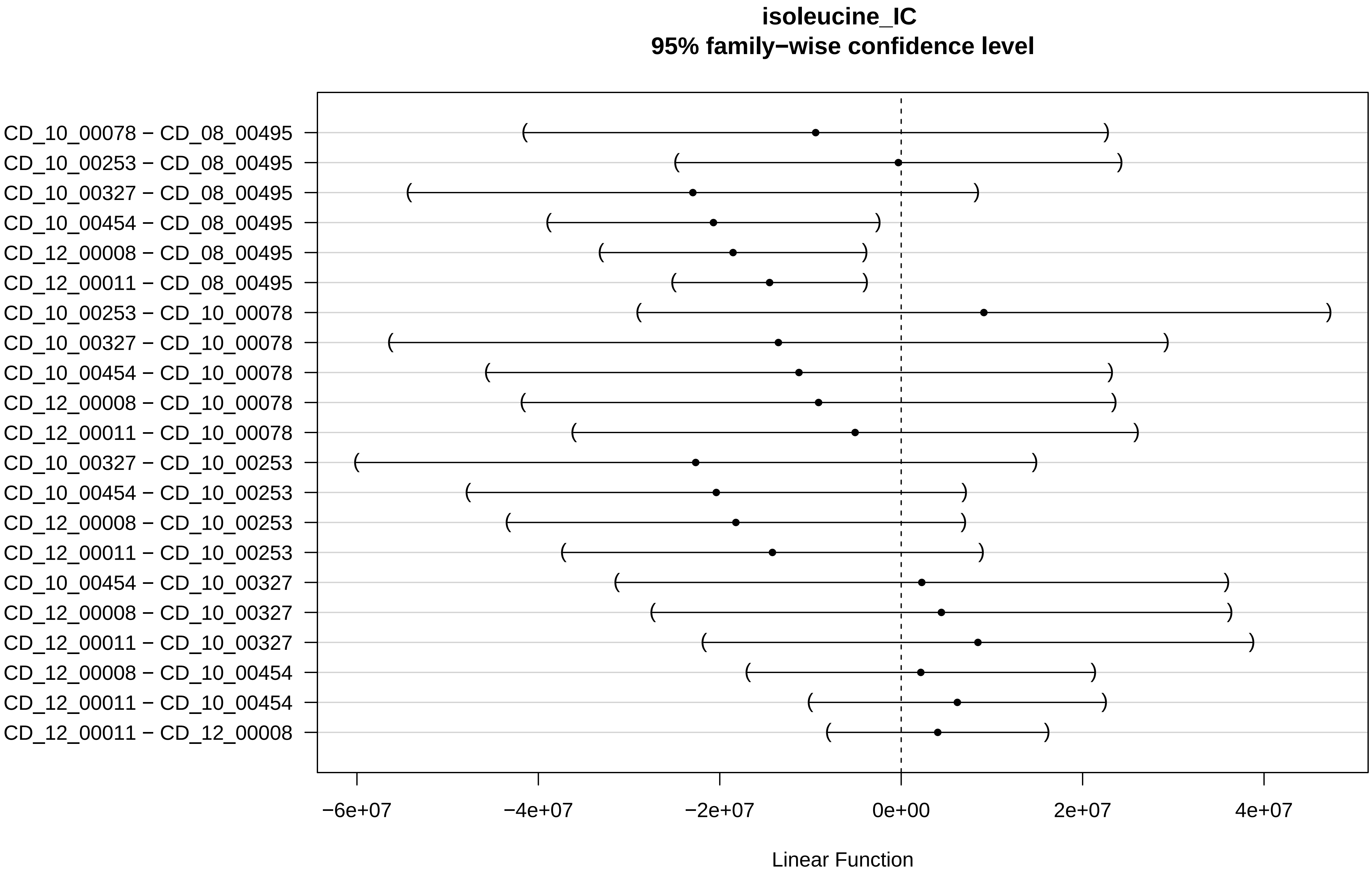
isoleucine_IC
95% family−wise confidence level
CD_10_00078 − CD_08_00495
CD_10_00253 − CD_08_00495
CD_10_00327 − CD_08_00495
CD_10_00454 − CD_08_00495
CD_12_00008 − CD_08_00495
CD_12_00011 − CD_08_00495
CD_10_00253 − CD_10_00078
CD_10_00327 − CD_10_00078
CD_10_00454 − CD_10_00078
CD_12_00008 − CD_10_00078
CD_12_00011 − CD_10_00078
CD_10_00327 − CD_10_00253
CD_10_00454 − CD_10_00253
CD_12_00008 − CD_10_00253
CD_12_00011 − CD_10_00253
CD_10_00454 − CD_10_00327
CD_12_00008 − CD_10_00327
CD_12_00011 − CD_10_00327
CD_12_00008 − CD_10_00454
CD_12_00011 − CD_10_00454
CD_12_00011 − CD_12_00008
−6e+07
−4e+07
−2e+07
0e+00
2e+07
4e+07
Linear Function

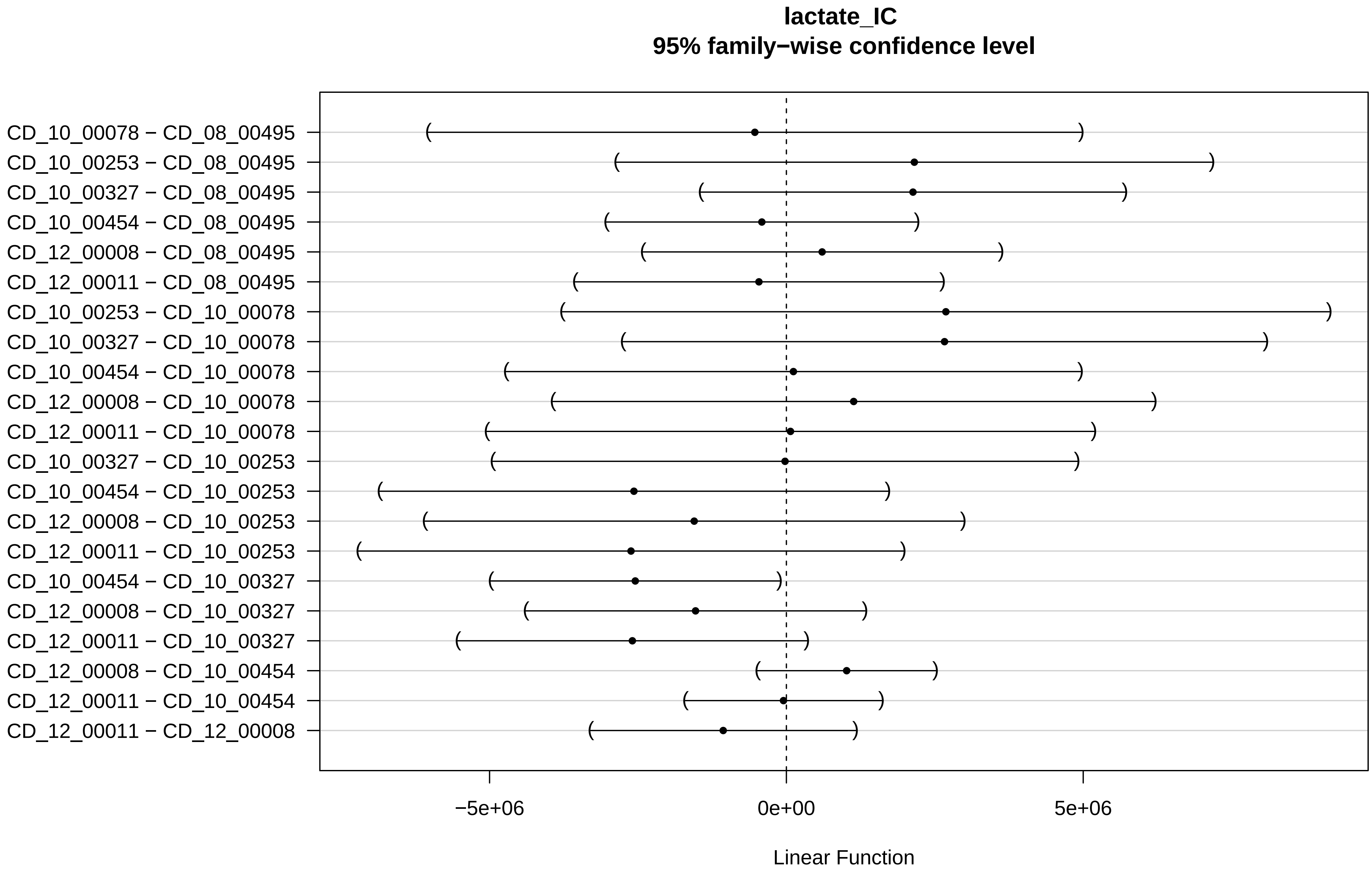
lactate_IC
95% family−wise confidence level
CD_10_00078 − CD_08_00495
CD_10_00253 − CD_08_00495
CD_10_00327 − CD_08_00495
CD_10_00454 − CD_08_00495
CD_12_00008 − CD_08_00495
CD_12_00011 − CD_08_00495
CD_10_00253 − CD_10_00078
CD_10_00327 − CD_10_00078
CD_10_00454 − CD_10_00078
CD_12_00008 − CD_10_00078
CD_12_00011 − CD_10_00078
CD_10_00327 − CD_10_00253
CD_10_00454 − CD_10_00253
CD_12_00008 − CD_10_00253
CD_12_00011 − CD_10_00253
CD_10_00454 − CD_10_00327
CD_12_00008 − CD_10_00327
CD_12_00011 − CD_10_00327
CD_12_00008 − CD_10_00454
CD_12_00011 − CD_10_00454
CD_12_00011 − CD_12_00008
−5e+06
0e+00
5e+06
Linear Function

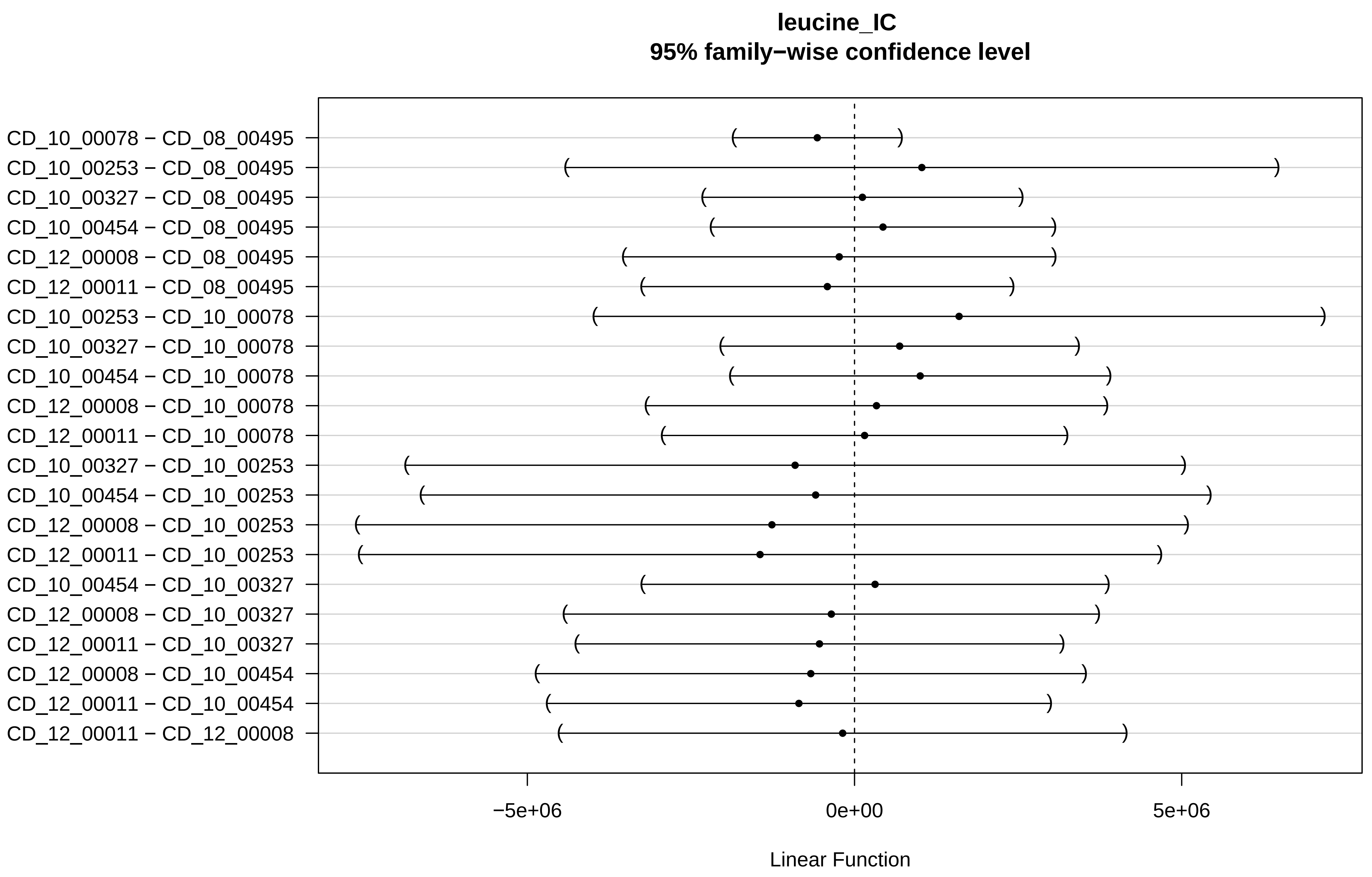
leucine_IC
95% family−wise confidence level
CD_10_00078 − CD_08_00495
CD_10_00253 − CD_08_00495
CD_10_00327 − CD_08_00495
CD_10_00454 − CD_08_00495
CD_12_00008 − CD_08_00495
CD_12_00011 − CD_08_00495
CD_10_00253 − CD_10_00078
CD_10_00327 − CD_10_00078
CD_10_00454 − CD_10_00078
CD_12_00008 − CD_10_00078
CD_12_00011 − CD_10_00078
CD_10_00327 − CD_10_00253
CD_10_00454 − CD_10_00253
CD_12_00008 − CD_10_00253
CD_12_00011 − CD_10_00253
CD_10_00454 − CD_10_00327
CD_12_00008 − CD_10_00327
CD_12_00011 − CD_10_00327
CD_12_00008 − CD_10_00454
CD_12_00011 − CD_10_00454
CD_12_00011 − CD_12_00008
−5e+06
0e+00
5e+06
Linear Function

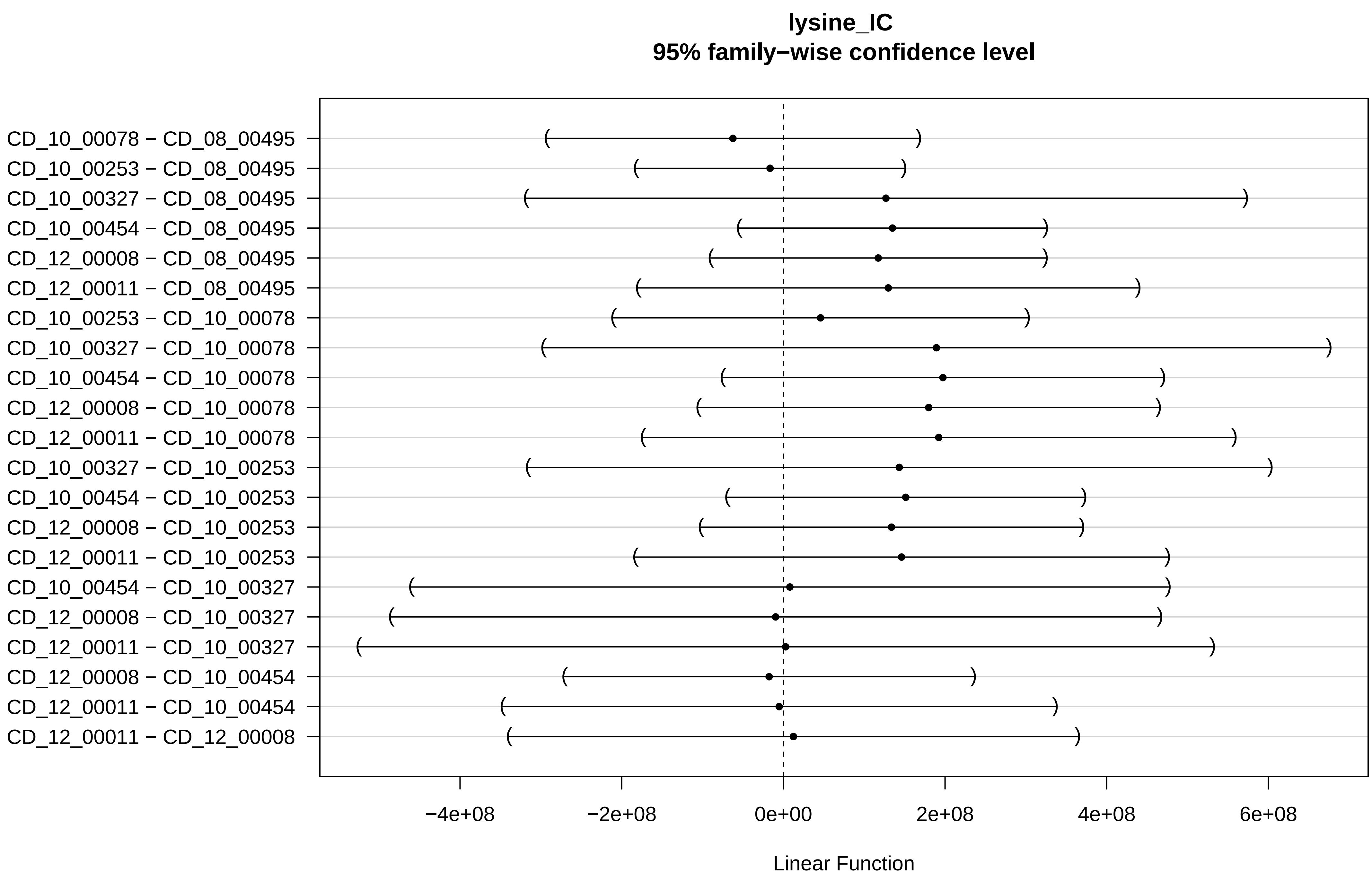

lysine_IC
95% family−wise confidence level
CD_10_00078 − CD_08_00495
CD_10_00253 − CD_08_00495
CD_10_00327 − CD_08_00495
CD_10_00454 − CD_08_00495
CD_12_00008 − CD_08_00495
CD_12_00011 − CD_08_00495
CD_10_00253 − CD_10_00078
CD_10_00327 − CD_10_00078
CD_10_00454 − CD_10_00078
CD_12_00008 − CD_10_00078
CD_12_00011 − CD_10_00078
CD_10_00327 − CD_10_00253
CD_10_00454 − CD_10_00253
CD_12_00008 − CD_10_00253
CD_12_00011 − CD_10_00253
CD_10_00454 − CD_10_00327
CD_12_00008 − CD_10_00327
CD_12_00011 − CD_10_00327
CD_12_00008 − CD_10_00454
CD_12_00011 − CD_10_00454
CD_12_00011 − CD_12_00008
−4e+08
−2e+08
0e+00
2e+08
4e+08
6e+08
Linear Function

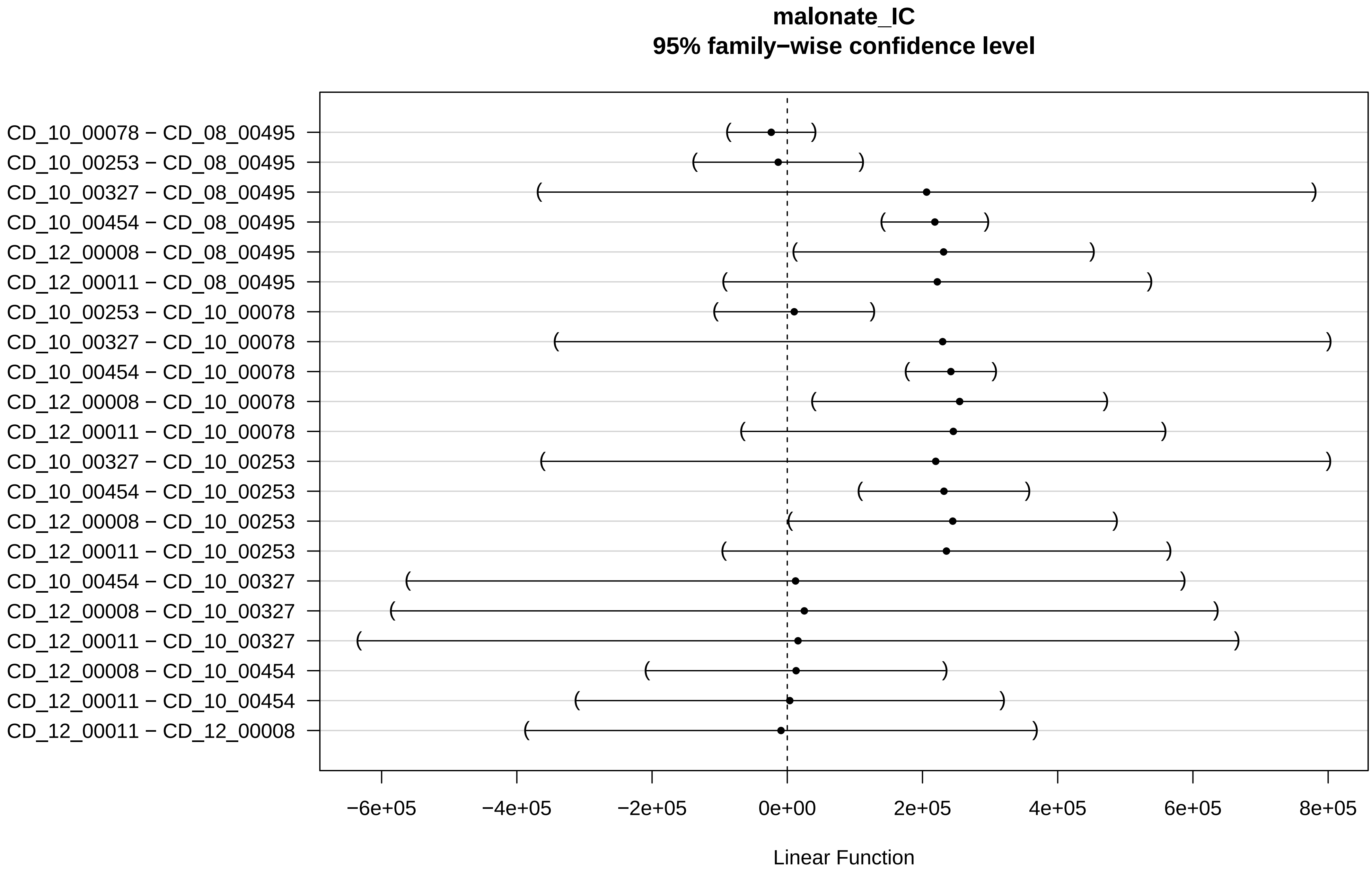
malonate_IC
95% family−wise confidence level
CD_10_00078 − CD_08_00495
CD_10_00253 − CD_08_00495
CD_10_00327 − CD_08_00495
CD_10_00454 − CD_08_00495
CD_12_00008 − CD_08_00495
CD_12_00011 − CD_08_00495
CD_10_00253 − CD_10_00078
CD_10_00327 − CD_10_00078
CD_10_00454 − CD_10_00078
CD_12_00008 − CD_10_00078
CD_12_00011 − CD_10_00078
CD_10_00327 − CD_10_00253
CD_10_00454 − CD_10_00253
CD_12_00008 − CD_10_00253
CD_12_00011 − CD_10_00253
CD_10_00454 − CD_10_00327
CD_12_00008 − CD_10_00327
CD_12_00011 − CD_10_00327
CD_12_00008 − CD_10_00454
CD_12_00011 − CD_10_00454
CD_12_00011 − CD_12_00008
−6e+05
−4e+05
−2e+05
0e+00
2e+05
4e+05
6e+05
8e+05
Linear Function

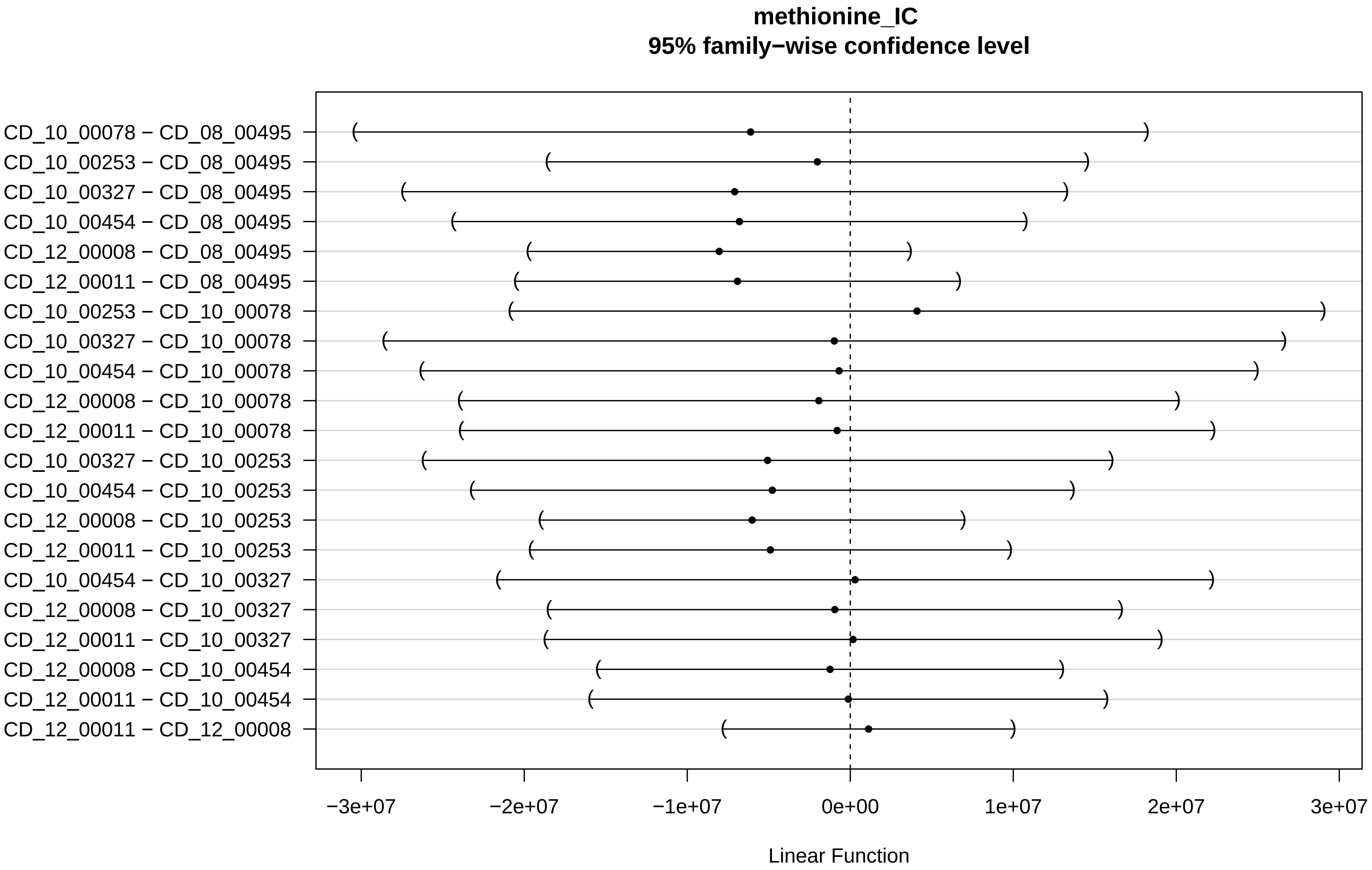
methionine_IC
95% family−wise confidence level
CD_10_00078 − CD_08_00495
CD_10_00253 − CD_08_00495
CD_10_00327 − CD_08_00495
CD_10_00454 − CD_08_00495
CD_12_00008 − CD_08_00495
CD_12_00011 − CD_08_00495
CD_10_00253 − CD_10_00078
CD_10_00327 − CD_10_00078
CD_10_00454 − CD_10_00078
CD_12_00008 − CD_10_00078
CD_12_00011 − CD_10_00078
CD_10_00327 − CD_10_00253
CD_10_00454 − CD_10_00253
CD_12_00008 − CD_10_00253
CD_12_00011 − CD_10_00253
CD_10_00454 − CD_10_00327
CD_12_00008 − CD_10_00327
CD_12_00011 − CD_10_00327
CD_12_00008 − CD_10_00454
CD_12_00011 − CD_10_00454
CD_12_00011 − CD_12_00008
−3e+07
−2e+07
−1e+07
0e+00
1e+07
2e+07
3e+07
Linear Function

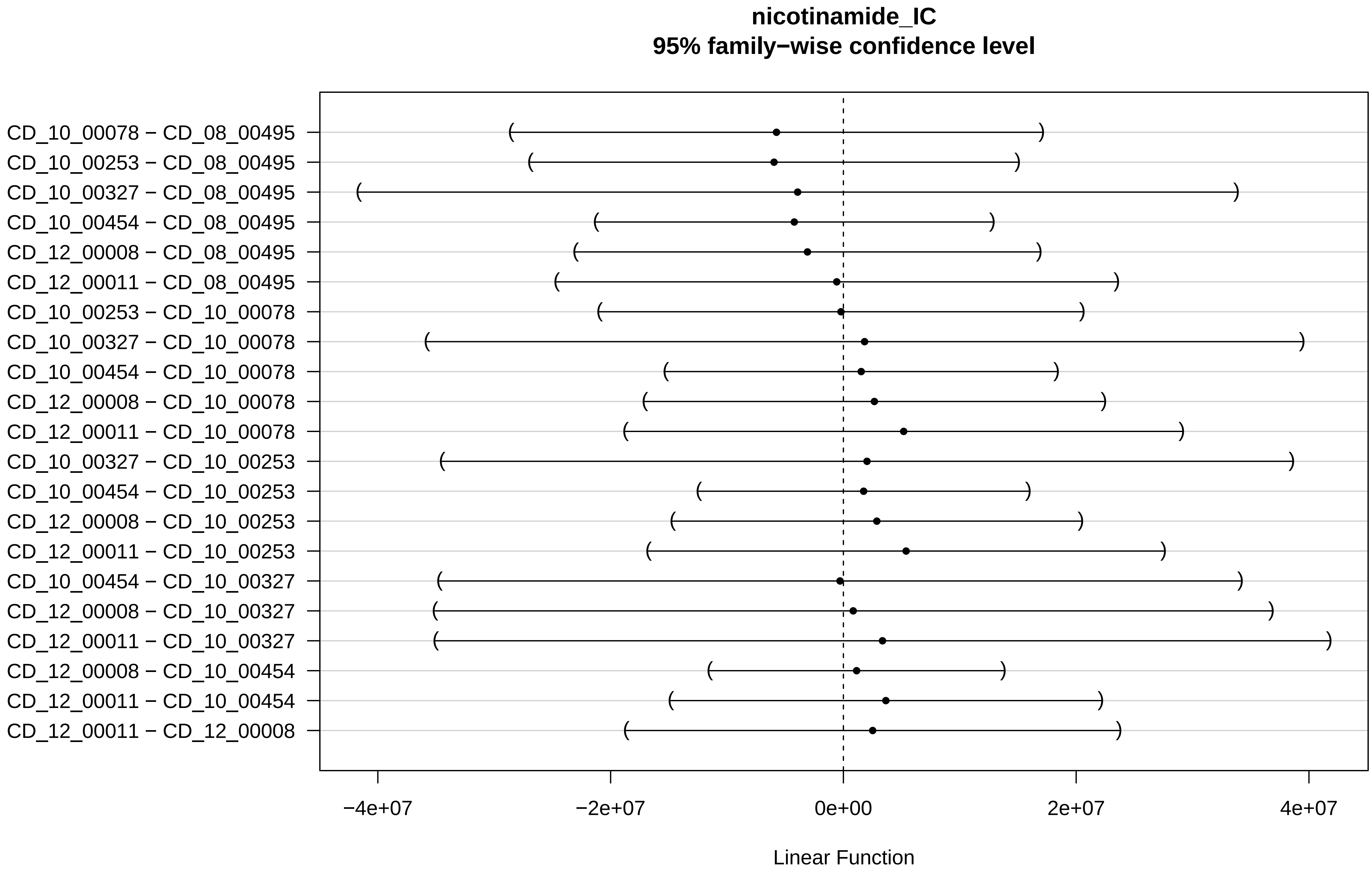
nicotinamide_IC
95% family−wise confidence level
CD_10_00078 − CD_08_00495
CD_10_00253 − CD_08_00495
CD_10_00327 − CD_08_00495
CD_10_00454 − CD_08_00495
CD_12_00008 − CD_08_00495
CD_12_00011 − CD_08_00495
CD_10_00253 − CD_10_00078
CD_10_00327 − CD_10_00078
CD_10_00454 − CD_10_00078
CD_12_00008 − CD_10_00078
CD_12_00011 − CD_10_00078
CD_10_00327 − CD_10_00253
CD_10_00454 − CD_10_00253
CD_12_00008 − CD_10_00253
CD_12_00011 − CD_10_00253
CD_10_00454 − CD_10_00327
CD_12_00008 − CD_10_00327
CD_12_00011 − CD_10_00327
CD_12_00008 − CD_10_00454
CD_12_00011 − CD_10_00454
CD_12_00011 − CD_12_00008
−4e+07
−2e+07
0e+00
2e+07
4e+07
Linear Function

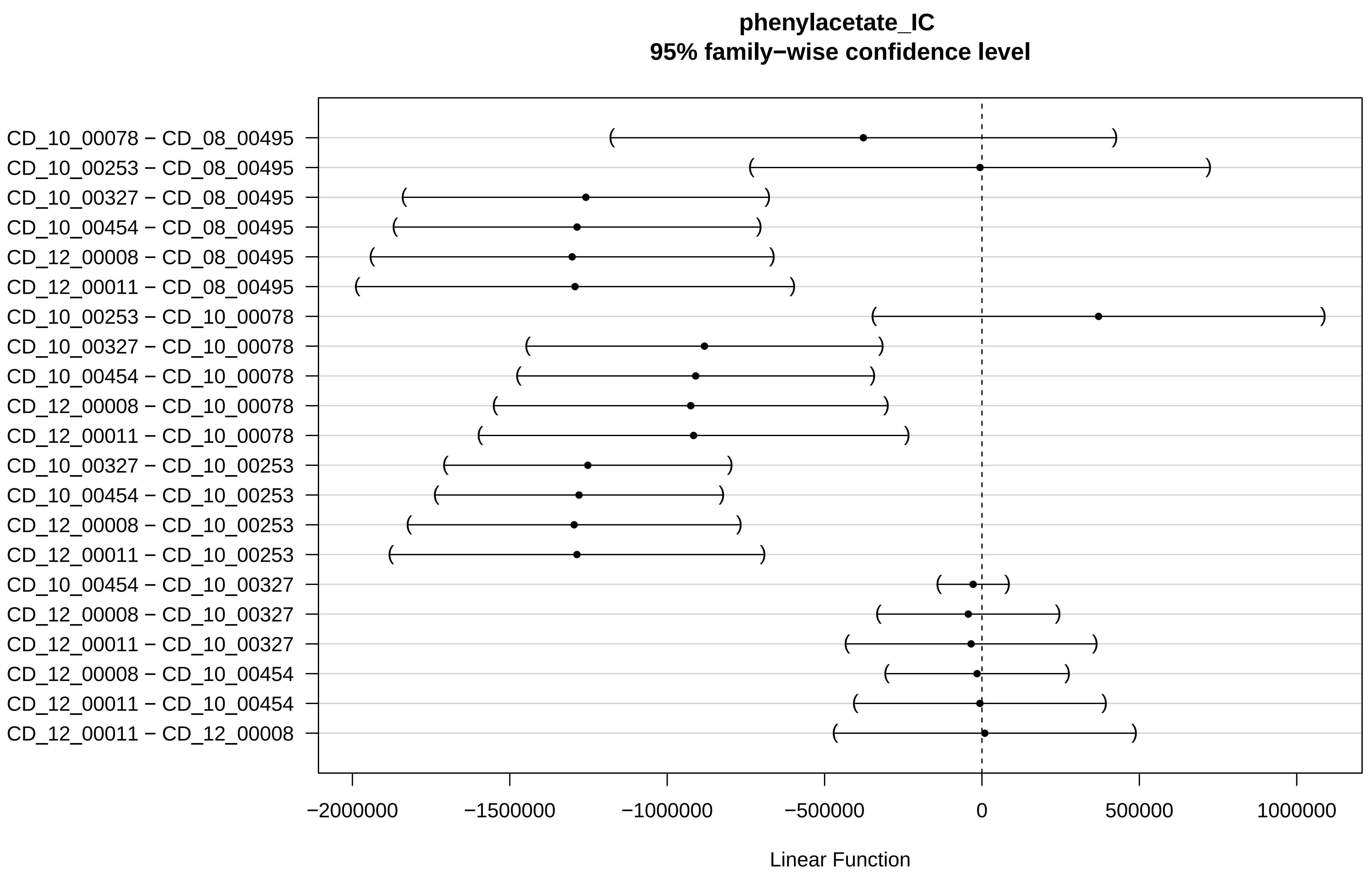
phenylacetate_IC
95% family−wise confidence level
CD_10_00078 − CD_08_00495
CD_10_00253 − CD_08_00495
CD_10_00327 − CD_08_00495
CD_10_00454 − CD_08_00495
CD_12_00008 − CD_08_00495
CD_12_00011 − CD_08_00495
CD_10_00253 − CD_10_00078
CD_10_00327 − CD_10_00078
CD_10_00454 − CD_10_00078
CD_12_00008 − CD_10_00078
CD_12_00011 − CD_10_00078
CD_10_00327 − CD_10_00253
CD_10_00454 − CD_10_00253
CD_12_00008 − CD_10_00253
CD_12_00011 − CD_10_00253
CD_10_00454 − CD_10_00327
CD_12_00008 − CD_10_00327
CD_12_00011 − CD_10_00327
CD_12_00008 − CD_10_00454
CD_12_00011 − CD_10_00454
CD_12_00011 − CD_12_00008
−2000000
−1500000
−1000000
−500000
0
500000
1000000
Linear Function

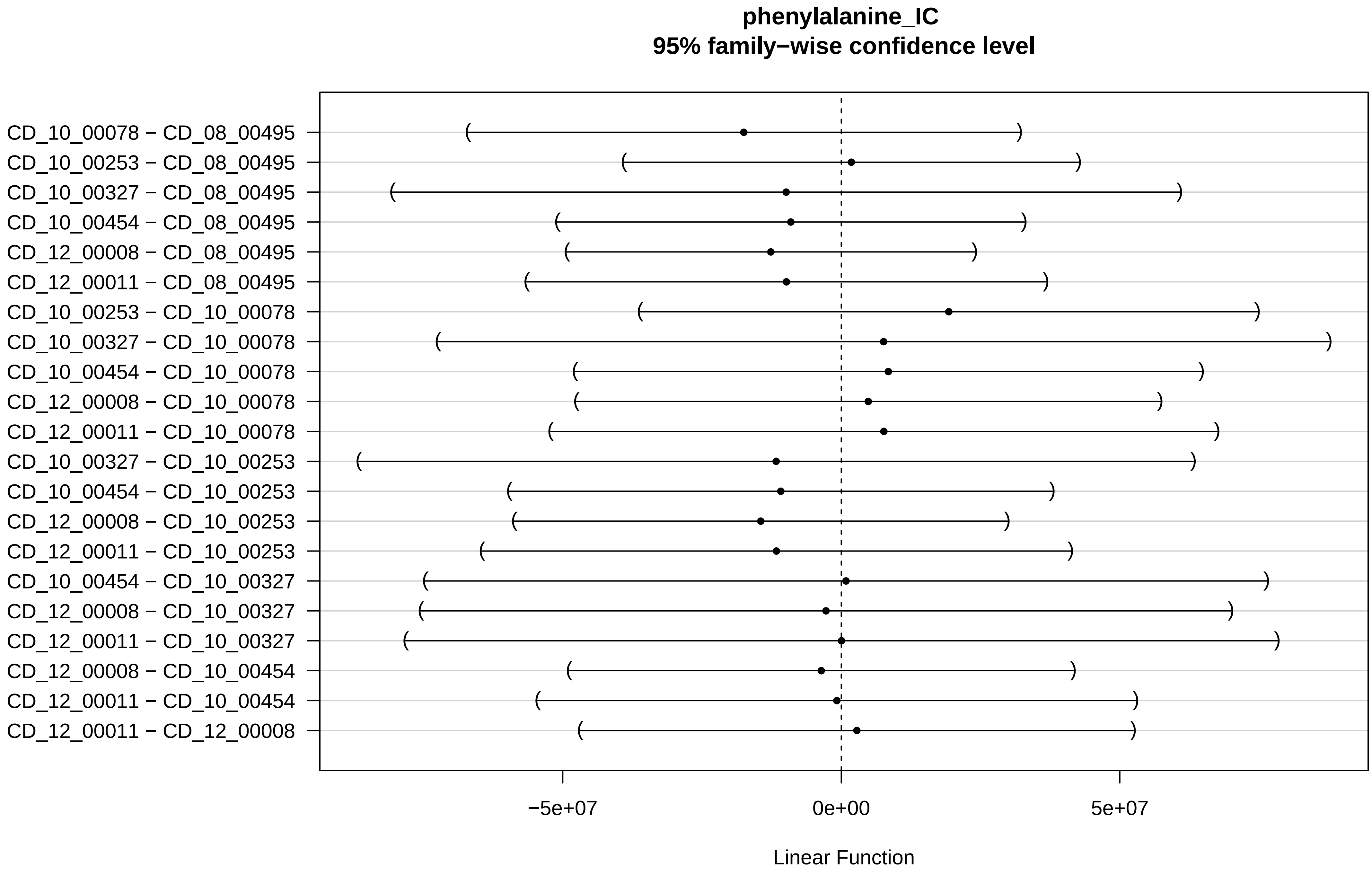
phenylalanine_IC
95% family−wise confidence level
CD_10_00078 − CD_08_00495
CD_10_00253 − CD_08_00495
CD_10_00327 − CD_08_00495
CD_10_00454 − CD_08_00495
CD_12_00008 − CD_08_00495
CD_12_00011 − CD_08_00495
CD_10_00253 − CD_10_00078
CD_10_00327 − CD_10_00078
CD_10_00454 − CD_10_00078
CD_12_00008 − CD_10_00078
CD_12_00011 − CD_10_00078
CD_10_00327 − CD_10_00253
CD_10_00454 − CD_10_00253
CD_12_00008 − CD_10_00253
CD_12_00011 − CD_10_00253
CD_10_00454 − CD_10_00327
CD_12_00008 − CD_10_00327
CD_12_00011 − CD_10_00327
CD_12_00008 − CD_10_00454
CD_12_00011 − CD_10_00454
CD_12_00011 − CD_12_00008
−5e+07
0e+00
5e+07
Linear Function

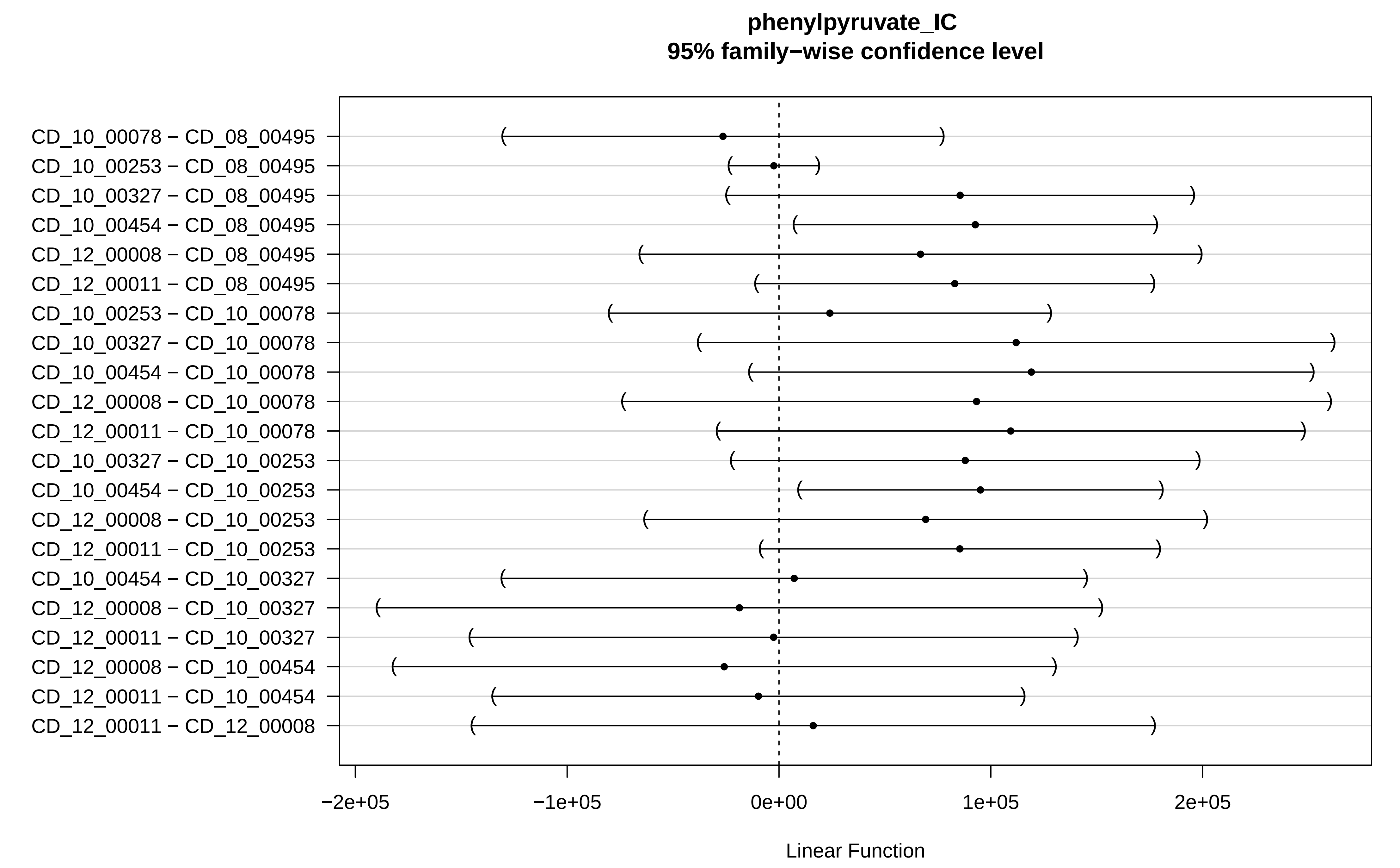
phenylpyruvate_IC
95% family−wise confidence level
CD_10_00078 − CD_08_00495
CD_10_00253 − CD_08_00495
CD_10_00327 − CD_08_00495
CD_10_00454 − CD_08_00495
CD_12_00008 − CD_08_00495
CD_12_00011 − CD_08_00495
CD_10_00253 − CD_10_00078
CD_10_00327 − CD_10_00078
CD_10_00454 − CD_10_00078
CD_12_00008 − CD_10_00078
CD_12_00011 − CD_10_00078
CD_10_00327 − CD_10_00253
CD_10_00454 − CD_10_00253
CD_12_00008 − CD_10_00253
CD_12_00011 − CD_10_00253
CD_10_00454 − CD_10_00327
CD_12_00008 − CD_10_00327
CD_12_00011 − CD_10_00327
CD_12_00008 − CD_10_00454
CD_12_00011 − CD_10_00454
CD_12_00011 − CD_12_00008
−2e+05
−1e+05
0e+00
1e+05
2e+05
Linear Function

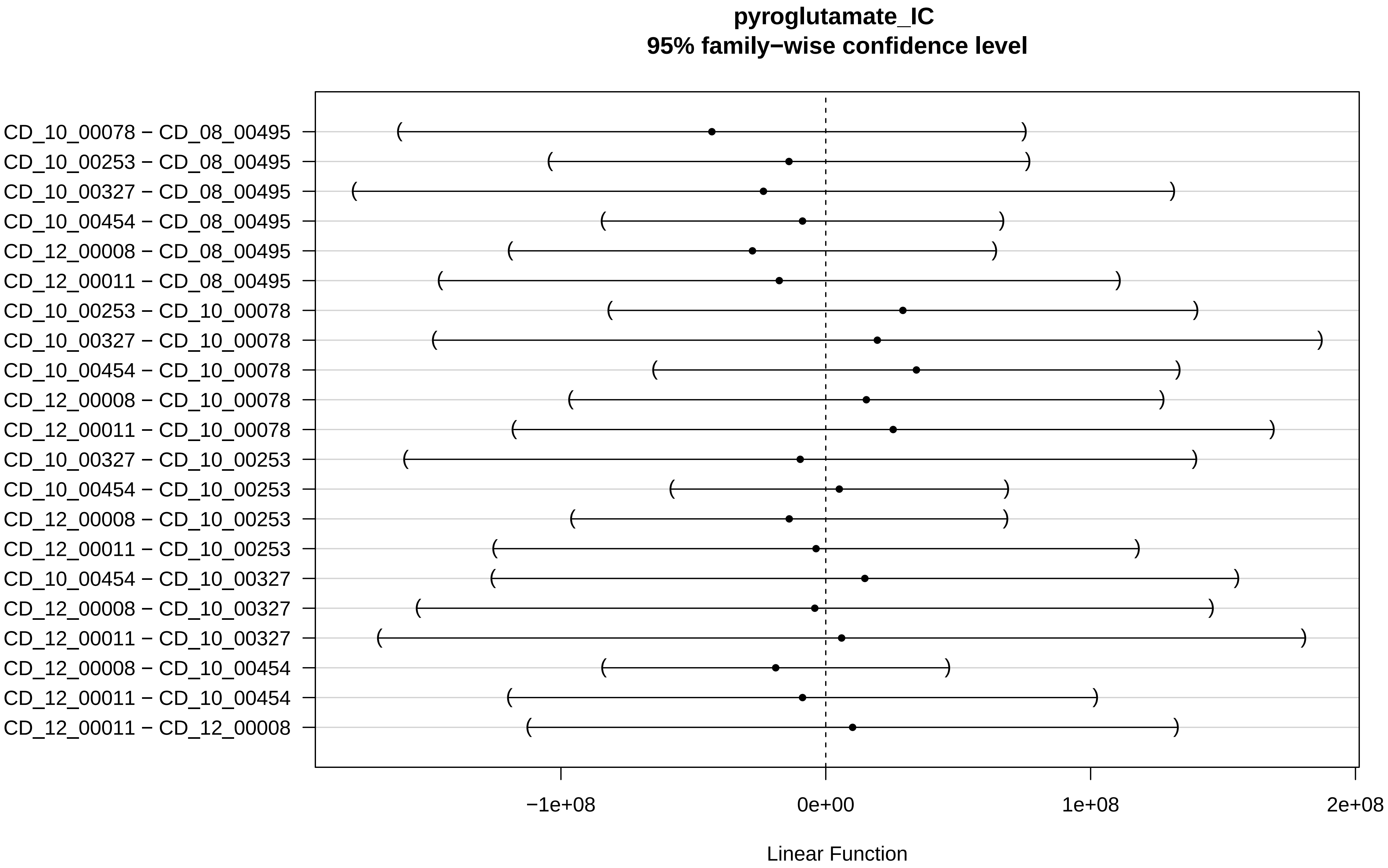
pyroglutamate_IC
95% family−wise confidence level
CD_10_00078 − CD_08_00495
CD_10_00253 − CD_08_00495
CD_10_00327 − CD_08_00495
CD_10_00454 − CD_08_00495
CD_12_00008 − CD_08_00495
CD_12_00011 − CD_08_00495
CD_10_00253 − CD_10_00078
CD_10_00327 − CD_10_00078
CD_10_00454 − CD_10_00078
CD_12_00008 − CD_10_00078
CD_12_00011 − CD_10_00078
CD_10_00327 − CD_10_00253
CD_10_00454 − CD_10_00253
CD_12_00008 − CD_10_00253
CD_12_00011 − CD_10_00253
CD_10_00454 − CD_10_00327
CD_12_00008 − CD_10_00327
CD_12_00011 − CD_10_00327
CD_12_00008 − CD_10_00454
CD_12_00011 − CD_10_00454
CD_12_00011 − CD_12_00008
−1e+08
0e+00
1e+08
2e+08
Linear Function

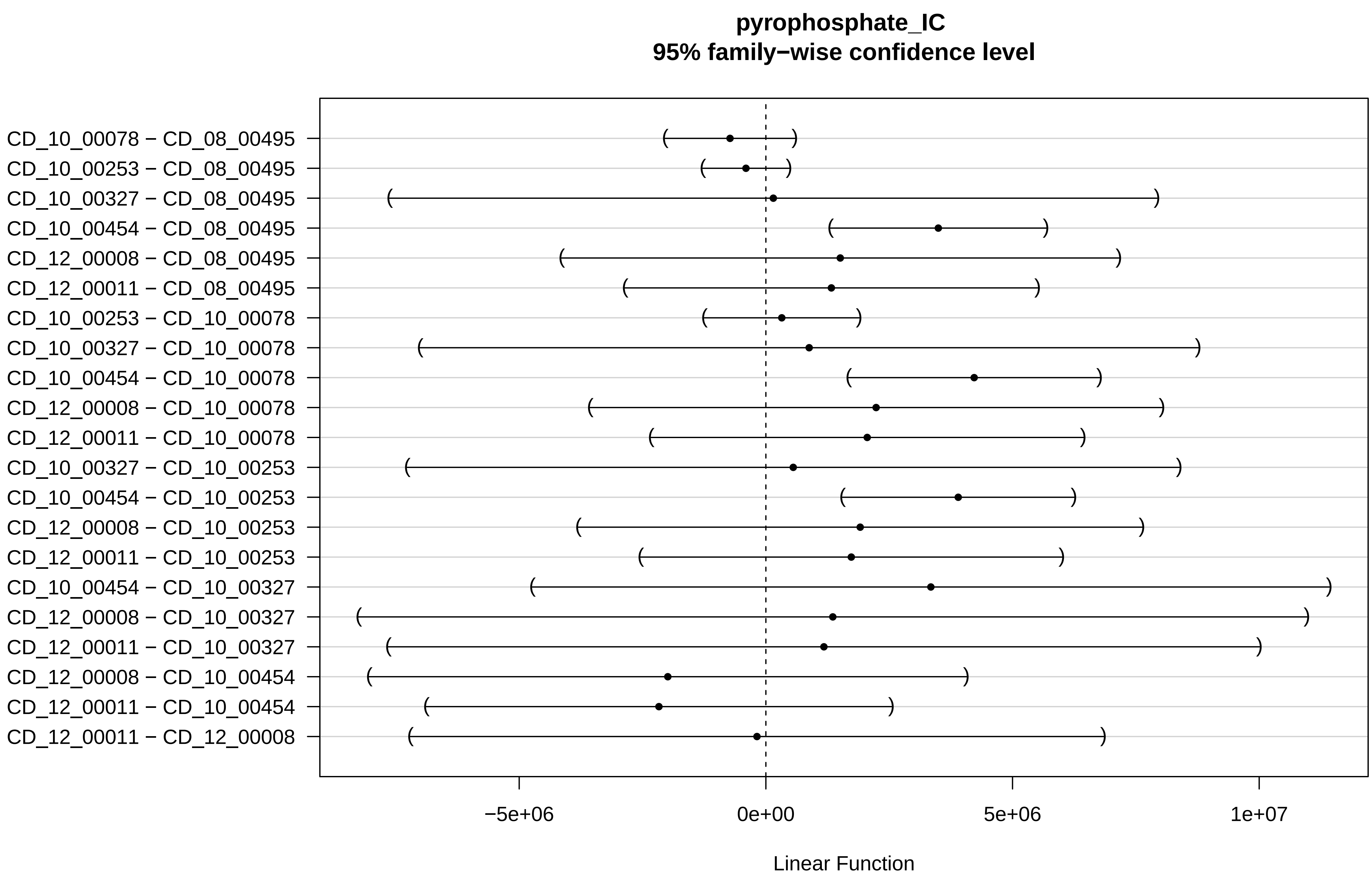

pyrophosphate_IC
95% family−wise confidence level
CD_10_00078 − CD_08_00495
CD_10_00253 − CD_08_00495
CD_10_00327 − CD_08_00495
CD_10_00454 − CD_08_00495
CD_12_00008 − CD_08_00495
CD_12_00011 − CD_08_00495
CD_10_00253 − CD_10_00078
CD_10_00327 − CD_10_00078
CD_10_00454 − CD_10_00078
CD_12_00008 − CD_10_00078
CD_12_00011 − CD_10_00078
CD_10_00327 − CD_10_00253
CD_10_00454 − CD_10_00253
CD_12_00008 − CD_10_00253
CD_12_00011 − CD_10_00253
CD_10_00454 − CD_10_00327
CD_12_00008 − CD_10_00327
CD_12_00011 − CD_10_00327
CD_12_00008 − CD_10_00454
CD_12_00011 − CD_10_00454
CD_12_00011 − CD_12_00008
−5e+06
0e+00
5e+06
1e+07
Linear Function

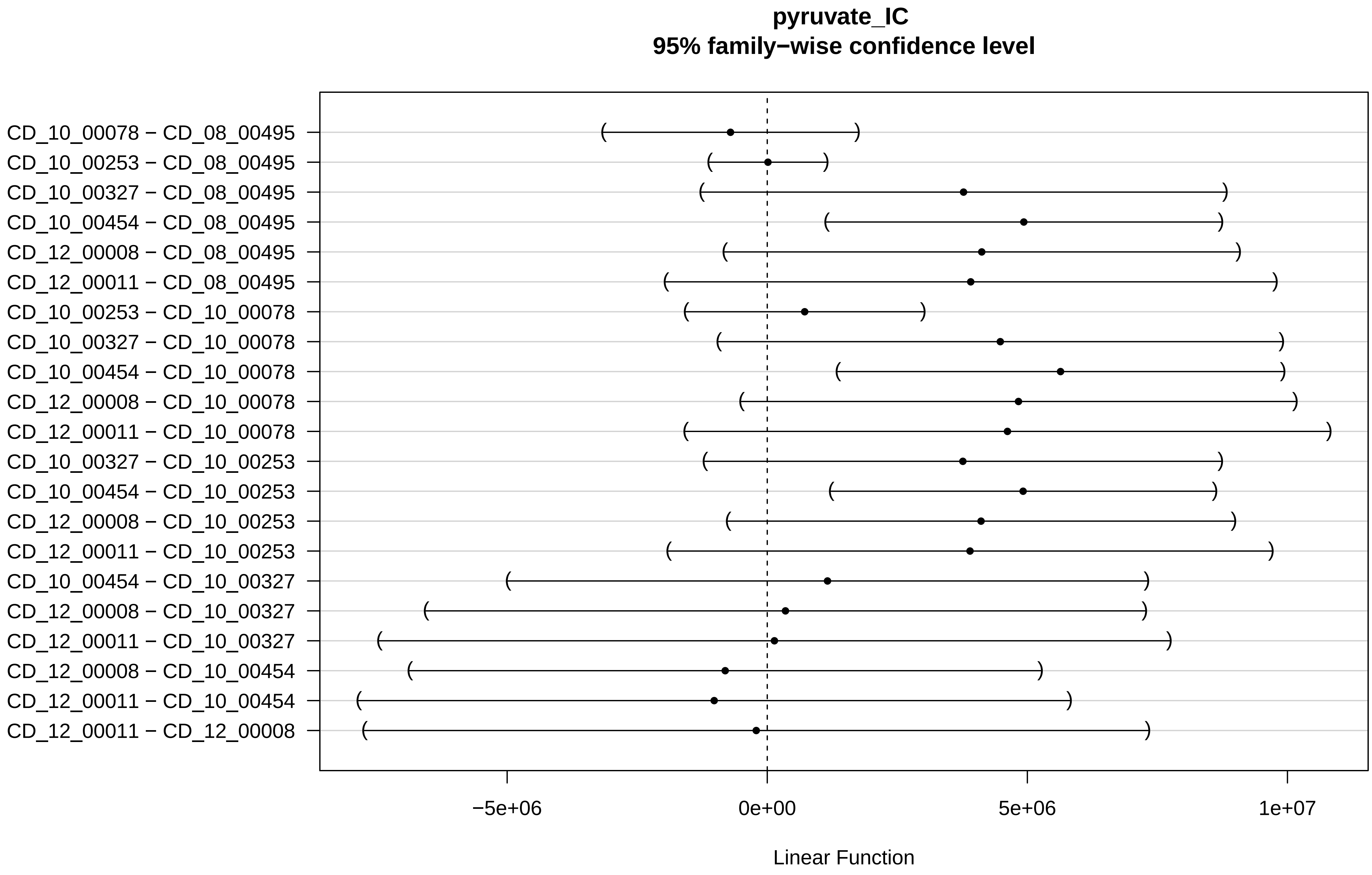
pyruvate_IC
95% family−wise confidence level
CD_10_00078 − CD_08_00495
CD_10_00253 − CD_08_00495
CD_10_00327 − CD_08_00495
CD_10_00454 − CD_08_00495
CD_12_00008 − CD_08_00495
CD_12_00011 − CD_08_00495
CD_10_00253 − CD_10_00078
CD_10_00327 − CD_10_00078
CD_10_00454 − CD_10_00078
CD_12_00008 − CD_10_00078
CD_12_00011 − CD_10_00078
CD_10_00327 − CD_10_00253
CD_10_00454 − CD_10_00253
CD_12_00008 − CD_10_00253
CD_12_00011 − CD_10_00253
CD_10_00454 − CD_10_00327
CD_12_00008 − CD_10_00327
CD_12_00011 − CD_10_00327
CD_12_00008 − CD_10_00454
CD_12_00011 − CD_10_00454
CD_12_00011 − CD_12_00008
−5e+06
0e+00
5e+06
1e+07
Linear Function

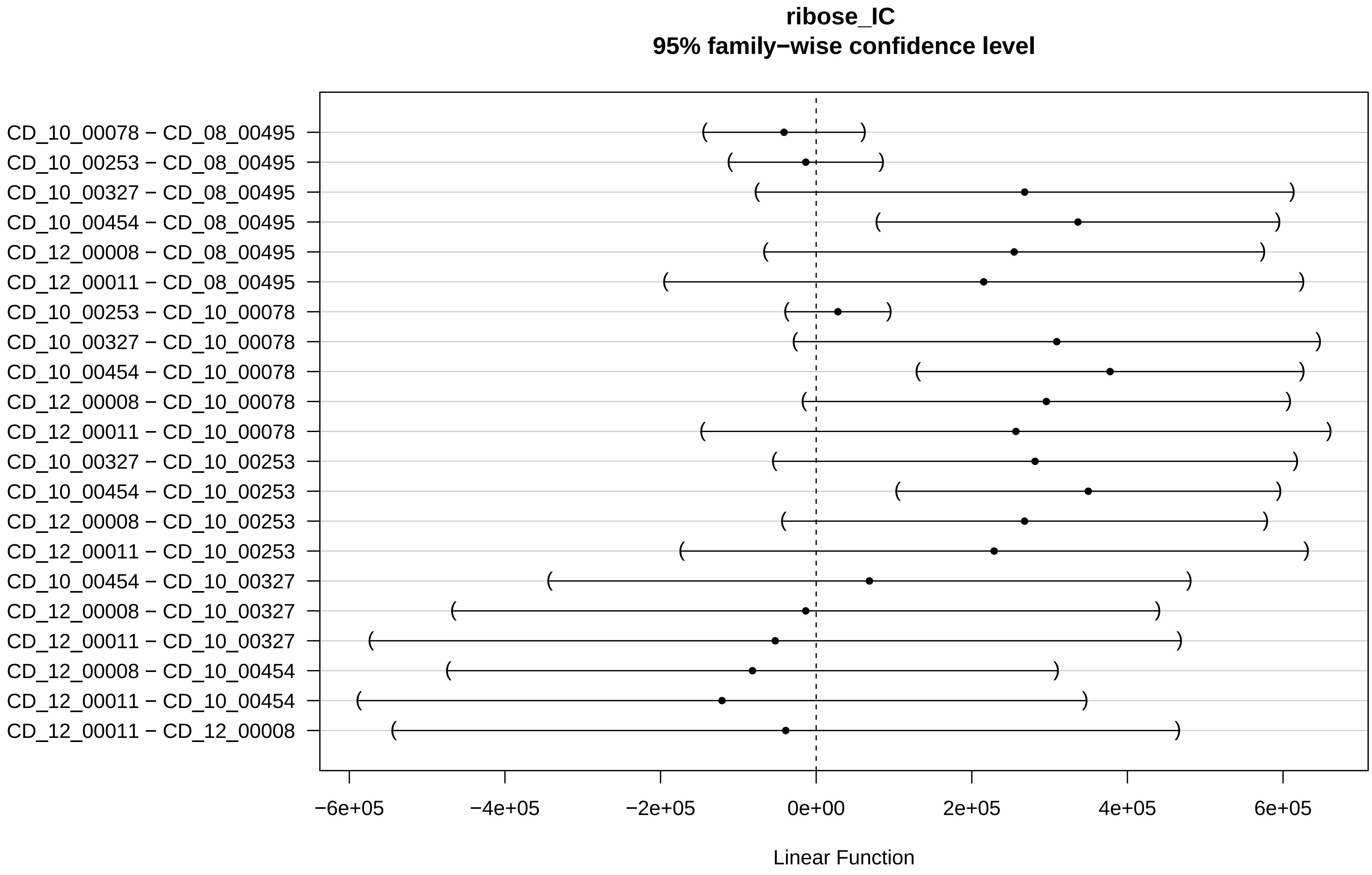
ribose_IC
95% family−wise confidence level
CD_10_00078 − CD_08_00495
CD_10_00253 − CD_08_00495
CD_10_00327 − CD_08_00495
CD_10_00454 − CD_08_00495
CD_12_00008 − CD_08_00495
CD_12_00011 − CD_08_00495
CD_10_00253 − CD_10_00078
CD_10_00327 − CD_10_00078
CD_10_00454 − CD_10_00078
CD_12_00008 − CD_10_00078
CD_12_00011 − CD_10_00078
CD_10_00327 − CD_10_00253
CD_10_00454 − CD_10_00253
CD_12_00008 − CD_10_00253
CD_12_00011 − CD_10_00253
CD_10_00454 − CD_10_00327
CD_12_00008 − CD_10_00327
CD_12_00011 − CD_10_00327
CD_12_00008 − CD_10_00454
CD_12_00011 − CD_10_00454
CD_12_00011 − CD_12_00008
−6e+05
−4e+05
−2e+05
0e+00
2e+05
4e+05
6e+05
Linear Function

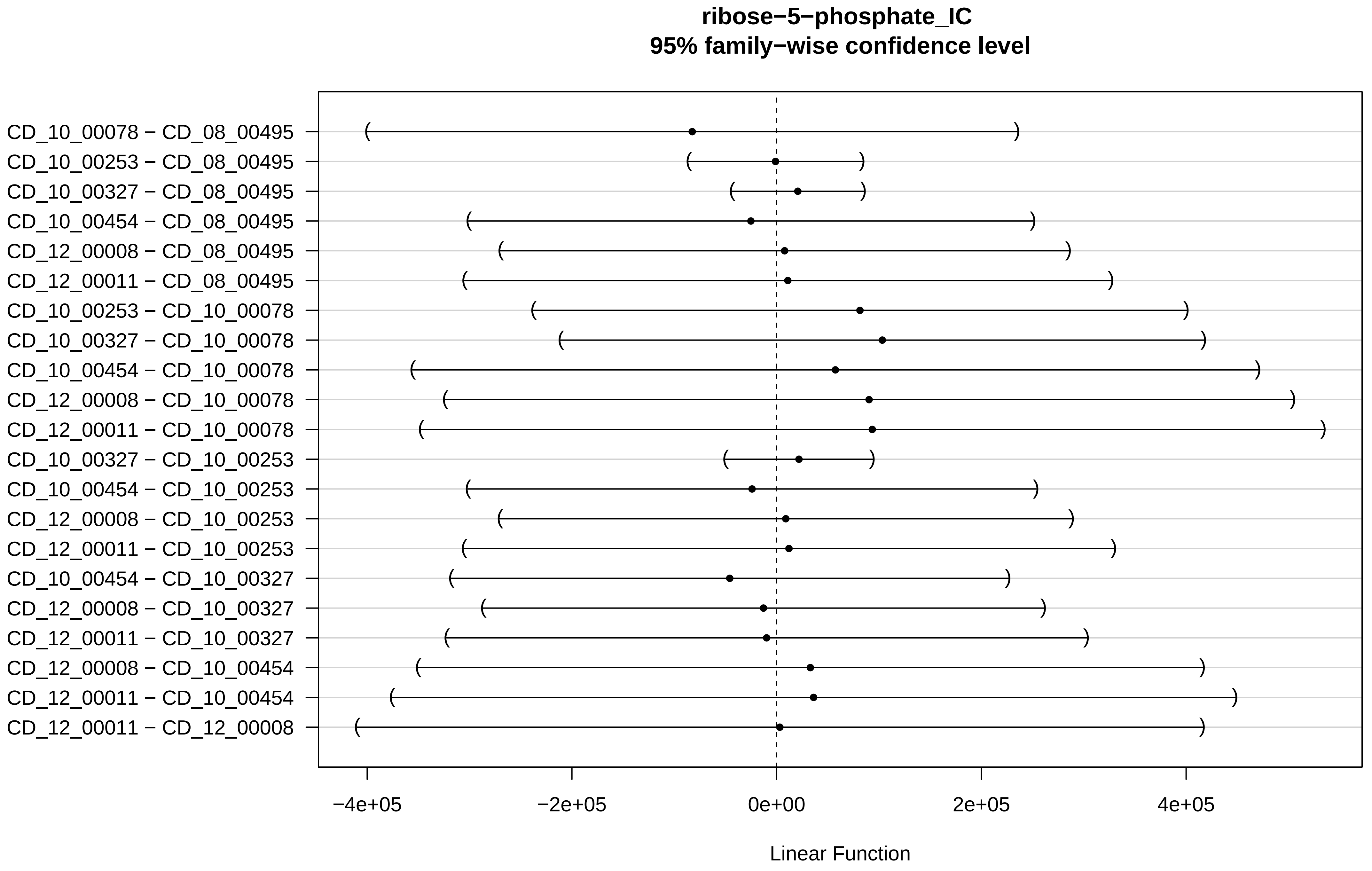
ribose−5−phosphate_IC
95% family−wise confidence level
CD_10_00078 − CD_08_00495
CD_10_00253 − CD_08_00495
CD_10_00327 − CD_08_00495
CD_10_00454 − CD_08_00495
CD_12_00008 − CD_08_00495
CD_12_00011 − CD_08_00495
CD_10_00253 − CD_10_00078
CD_10_00327 − CD_10_00078
CD_10_00454 − CD_10_00078
CD_12_00008 − CD_10_00078
CD_12_00011 − CD_10_00078
CD_10_00327 − CD_10_00253
CD_10_00454 − CD_10_00253
CD_12_00008 − CD_10_00253
CD_12_00011 − CD_10_00253
CD_10_00454 − CD_10_00327
CD_12_00008 − CD_10_00327
CD_12_00011 − CD_10_00327
CD_12_00008 − CD_10_00454
CD_12_00011 − CD_10_00454
CD_12_00011 − CD_12_00008
−4e+05
−2e+05
0e+00
2e+05
4e+05
Linear Function

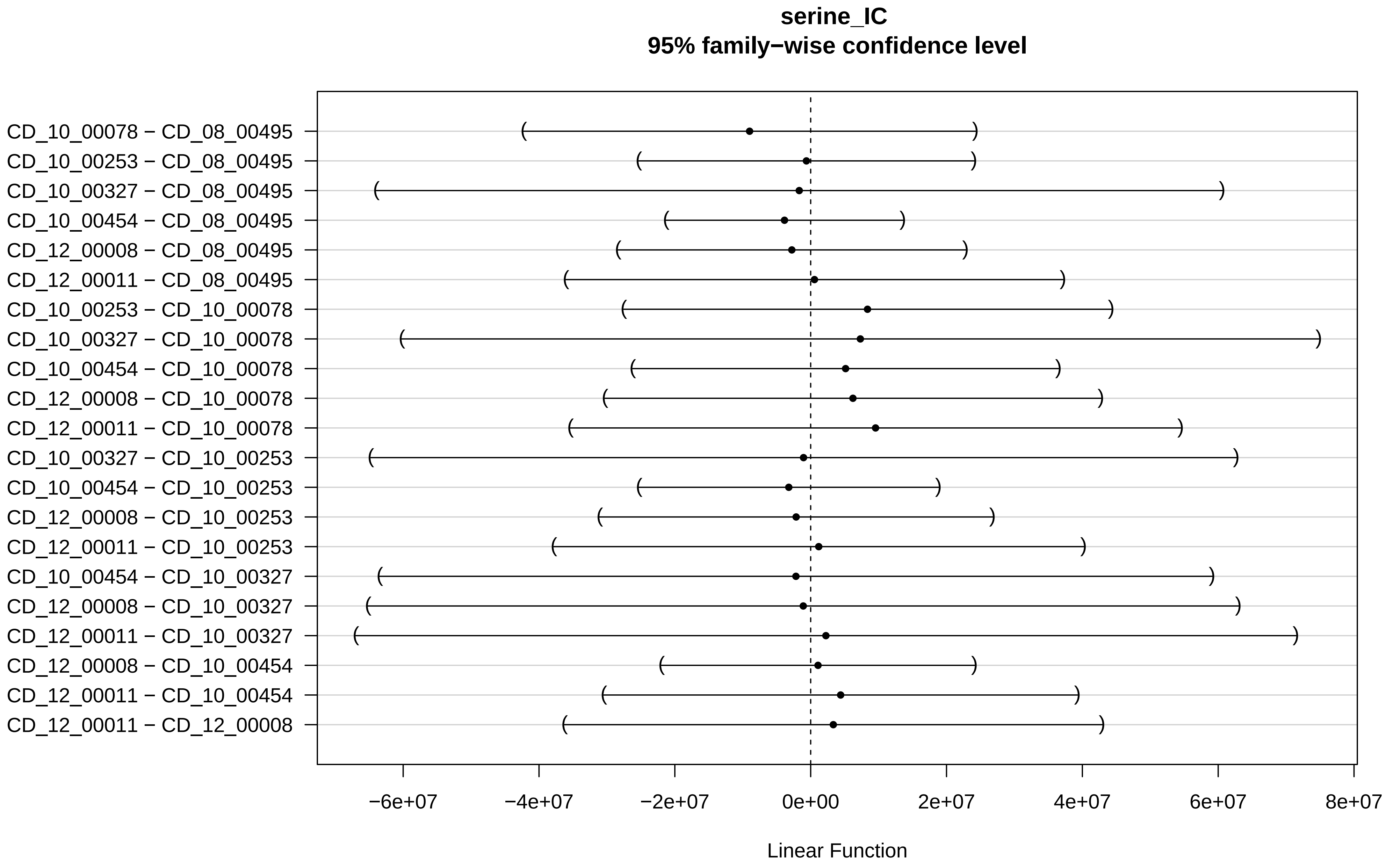
serine_IC
95% family−wise confidence level
CD_10_00078 − CD_08_00495
CD_10_00253 − CD_08_00495
CD_10_00327 − CD_08_00495
CD_10_00454 − CD_08_00495
CD_12_00008 − CD_08_00495
CD_12_00011 − CD_08_00495
CD_10_00253 − CD_10_00078
CD_10_00327 − CD_10_00078
CD_10_00454 − CD_10_00078
CD_12_00008 − CD_10_00078
CD_12_00011 − CD_10_00078
CD_10_00327 − CD_10_00253
CD_10_00454 − CD_10_00253
CD_12_00008 − CD_10_00253
CD_12_00011 − CD_10_00253
CD_10_00454 − CD_10_00327
CD_12_00008 − CD_10_00327
CD_12_00011 − CD_10_00327
CD_12_00008 − CD_10_00454
CD_12_00011 − CD_10_00454
CD_12_00011 − CD_12_00008
−6e+07
−4e+07
−2e+07
0e+00
2e+07
4e+07
6e+07
8e+07
Linear Function

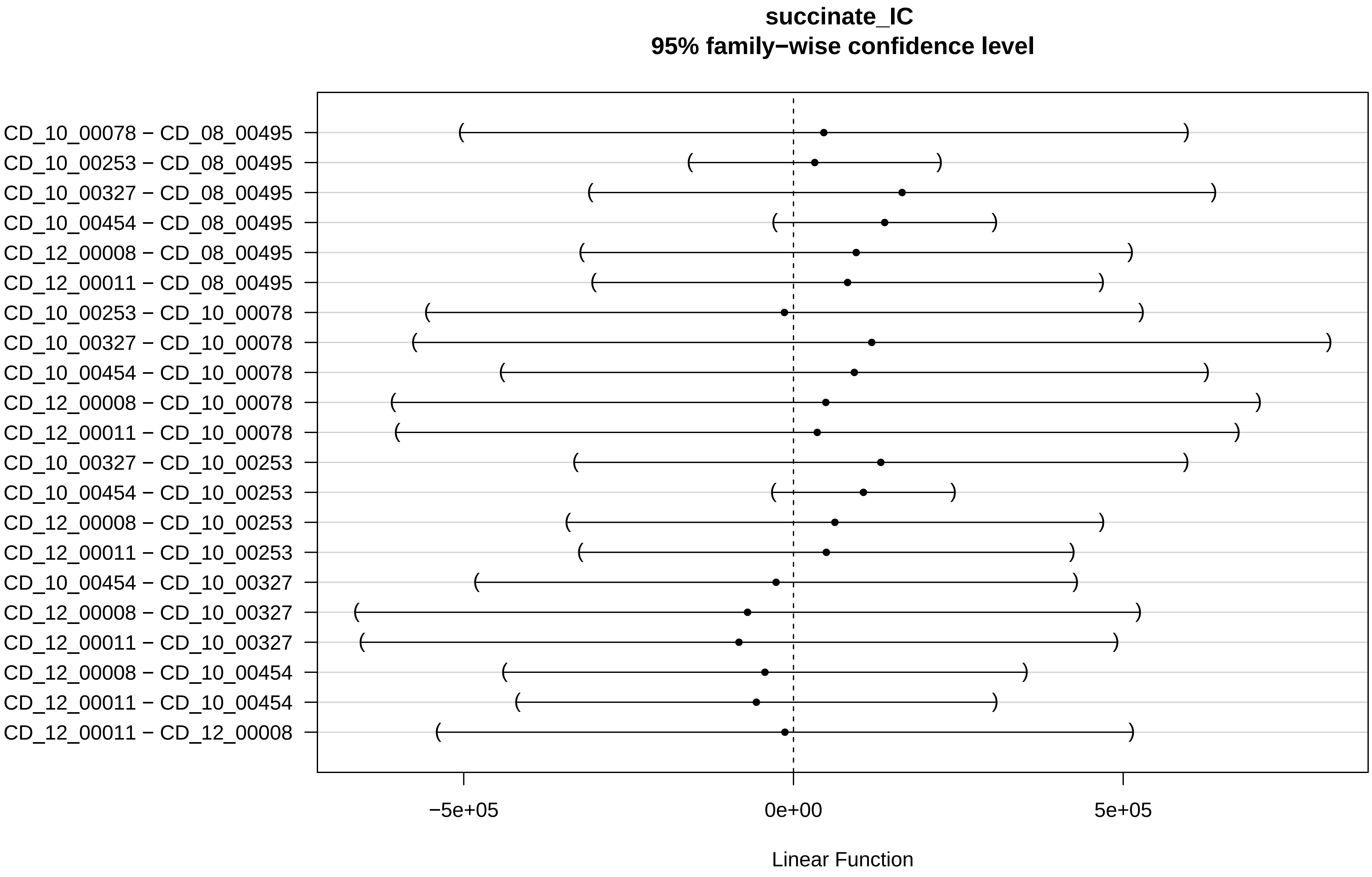
succinate_IC
95% family−wise confidence level
CD_10_00078 − CD_08_00495
CD_10_00253 − CD_08_00495
CD_10_00327 − CD_08_00495
CD_10_00454 − CD_08_00495
CD_12_00008 − CD_08_00495
CD_12_00011 − CD_08_00495
CD_10_00253 − CD_10_00078
CD_10_00327 − CD_10_00078
CD_10_00454 − CD_10_00078
CD_12_00008 − CD_10_00078
CD_12_00011 − CD_10_00078
CD_10_00327 − CD_10_00253
CD_10_00454 − CD_10_00253
CD_12_00008 − CD_10_00253
CD_12_00011 − CD_10_00253
CD_10_00454 − CD_10_00327
CD_12_00008 − CD_10_00327
CD_12_00011 − CD_10_00327
CD_12_00008 − CD_10_00454
CD_12_00011 − CD_10_00454
CD_12_00011 − CD_12_00008
−5e+05
0e+00
5e+05
Linear Function

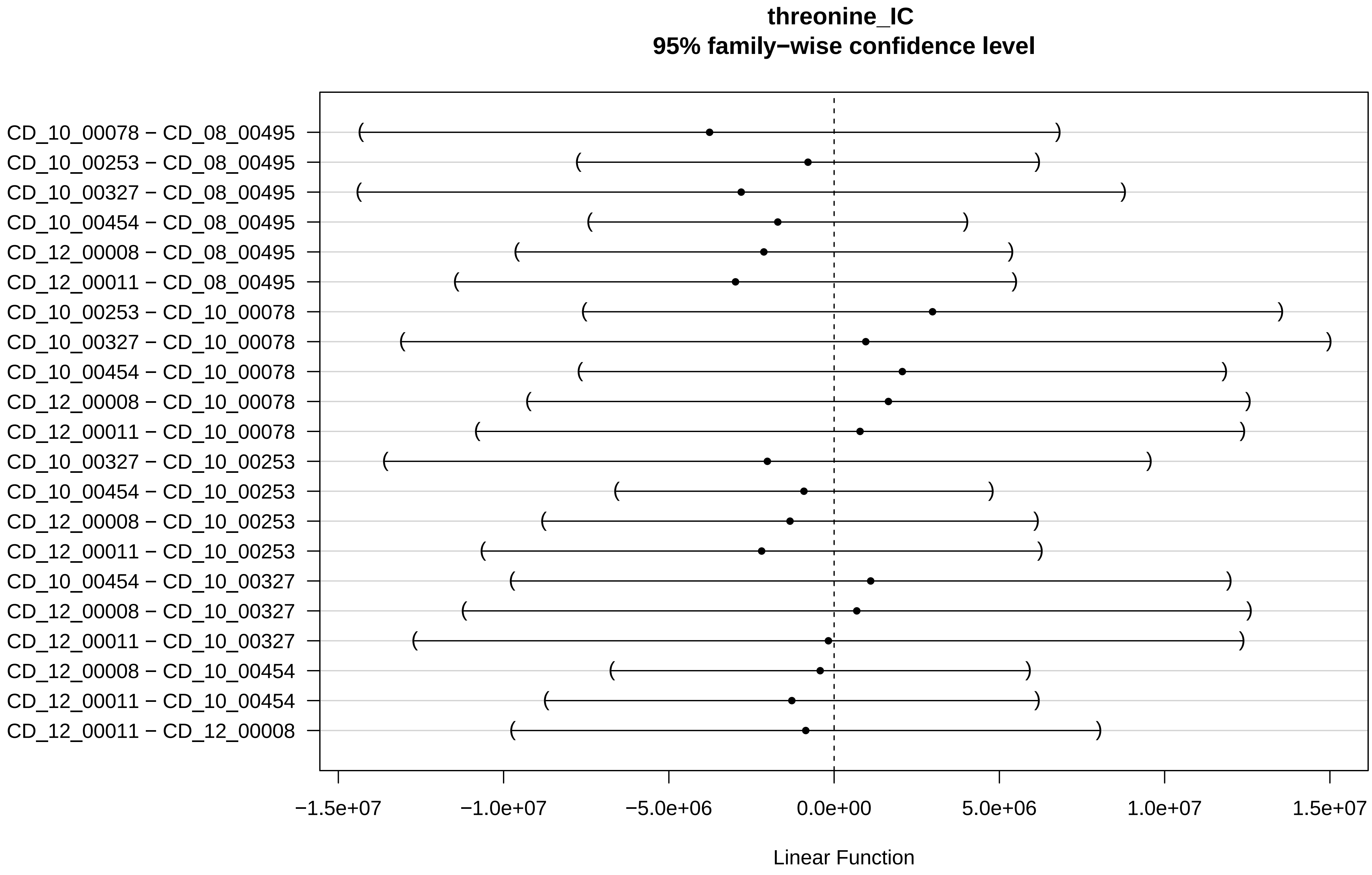
threonine_IC
95% family−wise confidence level
CD_10_00078 − CD_08_00495
CD_10_00253 − CD_08_00495
CD_10_00327 − CD_08_00495
CD_10_00454 − CD_08_00495
CD_12_00008 − CD_08_00495
CD_12_00011 − CD_08_00495
CD_10_00253 − CD_10_00078
CD_10_00327 − CD_10_00078
CD_10_00454 − CD_10_00078
CD_12_00008 − CD_10_00078
CD_12_00011 − CD_10_00078
CD_10_00327 − CD_10_00253
CD_10_00454 − CD_10_00253
CD_12_00008 − CD_10_00253
CD_12_00011 − CD_10_00253
CD_10_00454 − CD_10_00327
CD_12_00008 − CD_10_00327
CD_12_00011 − CD_10_00327
CD_12_00008 − CD_10_00454
CD_12_00011 − CD_10_00454
CD_12_00011 − CD_12_00008
−1.5e+07
−1.0e+07
−5.0e+06
0.0e+00
5.0e+06
1.0e+07
1.5e+07
Linear Function

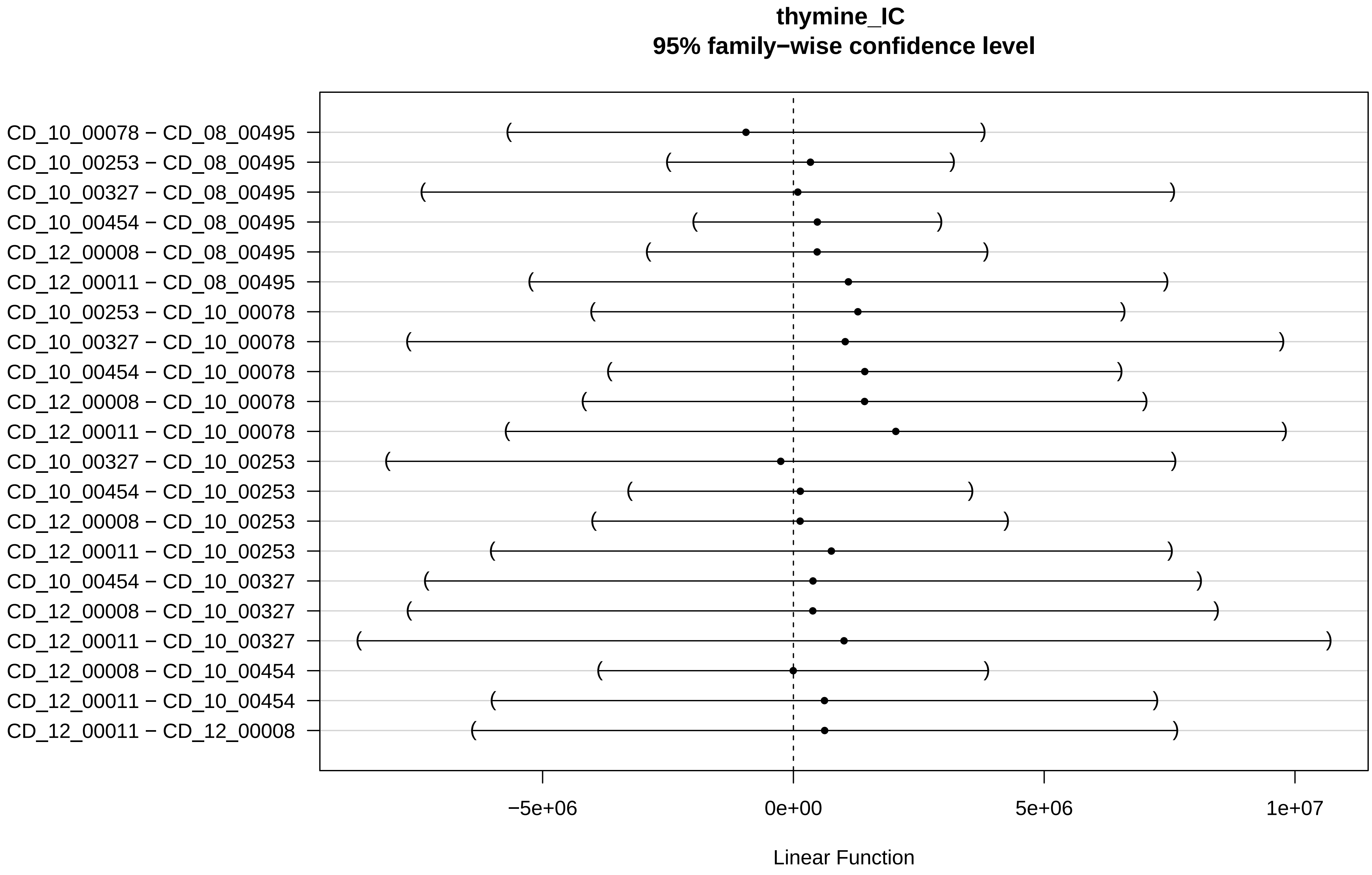
thymine_IC
95% family−wise confidence level
CD_10_00078 − CD_08_00495
CD_10_00253 − CD_08_00495
CD_10_00327 − CD_08_00495
CD_10_00454 − CD_08_00495
CD_12_00008 − CD_08_00495
CD_12_00011 − CD_08_00495
CD_10_00253 − CD_10_00078
CD_10_00327 − CD_10_00078
CD_10_00454 − CD_10_00078
CD_12_00008 − CD_10_00078
CD_12_00011 − CD_10_00078
CD_10_00327 − CD_10_00253
CD_10_00454 − CD_10_00253
CD_12_00008 − CD_10_00253
CD_12_00011 − CD_10_00253
CD_10_00454 − CD_10_00327
CD_12_00008 − CD_10_00327
CD_12_00011 − CD_10_00327
CD_12_00008 − CD_10_00454
CD_12_00011 − CD_10_00454
CD_12_00011 − CD_12_00008
−5e+06
0e+00
5e+06
1e+07
Linear Function

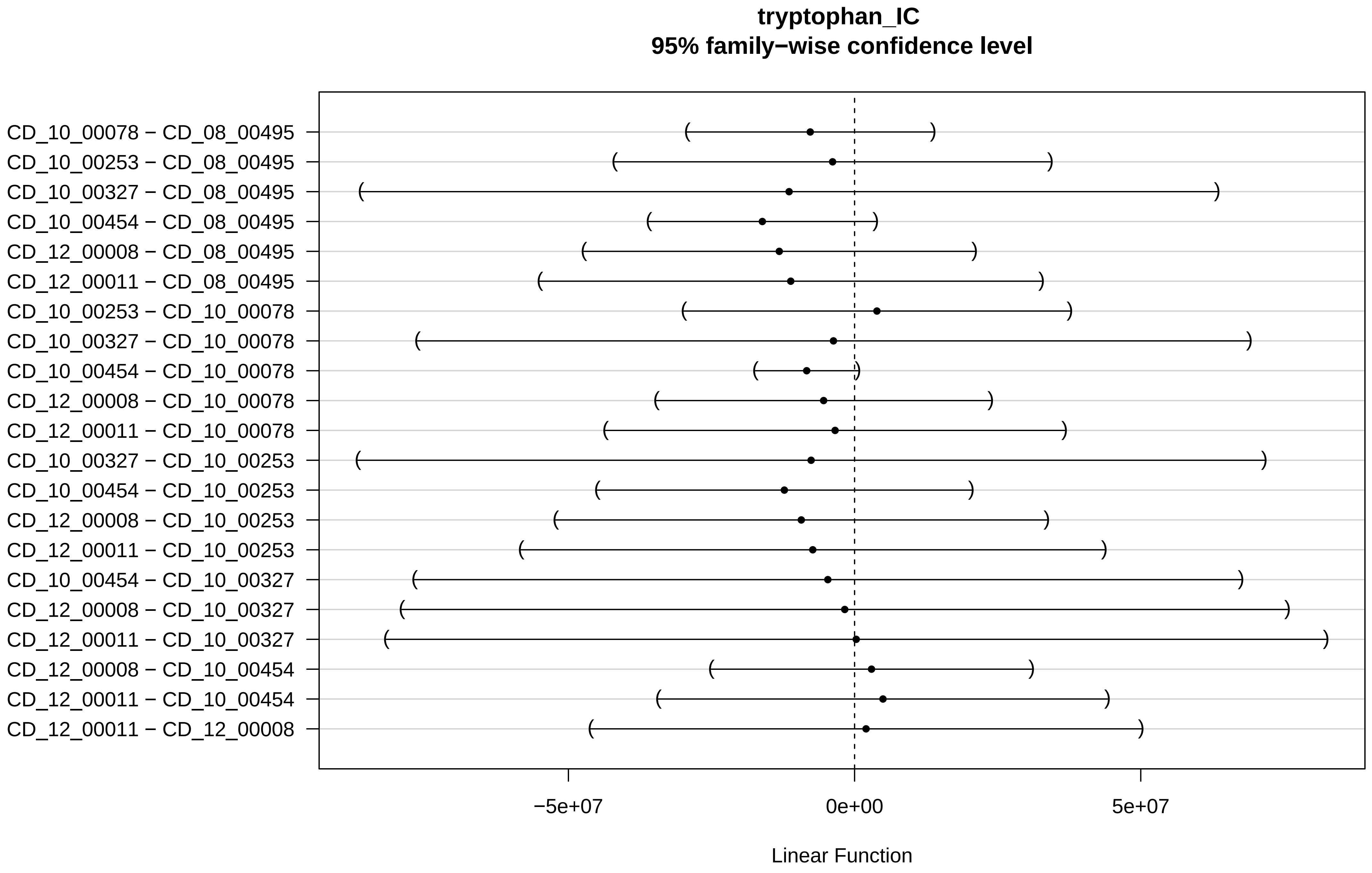

tryptophan_IC
95% family−wise confidence level
CD_10_00078 − CD_08_00495
CD_10_00253 − CD_08_00495
CD_10_00327 − CD_08_00495
CD_10_00454 − CD_08_00495
CD_12_00008 − CD_08_00495
CD_12_00011 − CD_08_00495
CD_10_00253 − CD_10_00078
CD_10_00327 − CD_10_00078
CD_10_00454 − CD_10_00078
CD_12_00008 − CD_10_00078
CD_12_00011 − CD_10_00078
CD_10_00327 − CD_10_00253
CD_10_00454 − CD_10_00253
CD_12_00008 − CD_10_00253
CD_12_00011 − CD_10_00253
CD_10_00454 − CD_10_00327
CD_12_00008 − CD_10_00327
CD_12_00011 − CD_10_00327
CD_12_00008 − CD_10_00454
CD_12_00011 − CD_10_00454
CD_12_00011 − CD_12_00008
−5e+07
0e+00
5e+07
Linear Function

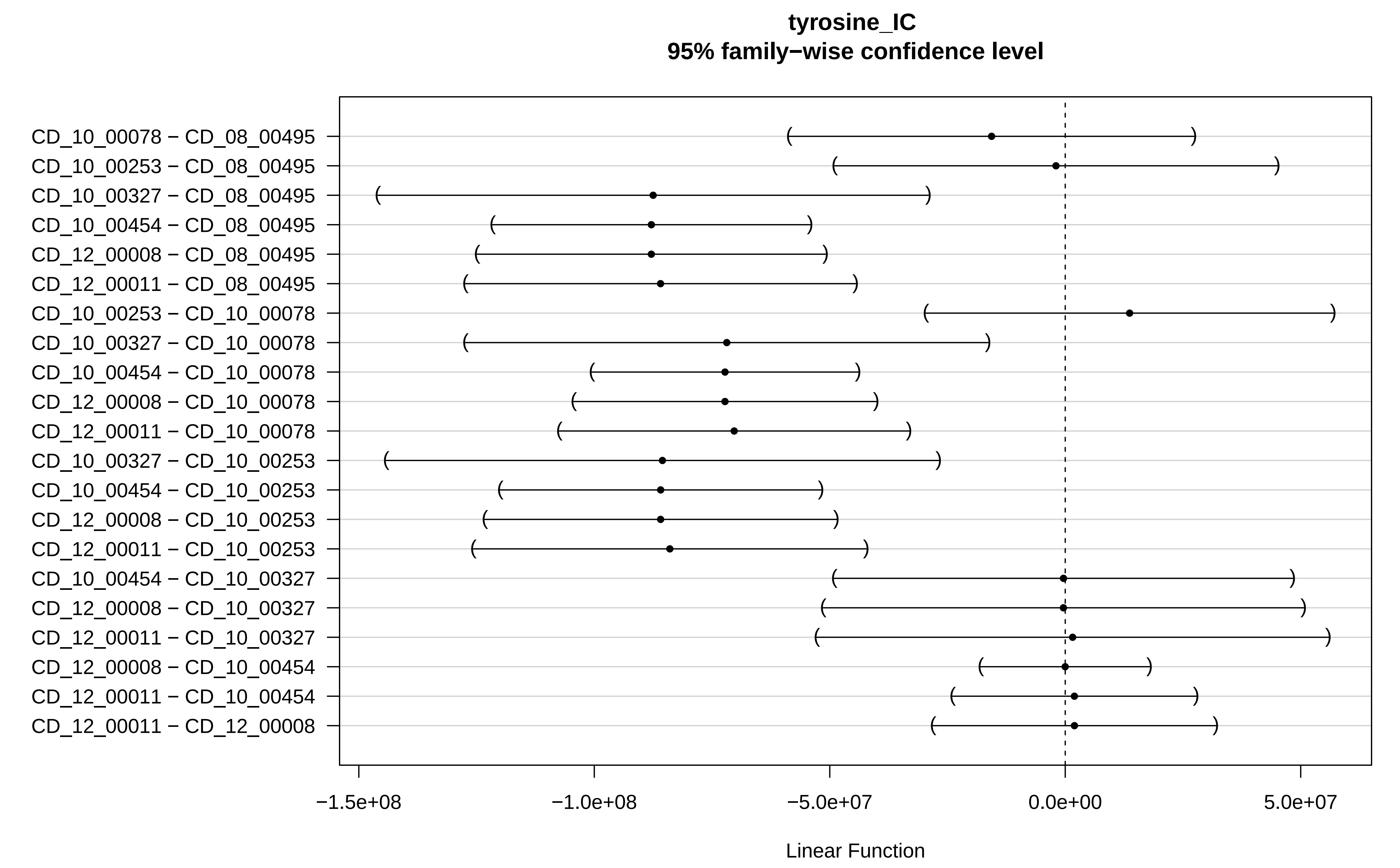
tyrosine_IC
95% family−wise confidence level
CD_10_00078 − CD_08_00495
CD_10_00253 − CD_08_00495
CD_10_00327 − CD_08_00495
CD_10_00454 − CD_08_00495
CD_12_00008 − CD_08_00495
CD_12_00011 − CD_08_00495
CD_10_00253 − CD_10_00078
CD_10_00327 − CD_10_00078
CD_10_00454 − CD_10_00078
CD_12_00008 − CD_10_00078
CD_12_00011 − CD_10_00078
CD_10_00327 − CD_10_00253
CD_10_00454 − CD_10_00253
CD_12_00008 − CD_10_00253
CD_12_00011 − CD_10_00253
CD_10_00454 − CD_10_00327
CD_12_00008 − CD_10_00327
CD_12_00011 − CD_10_00327
CD_12_00008 − CD_10_00454
CD_12_00011 − CD_10_00454
CD_12_00011 − CD_12_00008
−1.5e+08
−1.0e+08
−5.0e+07
0.0e+00
5.0e+07
Linear Function

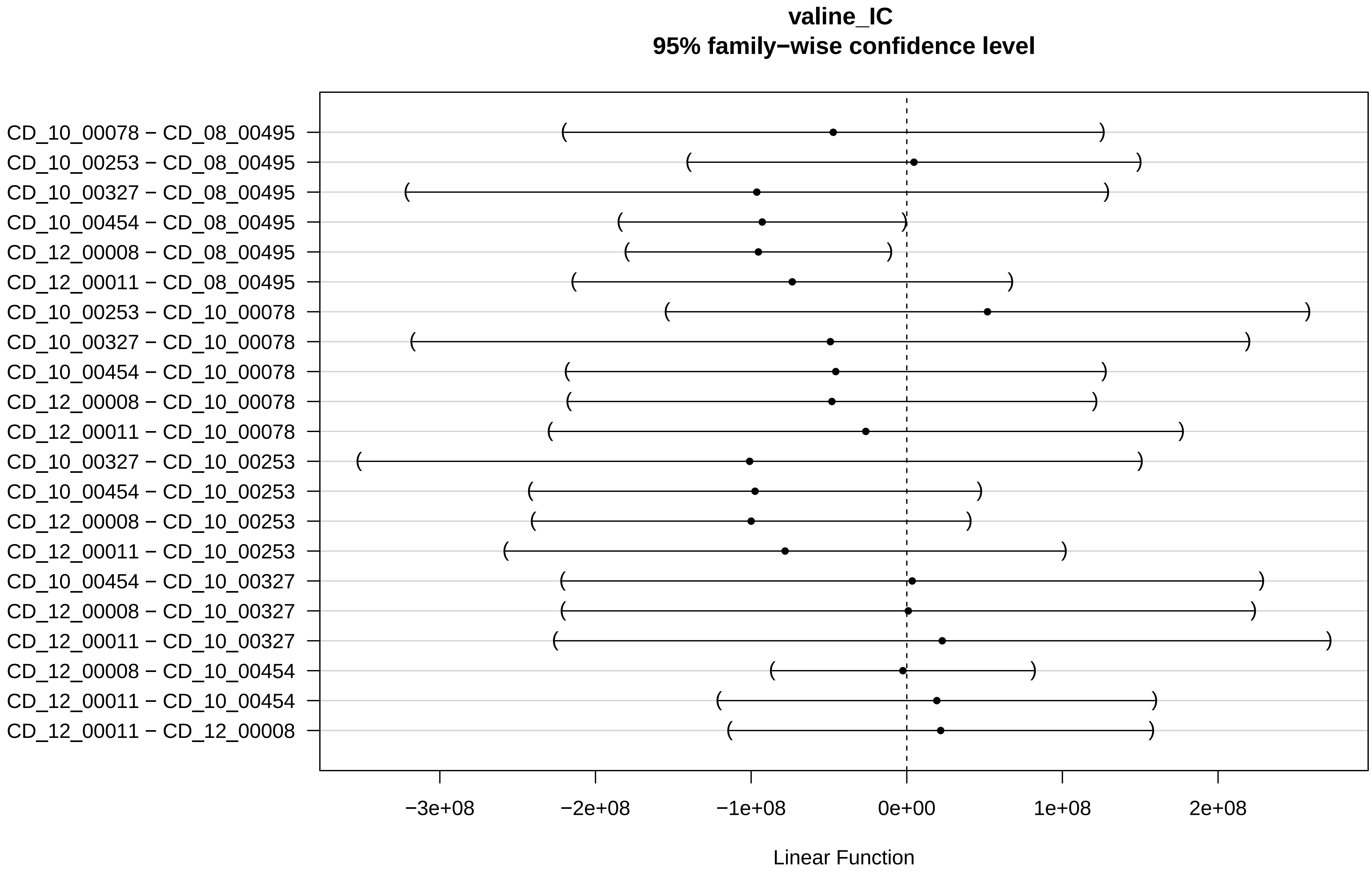
valine_IC
95% family−wise confidence level
CD_10_00078 − CD_08_00495
CD_10_00253 − CD_08_00495
CD_10_00327 − CD_08_00495
CD_10_00454 − CD_08_00495
CD_12_00008 − CD_08_00495
CD_12_00011 − CD_08_00495
CD_10_00253 − CD_10_00078
CD_10_00327 − CD_10_00078
CD_10_00454 − CD_10_00078
CD_12_00008 − CD_10_00078
CD_12_00011 − CD_10_00078
CD_10_00327 − CD_10_00253
CD_10_00454 − CD_10_00253
CD_12_00008 − CD_10_00253
CD_12_00011 − CD_10_00253
CD_10_00454 − CD_10_00327
CD_12_00008 − CD_10_00327
CD_12_00011 − CD_10_00327
CD_12_00008 − CD_10_00454
CD_12_00011 − CD_10_00454
CD_12_00011 − CD_12_00008
−3e+08
−2e+08
−1e+08
0e+00
1e+08
2e+08
Linear Function

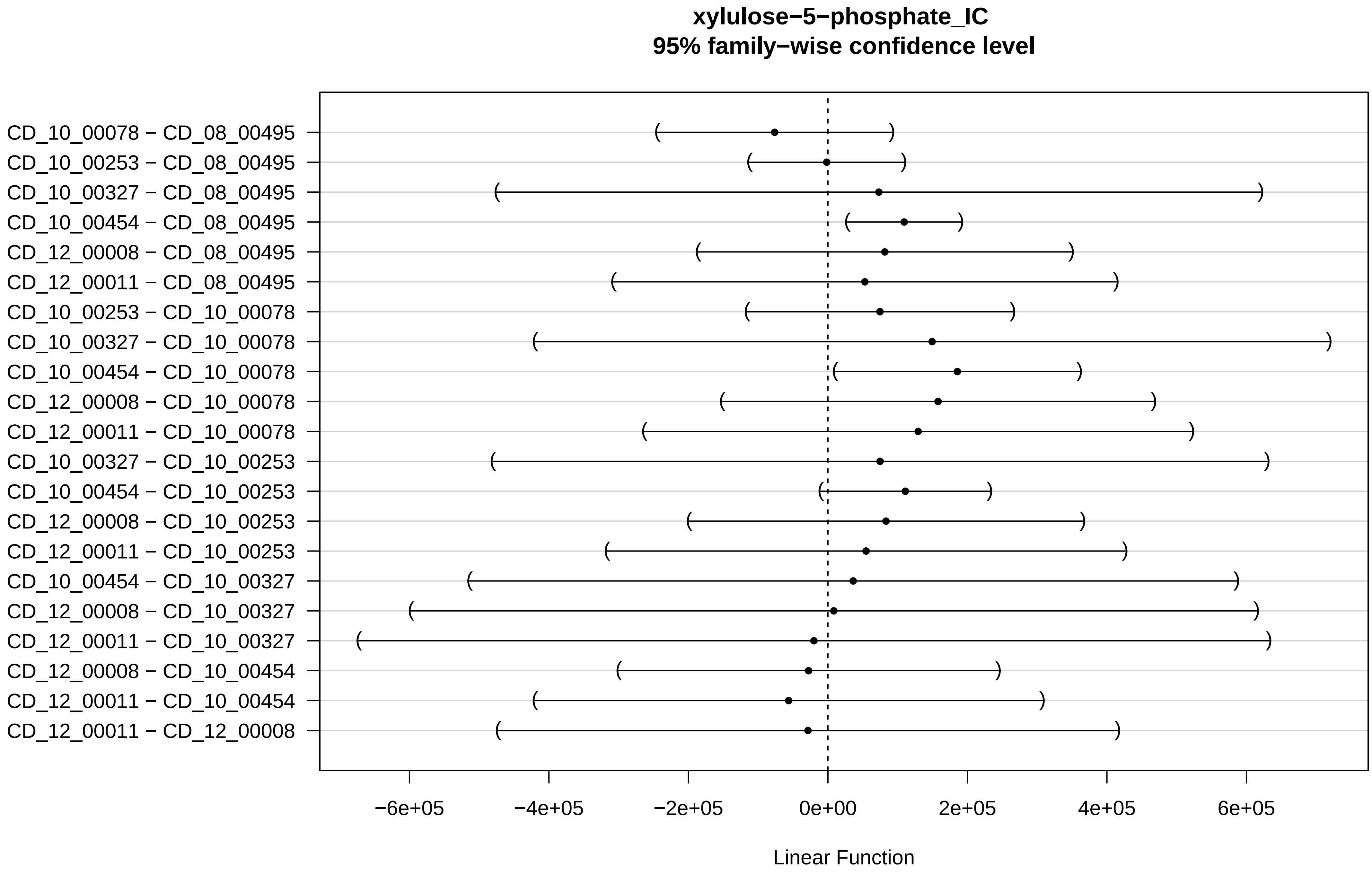

xylulose−5−phosphate_IC
95% family−wise confidence level
CD_10_00078 − CD_08_00495
CD_10_00253 − CD_08_00495
CD_10_00327 − CD_08_00495
CD_10_00454 − CD_08_00495
CD_12_00008 − CD_08_00495
CD_12_00011 − CD_08_00495
CD_10_00253 − CD_10_00078
CD_10_00327 − CD_10_00078
CD_10_00454 − CD_10_00078
CD_12_00008 − CD_10_00078
CD_12_00011 − CD_10_00078
CD_10_00327 − CD_10_00253
CD_10_00454 − CD_10_00253
CD_12_00008 − CD_10_00253
CD_12_00011 − CD_10_00253
CD_10_00454 − CD_10_00327
CD_12_00008 − CD_10_00327
CD_12_00011 − CD_10_00327
CD_12_00008 − CD_10_00454
CD_12_00011 − CD_10_00454
CD_12_00011 − CD_12_00008
−6e+05
−4e+05
−2e+05
0e+00
2e+05
4e+05
6e+05
Linear Function

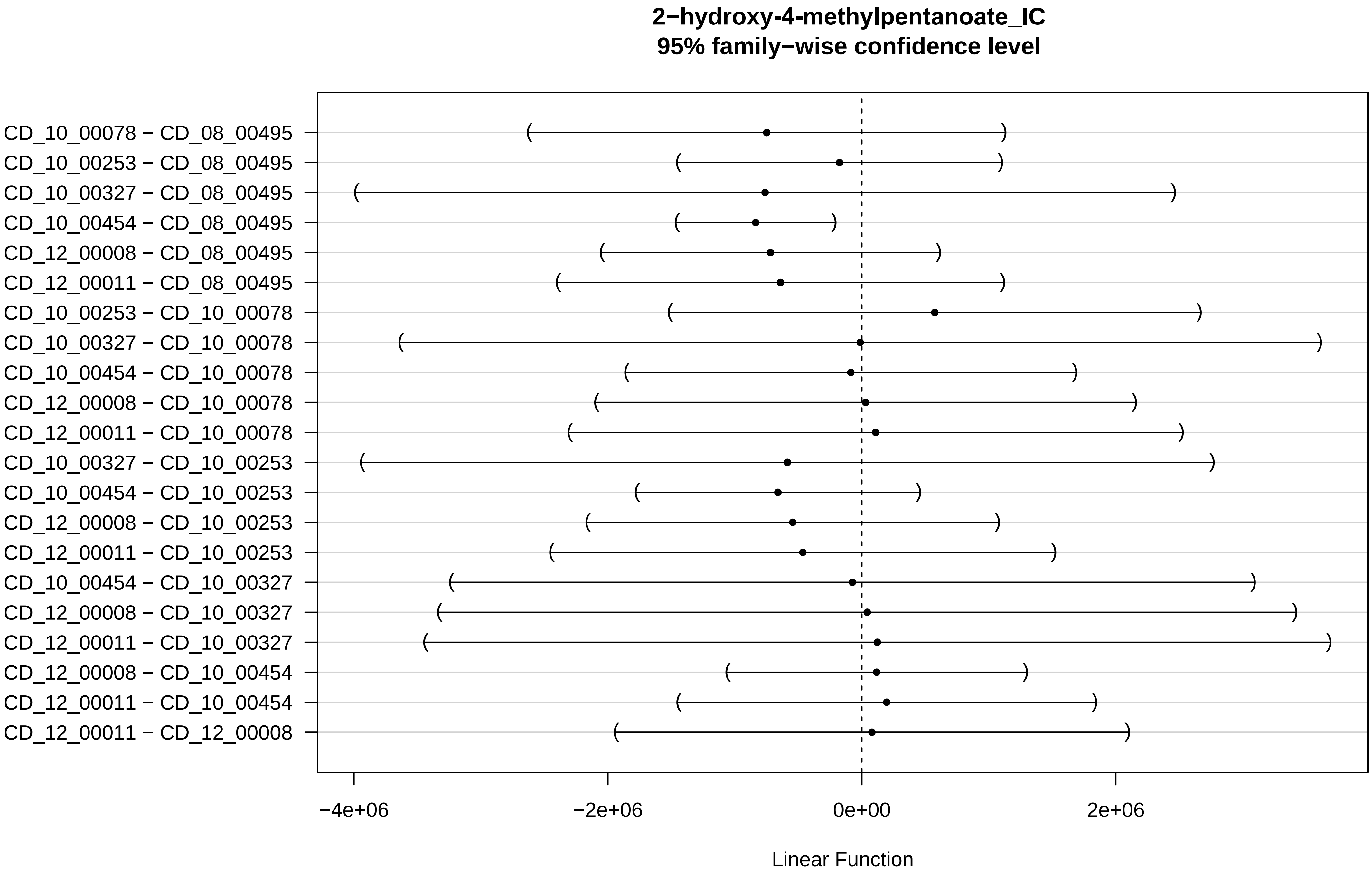
2−hydroxy-4-methylpentanoate_IC
95% family−wise confidence level
CD_10_00078 − CD_08_00495
CD_10_00253 − CD_08_00495
CD_10_00327 − CD_08_00495
CD_10_00454 − CD_08_00495
CD_12_00008 − CD_08_00495
CD_12_00011 − CD_08_00495
CD_10_00253 − CD_10_00078
CD_10_00327 − CD_10_00078
CD_10_00454 − CD_10_00078
CD_12_00008 − CD_10_00078
CD_12_00011 − CD_10_00078
CD_10_00327 − CD_10_00253
CD_10_00454 − CD_10_00253
CD_12_00008 − CD_10_00253
CD_12_00011 − CD_10_00253
CD_10_00454 − CD_10_00327
CD_12_00008 − CD_10_00327
CD_12_00011 − CD_10_00327
CD_12_00008 − CD_10_00454
CD_12_00011 − CD_10_00454
CD_12_00011 − CD_12_00008
−4e+06
−2e+06
0e+00
2e+06
Linear Function

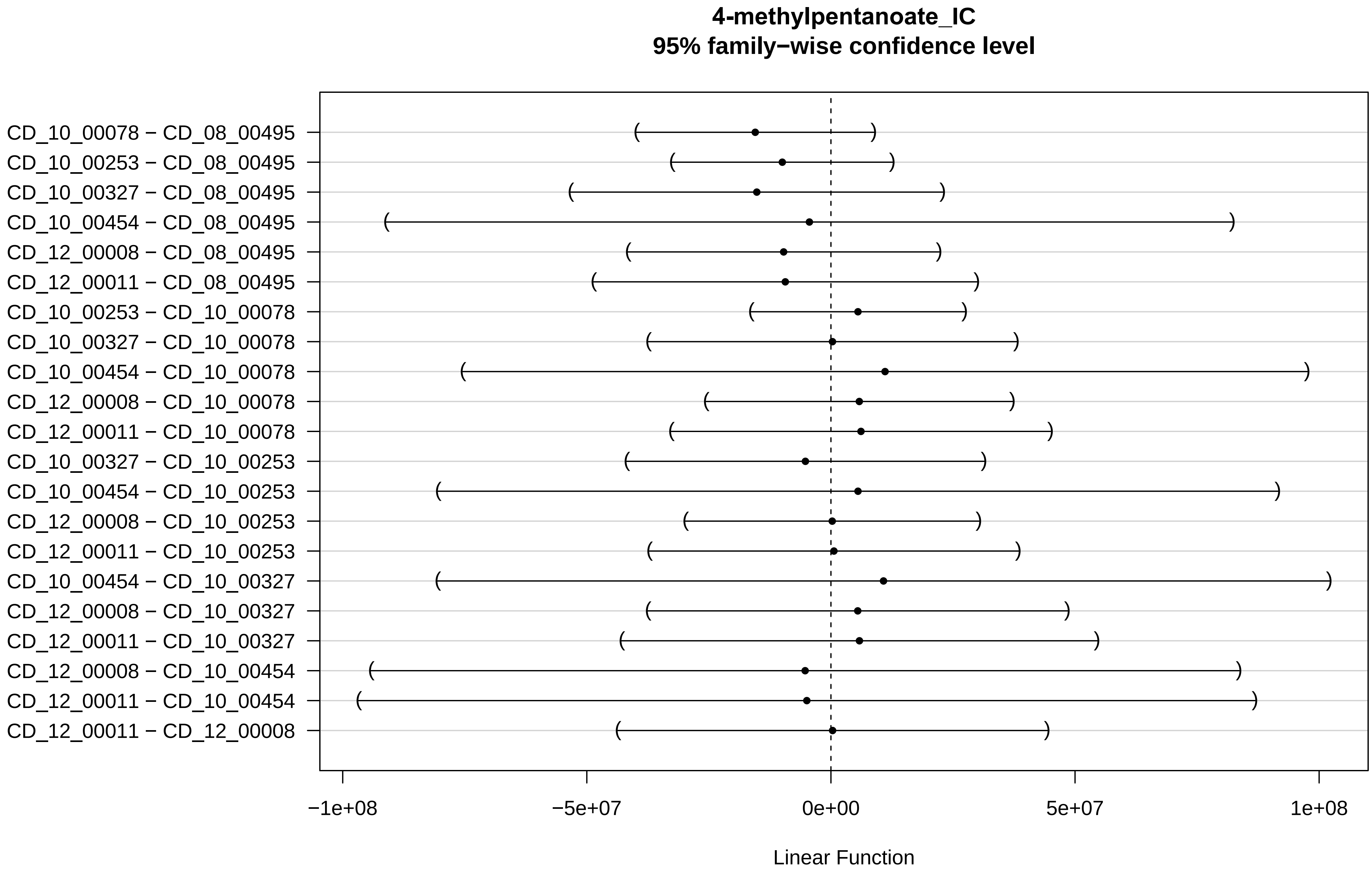
4-methylpentanoate_IC
95% family−wise confidence level
CD_10_00078 − CD_08_00495
CD_10_00253 − CD_08_00495
CD_10_00327 − CD_08_00495
CD_10_00454 − CD_08_00495
CD_12_00008 − CD_08_00495
CD_12_00011 − CD_08_00495
CD_10_00253 − CD_10_00078
CD_10_00327 − CD_10_00078
CD_10_00454 − CD_10_00078
CD_12_00008 − CD_10_00078
CD_12_00011 − CD_10_00078
CD_10_00327 − CD_10_00253
CD_10_00454 − CD_10_00253
CD_12_00008 − CD_10_00253
CD_12_00011 − CD_10_00253
CD_10_00454 − CD_10_00327
CD_12_00008 − CD_10_00327
CD_12_00011 − CD_10_00327
CD_12_00008 − CD_10_00454
CD_12_00011 − CD_10_00454
CD_12_00011 − CD_12_00008
−1e+08
−5e+07
0e+00
5e+07
1e+08
Linear Function

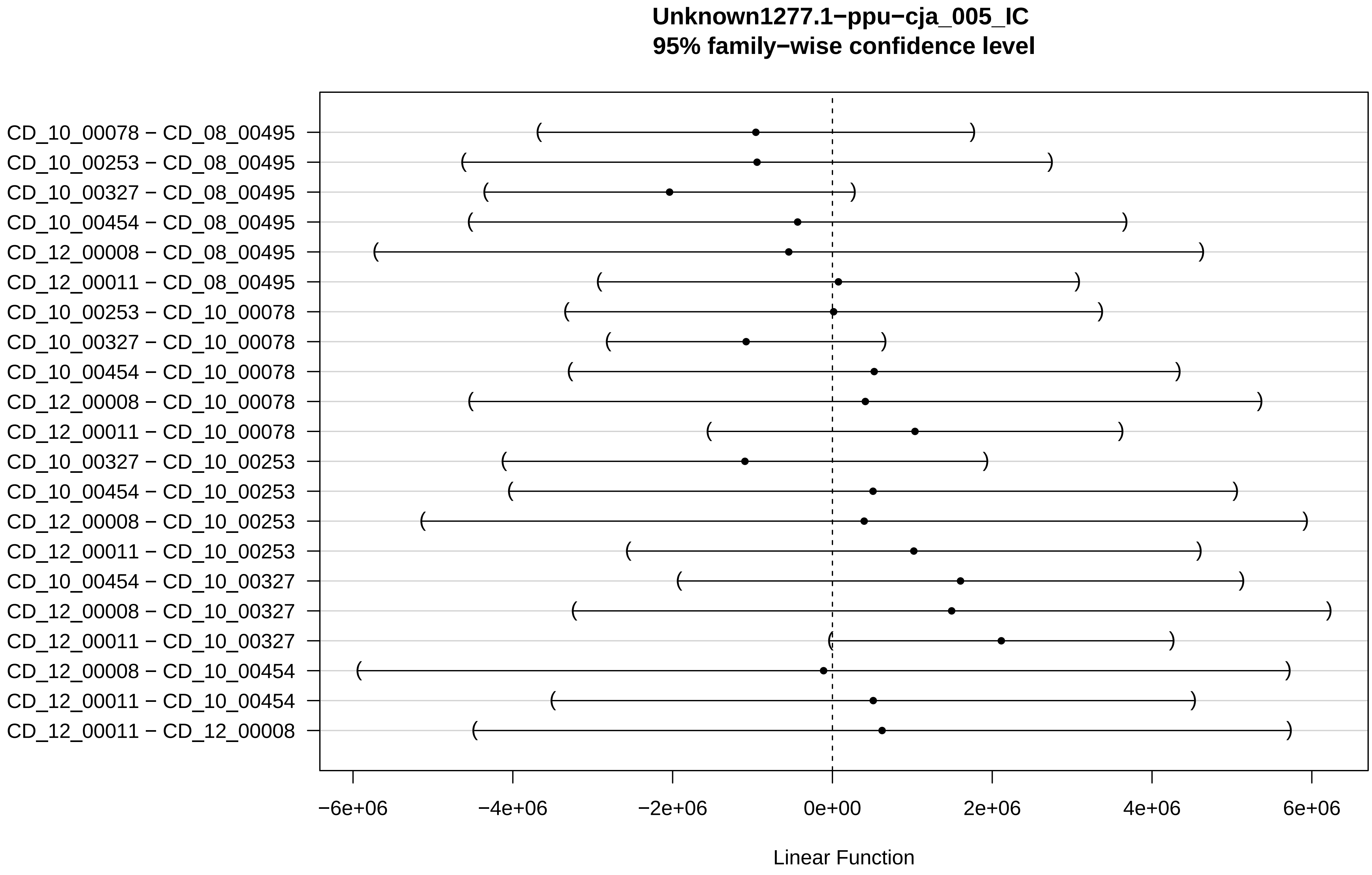
Unknown1277.1−ppu−cja_005_IC
95% family−wise confidence level
CD_10_00078 − CD_08_00495
CD_10_00253 − CD_08_00495
CD_10_00327 − CD_08_00495
CD_10_00454 − CD_08_00495
CD_12_00008 − CD_08_00495
CD_12_00011 − CD_08_00495
CD_10_00253 − CD_10_00078
CD_10_00327 − CD_10_00078
CD_10_00454 − CD_10_00078
CD_12_00008 − CD_10_00078
CD_12_00011 − CD_10_00078
CD_10_00327 − CD_10_00253
CD_10_00454 − CD_10_00253
CD_12_00008 − CD_10_00253
CD_12_00011 − CD_10_00253
CD_10_00454 − CD_10_00327
CD_12_00008 − CD_10_00327
CD_12_00011 − CD_10_00327
CD_12_00008 − CD_10_00454
CD_12_00011 − CD_10_00454
CD_12_00011 − CD_12_00008
−6e+06
−4e+06
−2e+06
0e+00
2e+06
4e+06
6e+06
Linear Function

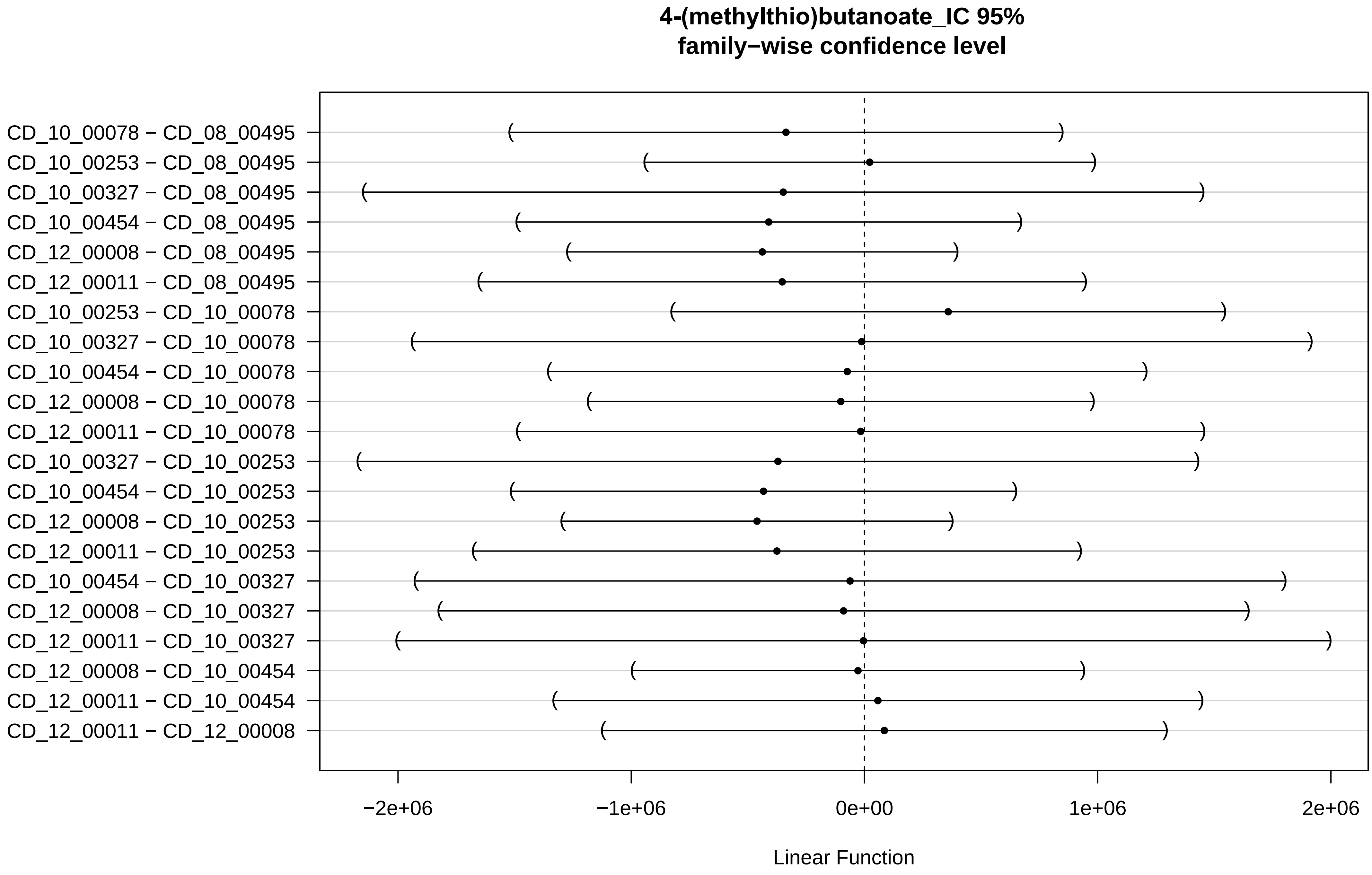
4-(methylthio)butanoate_IC 95%
family−wise confidence level
CD_10_00078 − CD_08_00495
CD_10_00253 − CD_08_00495
CD_10_00327 − CD_08_00495
CD_10_00454 − CD_08_00495
CD_12_00008 − CD_08_00495
CD_12_00011 − CD_08_00495
CD_10_00253 − CD_10_00078
CD_10_00327 − CD_10_00078
CD_10_00454 − CD_10_00078
CD_12_00008 − CD_10_00078
CD_12_00011 − CD_10_00078
CD_10_00327 − CD_10_00253
CD_10_00454 − CD_10_00253
CD_12_00008 − CD_10_00253
CD_12_00011 − CD_10_00253
CD_10_00454 − CD_10_00327
CD_12_00008 − CD_10_00327
CD_12_00011 − CD_10_00327
CD_12_00008 − CD_10_00454
CD_12_00011 − CD_10_00454
CD_12_00011 − CD_12_00008
−2e+06
−1e+06
0e+00
1e+06
2e+06
Linear Function

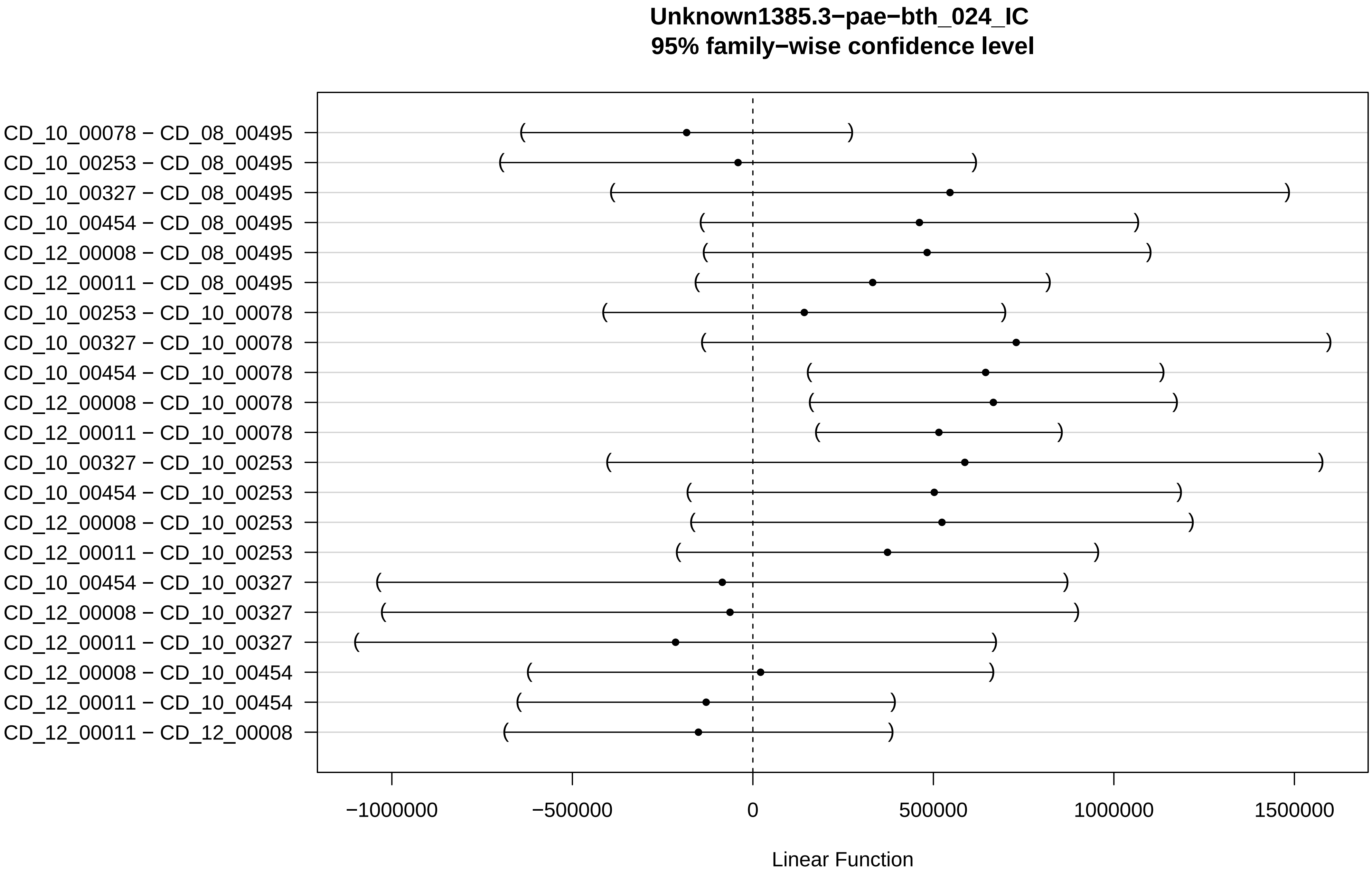

Unknown1385.3−pae−bth_024_IC
95% family−wise confidence level
CD_10_00078 − CD_08_00495
CD_10_00253 − CD_08_00495
CD_10_00327 − CD_08_00495
CD_10_00454 − CD_08_00495
CD_12_00008 − CD_08_00495
CD_12_00011 − CD_08_00495
CD_10_00253 − CD_10_00078
CD_10_00327 − CD_10_00078
CD_10_00454 − CD_10_00078
CD_12_00008 − CD_10_00078
CD_12_00011 − CD_10_00078
CD_10_00327 − CD_10_00253
CD_10_00454 − CD_10_00253
CD_12_00008 − CD_10_00253
CD_12_00011 − CD_10_00253
CD_10_00454 − CD_10_00327
CD_12_00008 − CD_10_00327
CD_12_00011 − CD_10_00327
CD_12_00008 − CD_10_00454
CD_12_00011 − CD_10_00454
CD_12_00011 − CD_12_00008
−1000000
−500000
0
500000
1000000
1500000
Linear Function

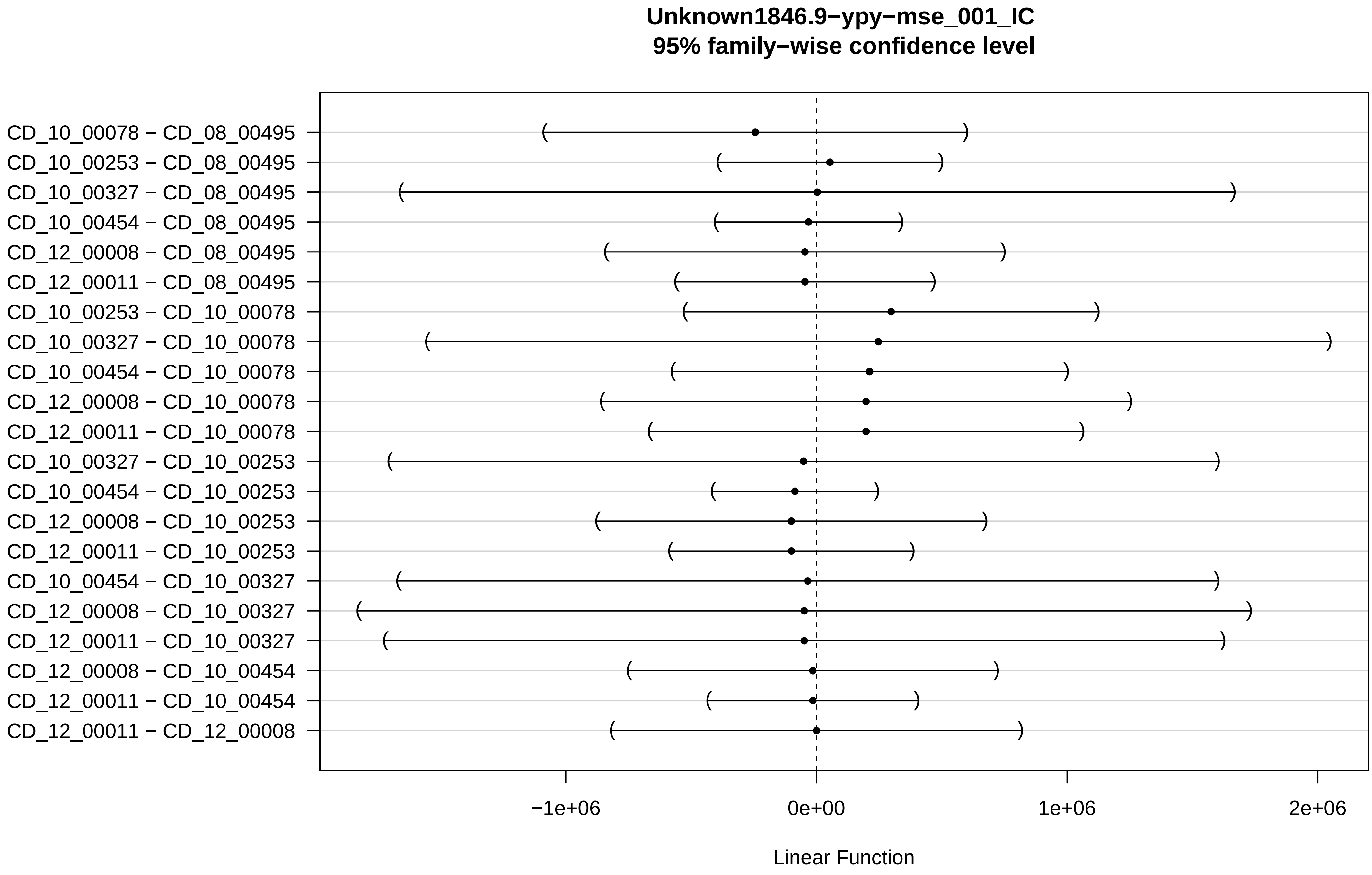
Unknown1846.9−ypy−mse_001_IC
95% family−wise confidence level
CD_10_00078 − CD_08_00495
CD_10_00253 − CD_08_00495
CD_10_00327 − CD_08_00495
CD_10_00454 − CD_08_00495
CD_12_00008 − CD_08_00495
CD_12_00011 − CD_08_00495
CD_10_00253 − CD_10_00078
CD_10_00327 − CD_10_00078
CD_10_00454 − CD_10_00078
CD_12_00008 − CD_10_00078
CD_12_00011 − CD_10_00078
CD_10_00327 − CD_10_00253
CD_10_00454 − CD_10_00253
CD_12_00008 − CD_10_00253
CD_12_00011 − CD_10_00253
CD_10_00454 − CD_10_00327
CD_12_00008 − CD_10_00327
CD_12_00011 − CD_10_00327
CD_12_00008 − CD_10_00454
CD_12_00011 − CD_10_00454
CD_12_00011 − CD_12_00008
−1e+06
0e+00
1e+06
2e+06
Linear Function

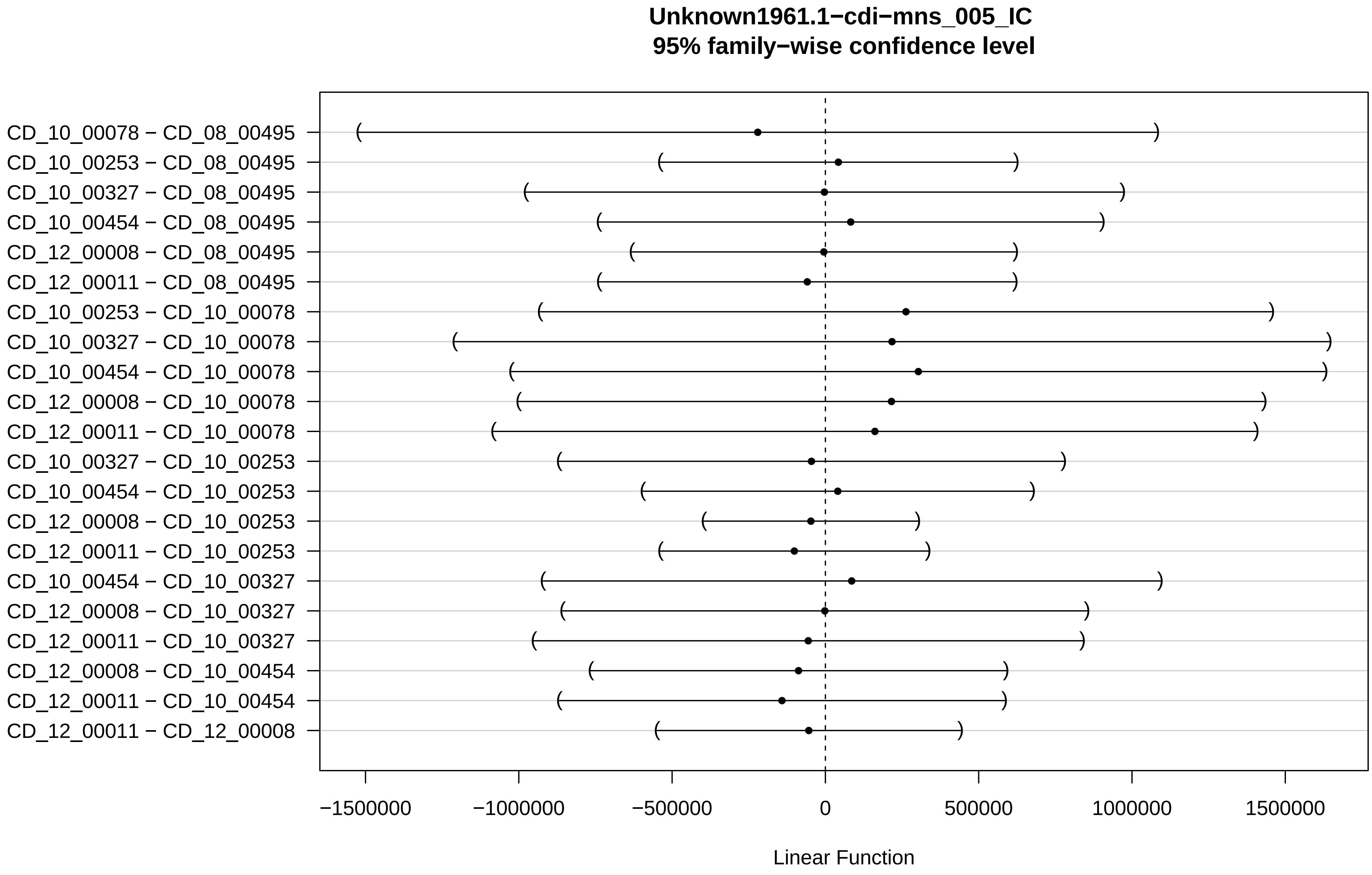
Unknown1961.1−cdi−mns_005_IC
95% family−wise confidence level
CD_10_00078 − CD_08_00495
CD_10_00253 − CD_08_00495
CD_10_00327 − CD_08_00495
CD_10_00454 − CD_08_00495
CD_12_00008 − CD_08_00495
CD_12_00011 − CD_08_00495
CD_10_00253 − CD_10_00078
CD_10_00327 − CD_10_00078
CD_10_00454 − CD_10_00078
CD_12_00008 − CD_10_00078
CD_12_00011 − CD_10_00078
CD_10_00327 − CD_10_00253
CD_10_00454 − CD_10_00253
CD_12_00008 − CD_10_00253
CD_12_00011 − CD_10_00253
CD_10_00454 − CD_10_00327
CD_12_00008 − CD_10_00327
CD_12_00011 − CD_10_00327
CD_12_00008 − CD_10_00454
CD_12_00011 − CD_10_00454
CD_12_00011 − CD_12_00008
−1500000
−1000000
−500000
0
500000
1000000
1500000
Linear Function

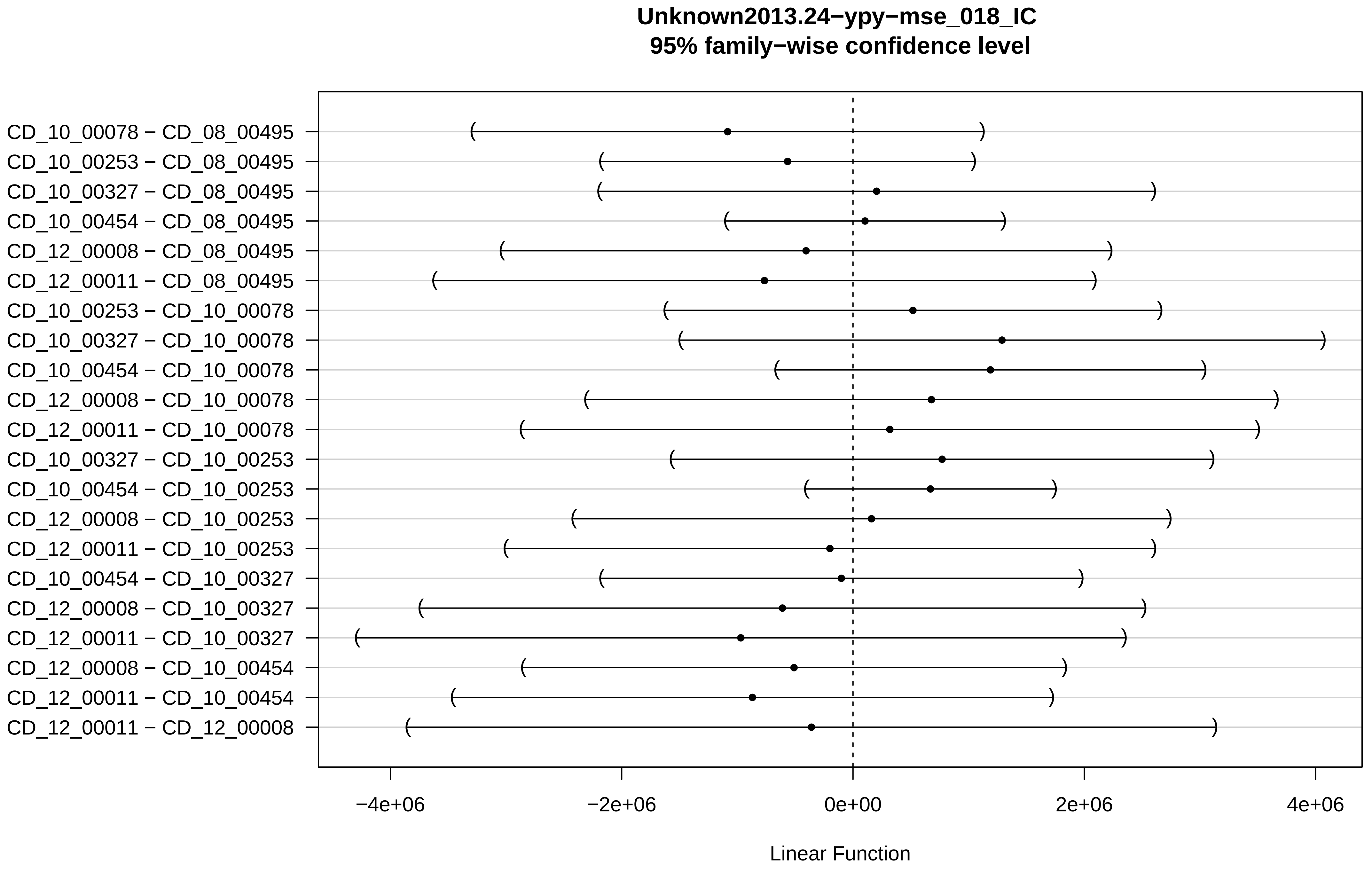
Unknown2013.24−ypy−mse_018_IC
95% family−wise confidence level
CD_10_00078 − CD_08_00495
CD_10_00253 − CD_08_00495
CD_10_00327 − CD_08_00495
CD_10_00454 − CD_08_00495
CD_12_00008 − CD_08_00495
CD_12_00011 − CD_08_00495
CD_10_00253 − CD_10_00078
CD_10_00327 − CD_10_00078
CD_10_00454 − CD_10_00078
CD_12_00008 − CD_10_00078
CD_12_00011 − CD_10_00078
CD_10_00327 − CD_10_00253
CD_10_00454 − CD_10_00253
CD_12_00008 − CD_10_00253
CD_12_00011 − CD_10_00253
CD_10_00454 − CD_10_00327
CD_12_00008 − CD_10_00327
CD_12_00011 − CD_10_00327
CD_12_00008 − CD_10_00454
CD_12_00011 − CD_10_00454
CD_12_00011 − CD_12_00008
−4e+06
−2e+06
0e+00
2e+06
4e+06
Linear Function

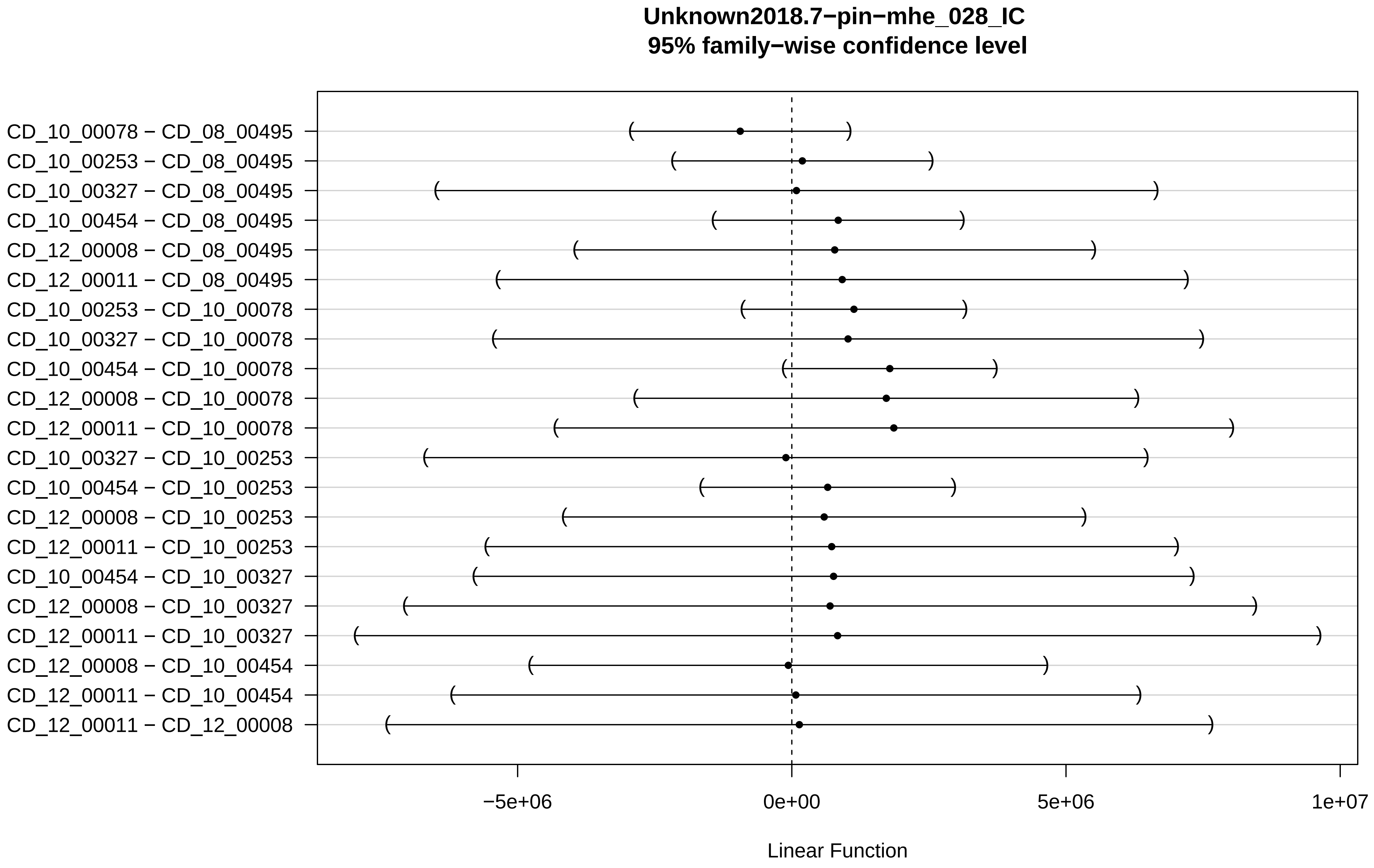
Unknown2018.7−pin−mhe_028_IC
95% family−wise confidence level
CD_10_00078 − CD_08_00495
CD_10_00253 − CD_08_00495
CD_10_00327 − CD_08_00495
CD_10_00454 − CD_08_00495
CD_12_00008 − CD_08_00495
CD_12_00011 − CD_08_00495
CD_10_00253 − CD_10_00078
CD_10_00327 − CD_10_00078
CD_10_00454 − CD_10_00078
CD_12_00008 − CD_10_00078
CD_12_00011 − CD_10_00078
CD_10_00327 − CD_10_00253
CD_10_00454 − CD_10_00253
CD_12_00008 − CD_10_00253
CD_12_00011 − CD_10_00253
CD_10_00454 − CD_10_00327
CD_12_00008 − CD_10_00327
CD_12_00011 − CD_10_00327
CD_12_00008 − CD_10_00454
CD_12_00011 − CD_10_00454
CD_12_00011 − CD_12_00008
−5e+06
0e+00
5e+06
1e+07
Linear Function
