# Supplementary figures and images for: Convergent Loss of ABC Transporter Genes From Clostridioides difficile Genomes Is Associated With Impaired Tyrosine Uptake and p-Cresol Production
Source: Front Microbiol. 2018 May 8;9:901. doi: 10.3389/fmicb.2018.00901 (PMC5951980; doi:10.3389/fmicb.2018.00901)

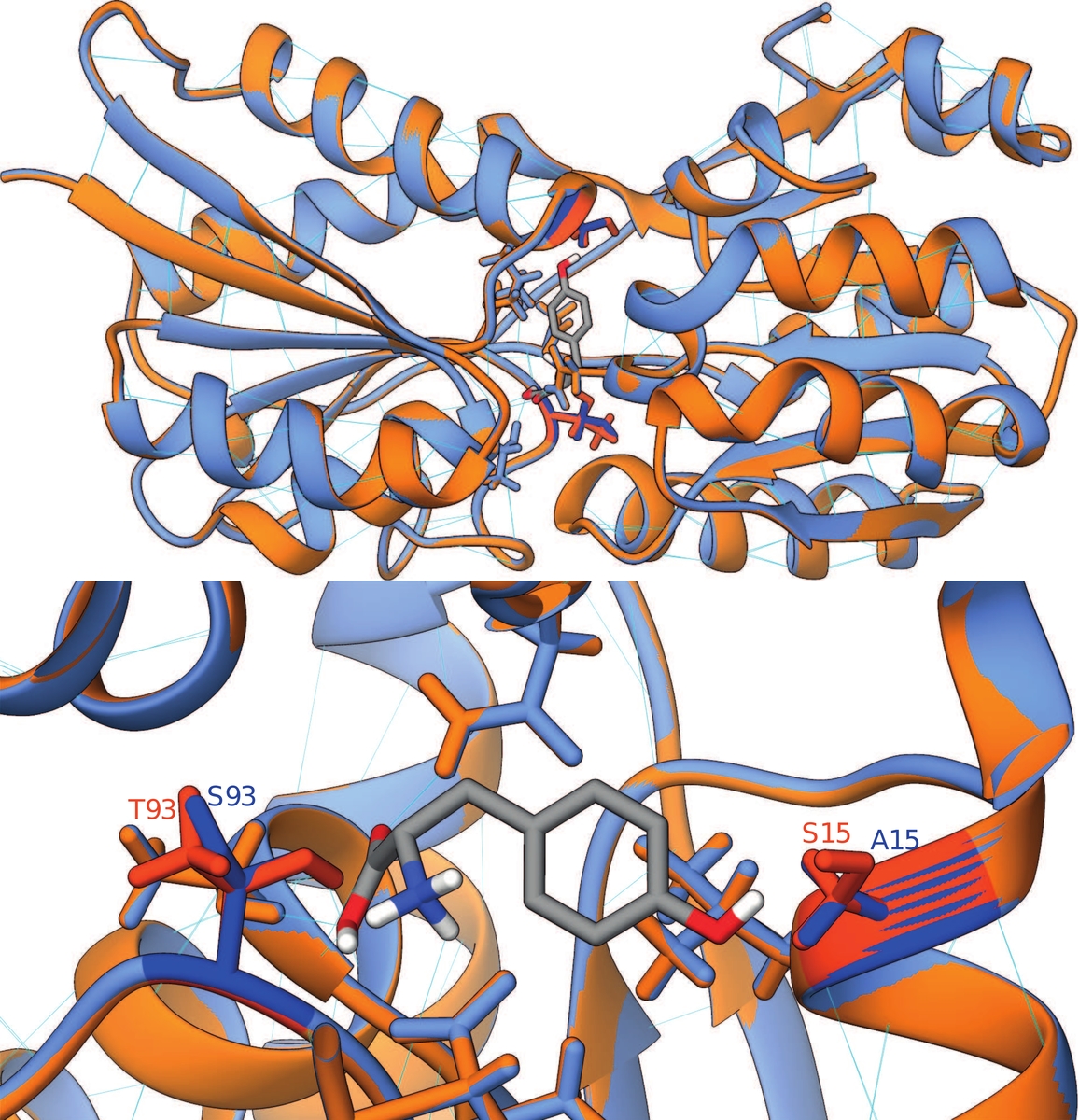

Supplement: FIGURE S2 — Modeled tertiary structures of the substrate-binding proteins encoded by genes CDR20291_0805 (orange) and CDR20291_0802 (blue), with L-tyrosine as ligand. The substrate-binding site is enlarged in the lower panel. Labeled amino acids differ between the two proteins and may affect their differential substrate specificity. [file Image_2.JPEG]

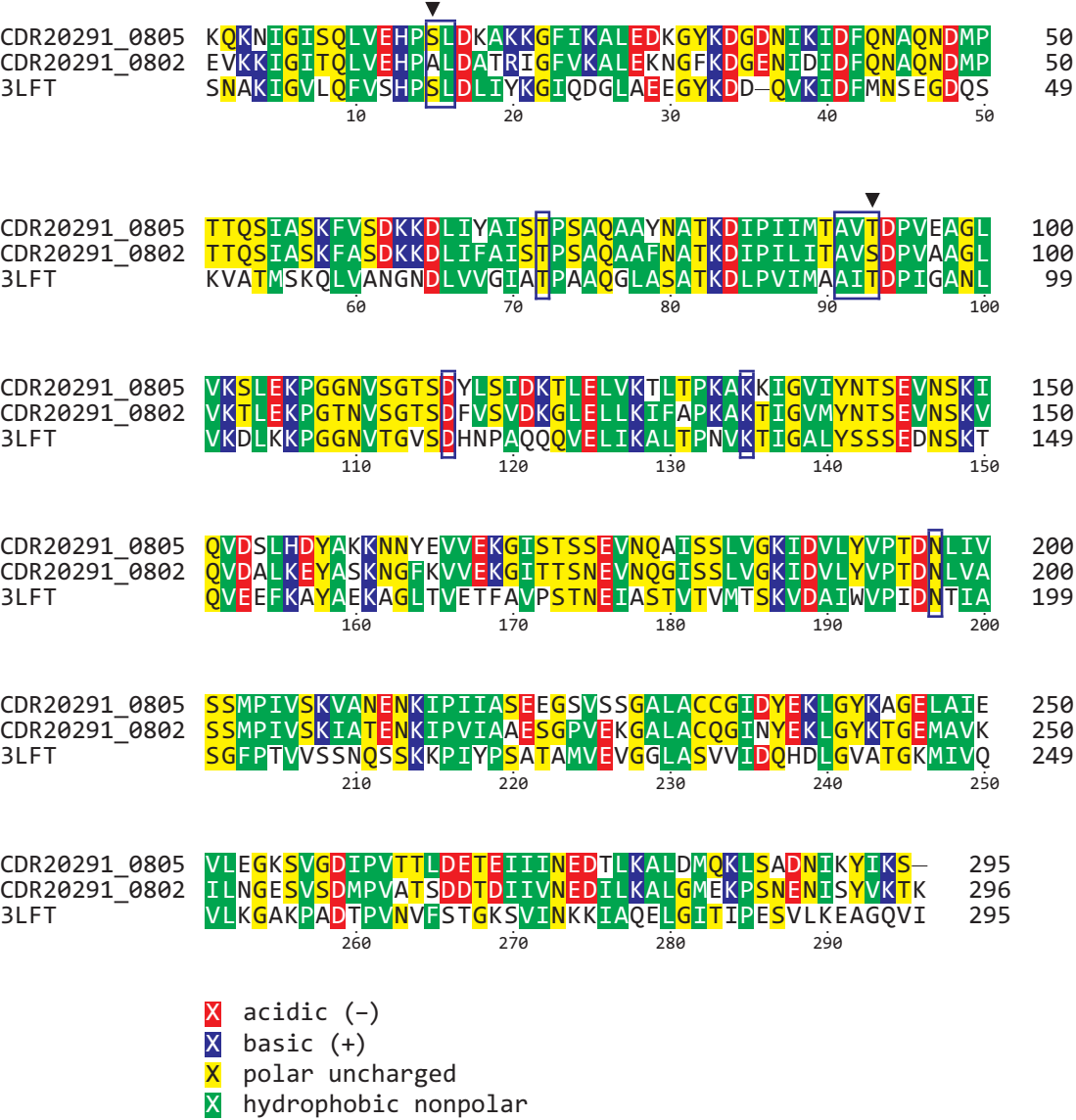

Supplement: FIGURE S3 — Alignment of the proteins encoded by C. difficile genes CDR20291_0805 and CDR20291_0802 with the substrate-binding protein from Streptococcus pneumoniae (PDB-ID, 3LFT). Shading colors indicate the hydropathy of each amino acid. Blue boxes highlight the amino acids that build the substrate-binding site. Black triangles indicate differences among amino acids within the substrate-binding site. [file Image_3.PDF]

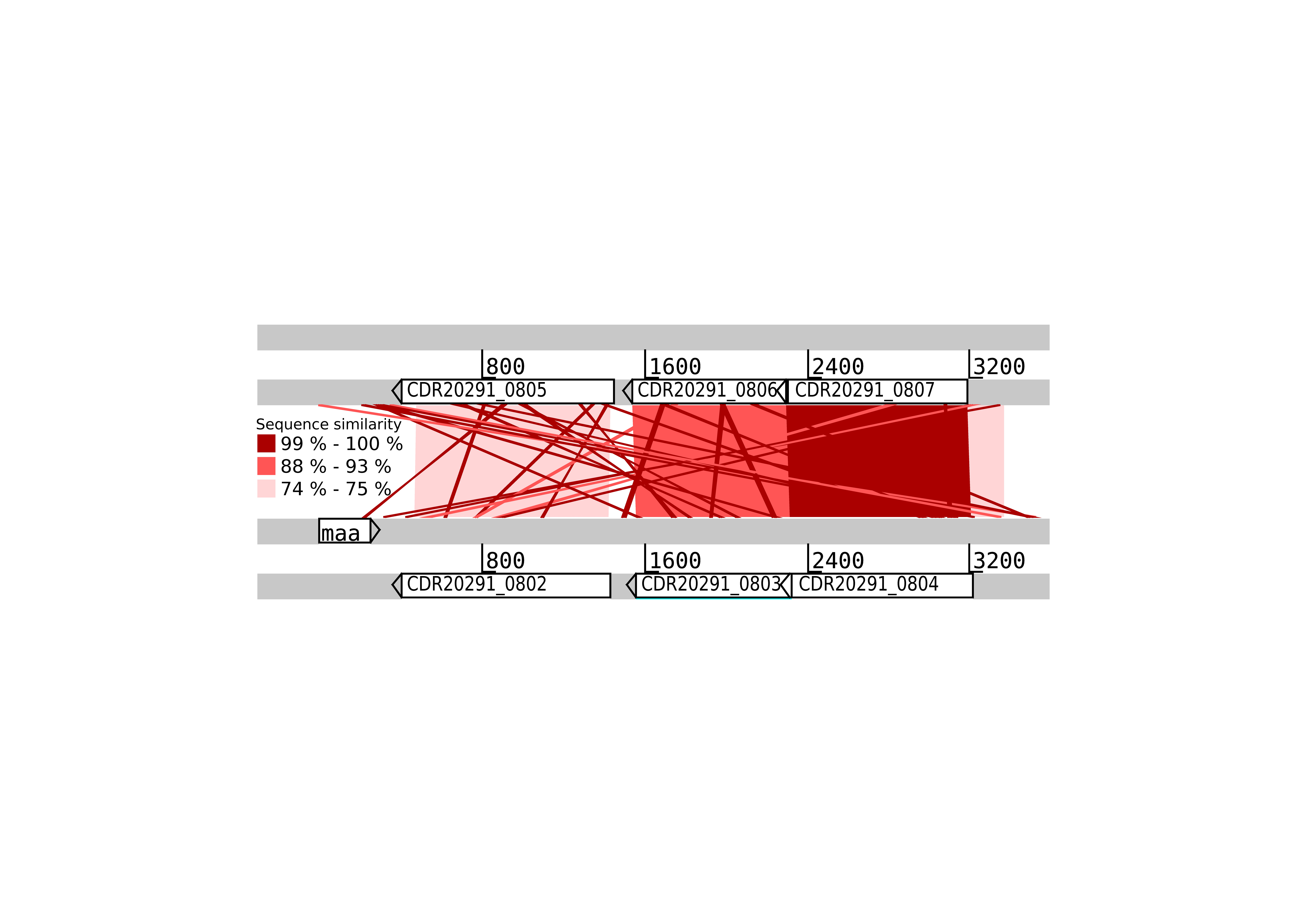

Supplement: FIGURE S4 — Alignment of genes CDR20291_0802 to CDR20291_0804 vs. CDR20291_0805 to CDR20291_0807, respectively, indicating sequence similarities. [file Image_4.JPEG]

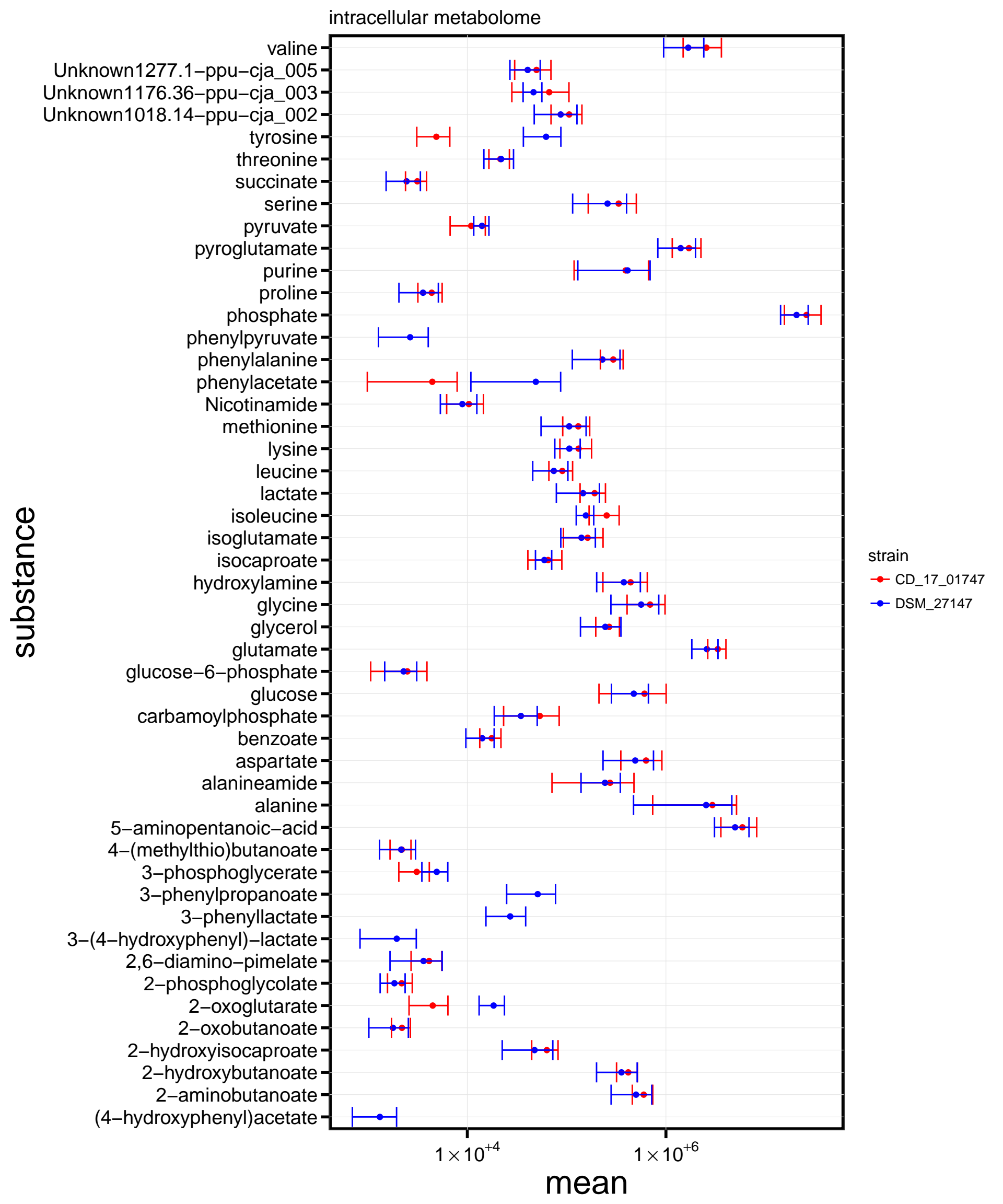

Supplement: FIGURE S5 — A comparison of intracellular metabolite profiles from isolates CD-17-01474 and DSM 27147. Mean normalized peak areas and 95% confidence intervals are indicated. Some metabolites were not detected for isolate CD-17-01474. [file Image_5.PDF]

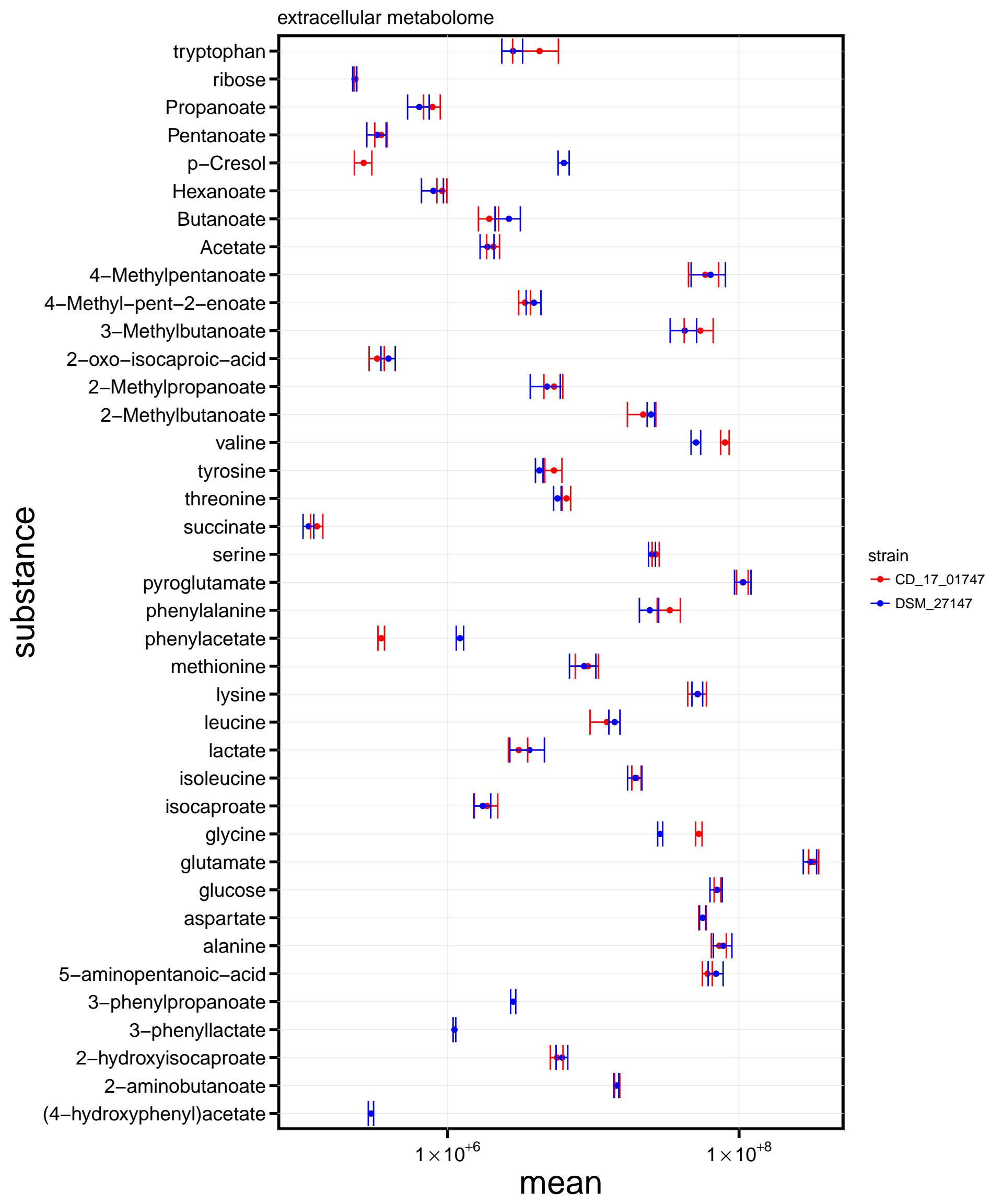

Supplement: FIGURE S6 — A comparison of extracellular metabolite profiles from isolates CD-17-01474 and DSM 27147. Mean normalized peak areas and 95% confidence intervals are indicated. Some metabolites were not detected for isolate CD-17-01474. [file Image_6.PDF]
